# Transcriptome-wide mining, characterization, and development of microsatellite markers in *Lychnis kiusiana* (Caryophyllaceae)

**DOI:** 10.1186/s12870-018-1621-x

**Published:** 2019-01-08

**Authors:** Seongjun Park, Sungwon Son, Myungju Shin, Noriyuki Fujii, Takuji Hoshino, SeonJoo Park

**Affiliations:** 1 Institute of Natural Science, Yeungnam University, Gyeongsan, Gyeongbuk 38541 South Korea; 2 Department of Life Sciences, Yeungnam University, Gyeongsan, Gyeongbuk 38541 South Korea; 3 Plant Conservation Division, Korea National Arboretum, Pocheon, Gyeonggi 11186 South Korea; 4 Department of Biological Science, Graduate School of Science and Technology, Kumamoto University, Chuo-ku, Kumamoto, 860-8555 Japan; 5 Faculty of Biosphere-Geosphere Science, Okayama University of Science, Kita-ku, Okayama, 700-0005 Japan

**Keywords:** *Lychnis*, RNA-seq, Single-nucleotide polymorphisms, Transcriptomic SSRs

## Abstract

**Background:**

*Lychnis kiusiana* Makino is an endangered perennial herb native to wetland areas in Korea and Japan. Despite its conservational and evolutionary significance, population genetic resources are lacking for this species. Next-generation sequencing has been accepted as a rapid and cost-effective solution for the identification of microsatellite markers in nonmodel plants.

**Results:**

Using Illumina HiSeq 2000 sequencing technology, we assembled 67,498,600 reads into 91,900 contigs and identified 11,403 microsatellite repeat motifs in 9563 contigs. A total of 4510 microsatellite-containing transcripts had Gene Ontology (GO) annotations, and Kyoto Encyclopedia of Genes and Genomes (KEGG) analysis identified 124 pathways with significant scores. Many microsatellites in the *L. kiusiana* leaf transcriptome were linked to genes involved in the plant response to light intensity, salt stress, temperature stimulus, and nutrient and water deprivation. A total of 12,486 single-nucleotide polymorphisms (SNPs) were identified on transcripts harboring microsatellites. The analysis of nucleotide substitution rates for 2389 unigenes indicated that 39 genes were under strong positive selection. The primers of 6911 microsatellites were designed, and 40 of 50 selected primer pairs were consistently and successfully amplified from 51 individuals. Twenty-five of these were polymorphic, and the average number of alleles per SSR locus was 6.96, with a range from 2 to 15. The observed and expected heterozygosities ranged from 0.137 to 0.902 and 0.131 to 0.827, respectively, and locus-specific *F*_IS_ estimates ranged from − 0.116 to 0.290. Eleven of the 25 primer pairs were successfully amplified in three additional species of *Lychnis*: 56% in *L. wilfordii*, 64% in *L. cognata* and 80% in *L. fulgens*.

**Conclusions:**

The transcriptomic SSR markers of *Lychnis kiusiana* provide a valuable resource for understanding the population genetics, evolutionary history, and effective conservation management of this species. Furthermore, the identified microsatellite loci linked to the annotated genes should be useful for developing functional markers of *L. kiusiana*. The developed markers represent a potentially valuable source of transcriptomic SSR markers for population genetic analyses with moderate levels of cross-taxon portability.

**Electronic supplementary material:**

The online version of this article (10.1186/s12870-018-1621-x) contains supplementary material, which is available to authorized users.

## Background

Microsatellites, or simple sequence repeats (SSRs), are tandem repeats of mono- to hexa-nucleotide sequence motifs that generally vary in length between five and 40 repeats. They are codominant, abundant, multiallelic and locus-specific markers and are found at high frequency in eukaryotic genomes [1, 2]. Microsatellites are powerful markers for population genetic studies on breeding systems, genetic diversity, and conservation genetics [3, 4]. However, traditional approaches used to identify microsatellite markers are usually time-consuming, labor-intensive, and costly processes [4]. Recently, next-generation sequencing (NGS) has been accepted as a rapid and cost-effective solution for the identification of microsatellite markers in nonmodel plants [5, 6]. Microsatellites can be identified in both genomic and transcriptomic sequences with contrasting features. For example, genomic SSRs have an uncertainty associated with protein-coding genes, while transcriptomic SSRs are potentially linked with protein-coding genes and their untranslated regions (UTRs) [4]. Genomic SSRs are highly polymorphic and are randomly dispersed throughout the genome, while transcriptomic SSRs exhibit relatively low polymorphism and occur infrequently [4, 7]. However, transcriptomic SSRs facilitate the understanding of their association with functional genes or phenotypic variation [8]. Transcriptomic SSRs are transferable among closely related species, whereas genomic SSRs have relatively low transferability [4]. Furthermore, transcriptomic SSRs may play an important role in adaptive evolution by generating genetic variation [9].

The angiosperm genus *Lychnis* (Caryophyllaceae) is well studied with respect to the genetic effects of habitat fragmentation on population structure [10–12]. Many *Lychnis* species are drought-tolerant perennials, but some of them require water to survive. Previous studies have shown that drought stress, mating history and nutrient availability influence inbreeding depression in *Lychnis* populations [13, 14]. This genus exhibits diversity in their sexual and mating systems, breeding system and host-pathogen dynamics, sex chromosome evolution, genomic conflict and speciation, and biological invasions [15]. *Lychnis kiusiana* Makino is an endangered perennial herb native to wetland areas in Korea and Japan [16]. The loss of wet meadow habitats has resulted in decreases in the size and number of populations of this species [17]. Fragmentation of habitats may influence the breeding system and may lead to a reduction in genetic variation, resulting in diminished population capacity to adapt to environmental changes [18–21]. Information on genetic diversity is important for planning the management of *L. kiusiana*. Despite its conservational and evolutionary significance, population genetic resources are lacking for *L. kiusiana*. Only five polymorphic genomic SSR markers have been identified using traditional approaches, and an applied genetic diversity study has been conducted in Japanese populations of the species [17]. However, enriched genomic resources and genetic markers are needed to interpret genetic variation within populations and to investigate the genetic diversity of *L. kiusiana*. Transcriptomic sequencing has been applied for mining microsatellite markers in related species, i.e., *Silene vulgaris* [22], and the results indicated that transcriptomic SSRs provide potential for genomic and population genetic approaches.

In this study, we developed microsatellites from the *L. kiusiana* transcriptome and characterized their frequency, distribution, and function, which promotes an understanding of microsatellite evolution in the *L. kiusiana* transcriptome. We estimated nonsynonymous (*d*_N_) and synonymous (*d*_S_) substitution rates of open reading frames (ORFs) that contained at least one single-nucleotide polymorphism (SNP). In addition, we designed primers for amplifying microsatellite loci and validated the availability of selected primers. Our results offer a valuable resource for studies of population genetics in *L. kiusiana* as well as three other *Lychnis* species.

## Results

### Characteristics of microsatellites in the *Lychnis kiusiana* transcriptome

Using the Illumina HiSeq 2000 sequencing technology tool, we assembled 67,498,600 reads into 91,900 contigs with an average length of 800 bp. A total of 11,403 microsatellite repeat motifs were identified from 9563 contigs (10.4%; Additional file 1: Table S1). Of the total 11,403 SSRs, tri-nucleotide microsatellites were the most abundant motif (42.2%), followed by mono- (35.3%), di- (13.7%), hexa- (9.2%), penta- (3.6%), and tetra-nucleotide (1.7%) types (Additional file 2: Figure S1). A/T motifs (97.7%) were the most abundant in mono-nucleotide repeats (Additional file 2: Figure S1). With respect to the di-nucleotide motif, AG/CT was the most abundant type, with a total of 800 (51.4%), while there was only one CG/GC. Ten tri-nucleotide motif types were identified with AAT/ATT accounting for approximately 21.4%, while CCG/CGG only accounted for 1.7% (Additional file 2: Figure S1). The hexa-nucleotide motif accounted for the highest number (77) across all types (Additional file 2: Figure S1).

The average length of the microsatellites from the *L. kiusiana* transcriptome was 19.75 bp (Additional file 2: Figure S2A). The length variation of microsatellites was significantly affected by the repeat motif size (Kruskal-Wallis rank sum test, *p* < 1 × 10^− 15^; Additional file 2: Figure S2B). The length differences between each motif size class were statistically significant (pairwise Wilcoxon rank-sum test, *p* < 1 × 10^− 15^ after Bonferroni correction; Additional file 2: Figure S2B), except for two comparisons between mono- and di-nucleotide and between tetra- and penta-nucleotide motifs. The mono-nucleotide motifs had the shortest average length (16.7 bp), while hexa-nucleotide motifs were the longest, with an average of 27.4 bp. The longest microsatellite identified was 96 bp, which was composed of a 16-fold repetition of a hexa-nucleotide motif.

### Microsatellite distribution in the *Lychnis kiusiana* transcriptome

**Fig. 1:**
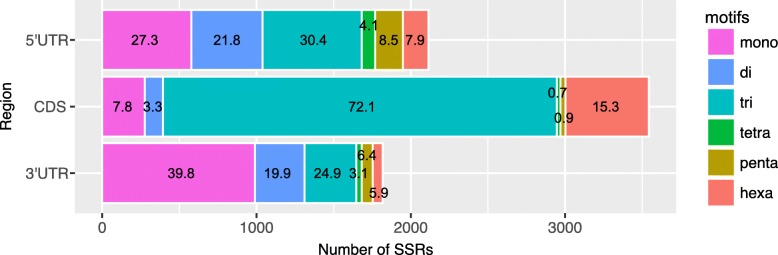
Frequencies of SSRs in different genic regions

The distribution of microsatellites in the 5′ UTR, CDS region, and 3′ UTR was investigated. Of 11,403 microsatellites, 2115, 3543 and 1818 were located in the 5′ UTR, CDS region, and 3′ UTR, respectively (Additional file 1: Table S2). The remaining 3927 microsatellites were excluded from analyses because the transcripts lacked information to determine the CDS region. The frequency of microsatellites in the CDS region was similar to that in the UTRs (Additional file 1: Table S2). However, the frequencies of the motif size classes were significantly affected by the location (Kruskal-Wallis rank sum test, *p* < 1 × 10^− 15^; Fig. 1). Microsatellites located in the CDS regions were mostly tri-nucleotide motifs (72.1%). Mono- and di-nucleotide microsatellites dominated in the UTRs, showing that the proportions of mono- (mean 39.8%) and di-nucleotide motifs (mean 19.9%) in the UTRs were much higher than those in the CDS regions (Fig. 1). The mean lengths of microsatellites differed significantly between the CDS regions and each UTR (Kruskal-Wallis rank sum test, *p* < 1 × 10^− 15^; Additional file 2: Figure S3A). The mean microsatellite lengths of only two motif size classes were significantly affected by location (Kruskal-Wallis rank sum test, mono-: *p* < 1 × 10^− 13^, di-: *p* = 0.009301; Additional file 2: Figure S3B).

### Functional annotation of genes containing microsatellites

**Fig. 2:**
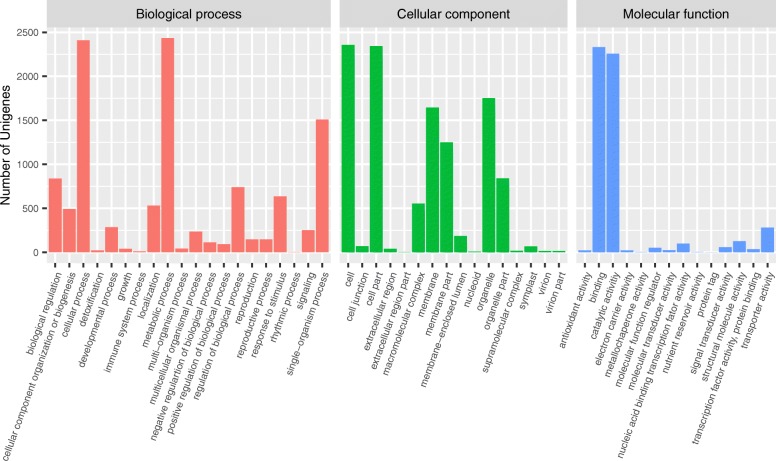
Classification of genes containing microsatellite loci based on the Gene Ontology (GO) annotation

**Fig. 3:**
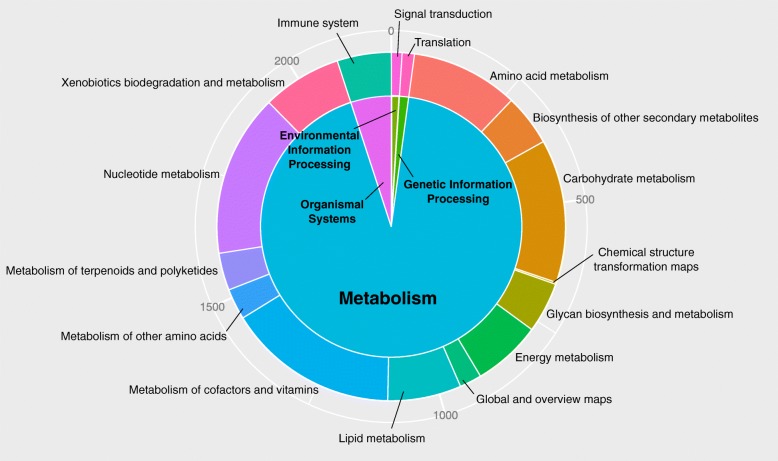
KEGG pathways involved the genes containing microsatellites

Gene Ontology (GO) assignment was used to classify the transcripts according to their function. Of the 9563 contigs in *L. kiusiana*, 6560 (68.6%) had blast hits against “land plants (taxa: 3193)” of the nonredundant (nr) database. In total, 4510 (47.2%) microsatellite-containing transcripts had GO annotations and were categorized into three functional groups and 50 subgroups (Fig. 2). *Cellular* (2401) and *metabolic processes* (2429) were the top two subgroups that involved the most genes in the “biological process” group. In the “cellular component” group, the *cell* (2259) and *cell parts* (2246) were the top two subgroups that involved the most genes. Two subgroups, *catalytic activity* (2241) and *binding* (2239), involved the most genes among the “molecular function” group. A suite of transcripts was annotated as transcription factors (TFs) that contain typical DNA binding motifs, such as AP2/ERF, basic leucine zipper (bZIP), MADS-box, MYB, and WRKY (Additional file 1: Table S3). The functions of microsatellite-containing transcripts were further surveyed by KEGG pathway analysis. The results showed that the transcripts were involved in a total of 124 pathways (Additional file 1: Table S4). The KEGG pathways, including metabolism (92.9%), organismal systems (5.0%), genetic information processing (1.1%), and environmental information processing (1.0%), were grouped into 16 functional categories (Fig. 3). The most represented pathways included purine, pyrimidine and thiamine metabolism, biosynthesis of antibiotics, aminobenzoate degradation, the T-cell receptor signaling pathway, Th1 and Th2 cell differentiation, and starch and sucrose metabolism (Additional file 1: Table S4).

### Identification of selection signatures in transcriptomic SSR markers

**Fig. 4:**
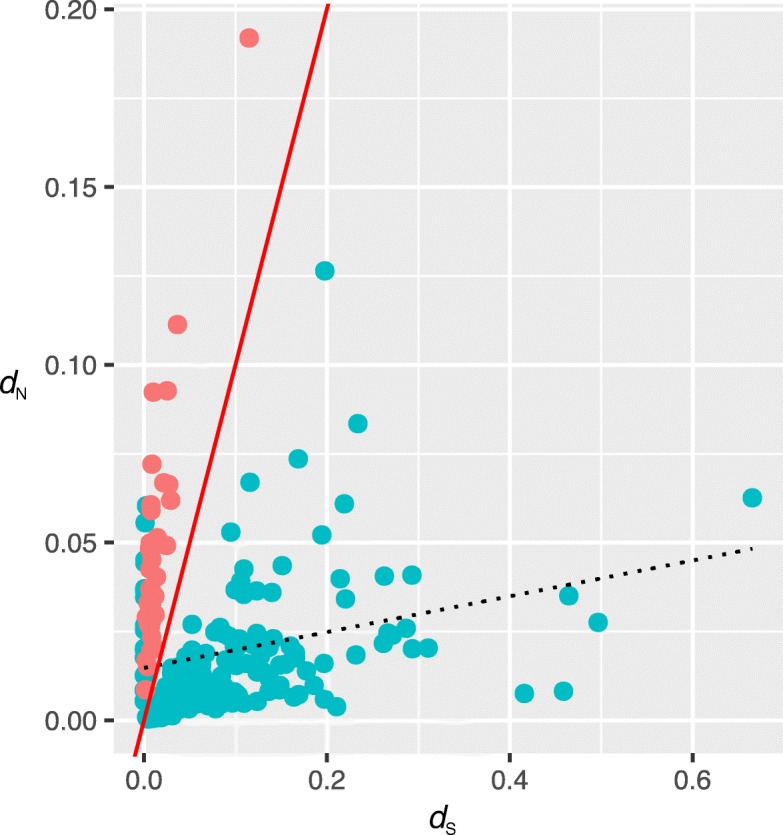
Correlation of synonymous and nonsynonymous substitution rates of SNPs. The solid red line indicates the *d*_N_/*d*_S_ ratio is equal to one. The dashed line represents the regression, which was analyzed using *d*_N_ and *d*_S_ for all unigenes. The red circles represent unigenes with *d*_N_/*d*_S_ ratios > 1

To identify transcriptomic SSR markers under selection, we calculated SNPs on transcripts and investigated their effect on nucleotide substitution rates. A total of 67,498,600 reads were mapped onto the 9563 transcripts that contained microsatellites, generating 12,486 SNPs after quality control and filtration. The proportions of transition substitutions were 30.5% for A/G and 29.9% for C/T, compared with smaller proportions of transversion for A/C (9.5%), A/T (13.1%), C/G (7.3%), and G/T (9.7%). Among all SNPs detected, 3087 were in the putative 2389 ORF regions, of which 1943 were nonsynonymous (62.9%) and 1144 were synonymous (37.1%). We estimated nonsynonymous (*d*_N_) and synonymous (*d*_S_) substitution rates of the ORFs that contained at least one SNP. The results showed that the *d*_N_/*d*_S_ ratios of 39 ORFs were greater than one, indicating that the loci are putatively under positive selection (Fig. 4). Investigation of these ORFs may represent genes with interesting evolutionary histories that have led to high levels of accumulated polymorphism. Functional annotations for 39 ORFs showed that some of the ORFs included genes encoding Cullins, ABC transporters, serine/threonine-protein kinase SRK2E, and synaptotagmin-5 (Additional file 1: Table S5).

### Validation of transcriptomic SSR markers

**Fig. 5:**
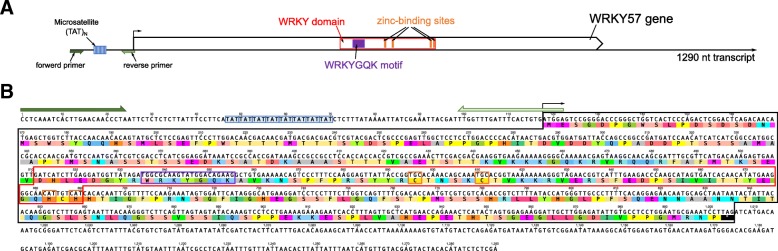
The *LKI09* transcriptomic SSR. **a**. Schematic diagram of the *LKI09* transcriptomic SSR surrounding the WRKY57 gene from *L. kiusiana*. Dark and bright green arrows indicate the forward primer and reverse primer, respectively. The blue box indicates a microsatellite. The red box indicates a conserved domain (WRKY). Purple and orange boxes indicate the well-known WRKYGQK motif and a zinc-binding site, respectively [29]. **b**. DNA and amino acid sequences of the nuclear-encoded WRKY conserved domain, including the UTRs. Colored boxes indicate primers, microsatellites, conserved domains, motifs and zinc-binding sites corresponding to A. Asterisks indicate stop codons

The primers of 6911 microsatellites were designed based on 9563 transcripts containing microsatellites. Apart from transcriptomic SSR markers under selection, a total of 50 pairs of primers were selected (except mononucleotide repeats). Ten primer pairs gave no amplicon, but 40 pairs were consistently and successfully amplified from 51 individuals of the Minamioguni (MG, Kumamoto, Japan) population. Of the 40 primer pairs, 38 produced an amplicon of the expected size, whereas the others produced amplicons larger than the expected size. Twenty-five microsatellite loci were polymorphic and were successfully genotyped in the 51 individuals of MG, generating a total of 174 alleles. The average number of alleles per SSR locus was 6.96 (Table 1). The estimated null allele frequency for *LKI20* was greater than 0.1 (Table 1), and MICRO-CHECKER also indicated a higher frequency of null alleles at the same locus. No significant deviation from Hardy-Weinberg equilibrium (HWE) was found except for one (*LKI20*), following the correction based on the false discovery rate (*p* < 0.05). A linkage disequilibrium (LD) test revealed that most of the loci were in linkage equilibrium except three pairs of loci (*LKI05* and *LKI17*, *LKI16* and *LKI17*, and *LKI20* and *LKI34*) (Additional file 2: Figure S4). Observed (*H*_O_) and expected (*H*_E_) heterozygosities ranged from 0.1373 to 0.9020 and 0.1314 to 0.8270, respectively, and locus-specific *F*_IS_ estimates ranged from − 0.116 (*LKI29*) to 0.290 (*LKI20*). The positive *F*_IS_ value of *LKI20* was significantly higher than zero (Wilcoxon signed-rank test; *p* < 0.05). The polymorphism information content (PIC) of 25 SSR markers ranged from 0.126 to 0.798. At the population level, the MG population exhibited high genetic diversity (*H*_O_ = 0.6455, *H*_E_ = 0.6617) and a low inbreeding coefficient (*F*_IS_ = 0.023). The mean value of the Garza-Williamson (*G-W*) index for the MG population was 0.8278. Twenty-one of the identified microsatellite markers had GO annotations (Additional file 1: Table S6). In particular, *LKI09* which exhibited a high level of polymorphism (Table 1) was associated with WRKY transcription factor 57 (WRKY57) (Fig. 5).

**Table 1:** Summary statistics for 25 polymorphic microsatellite loci developed for *Lychnis kiusiana*. Number of alleles (*N*_A_), observed (*H*_O_) and expected (*H*_E_) heterozygosities, inbreeding coefficient (*F*_IS_), Hardy-Weinberg equilibrium (HWE), and polymorphism information content (PIC). Significant values after false discovery rate correction are indicated with asterisks (* *p* < 0.05, ** *p* < 0.01)

Locus	Size range (bp)	*N*_A_	*F*null	*H*_O_	*H*_E_	*F*_IS_	HWE	PIC
*LKI04*	201–217	4	0.00000	0.37255	0.36905	−0.010	0.2921	0.341
*LKI05*	230–260	6	0.02708	0.68627	0.72821	0.058	0.0108*	0.672
*LKI08*	211–229	6	0.08018	0.52941	0.64221	0.177	0.0942	0.576
*LKI09*	119–173	15	0.03127	0.76471	0.77927	0.019	0.0253*	0.755
*LKI10*	146–176	7	0.00000	0.82353	0.73986	−0.114	0.3221	0.695
*LKI12*	124–157	10	0.00000	0.84314	0.80431	−0.049	0.8835	0.767
*LKI15*	198–240	8	0.01274	0.78431	0.82702	0.052	0.7256	0.798
*LKI16*	222–240	5	0.02202	0.68627	0.68744	0.002	0.1274	0.625
*LKI17*	183–219	12	0.00013	0.72549	0.75306	0.037	0.6855	0.718
*LKI20*	241–268	8	0.12785	0.54902	0.77053	0.290^†^	0.0006**	0.742
*LKI28*	158–188	8	0.00000	0.82353	0.75791	−0.074	0.0502	0.725
*LKI29*	211–253	10	0.00000	0.90196	0.80897	−0.116	0.0756	0.777
*LKI32*	226–244	4	0.00003	0.64706	0.64007	−0.011	0.5823	0.572
*LKI33*	126–152	8	0.05123	0.64706	0.75908	0.149	0.0593	0.711
*LKI34*	181–208	9	0.05066	0.7451	0.82411	0.097	0.0215*	0.797
*LKI35*	185–197	5	0.00001	0.56863	0.56824	−0.046	0.6829	0.414
*LKI38*	151–169	6	0.00000	0.7451	0.70511	−0.057	0.6523	0.653
*LKI39*	227–236	4	0.00002	0.56863	0.56824	−0.001	0.3841	0.510
*LKI41*	195–210	5	0.00679	0.70588	0.70258	−0.005	0.5343	0.649
*LKI42*	127–130	2	0.05875	0.39216	0.48146	0.187	0.2406	0.363
*LKI43*	197–203	3	0.00001	0.13725	0.13143	−0.045	1.0000	0.126
*LKI44*	245–248	2	0.00005	0.5098	0.50476	−0.010	1.0000	0.375
*LKI45*	168–192	9	0.00000	0.84314	0.76199	−0.108	0.8721	0.718
*LKI46*	213–237	7	0.00000	0.60784	0.60435	−0.006	0.2555	0.559
*LKI48*	251–279	10	0.07888	0.62745	0.70879	0.116	0.0291*	0.656
Total		174						
Mean		6.96		0.6455	0.6617	0.023		0.6118

To examine cross-species transferability, 25 newly developed microsatellite markers were tested on the other *Lychnis* species (*L. cognate*, *L. fulgens*, and *L. wilfordii*). Eleven markers were successfully amplified in all three *Lychnis* species (44%; Table 2). Twenty and 16 loci were amplified in *L. fulgens* and *L. cognate*, respectively, while only 14 loci were amplified in *L. wilfordii* (Table 2).

**Table 2:**
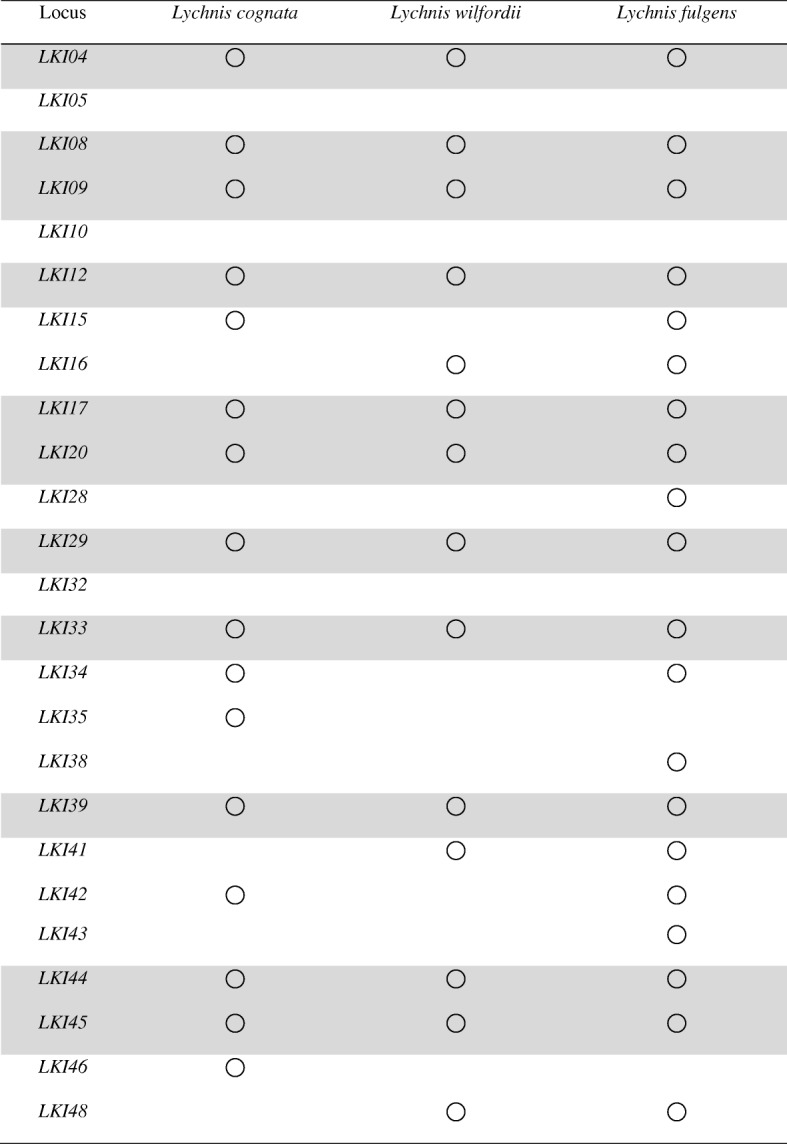
Amplification success of 25 microsatellites developed for *Lychnis kiusiana* across three other *Lychnis* species**.** Gray shading indicates a locus that was successfully amplified in all three *Lychnis* species

## Discussion

Deep transcriptome sequencing technologies are rapid and cost-effective tools to characterize gene content and identify polymorphic markers in nonmodel plants [6]. In this study, we generated a leaf transcriptome from the angiosperm *L. kiusiana* and characterized microsatellites in the transcriptome. The results showed that microsatellites were present in a small proportion of the transcripts (10.4%), which is higher than the estimation that 2–5% transcripts contain microsatellites [23]. These transcripts are involved in a wide range of potential functions, such as *cellular* and *metabolic processes*, *cell* and *cell parts*, and *catalytic activity* and *binding* (Fig. 2). The transcriptomic SSR markers provide a valuable resource for the development of genetic markers in *L. kiusiana* and offer a chance to classify the functions of these microsatellite-containing genes. In addition, a moderate level of transferability suggests that the microsatellite loci may be useful for studies of the other *Lychnis* species, although screening of microsatellite markers will be required to test polymorphisms.

Microsatellite distributions in genes, including coding regions, UTRs, and introns, are nonrandom and strongly biased [24]. Our results showed that the repeat motif size classes were significantly affected by location in the CDS regions or UTRs (Fig. 1). Of the six motif size classes, tri- and hexa- nucleotides were found in CDS regions with a high frequency (87.4%) because these types of microsatellites are less likely to cause frameshift mutations. In *L. kiusiana*, 5′-UTRs and 3′-UTRs contain more mono- and di-nucleotide motifs than coding regions. The 5′-UTRs contained more tri-nucleotide motifs than the 3′-UTRs in *L. kiusiana* (30.4% vs 24.9%). This is because microsatellite variations in 5′-UTRs influence gene expression and lead to protein adaptation [24]. Microsatellite length also reflects the effect of evolution and selection on microsatellite loci development. The *Lychnis* transcriptomic SSRs in UTRs were much longer than those in CDS regions (Additional file 2: Figure S3), reflecting higher evolutionary constraints on the microsatellites in the CDS regions than in UTRs.

Microsatellite repeat motif variation can influence gene regulation, transcription, translation, and protein function [9]. We found that many microsatellites in the *L. kiusiana* leaf transcriptome were linked to the genes involved in plant response to light intensity, salt stress, temperature stimulus, and nutrient and water deprivation. Some of these transcripts, such as aldehyde oxidase and cytochrome P450, are related to resistance to environmental stresses and insecticides [25, 26]. *Lychnis kiusiana* inhabits wetlands in mountain areas of Korea and Japan [16], and wetland plants are exposed to environmental factors such as salinity, soil anaerobiosis, low nutrient conditions, sediment deficiency, and fluctuating water regimes [27]. Previous studies have shown that inbreeding depression in *Lychnis* populations is affected by drought stress, mating history and nutrient availability [13, 14]. Thus, microsatellites located in these genes may be crucial for the accumulation of adaptive genetic variations that enable *L. kiusiana* to thrive in wetlands of mountain areas.

Transcription factors (TFs) play a crucial role in controlling cellular processes, such as plant growth and development pathways [28]. The *Arabidopsis* and rice genomes contain some transcripts harboring microsatellites that are related to TFs [29]. In the *L. kiusiana* leaf transcriptome, we found that many transcripts harboring microsatellites have transcription factor activity (Additional file 1: Table S3). Interestingly, among the 25 transcriptomic SSR markers, high levels of microsatellite polymorphisms (15 alleles) were detected in the *LKI09* marker linked to a gene encoding probable WRKY57 (Fig. 5 and Table 1). WRKY TFs are key regulators of many processes in plants, including the responses to biotic (e.g., pathogen) and abiotic (e.g., drought and cold) stresses, leaf senescence and seed dormancy, germination and developmental processes [30]. In addition to WRKY TFs, AP2/ERF or MYB TFs also regulate various biological processes, including development, defense, and biotic and abiotic stress responses [31–34]. Many transcripts associated with the AP2/ERF or MYB TF family were identified in the *L. kiusiana* leaf transcriptome (Additional file 1: Table S3). These microsatellites related to TFs may function as an important “tuning knobs” for the expression of genes [35, 36]. Examination of microsatellite polymorphisms in *Lychnis* transcripts linked to TFs requires further study to provide valuable insights into understanding environmental stresses.

For genetic diversity study, including structure analysis, it is critical to identify loci affected by selection in order to exclude them. We applied outlier tests based on detecting SNP loci under selection. Examination of the rate of variation in the ORFs that contained at least one SNP revealed the acceleration of the evolution of some genes, probably to adapt to the extreme environmental stress in wetlands of mountain areas. For example, the *d*_N_/*d*_S_ ratios of two transcripts, Cullins and ABC transporters, were higher than one (Additional file 1: Table S5). Cullins are a family of hydrophobic proteins that provide a scaffold for ubiquitin ligases (E3). E3 is a member of a ubiquitination system that operates in conjunction with an E1 (ubiquitin activating enzyme; UBA) and an E2 (ubiquitin conjugating enzyme; UBC) and functions in regulating abiotic stress (e.g., drought, salinity, cold and nutrient deprivation) responses [37]. In plants, ATP-binding cassette transporters (ABC transporters) are involved in essential aspects of a terrestrial plant’s lifestyle [38]. ABC transporters also play an important role in organ growth, plant nutrition, plant development, response to abiotic stress, and the interaction of the plant with its environment [39].

The genetic variability of microsatellites is extensively exploited in evolutionary studies of a wide variety of plant species [40]. Genetic diversity and fitness play an important role in species conservation, especially in rare plants and small populations [41, 42]. *Lychnis kiusiana* is an endangered perennial herb native to wetland areas, and the sizes and numbers of populations of this species are decreasing [17]. A previous study showed high genetic diversity (mean *H*_E_ = 0.791 and mean *N*_A_ = 12.0) within the seven *L*. *kiusiana* populations based on five genomic SSR markers [17]. Although these novel genomic SSR markers are useful for investigating genetic diversity and molecular-assisted breeding, these markers are still insufficient for genetic population study. Herein, we identified applicable microsatellites from the *L*. *kiusiana* leaf transcriptome, and 25 transcriptomic SSR markers have been proven to be efficient genetic markers. Our results showed that the genetic diversity of the MG population with the 25 transcriptomic SSR markers was higher (*N*_A_ = 6.96 and *H*_E_ = 0.6617), although the five genomic SSRs revealed higher genetic variations (*N*_A_ = 14.2 and *H*_E_ = 0.817) than the transcriptomic SSR in the MG population. This is because genomic SSR markers generally have a higher level of polymorphism compared with transcriptomic SSR markers [7]. Compared with other transcriptomic SSR markers [43–46], the developed *L*. *kiusiana* transcriptomic SSR markers have a high level of genetic diversity. Full understanding of the genetic diversity and structure of *L*. *kiusiana* requires evaluation of genetic variation among the other Korean and Japanese populations. SSR neutrality tests in the populations of *L. kiusiana* are also needed to identify either neutral or outlier loci among the 25 newly developed markers.

Given the high transferability of transcriptomic SSR markers, the microsatellites identified from *L*. *kiusiana* will have wide application in other *Lychnis* species. We applied 25 newly developed transcriptomic SSR markers to three species of the genus *Lychnis* to evaluate the transferability of these markers. The results showed that the transferability ratios were 56% in *L. wilfordii*, 64% in *L. cognate*, and 80% in *L. fulgens*, consistent with the estimation (46.8–100%) for a genus [7]. The high transferability of *L*. *kiusiana* transcriptomic SSRs to *L. fulgens* may be due to a high similarity of the sequences flanking the SSR between two species. In particular, the 14 amplified SSR markers in *L. wilfordii* will be a valuable source for its population genetic analyses because *L. wilfordii* is an endangered species in Korea.

## Conclusions

In this study, we characterized a large number of transcriptome-derived microsatellites from *Lychnis kiusiana*, and functional annotation of microsatellite-containing transcripts provides new insights into the evolution of microsatellites. Several microsatellites in the *L. kiusiana* leaf transcriptome were linked to the genes involved in plant response to biotic and abiotic stresses. The microsatellites located in these genes may be crucial for *L. kiusiana* to evaluate adaptive genetic variations to adapt to wetlands in mountain areas. Our results showed that the identified microsatellite loci that have high allele numbers and heterozygosity could provide genetic markers in *L. kiusiana* populations. Furthermore, many microsatellites were transferable across the other species of the genus.

## Methods

### Sample collection, RNA extraction and sequencing

Leaf samples of *Lychnis kiusiana* were collected from a single individual in the Korea National Arboretum, South Korea. Total RNA was extracted from fresh leaf tissue (100 mg) using the methods of Ghawana et al. [47] and was treated with DNase I (Invitrogen, California, USA). The *Lychnis* RNA (29.7 μg) was sequenced on the Illumina HiSeq 2000 platform at LabGenomics (Seongnam, South Korea), generating 6 Gb of 100 bp of paired-end reads.

### Transcriptome assembly and microsatellite identification

RNA sequence reads were assembled with Trinity v2.2.0 [48] on a 12-core 3.33-GHz Linux Workstation with 192 GB memory. These assembled contigs were used to detect microsatellite loci using the MIcroSAtellite identification tool (MISA) [49] with the following criteria: mono-nucleotide repeat motifs with at least 12 repeats, di-nucleotide repeat motifs with at least six repeats, tri- and tetra-nucleotide repeat motifs with at least five motifs, and penta- and hexa-nucleotide repeat motifs with at least four repeats. The criterion for compound microsatellites was that the interval between two repeat motifs was shorter than 100 nt. The assembled contigs were deposited in the Dryad Digital Repository (doi:10.5061/dryad.rc47tt4).

To investigate the distribution of microsatellites in the *L. kiusiana* leaf transcriptome, candidate coding regions within transcript sequences were identified using TransDecoder v3.0.1 (http://transdecoder.github.io/). The location of microsatellite was determined based on the predicted CDS region, 5′ UTR and 3′ UTR. Characteristics of motif type were determined and compared with each other among microsatellite loci located in the CDS region, 5′ UTR and 3′ UTR.

### Functional annotation

To understand the possible function of microsatellites, all the transcripts harboring a microsatellite were searched against the GenBank nr protein database using BLASTx with an *e*-value cut-off of 10^− 5^ using Blast2GO v4.1.9 [50]. Gene ontology (GO) and Kyoto Encyclopedia of Genes and Genomes (KEGG) were used.

### SNP identification and estimation of synonymous and nonsynonymous sites

To identify putative SNPs in the transcripts containing SSRs, all reads were mapped onto the assembled transcripts using Bowtie v2.2.9 [51] with default parameters. The SAMtools “mpileup” utility and BCFtools [52] were used to identify SNPs. The SNPs with a read depth ≥ 10 and mapping quality ≥20 were retained. To detect transcriptomic SSRs under directional selection, nonsynonymous (*d*_N_) and synonymous (*d*_S_) rates of ORFs that contained at least one SNP were estimated by KaKs_Calculator v2.0 [53]. A *d*_N_/*d*_S_ ratio greater than one suggests positive selection and less than one indicates purifying selection [54]. We estimated that ORFs with *d*_N_/*d*_S_ > 1 and *p* < 0.05 (Fisher’s exact test) were under positive selection.

### Microsatellite detection and genotyping

Previously extracted genomic DNA [17] for 51 individuals collected from the population of Minamioguni (MG, Kumamoto, Japan) was used. To explore amplification and polymorphism of the microsatellite loci identified in the *L. kiusiana* leaf transcriptome, the primers were designed based on the sequences flanking the microsatellite loci using Primer3 [55]. Apart from transcriptomic SSR markers under selection, 50 primer pairs were selected from the transcripts with a large number of repeat sizes and then used to amplify the microsatellite loci. Polymerase chain reaction (PCR) was performed in a 25-μl reaction volume containing 10 ng genomic DNA, 0.2 mM of each dNTP, 0.25 μM of each primer, 5 μl 10× *h*-*Taq* reaction buffer, and 0.25 units of *h*-*Taq* polymerase (Solgent Co., Daejeon, South Korea). PCR was performed under the following conditions: an initial denaturation at 95 °C for 15 min followed by 30 cycles each of 95 °C for 20 s, 58 °C or 60 °C for 40 s, and 72 °C for 30 s, and a final extension at 72 °C for 5 min. Electrophoresis of the amplified fragments was performed on 2% agarose gel to confirm the success of amplification and polymorphism. Forward or reverse primers selected for the population genetic analyses (Table S7) were labeled with 6-FAM, HEX, or TAMRA fluorescent dyes (Macrogen Inc.; Seoul, South Korea). For fragment analyses, PCR products amplified with fluorescent primers were genotyped on an ABI 3730xl DNA Analyzer (Applied Biosystems, California, USA) at Macrogen Inc. (Seoul, South Korea).

Microsatellite polymorphisms of *L. kiusiana* were calculated based on the average number of alleles per locus (*N*_A_), observed (*H*_O_) and expected (*H*_E_) heterozygosities and inbreeding coefficient (*F*_IS_) [56] in FSTAT 2.9.3 [57] and in GENEPOP v4.2 [58]. Departure from genotypic proportions expected under Hardy-Weinberg equilibrium (HWE) was examined by Fisher’s exact test using permutations [59] implemented in GENEPOP. The linkage disequilibrium between loci was also detected using Fisher’s exact test under the Markov chain algorithm implemented in GENEPOP. PIC was calculated using CERVUS 3.0 [60]. Critical significance levels were adjusted for multiple comparisons using the false discovery rate [61]. The statistical analysis was conducted using R v.3.3.3 [62].

### Cross-species amplification

Three species of the genus *Lychnis* (*L. cognate*, *L. fulgens,* and *L. wilfordii*) were chosen to evaluate the transferability of these newly developed microsatellite markers to other related species. Total genomic DNA was isolated from herbarium specimens using the methods of Allen et al. [63]. PCR was performed as described above with the primer pairs. Electrophoresis of PCR products was performed on 2% agarose gel to check the success of amplification. The experiments were repeated three times with the same controls to confirm reproducibility and consistency.

## Additional files

Additional file 1: **Table S1.** General information for the microsatellite analysis. **Table S2**. Distribution and characteristics of the microsatellites in different transcript regions. **Table S3.** Results of the KEGG pathway analysis. **Table S4.** Blast results of 9563 transcripts that contain SSRs in *Lychnis* leaf transcriptome. **Table S5.** Blast results of the 39 ORFs showing positive selection (*d*_N_/*d*_S_ > 1). **Table S6.** Blast results of the 25 newly developed transcriptomic SSR markers. **Table S7.** NCBI accession numbers, primer sequences and characterization of the 25 microsatellite loci developed for *Lychnis kiusiana*. (DOCX 1498 kb)

Additional file 2: **Figure S1.** The distributions of the major repeat types in the *Lychnis kiusiana* leaf transcriptome. **Figure S2.** Box plots of the sizes of different repeat motifs. **Figure S3.** Box plots of the sizes of different repeat motifs in different genic regions. **Figure S4**. Linkage disequilibrium (LD) map for the 25 transcriptomic SSR markers. (PDF 556 kb)

## Abbreviations

*d*_N_: Number of substitutions per nonsynonymous site
*d*_S_: Number of substitutions per synonymous site
*F*_IS_: the inbreeding coefficient within individuals relative to the subpopulation
GO: Gene Ontology
*H*_E_: expected heterozygosity
*H*_O_: observed heterozygosity
HWE: Hardy-Weinberg equilibrium
KEGG: Kyoto Encyclopedia of Genes and Genomes
LD: Linkage disequilibrium
ORF: Open reading frame
PIC: Polymorphism information content
SNP: Single-nucleotide polymorphism
SSR: Simple sequence repeat
