# Supplementary material for: Transcriptome-wide mining, characterization, and development of microsatellite markers in Lychnis kiusiana (Caryophyllaceae)
Source: BMC Plant Biol. 2019 Jan 8;19:14. doi: 10.1186/s12870-018-1621-x (PMC6325733; doi:10.1186/s12870-018-1621-x)
Supplement: Supplementary file 1 — Table S1. General information for the microsatellite analysis. Table S2. Distribution and characteristics of the microsatellites in different transcript regions. Table S3. Results of the KEGG pathway analysis. Table S4. Blast results of 9563 transcripts that contain SSRs in Lychnis leaf transcriptome. Table S5. Blast results of the 39 ORFs showing positive selection (dN/dS > 1). Table S6. Blast results of the 25 newly developed transcriptomic SSR markers. Table S7. NCBI accession numbers, primer sequences and characterization of the 25 microsatellite loci developed for Lychnis kiusiana. (DOCX 1498 kb) [file 12870_2018_1621_MOESM1_ESM.docx]

Table S1. General information for the microsatellite analysis.

| Items | Number |
| --- | --- |
| Total number of sequences examined | 91,900 |
| Total size of the examined sequences (bp) | 73,574,123 |
| Total number of identified SSRs | 11,403 |
| Number of SSR-containing sequences | 9,563 |
| Number of sequences containing more than 1 SSR | 1,503 |
| Number of SSRs present in compound formation | 962 |

Table S2. Distribution and characteristics of the microsatellites in different transcript regions.

| Region | Mono | Di | Tri | Tetra | Penta | Hexa | Total |
| --- | --- | --- | --- | --- | --- | --- | --- |
| CDS | 275 | 118 | 2553 | 24 | 32 | 541 | 3543 |
| UTRs | 1566 | 784 | 979 | 120 | 252 | 232 | 3933 |
| 5'UTR | 577 | 462 | 643 | 86 | 180 | 167 | 2115 |
| 3'UTR | 989 | 322 | 336 | 34 | 72 | 65 | 1818 |

Table S3. Result of the KEGG pathway analysis.

| KEGG | Categories | Pathways | Seqs | Enzs |
| --- | --- | --- | --- | --- |
| 1. Metabolism | 1.0 Global and overview maps | Biosynthesis of antibiotics | 140 | 76 |
|  | 1.1 Carbohydrate metabolism | Amino sugar and nucleotide sugar metabolism | 30 | 15 |
|  |  | Ascorbate and aldarate metabolism | 14 | 4 |
|  |  | Butanoate metabolism | 7 | 4 |
|  |  | C5-Branched dibasic acid metabolism | 5 | 3 |
|  |  | Citrate cycle (TCA cycle) | 5 | 6 |
|  |  | Fructose and mannose metabolism | 16 | 8 |
|  |  | Galactose metabolism | 17 | 10 |
|  |  | Glycolysis / Gluconeogenesis | 37 | 15 |
|  |  | Glyoxylate and dicarboxylate metabolism | 22 | 14 |
|  |  | Inositol phosphate metabolism | 8 | 4 |
|  |  | Pentose and glucuronate interconversions | 17 | 7 |
|  |  | Pentose phosphate pathway | 21 | 12 |
|  |  | Propanoate metabolism | 13 | 8 |
|  |  | Pyruvate metabolism | 33 | 20 |
|  |  | Starch and sucrose metabolism | 47 | 23 |
|  | 1.2 Energy metabolism | Carbon fixation in photosynthetic organisms | 29 | 15 |
|  |  | Carbon fixation pathways in prokaryotes | 16 | 10 |
|  |  | Methane metabolism | 19 | 10 |
|  |  | Nitrogen metabolism | 10 | 5 |
|  |  | Oxidative phosphorylation | 18 | 6 |
|  |  | Sulfur metabolism | 11 | 7 |
|  | 1.3 Lipid metabolism | Alpha-Linolenic acid metabolism | 10 | 7 |
|  |  | Arachidonic acid metabolism | 4 | 2 |
|  |  | Biosynthesis of unsaturated fatty acids | 12 | 5 |
|  |  | Cutin, suberine and wax biosynthesis | 2 | 2 |
|  |  | Ether lipid metabolism | 9 | 5 |
|  |  | Fatty acid biosynthesis | 21 | 8 |
|  |  | Fatty acid biosynthesis | 13 | 6 |
|  |  | Fatty acid elongation | 5 | 3 |
|  |  | Glycerolipid metabolism | 25 | 12 |
|  |  | Glycerophospholipid metabolism | 23 | 13 |
|  |  | Linoleic acid metabolism | 2 | 1 |
|  |  | Primary bile acid biosynthesis | 1 | 1 |
|  |  | Sphingolipid metabolism | 8 | 5 |
|  |  | Steroid biosynthesis | 8 | 5 |
|  |  | Steroid hormone biosynthesis | 6 | 3 |
|  |  | Synthesis and degradation of ketone bodies | 1 | 1 |
|  | 1.4 Nucleotide metabolism | Purine metabolism | 281 | 16 |
|  |  | Pyrimidine metabolism | 50 | 14 |
|  | 1.5 Amino acid metabolism | Alanine, aspartate and glutamate metabolism | 19 | 7 |
|  |  | Arginine and proline metabolism | 15 | 5 |
|  |  | Arginine biosynthesis | 14 | 8 |
|  |  | Cysteine and methionine metabolism | 21 | 17 |
|  |  | Glycine, serine and threonine metabolism | 17 | 13 |
|  |  | Histidine metabolism | 12 | 3 |
|  |  | Lysine biosynthesis | 12 | 6 |
|  |  | Lysine degradation | 12 | 4 |
|  |  | Phenylalanine metabolism | 27 | 14 |
|  |  | Phenylalanine, tyrosine and tryptophan biosynthesis | 11 | 8 |
|  |  | Tryptophan metabolism | 14 | 5 |
|  |  | Tyrosine metabolism | 23 | 12 |
|  |  | Valine, leucine and isoleucine biosynthesis | 5 | 3 |
|  |  | Valine, leucine and isoleucine degradation | 17 | 8 |
|  | 1.6 Metabolism of other amino acids | Beta-Alanine metabolism | 16 | 6 |
|  |  | Cyanoamino acid metabolism | 15 | 2 |
|  |  | D-Arginine and D-ornithine metabolism | 1 | 1 |
|  |  | D-Glutamine and D-glutamate metabolism | 4 | 2 |
|  |  | Glutathione metabolism | 16 | 7 |
|  |  | Phosphonate and phosphinate metabolism | 2 | 1 |
|  |  | Selenocompound metabolism | 8 | 5 |
|  |  | Taurine and hypotaurine metabolism | 1 | 1 |
|  | 1.7 Glycan biosynthesis and metabolism | Glycosaminoglycan biosynthesis - chondroitin sulfate / dermatan sulfate | 1 | 1 |
|  |  | Glycosaminoglycan biosynthesis - heparan sulfate | 2 | 2 |
|  |  | Glycosaminoglycan degradation | 4 | 2 |
|  |  | Glycosphingolipid biosynthesis - ganglio series | 4 | 2 |
|  |  | Glycosphingolipid biosynthesis - globo and isoglobo series | 3 | 2 |
|  |  | Lipopolysaccharide biosynthesis | 4 | 2 |
|  |  | Mannose type O-glycan biosynthesis | 1 | 1 |
|  |  | N-Glycan biosynthesis | 3 | 1 |
|  |  | Other glycan degradation | 18 | 4 |
|  |  | Various types of N-glycan biosynthesis | 5 | 2 |
|  | 1.8 Metabolism of cofactors and vitamins | Biotin metabolism | 17 | 4 |
|  |  | Folate biosynthesis | 2 | 2 |
|  |  | Lipoic acid metabolism | 1 | 1 |
|  |  | Nicotinate and nicotinamide metabolism | 5 | 3 |
|  |  | One carbon pool by folate | 8 | 4 |
|  |  | Pantothenate and CoA biosynthesis | 5 | 3 |
|  |  | Porphyrin and chlorophyll metabolism | 24 | 9 |
|  |  | Retinol metabolism | 8 | 4 |
|  |  | Riboflavin metabolism | 5 | 3 |
|  |  | Thiamine metabolism | 244 | 5 |
|  |  | Ubiquinone and other terpenoid-quinone biosynthesis | 21 | 8 |
|  |  | Vitamin B6 metabolism | 9 | 5 |
|  | 1.9 Metabolism of terpenoids and polyketides | Biosynthesis of ansamycins | 2 | 1 |
|  |  | Carotenoid biosynthesis | 5 | 2 |
|  |  | Diterpenoid biosynthesis | 15 | 2 |
|  |  | Geraniol degradation | 3 | 2 |
|  |  | Insect hormone biosynthesis | 7 | 1 |
|  |  | Limonene and pinene degradation | 9 | 2 |
|  |  | Sesquiterpenoid and triterpenoid biosynthesis | 4 | 1 |
|  |  | Terpenoid backbone biosynthesis | 26 | 8 |
|  |  | Tetracycline biosynthesis | 2 | 1 |
|  |  | Zeatin biosynthesis | 3 | 3 |
|  | 1.10 Biosynthesis of other secondary metabolites | Aflatoxin biosynthesis | 2 | 1 |
|  |  | Flavone and flavonol biosynthesis | 1 | 1 |
|  |  | Flavonoid biosynthesis | 18 | 5 |
|  |  | Indole alkaloid biosynthesis | 1 | 1 |
|  |  | Isoquinoline alkaloid biosynthesis | 9 | 5 |
|  |  | Monobactam biosynthesis | 6 | 3 |
|  |  | Neomycin, kanamycin and gentamicin biosynthesis | 6 | 2 |
|  |  | Novobiocin biosynthesis | 6 | 4 |
|  |  | Phenylpropanoid biosynthesis | 35 | 10 |
|  |  | Stilbenoid, diarylheptanoid and gingerol biosynthesis | 2 | 2 |
|  |  | Streptomycin biosynthesis | 10 | 4 |
|  |  | Tropane, piperidine and pyridine alkaloid biosynthesis | 9 | 6 |
|  | 1.11 Xenobiotics biodegradation and metabolism | Aminobenzoate degradation | 78 | 3 |
|  |  | Benzoate degradation | 3 | 3 |
|  |  | Caprolactam degradation | 4 | 2 |
|  |  | Chloroalkane and chloroalkene degradation | 9 | 3 |
|  |  | Chloroalkane and chloroalkene degradation | 1 | 1 |
|  |  | Drug metabolism - cytochrome P450 | 15 | 5 |
|  |  | Drug metabolism - other enzymes | 34 | 6 |
|  |  | Ethylbenzene degradation | 1 | 1 |
|  |  | Metabolism of xenobiotics by cytochrome P450 | 12 | 4 |
|  |  | Naphthalene degradation | 1 | 1 |
|  |  | Steroid degradation | 3 | 1 |
|  |  | Styrene degradation | 1 | 1 |
|  | 1.12 Chemical structure transformation maps | Biosynthesis of terpenoids and steroids | 4 | 1 |
| 2. Genetic Information Processing | 2.2 Translation | Aminoacyl-tRNA biosynthesis | 25 | 11 |
| 3. Environmental Information Processing | 3.2 Signal transduction | mTOR signaling pathway | 3 | 1 |
|  |  | Phosphatidylinositol signaling system | 19 | 6 |
| 5. Organismal Systems | 5.1 Immune system | T cell receptor signaling pathway | 62 | 2 |
|  |  | Th1 and Th2 cell differentiation | 48 | 1 |

Table S4. Blast results of 9,563 transcripts that containing SSRs in *Lychnis* leaf transcriptome.

| SeqName | Description | Length | e-Value | sim mean |
| --- | --- | --- | --- | --- |
| TRINITY_DN10005_c0_g1_i1 | nodulin-related 1 | 544 | 1.66E-36 | 70.6 |
| TRINITY_DN10006_c0_g1_i1 | F-box PP2-A15 | 682 | 5.11E-65 | 82.25 |
| TRINITY_DN10006_c0_g2_i1 | F-box PP2-A15 | 1654 | 1.08E-171 | 85.8 |
| TRINITY_DN10016_c0_g1_i1 | probable histone-arginine methyltransferase | 2239 | 0 | 87.75 |
| TRINITY_DN10018_c0_g1_i2 | ---NA--- | 762 |  |  |
| TRINITY_DN10018_c0_g1_i3 | ---NA--- | 520 |  |  |
| TRINITY_DN10029_c0_g1_i1 | 3-epi-6-deoxocathasterone 23-monooxygenase | 2014 | 0 | 82.4 |
| TRINITY_DN1002_c0_g1_i1 | kDa class I heat shock -like | 933 | 5.19E-46 | 59.7 |
| TRINITY_DN10039_c0_g1_i1 | PREDICTED: uncharacterized protein LOC104897219 | 693 | 6.29E-18 | 86.2 |
| TRINITY_DN10040_c0_g1_i1 | calmodulin | 528 | 3.72E-20 | 76.3 |
| TRINITY_DN10041_c0_g1_i1 | CAAX prenyl protease 2 | 1345 | 1.84E-107 | 71.4 |
| TRINITY_DN10043_c0_g1_i1 | xyloglucan endotransglycosylase hydrolase | 1079 | 1.60E-171 | 87.45 |
| TRINITY_DN10057_c0_g1_i1 | hypothetical protein BVRB_7g166330 | 919 | 1.74E-31 | 63.5 |
| TRINITY_DN10073_c0_g1_i1 | glycine-rich cell wall structural 2-like | 636 | 4.27E-12 | 77 |
| TRINITY_DN10073_c1_g1_i1 | ---NA--- | 1130 |  |  |
| TRINITY_DN10076_c0_g1_i1 | ---NA--- | 489 |  |  |
| TRINITY_DN10086_c0_g1_i1 | dof zinc finger -like | 1160 | 1.39E-71 | 54.8 |
| TRINITY_DN10100_c0_g1_i1 | 5 -3 exoribonuclease 3 isoform X2 | 1587 | 5.90E-167 | 64.35 |
| TRINITY_DN10102_c0_g1_i1 | ---NA--- | 732 |  |  |
| TRINITY_DN10102_c0_g2_i1 | ---NA--- | 732 |  |  |
| TRINITY_DN10108_c0_g1_i1 | N-lysine methyltransferase METTL21A | 961 | 3.84E-133 | 83.05 |
| TRINITY_DN10128_c0_g1_i1 | Chaperone binding,ATPase activators | 1002 | 8.20E-107 | 83.05 |
| TRINITY_DN10139_c0_g1_i1 | ---NA--- | 588 |  |  |
| TRINITY_DN10141_c0_g1_i1 | ATP binding | 479 | 2.32E-39 | 75.15 |
| TRINITY_DN10143_c0_g1_i1 | ---NA--- | 251 |  |  |
| TRINITY_DN10143_c0_g2_i1 | ---NA--- | 254 |  |  |
| TRINITY_DN10143_c0_g3_i1 | ---NA--- | 254 |  |  |
| TRINITY_DN10153_c0_g1_i1 | DEAD-box ATP-dependent RNA helicase 5 | 2245 | 0 | 88.42 |
| TRINITY_DN10153_c0_g1_i2 | DEAD-box ATP-dependent RNA helicase 5 | 2244 | 0 | 86.83 |
| TRINITY_DN10168_c0_g1_i1 | RETICULATA-RELATED chloroplastic | 1536 | 1.02E-175 | 89.55 |
| TRINITY_DN10194_c0_g1_i1 | PXMP2 4 family 4-like | 825 | 1.09E-105 | 85.85 |
| TRINITY_DN10202_c0_g1_i1 | transcription factor MYC2 | 1173 | 2.02E-92 | 70 |
| TRINITY_DN10202_c0_g1_i3 | transcription factor MYC2 | 1268 | 2.48E-95 | 69.8 |
| TRINITY_DN10206_c0_g2_i1 | cytochrome P450 89A2-like | 1908 | 0 | 75.35 |
| TRINITY_DN10208_c0_g1_i1 | ER lumen -retaining receptor | 1513 | 1.67E-121 | 76.65 |
| TRINITY_DN1020_c0_g1_i1 | hypothetical protein BVRB_4g080140 | 735 | 2.61E-11 | 57 |
| TRINITY_DN10216_c0_g1_i1 | ---NA--- | 513 |  |  |
| TRINITY_DN10219_c0_g1_i1 | LOB domain-containing 4-like | 1293 | 1.94E-87 | 85.5 |
| TRINITY_DN10219_c0_g1_i2 | LOB domain-containing 4-like | 1274 | 1.61E-87 | 85.5 |
| TRINITY_DN10228_c0_g1_i1 | ---NA--- | 586 |  |  |
| TRINITY_DN10233_c0_g1_i1 | Structural poly | 784 | 3.61E-36 | 59 |
| TRINITY_DN10235_c0_g1_i1 | galactan beta-1,4-galactosyltransferase GALS1 | 1646 | 0 | 84.15 |
| TRINITY_DN1025_c0_g1_i1 | probable acyl-activating enzyme 6 | 1842 | 0 | 76.05 |
| TRINITY_DN10263_c0_g1_i1 | endoglucanase 12 | 755 | 8.48E-79 | 74.15 |
| TRINITY_DN10268_c0_g1_i1 | DNA polymerase epsilon catalytic subunit | 803 | 4.38E-36 | 63 |
| TRINITY_DN10282_c0_g1_i1 | WD repeat-containing 75 | 2907 | 0 | 75.65 |
| TRINITY_DN10282_c0_g2_i1 | WD repeat-containing 75 | 2100 | 0 | 77.9 |
| TRINITY_DN10302_c0_g1_i1 | probable NAD(P)H dehydrogenase (quinone) FQR1-like 3 | 1015 | 1.93E-115 | 87.9 |
| TRINITY_DN10302_c0_g1_i2 | probable NAD(P)H dehydrogenase (quinone) FQR1-like 3 | 1291 | 3.90E-109 | 88.7 |
| TRINITY_DN10309_c0_g1_i1 | ---NA--- | 330 |  |  |
| TRINITY_DN10309_c0_g2_i1 | ---NA--- | 330 |  |  |
| TRINITY_DN1030_c0_g1_i1 | ---NA--- | 523 |  |  |
| TRINITY_DN10310_c0_g1_i1 | pre-rRNA-processing las1 isoform X5 | 1365 | 3.00E-93 | 59.45 |
| TRINITY_DN10310_c0_g2_i1 | pre-rRNA-processing las1 isoform X4 | 622 | 1.11E-28 | 61.55 |
| TRINITY_DN10349_c0_g1_i1 | homeobox-leucine zipper HAT4 | 1018 | 1.39E-98 | 72.15 |
| TRINITY_DN1034_c0_g1_i1 | ---NA--- | 637 |  |  |
| TRINITY_DN1034_c0_g2_i1 | ---NA--- | 259 |  |  |
| TRINITY_DN10356_c0_g1_i1 | ---NA--- | 352 |  |  |
| TRINITY_DN10356_c0_g2_i1 | ---NA--- | 352 |  |  |
| TRINITY_DN10361_c0_g1_i1 | NADH-ubiquinone oxidoreductase chain 5 | 961 | 7.94E-33 | 88.15 |
| TRINITY_DN10369_c0_g1_i1 | ---NA--- | 235 |  |  |
| TRINITY_DN10369_c0_g2_i1 | ---NA--- | 241 |  |  |
| TRINITY_DN10372_c0_g1_i1 | E3 ubiquitin- ligase Os04g0590900-like | 967 | 2.46E-38 | 81.15 |
| TRINITY_DN10372_c0_g2_i1 | E3 ubiquitin- ligase Os04g0590900-like | 1014 | 4.67E-38 | 81.25 |
| TRINITY_DN10383_c0_g1_i1 | hydrophobic RCI2B | 601 | 1.10E-24 | 95 |
| TRINITY_DN10391_c0_g1_i1 | auxin-responsive IAA13 | 635 | 9.16E-40 | 85.35 |
| TRINITY_DN10391_c0_g2_i1 | auxin-responsive IAA13 | 961 | 3.99E-95 | 70.4 |
| TRINITY_DN10393_c0_g1_i1 | glycine-rich RNA-binding mitochondrial | 810 | 2.27E-40 | 84.7 |
| TRINITY_DN10393_c0_g2_i1 | glycine-rich RNA-binding mitochondrial | 828 | 5.55E-44 | 84.7 |
| TRINITY_DN10401_c0_g1_i1 | phosphoserine aminotransferase chloroplastic-like | 1455 | 0 | 82.65 |
| TRINITY_DN10414_c0_g1_i1 | DTW domain-containing isoform 1 | 1152 | 0 | 80.2 |
| TRINITY_DN10414_c0_g1_i2 | DTW domain-containing isoform 1 | 1275 | 0 | 80.2 |
| TRINITY_DN10417_c0_g1_i1 | CASP 5B2 | 1001 | 4.24E-78 | 87.5 |
| TRINITY_DN10419_c0_g1_i1 | peptidyl-tRNA hydrolase mitochondrial isoform X1 | 881 | 2.06E-46 | 90.75 |
| TRINITY_DN10419_c0_g1_i2 | peptidyl-tRNA hydrolase mitochondrial | 1101 | 4.30E-68 | 84.65 |
| TRINITY_DN10419_c0_g1_i3 | peptidyl-tRNA hydrolase mitochondrial isoform X1 | 1061 | 7.36E-56 | 83.65 |
| TRINITY_DN10419_c0_g1_i4 | peptidyl-tRNA hydrolase mitochondrial isoform X1 | 921 | 7.01E-63 | 90.5 |
| TRINITY_DN10425_c0_g1_i1 | peptidyl-prolyl cis-trans isomerase chloroplastic | 1562 | 0 | 85.5 |
| TRINITY_DN10425_c0_g2_i1 | peptidyl-prolyl cis-trans isomerase chloroplastic | 1529 | 0 | 87.6 |
| TRINITY_DN10425_c0_g3_i1 | Peptidyl-prolyl cis-trans isomerase chloroplastic | 912 | 1.67E-155 | 89.95 |
| TRINITY_DN10440_c1_g1_i1 | pentatricopeptide repeat-containing mitochondrial | 240 | 3.71E-12 | 71.2 |
| TRINITY_DN10442_c0_g1_i1 | ---NA--- | 590 |  |  |
| TRINITY_DN10444_c0_g2_i1 | LITTLE ZIPPER 1-like | 751 | 2.34E-19 | 71.33 |
| TRINITY_DN10444_c0_g2_i2 | ---NA--- | 507 |  |  |
| TRINITY_DN10445_c0_g1_i1 | 60S ribosomal L31 | 883 | 3.77E-60 | 98.1 |
| TRINITY_DN10446_c0_g1_i1 | PREDICTED: uncharacterized protein LOC104889894 | 926 | 1.42E-89 | 67.8 |
| TRINITY_DN10446_c0_g1_i2 | PREDICTED: uncharacterized protein LOC104889894 | 897 | 1.03E-89 | 67.8 |
| TRINITY_DN10448_c0_g1_i1 | transcription initiation factor TFIID subunit 8-like | 1450 | 2.56E-90 | 54.7 |
| TRINITY_DN1044_c0_g2_i1 | endoribonuclease Dicer homolog 3 isoform X1 | 3359 | 0 | 70.1 |
| TRINITY_DN10453_c0_g1_i1 | DNA polymerase epsilon subunit C | 1361 | 1.76E-39 | 72.55 |
| TRINITY_DN10464_c0_g1_i1 | cytoplasmic tRNA 2-thiolation 2 | 1855 | 0 | 75.45 |
| TRINITY_DN10467_c0_g1_i1 | ubiquitin carboxyl-terminal hydrolase isozyme L5 | 1306 | 0 | 92.35 |
| TRINITY_DN10467_c0_g2_i1 | ubiquitin carboxyl-terminal hydrolase isozyme L5 | 1309 | 0 | 92.35 |
| TRINITY_DN10475_c0_g1_i1 | ---NA--- | 1028 |  |  |
| TRINITY_DN10482_c0_g1_i1 | ---NA--- | 662 |  |  |
| TRINITY_DN10483_c0_g1_i1 | thioredoxin chloroplastic | 836 | 1.21E-83 | 72.15 |
| TRINITY_DN10494_c0_g2_i1 | phospholipase D alpha 1 | 449 | 1.28E-06 | 75 |
| TRINITY_DN10494_c0_g3_i1 | phospholipase D alpha 1 | 673 | 6.17E-77 | 79.65 |
| TRINITY_DN10500_c0_g1_i1 | ---NA--- | 983 |  |  |
| TRINITY_DN10503_c0_g1_i1 | ---NA--- | 236 |  |  |
| TRINITY_DN10532_c0_g1_i1 | F-box At1g67340-like | 483 | 1.97E-20 | 57.6 |
| TRINITY_DN10543_c0_g1_i1 | senescence-associated SAG102 | 1671 | 1.79E-107 | 59 |
| TRINITY_DN10543_c0_g2_i1 | ---NA--- | 1290 |  |  |
| TRINITY_DN1054_c0_g1_i1 | Late embryogenesis abundant | 553 | 2.29E-29 | 67.45 |
| TRINITY_DN1054_c0_g2_i1 | Late embryogenesis abundant | 557 | 6.11E-30 | 66.35 |
| TRINITY_DN10562_c0_g1_i1 | 50S ribosomal L25 | 1268 | 1.21E-112 | 84.3 |
| TRINITY_DN10562_c0_g1_i2 | 50S ribosomal L25 | 1386 | 4.47E-112 | 84.3 |
| TRINITY_DN10571_c0_g1_i1 | DUF2301 domain-containing | 1479 | 3.10E-108 | 79.75 |
| TRINITY_DN10579_c0_g1_i1 | zinc finger GIS2 | 1211 | 7.29E-101 | 85.1 |
| TRINITY_DN10579_c0_g2_i1 | zinc finger GIS2 | 1426 | 6.75E-113 | 85.9 |
| TRINITY_DN10582_c0_g1_i1 | RNA-binding 39 | 450 | 7.34E-07 | 81.27 |
| TRINITY_DN10582_c0_g1_i2 | RNA-binding 39 | 524 | 1.11E-06 | 81.3 |
| TRINITY_DN10582_c1_g1_i1 | B2 | 479 | 1.89E-10 | 85 |
| TRINITY_DN10584_c0_g1_i1 | indeterminate-domain chloroplastic-like | 707 | 1.12E-29 | 94 |
| TRINITY_DN10584_c0_g1_i2 | ---NA--- | 640 |  |  |
| TRINITY_DN10585_c0_g1_i1 | ribosomal RNA small subunit methyltransferase nep-1 | 1188 | 3.25E-160 | 79.9 |
| TRINITY_DN10597_c0_g1_i1 | probable calcium-binding CML44 | 655 | 3.41E-60 | 65.15 |
| TRINITY_DN10602_c0_g1_i1 | RNA polymerase sigma factor chloroplastic isoform X1 | 1874 | 0 | 69.4 |
| TRINITY_DN10604_c0_g1_i1 | GID1b | 1760 | 0 | 89.45 |
| TRINITY_DN10624_c1_g1_i1 | heat stress transcription factor B-2a | 527 | 2.49E-06 | 61 |
| TRINITY_DN10635_c0_g1_i1 | ---NA--- | 406 |  |  |
| TRINITY_DN10636_c0_g1_i1 | AT-hook motif nuclear-localized 9 | 1996 | 4.26E-107 | 65.2 |
| TRINITY_DN10636_c0_g2_i1 | AT-hook motif nuclear-localized 9 | 1993 | 2.13E-112 | 65.7 |
| TRINITY_DN10638_c0_g1_i1 | 33 kDa chloroplastic | 985 | 7.99E-96 | 83.4 |
| TRINITY_DN10647_c0_g1_i1 | J JJJ2-like | 992 | 1.66E-75 | 75.75 |
| TRINITY_DN10647_c0_g2_i1 | J JJJ2-like | 854 | 4.16E-76 | 75.75 |
| TRINITY_DN10648_c0_g1_i1 | PREDICTED: uncharacterized protein LOC104901667 | 548 | 7.58E-28 | 74.3 |
| TRINITY_DN10664_c0_g1_i1 | glutathione S-transferase T1 | 977 | 1.84E-136 | 86.25 |
| TRINITY_DN10678_c0_g1_i1 | hypothetical protein SOVF_167710 | 781 | 1.48E-47 | 58.55 |
| TRINITY_DN10678_c0_g1_i2 | hypothetical protein SOVF_167710 | 222 | 2.40E-10 | 84.15 |
| TRINITY_DN10699_c0_g1_i1 | hypothetical protein SOVF_081080 | 933 | 3.53E-26 | 52 |
| TRINITY_DN10699_c0_g2_i1 | hypothetical protein SOVF_081080 | 699 | 4.54E-27 | 52 |
| TRINITY_DN1069_c0_g1_i1 | ---NA--- | 988 |  |  |
| TRINITY_DN10712_c0_g1_i1 | ---NA--- | 211 |  |  |
| TRINITY_DN10715_c0_g1_i1 | DEAD-box ATP-dependent RNA helicase chloroplastic | 2636 | 0 | 86.85 |
| TRINITY_DN10719_c0_g1_i1 | 2-oxoisovalerate dehydrogenase subunit alpha mitochondrial | 1938 | 0 | 83.15 |
| TRINITY_DN1075_c0_g2_i1 | PREDICTED: uncharacterized protein LOC104888521 | 1992 | 1.85E-140 | 55.4 |
| TRINITY_DN10770_c0_g2_i1 | high-affinity nickel-transport family | 1536 | 5.68E-135 | 82.9 |
| TRINITY_DN10777_c0_g1_i1 | ---NA--- | 229 |  |  |
| TRINITY_DN10778_c0_g1_i1 | apoptotic chromatin condensation inducer in the nucleus | 2918 | 0 | 66.85 |
| TRINITY_DN10779_c0_g2_i1 | biotin-- ligase 2-like | 758 | 6.09E-84 | 81.25 |
| TRINITY_DN10779_c0_g3_i1 | biotin-- ligase 2-like | 948 | 5.00E-133 | 84.15 |
| TRINITY_DN1078_c0_g1_i1 | probable pectate lyase 18 | 1714 | 0 | 91.8 |
| TRINITY_DN10791_c0_g1_i2 | ---NA--- | 961 |  |  |
| TRINITY_DN10791_c0_g1_i3 | ---NA--- | 990 |  |  |
| TRINITY_DN10804_c0_g1_i1 | transcription factor MYB44-like | 393 | 6.18E-07 | 53 |
| TRINITY_DN10811_c0_g1_i1 | ---NA--- | 390 |  |  |
| TRINITY_DN10811_c0_g1_i2 | ---NA--- | 361 |  |  |
| TRINITY_DN10811_c0_g1_i3 | ---NA--- | 655 |  |  |
| TRINITY_DN10811_c0_g1_i4 | ---NA--- | 324 |  |  |
| TRINITY_DN10814_c0_g1_i1 | inactive receptor-like serine threonine- kinase At2g40270 | 2501 | 0 | 83.25 |
| TRINITY_DN10819_c0_g1_i1 | Zinc RING-CH-type | 1442 | 4.35E-169 | 82.65 |
| TRINITY_DN10819_c0_g2_i1 | Zinc RING-CH-type | 1361 | 1.63E-169 | 82.65 |
| TRINITY_DN10820_c0_g1_i1 | ---NA--- | 223 |  |  |
| TRINITY_DN10820_c1_g1_i1 | ras-related RHN1 | 1152 | 5.50E-121 | 95.15 |
| TRINITY_DN10820_c1_g1_i2 | ras-related RHN1 | 1139 | 4.60E-121 | 95.15 |
| TRINITY_DN10830_c0_g1_i1 | probable indole-3-acetic acid-amido synthetase | 2011 | 0 | 90.1 |
| TRINITY_DN10830_c0_g3_i1 | probable indole-3-acetic acid-amido synthetase | 757 | 1.96E-118 | 90.7 |
| TRINITY_DN10832_c0_g1_i1 | pentatricopeptide repeat-containing mitochondrial | 1723 | 0 | 81.1 |
| TRINITY_DN10833_c0_g1_i1 | ---NA--- | 466 |  |  |
| TRINITY_DN10839_c0_g1_i1 | hypothetical protein BVRB_9g226030 | 549 | 5.14E-14 | 47.14 |
| TRINITY_DN10839_c0_g1_i2 | hypothetical protein BVRB_9g226030 | 549 | 2.06E-12 | 44.6 |
| TRINITY_DN10840_c0_g1_i1 | ---NA--- | 523 |  |  |
| TRINITY_DN10840_c0_g2_i1 | hypothetical protein BVRB_2g041740 | 1235 | 1.70E-13 | 55.45 |
| TRINITY_DN10843_c0_g1_i1 | polycomb group EMBRYONIC FLOWER 2 isoform X1 | 2329 | 0 | 75.9 |
| TRINITY_DN10844_c0_g1_i1 | Peptidase S41 family | 1025 | 6.50E-135 | 90.2 |
| TRINITY_DN10844_c0_g1_i2 | carboxyl-terminal-processing peptidase chloroplastic isoform X1 | 1919 | 0 | 88.2 |
| TRINITY_DN10844_c0_g2_i1 | carboxyl-terminal-processing peptidase chloroplastic | 775 | 1.81E-11 | 75.3 |
| TRINITY_DN10854_c0_g1_i1 | ---NA--- | 446 |  |  |
| TRINITY_DN10854_c0_g1_i2 | ---NA--- | 523 |  |  |
| TRINITY_DN10855_c0_g1_i1 | probable LRR receptor-like serine threonine- kinase At3g47570 | 3642 | 0 | 64.05 |
| TRINITY_DN10859_c0_g1_i1 | ---NA--- | 212 |  |  |
| TRINITY_DN10859_c0_g2_i1 | ---NA--- | 252 |  |  |
| TRINITY_DN10866_c0_g1_i2 | NEDD8-activating enzyme E1 catalytic subunit | 2212 | 0 | 89.2 |
| TRINITY_DN10868_c0_g1_i1 | bifunctional chloroplastic | 1588 | 3.11E-154 | 90.15 |
| TRINITY_DN10868_c0_g2_i1 | bifunctional chloroplastic | 1587 | 0 | 90.75 |
| TRINITY_DN10881_c0_g1_i1 | neutral alkaline invertase chloroplastic | 2550 | 0 | 82.1 |
| TRINITY_DN10883_c0_g1_i1 | ---NA--- | 273 |  |  |
| TRINITY_DN10884_c0_g1_i1 | membrane steroid-binding 2-like | 1312 | 6.60E-87 | 75.9 |
| TRINITY_DN10904_c0_g1_i1 | Sterol regulatory element-binding cleavage-activating | 925 | 7.77E-42 | 80.8 |
| TRINITY_DN10904_c0_g2_i1 | Sterol regulatory element-binding cleavage-activating | 924 | 5.55E-52 | 65.5 |
| TRINITY_DN10905_c0_g1_i1 | Cytochrome P450 | 893 | 7.62E-80 | 68.25 |
| TRINITY_DN10916_c0_g1_i1 | chloride channel 1 | 956 | 1.05E-25 | 83.55 |
| TRINITY_DN10929_c0_g1_i1 | translation initiation factor IF-2-like isoform X1 | 1497 | 3.00E-29 | 50.95 |
| TRINITY_DN10932_c0_g1_i1 | topless-related 4 isoform X1 | 3672 | 0 | 85.05 |
| TRINITY_DN10932_c0_g1_i2 | topless-related 4 isoform X1 | 3663 | 0 | 84.8 |
| TRINITY_DN10939_c0_g1_i3 | UMP-CMP kinase isoform X1 | 1224 | 4.08E-128 | 79.45 |
| TRINITY_DN10942_c0_g1_i2 | transmembrane 33 homolog | 1470 | 8.54E-168 | 83.7 |
| TRINITY_DN10958_c0_g1_i1 | TVP38 TMEM64 family membrane slr0305-like | 1583 | 3.20E-107 | 92.45 |
| TRINITY_DN10967_c0_g1_i1 | beta-adaptin B | 2991 | 0 | 92.9 |
| TRINITY_DN10972_c0_g1_i1 | trihelix transcription factor GT-2-like | 1394 | 8.59E-61 | 76.75 |
| TRINITY_DN10972_c0_g1_i2 | trihelix transcription factor GT-2-like | 1382 | 7.73E-61 | 76.75 |
| TRINITY_DN10973_c0_g1_i1 | hypothetical protein SOVF_058070 | 485 | 4.64E-16 | 73 |
| TRINITY_DN10973_c0_g2_i1 | hypothetical protein SOVF_058070 | 803 | 5.12E-27 | 72.2 |
| TRINITY_DN10979_c0_g1_i1 | DEAD-box ATP-dependent RNA helicase 16 | 1976 | 0 | 83.85 |
| TRINITY_DN10980_c0_g1_i1 | photosystem I subunit IX (chloroplast) | 539 | 8.15E-20 | 98.85 |
| TRINITY_DN10981_c0_g1_i1 | methyl- -binding domain-containing 4-like | 1185 | 1.60E-100 | 78 |
| TRINITY_DN10991_c1_g1_i1 | 26S protease regulatory subunit 6B homolog | 1447 | 0 | 97.9 |
| TRINITY_DN10999_c0_g1_i1 | serine threonine- kinase D6PKL2-like | 2349 | 0 | 83.8 |
| TRINITY_DN11015_c0_g1_i1 | probable NOT transcription complex subunit VIP2 isoform X2 | 2263 | 0 | 80.75 |
| TRINITY_DN11015_c0_g1_i2 | probable NOT transcription complex subunit VIP2 isoform X2 | 2113 | 0 | 80.55 |
| TRINITY_DN11016_c0_g1_i1 | SUMO-conjugating enzyme UBC9 | 303 | 1.88E-19 | 99.6 |
| TRINITY_DN11020_c0_g1_i1 | hypothetical protein MANES_01G232200 | 402 | 5.70E-07 | 56 |
| TRINITY_DN11025_c0_g1_i1 | ---NA--- | 760 |  |  |
| TRINITY_DN11040_c0_g1_i1 | ---NA--- | 623 |  |  |
| TRINITY_DN11042_c0_g1_i1 | transcription factor PCL1 | 1103 | 5.41E-110 | 68.35 |
| TRINITY_DN11048_c0_g1_i1 | diaminopimelate chloroplastic | 1409 | 0 | 86.65 |
| TRINITY_DN11060_c0_g1_i1 | 40S ribosomal S17-4-like | 901 | 6.36E-76 | 94.4 |
| TRINITY_DN11062_c0_g1_i1 | ---NA--- | 850 |  |  |
| TRINITY_DN11079_c0_g1_i1 | ---NA--- | 346 |  |  |
| TRINITY_DN11079_c0_g2_i1 | ---NA--- | 451 |  |  |
| TRINITY_DN11079_c1_g1_i1 | acidic leucine-rich nuclear phospho 32 family member B | 1034 | 9.50E-09 | 71 |
| TRINITY_DN11084_c0_g1_i1 | histidine-containing phosphotransfer 1-like | 1135 | 6.52E-73 | 77.7 |
| TRINITY_DN11086_c0_g1_i1 | ---NA--- | 276 |  |  |
| TRINITY_DN11097_c0_g1_i1 | Rad60-SLD domain-containing | 1065 | 8.92E-58 | 64.6 |
| TRINITY_DN11097_c0_g2_i1 | Small ubiquitin-related SUMO | 989 | 1.12E-48 | 68.95 |
| TRINITY_DN11099_c0_g1_i1 | hypothetical protein BVRB_5g125770 | 942 | 1.43E-23 | 60 |
| TRINITY_DN11113_c0_g1_i1 | cyclic dof factor 3 | 1536 | 5.46E-109 | 56.1 |
| TRINITY_DN11115_c0_g1_i1 | probable phosphatase 2C 53 | 2397 | 0 | 73.4 |
| TRINITY_DN11115_c0_g2_i1 | probable phosphatase 2C 53 | 2399 | 0 | 73.4 |
| TRINITY_DN11127_c0_g1_i1 | ---NA--- | 380 |  |  |
| TRINITY_DN11128_c0_g1_i1 | mediator of RNA polymerase II transcription subunit 29 | 842 | 2.60E-29 | 65.3 |
| TRINITY_DN11135_c0_g2_i1 | phytosulfokine receptor 2-like | 2582 | 0 | 81.05 |
| TRINITY_DN11139_c0_g1_i1 | photosystem II core complex s chloroplastic | 690 | 4.72E-58 | 75.85 |
| TRINITY_DN11139_c0_g1_i2 | photosystem II core complex s chloroplastic | 471 | 7.05E-55 | 77.75 |
| TRINITY_DN11147_c0_g1_i2 | auxin efflux carrier component 5 | 1437 | 0 | 78.1 |
| TRINITY_DN11168_c0_g1_i1 | transmembrane C9orf5 | 1843 | 0 | 83.95 |
| TRINITY_DN11169_c0_g1_i1 | J JJJ2-like | 1232 | 2.71E-90 | 60.1 |
| TRINITY_DN11174_c0_g1_i1 | ---NA--- | 290 |  |  |
| TRINITY_DN11178_c0_g1_i1 | kanadaptin | 2893 | 0 | 74.1 |
| TRINITY_DN1119_c0_g1_i1 | PREDICTED: uncharacterized protein LOC104902589 isoform X6 | 572 | 5.50E-08 | 77 |
| TRINITY_DN11206_c0_g1_i1 | ---NA--- | 509 |  |  |
| TRINITY_DN11214_c0_g1_i1 | TBC1 domain family member 2A | 757 | 3.52E-105 | 93.7 |
| TRINITY_DN11223_c0_g1_i1 | GTP-binding nuclear Ran-3 | 754 | 9.72E-63 | 94.9 |
| TRINITY_DN11223_c0_g2_i1 | GTP-binding nuclear Ran-3 | 755 | 2.05E-58 | 94.9 |
| TRINITY_DN11226_c0_g1_i1 | zinc finger family | 1842 | 5.36E-101 | 64.35 |
| TRINITY_DN11227_c0_g1_i1 | NAD(P)H-quinone oxidoreductase subunit chloroplastic | 953 | 2.58E-128 | 79.5 |
| TRINITY_DN11227_c1_g1_i1 | Serine arginine repetitive matrix | 1059 | 9.88E-90 | 49.35 |
| TRINITY_DN11228_c0_g1_i1 | hypothetical protein B456_005G056700 | 713 | 2.56E-40 | 91.95 |
| TRINITY_DN1122_c0_g1_i1 | polygalacturonase At1g48100 | 827 | 6.75E-118 | 88.35 |
| TRINITY_DN11253_c0_g1_i1 | chloroplast photosystem II | 861 | 2.44E-38 | 73.3 |
| TRINITY_DN11255_c0_g1_i1 | PREDICTED: uncharacterized protein LOC104892271 | 1094 | 1.61E-121 | 79.7 |
| TRINITY_DN11272_c0_g1_i1 | TRANSPORT INHIBITOR RESPONSE 1 | 2769 | 0 | 88.55 |
| TRINITY_DN11273_c0_g1_i1 | ---NA--- | 346 |  |  |
| TRINITY_DN11277_c0_g1_i1 | ECERIFERUM 1 | 1372 | 2.69E-129 | 61.35 |
| TRINITY_DN11287_c0_g2_i2 | carbonic chloroplastic-like | 1518 | 1.09E-150 | 80.3 |
| TRINITY_DN11297_c0_g1_i1 | EMBRYO SAC DEVELOPMENT ARREST chloroplastic | 768 | 1.09E-70 | 76.25 |
| TRINITY_DN11308_c0_g1_i1 | 23 kDa jasmonate-induced -like | 1066 | 1.17E-92 | 69 |
| TRINITY_DN11308_c0_g2_i1 | 23 kDa jasmonate-induced -like | 358 | 6.37E-20 | 62.44 |
| TRINITY_DN11323_c0_g1_i3 | proline dehydrogenase mitochondrial-like | 1310 | 0 | 86 |
| TRINITY_DN11323_c0_g1_i4 | proline dehydrogenase mitochondrial-like | 1406 | 0 | 86.55 |
| TRINITY_DN11340_c0_g1_i1 | F-box At1g78280 | 443 | 3.78E-40 | 71.85 |
| TRINITY_DN11340_c0_g2_i1 | F-box At1g78280 | 544 | 2.66E-63 | 74.1 |
| TRINITY_DN11344_c0_g1_i1 | ---NA--- | 253 |  |  |
| TRINITY_DN11352_c0_g1_i1 | ---NA--- | 244 |  |  |
| TRINITY_DN11362_c0_g2_i1 | ---NA--- | 565 |  |  |
| TRINITY_DN11364_c0_g1_i1 | #NAME? | 1166 | 4.26E-143 | 76.15 |
| TRINITY_DN11365_c0_g1_i1 | ATP-dependent DNA helicase Q-like 1 | 2466 | 0 | 79.1 |
| TRINITY_DN11365_c0_g3_i1 | ATP-dependent DNA helicase Q-like 1 | 806 | 1.66E-47 | 63.75 |
| TRINITY_DN11378_c0_g1_i1 | Metallo-hydrolase oxidoreductase superfamily isoform 1 | 1225 | 1.65E-157 | 86 |
| TRINITY_DN11378_c0_g1_i2 | Metallo-hydrolase oxidoreductase superfamily isoform 1 | 1494 | 7.29E-152 | 86.2 |
| TRINITY_DN11379_c0_g1_i1 | endoglucanase 25-like | 2353 | 0 | 90.5 |
| TRINITY_DN11380_c0_g1_i1 | golgin subfamily A member 4-like | 567 | 1.19E-60 | 86.5 |
| TRINITY_DN11382_c0_g1_i1 | probable nuclear hormone receptor HR38 isoform X1 | 581 | 3.08E-40 | 78.9 |
| TRINITY_DN11382_c0_g1_i4 | probable nuclear hormone receptor HR38 isoform X1 | 505 | 1.24E-39 | 77.45 |
| TRINITY_DN11383_c0_g1_i1 | calcineurin subunit B-like isoform X2 | 647 | 7.24E-27 | 94 |
| TRINITY_DN11383_c0_g2_i1 | calcineurin subunit B | 546 | 3.83E-40 | 94.85 |
| TRINITY_DN11388_c0_g1_i1 | exosome complex component RRP41 homolog | 1346 | 4.04E-162 | 96.75 |
| TRINITY_DN11389_c0_g1_i1 | ---NA--- | 540 |  |  |
| TRINITY_DN11400_c0_g1_i1 | ---NA--- | 237 |  |  |
| TRINITY_DN11402_c0_g1_i1 | ---NA--- | 424 |  |  |
| TRINITY_DN11406_c0_g1_i1 | ---NA--- | 407 |  |  |
| TRINITY_DN11411_c0_g1_i1 | ---NA--- | 261 |  |  |
| TRINITY_DN11411_c0_g1_i2 | ---NA--- | 260 |  |  |
| TRINITY_DN11414_c0_g1_i1 | hypothetical protein TSUD_22260 | 481 | 6.93E-23 | 90.35 |
| TRINITY_DN11414_c0_g1_i2 | ---NA--- | 265 |  |  |
| TRINITY_DN11414_c0_g1_i3 | hypothetical protein TSUD_22260 | 486 | 3.66E-23 | 90.35 |
| TRINITY_DN11414_c0_g1_i4 | ---NA--- | 302 |  |  |
| TRINITY_DN11414_c0_g1_i5 | hypothetical protein TSUD_22260 | 523 | 5.32E-23 | 90.35 |
| TRINITY_DN11414_c0_g1_i6 | ---NA--- | 260 |  |  |
| TRINITY_DN11419_c0_g1_i1 | ---NA--- | 377 |  |  |
| TRINITY_DN11421_c0_g1_i1 | auxin-responsive IAA14 | 851 | 2.36E-115 | 84.1 |
| TRINITY_DN11426_c0_g1_i1 | telomere repeat-binding 5 isoform X1 | 2459 | 0 | 62.7 |
| TRINITY_DN1142_c0_g1_i1 | CURVATURE THYLAKOID chloroplastic-like | 1469 | 5.78E-47 | 62.05 |
| TRINITY_DN11442_c0_g1_i1 | (S)-ureidoglycine aminohydrolase | 1194 | 1.47E-171 | 87.1 |
| TRINITY_DN11442_c0_g1_i2 | (S)-ureidoglycine aminohydrolase | 1127 | 2.66E-120 | 92.05 |
| TRINITY_DN11451_c0_g1_i1 | thioredoxin chloroplastic | 1305 | 8.18E-114 | 87.7 |
| TRINITY_DN11451_c0_g2_i1 | thioredoxin chloroplastic | 1418 | 8.55E-85 | 86.45 |
| TRINITY_DN11454_c0_g1_i1 | ---NA--- | 469 |  |  |
| TRINITY_DN11454_c0_g1_i2 | ---NA--- | 558 |  |  |
| TRINITY_DN11455_c0_g1_i1 | ethylene-responsive transcription factor 3-like | 1776 | 1.92E-42 | 56.05 |
| TRINITY_DN11463_c1_g1_i1 | condensin-2 complex subunit H2 | 1435 | 2.42E-151 | 64.55 |
| TRINITY_DN11466_c0_g1_i1 | ---NA--- | 235 |  |  |
| TRINITY_DN11466_c1_g1_i1 | ---NA--- | 305 |  |  |
| TRINITY_DN11469_c0_g1_i1 | DDT domain-containing DDR4 | 265 | 7.42E-06 | 69 |
| TRINITY_DN11472_c0_g1_i1 | nuclear pore complex NUP1 | 2496 | 0 | 53.2 |
| TRINITY_DN11473_c0_g2_i1 | WVD2-like 4 | 1947 | 1.29E-87 | 70.95 |
| TRINITY_DN11473_c0_g2_i2 | WVD2-like 4 | 1929 | 6.34E-90 | 68.15 |
| TRINITY_DN11473_c1_g1_i1 | Signal transducer and transcription activator isoform 1 | 900 | 1.20E-58 | 83.1 |
| TRINITY_DN11484_c0_g1_i1 | histone H1 | 1050 | 1.51E-45 | 77.35 |
| TRINITY_DN11486_c0_g1_i1 | Tumor necrosis factor receptor superfamily member 21 | 881 | 1.01E-51 | 77.25 |
| TRINITY_DN11489_c0_g1_i1 | 60S ribosomal L19-1 | 1137 | 3.00E-115 | 96.65 |
| TRINITY_DN11489_c0_g2_i1 | 60S ribosomal L19-1 | 1137 | 4.79E-112 | 97.15 |
| TRINITY_DN11493_c0_g1_i1 | hypothetical protein SOVF_045590 | 599 | 3.54E-23 | 88.7 |
| TRINITY_DN11493_c0_g1_i2 | hypothetical protein SOVF_045590 | 636 | 4.94E-23 | 88.7 |
| TRINITY_DN11509_c0_g1_i1 | 60S ribosomal L18a | 955 | 2.43E-34 | 67.7 |
| TRINITY_DN11510_c1_g1_i1 | rRNA-processing fcf2-like | 1004 | 1.67E-80 | 81.9 |
| TRINITY_DN11528_c0_g1_i1 | PREDICTED: uncharacterized protein LOC104903552 | 1267 | 1.04E-72 | 63.35 |
| TRINITY_DN11528_c0_g2_i1 | ---NA--- | 400 |  |  |
| TRINITY_DN11537_c0_g1_i1 | autophagy-related 9 | 3307 | 0 | 79.5 |
| TRINITY_DN11537_c0_g3_i1 | Autophagy-related 9 | 1454 | 0 | 88.15 |
| TRINITY_DN11538_c0_g1_i1 | DNA-damage-repair toleration chloroplastic | 743 | 1.48E-27 | 63.55 |
| TRINITY_DN11546_c0_g1_i1 | cysteine ase inhibitor 1-like | 520 | 6.66E-24 | 76.85 |
| TRINITY_DN11549_c0_g2_i1 | ethylene-responsive transcription factor ERF071 | 1149 | 1.19E-80 | 62.35 |
| TRINITY_DN11559_c0_g1_i1 | ---NA--- | 409 |  |  |
| TRINITY_DN1155_c0_g1_i1 | ---NA--- | 253 |  |  |
| TRINITY_DN11563_c0_g1_i1 | calvin cycle CP12- chloroplastic-like | 936 | 6.85E-30 | 70.3 |
| TRINITY_DN11571_c0_g1_i1 | PRA1 family B1-like | 1151 | 6.39E-52 | 87.2 |
| TRINITY_DN11589_c0_g1_i1 | probable histone | 503 | 7.46E-26 | 72.9 |
| TRINITY_DN11593_c0_g1_i1 | elongation of fatty acids 3-like | 1497 | 1.27E-136 | 82.15 |
| TRINITY_DN11593_c0_g1_i2 | elongation of fatty acids 3-like | 1486 | 1.14E-136 | 82.15 |
| TRINITY_DN11596_c0_g1_i1 | nucleolar 56-like | 309 | 1.89E-15 | 89.6 |
| TRINITY_DN11611_c0_g1_i1 | photosystem I reaction center subunit chloroplastic | 937 | 6.49E-114 | 84.7 |
| TRINITY_DN11633_c0_g1_i1 | ATP-dependent zinc metalloprotease FTSH mitochondrial | 2923 | 0 | 89.8 |
| TRINITY_DN11638_c0_g1_i1 | hypothetical protein SOVF_119280 | 402 | 5.10E-37 | 58.85 |
| TRINITY_DN11638_c0_g2_i1 | hypothetical protein BVRB_2g027330 | 674 | 1.75E-58 | 60.9 |
| TRINITY_DN11638_c2_g1_i1 | KH domain-containing At4g18375 | 1036 | 1.80E-42 | 77.05 |
| TRINITY_DN11642_c0_g1_i1 | pentatricopeptide repeat-containing chloroplastic | 2462 | 0 | 77.3 |
| TRINITY_DN11654_c0_g1_i1 | pectinesterase pectinesterase inhibitor | 1157 | 0 | 86.75 |
| TRINITY_DN11671_c0_g1_i1 | dynein light chain cytoplasmic | 654 | 1.75E-46 | 84.7 |
| TRINITY_DN11677_c0_g1_i1 | ---NA--- | 248 |  |  |
| TRINITY_DN11678_c0_g1_i1 | probable S-acyltransferase 19 | 714 | 3.62E-21 | 75.4 |
| TRINITY_DN11682_c0_g1_i1 | LEAF RUST 10 DISEASE-RESISTANCE LOCUS RECEPTOR-LIKE PROTEIN KINASE-like isoform X2 | 919 | 5.73E-144 | 82.4 |
| TRINITY_DN11691_c0_g1_i1 | calmodulin-binding 60 B | 2592 | 0 | 82.25 |
| TRINITY_DN11691_c0_g1_i2 | calmodulin-binding 60 B | 2822 | 0 | 82 |
| TRINITY_DN11691_c0_g1_i3 | calmodulin-binding 60 B | 2598 | 0 | 82 |
| TRINITY_DN11691_c0_g1_i4 | calmodulin-binding 60 B | 2816 | 0 | 82.25 |
| TRINITY_DN11695_c0_g1_i1 | neurogenic mastermind [Tarenaya hassleriana] | 610 | 1.65E-44 | 92 |
| TRINITY_DN11695_c0_g1_i2 | neurogenic mastermind [Tarenaya hassleriana] | 657 | 8.30E-41 | 92 |
| TRINITY_DN11695_c0_g2_i1 | neurogenic mastermind [Tarenaya hassleriana] | 341 | 1.28E-34 | 90.7 |
| TRINITY_DN11696_c0_g1_i1 | ---NA--- | 261 |  |  |
| TRINITY_DN11699_c0_g1_i1 | ---NA--- | 629 |  |  |
| TRINITY_DN11704_c1_g1_i1 | ---NA--- | 210 |  |  |
| TRINITY_DN11705_c0_g1_i1 | transcription factor MYB1R1 | 1209 | 6.27E-67 | 76.2 |
| TRINITY_DN11707_c0_g1_i1 | late embryogenesis abundant D-29-like | 1170 | 1.65E-14 | 52.45 |
| TRINITY_DN1171_c0_g1_i1 | isoform 1 | 1693 | 0 | 84 |
| TRINITY_DN1171_c0_g2_i1 | isoform 1 | 1696 | 0 | 84.1 |
| TRINITY_DN11725_c0_g1_i1 | ---NA--- | 236 |  |  |
| TRINITY_DN11725_c0_g1_i2 | ---NA--- | 221 |  |  |
| TRINITY_DN11727_c0_g1_i1 | myb family transcription factor EFM | 1615 | 1.40E-162 | 69.5 |
| TRINITY_DN11729_c0_g1_i1 | WUSCHEL-related homeobox 5-like | 613 | 9.38E-78 | 72.35 |
| TRINITY_DN11736_c0_g1_i1 | disulfide isomerase-like 5-2 | 1595 | 6.94E-163 | 85 |
| TRINITY_DN11739_c0_g1_i1 | annexin D2-like | 1215 | 0 | 88.85 |
| TRINITY_DN11739_c0_g2_i1 | annexin RJ4 | 1215 | 0 | 87.65 |
| TRINITY_DN11741_c0_g1_i1 | aspartate aminotransferase | 1890 | 0 | 89.95 |
| TRINITY_DN11747_c0_g1_i2 | ---NA--- | 509 |  |  |
| TRINITY_DN11748_c0_g1_i1 | palmitoyl-acyl carrier chloroplastic | 2025 | 0 | 87.8 |
| TRINITY_DN11749_c0_g1_i1 | serine threonine- phosphatase PP1 isozyme 2-like | 419 | 5.36E-12 | 96.9 |
| TRINITY_DN11749_c0_g2_i1 | serine threonine- phosphatase PP1 isozyme 2-like | 419 | 5.36E-12 | 96.9 |
| TRINITY_DN11752_c0_g1_i1 | cinnamoyl- reductase 1 | 1250 | 7.61E-174 | 84.8 |
| TRINITY_DN11765_c0_g1_i1 | AF334834_1 chaperon | 1024 | 1.76E-50 | 77.55 |
| TRINITY_DN11781_c0_g1_i1 | Histone deacetylase 18 | 771 | 1.39E-38 | 55.15 |
| TRINITY_DN11782_c0_g2_i1 | SMG7 | 3029 | 0 | 68.2 |
| TRINITY_DN11793_c0_g1_i1 | pectinesterase 2 | 1571 | 0 | 74.55 |
| TRINITY_DN11794_c0_g1_i1 | ---NA--- | 820 |  |  |
| TRINITY_DN11794_c0_g1_i2 | ---NA--- | 717 |  |  |
| TRINITY_DN11814_c0_g1_i1 | probable steroid-binding 3 | 664 | 2.26E-53 | 90.4 |
| TRINITY_DN11823_c0_g1_i1 | ATP-dependent Clp protease proteolytic subunit chloroplastic | 1483 | 1.05E-140 | 84.7 |
| TRINITY_DN11824_c0_g1_i1 | ---NA--- | 724 |  |  |
| TRINITY_DN11825_c0_g1_i1 | ---NA--- | 301 |  |  |
| TRINITY_DN11825_c0_g2_i1 | ---NA--- | 314 |  |  |
| TRINITY_DN11831_c0_g1_i1 | probable transcription factor At3g04930 | 873 | 6.77E-21 | 63.1 |
| TRINITY_DN11834_c0_g1_i1 | ---NA--- | 408 |  |  |
| TRINITY_DN11834_c1_g1_i1 | glutaredoxin- chloroplastic | 571 | 4.83E-62 | 94.65 |
| TRINITY_DN11843_c0_g1_i1 | CDP-diacylglycerol--glycerol-3-phosphate 3-phosphatidyltransferase 2 | 1304 | 5.87E-104 | 87.4 |
| TRINITY_DN11843_c0_g2_i1 | CDP-diacylglycerol--glycerol-3-phosphate 3-phosphatidyltransferase 2 | 1219 | 4.90E-103 | 87.55 |
| TRINITY_DN11843_c2_g1_i1 | UPSTREAM OF FLC | 543 | 9.33E-28 | 72.95 |
| TRINITY_DN11855_c0_g1_i1 | ---NA--- | 674 |  |  |
| TRINITY_DN11855_c0_g1_i2 | PREDICTED: protein E6-like | 509 | 8.28E-06 | 93 |
| TRINITY_DN11855_c1_g1_i1 | ---NA--- | 335 |  |  |
| TRINITY_DN11882_c0_g1_i1 | ---NA--- | 481 |  |  |
| TRINITY_DN11882_c0_g2_i1 | ---NA--- | 341 |  |  |
| TRINITY_DN11889_c0_g1_i1 | ---NA--- | 539 |  |  |
| TRINITY_DN11889_c0_g1_i2 | Homeobox-leucine zipper HAT22 | 969 | 1.36E-48 | 67.5 |
| TRINITY_DN11892_c0_g1_i1 | transcription factor bHLH123-like | 379 | 1.31E-10 | 57.4 |
| TRINITY_DN11892_c0_g2_i1 | transcription factor bHLH123-like isoform X1 | 900 | 4.47E-36 | 59.9 |
| TRINITY_DN11907_c0_g1_i1 | 40S ribosomal S19-3 | 810 | 6.98E-75 | 94.4 |
| TRINITY_DN11910_c0_g1_i1 | ---NA--- | 675 |  |  |
| TRINITY_DN11918_c0_g1_i1 | phospholipase D delta | 3106 | 0 | 83.1 |
| TRINITY_DN11921_c0_g1_i1 | ---NA--- | 889 |  |  |
| TRINITY_DN11921_c0_g2_i1 | ---NA--- | 889 |  |  |
| TRINITY_DN11924_c0_g1_i1 | ---NA--- | 323 |  |  |
| TRINITY_DN1192_c0_g1_i1 | ---NA--- | 414 |  |  |
| TRINITY_DN1192_c1_g1_i1 | hypothetical protein BVRB_2g028690 | 536 | 4.48E-09 | 67 |
| TRINITY_DN11931_c0_g1_i1 | zinc finger A20 and AN1 domain-containing stress-associated 5 | 377 | 4.86E-27 | 97.5 |
| TRINITY_DN11931_c1_g1_i1 | ---NA--- | 232 |  |  |
| TRINITY_DN11937_c0_g1_i1 | ---NA--- | 765 |  |  |
| TRINITY_DN11938_c0_g2_i1 | abscisic acid 8 -hydroxylase 1 | 462 | 1.80E-64 | 92.65 |
| TRINITY_DN11940_c0_g1_i1 | probable sodium metabolite cotransporter chloroplastic | 1572 | 8.47E-163 | 82.35 |
| TRINITY_DN11951_c0_g1_i1 | PREDICTED: uncharacterized protein LOC104887230 isoform X2 | 1935 | 0 | 68.15 |
| TRINITY_DN11952_c0_g1_i1 | ---NA--- | 221 |  |  |
| TRINITY_DN11954_c0_g1_i1 | probable WRKY transcription factor 15 | 928 | 4.39E-79 | 85.55 |
| TRINITY_DN11958_c0_g1_i1 | mitotic-spindle organizing 1A-like | 397 | 6.07E-28 | 92.15 |
| TRINITY_DN11962_c0_g1_i1 | acetyltransferase At1g77540 | 948 | 3.75E-40 | 81.8 |
| TRINITY_DN11971_c0_g2_i1 | PREDICTED: uncharacterized protein LOC104884345 | 948 | 9.42E-31 | 84.3 |
| TRINITY_DN11971_c0_g3_i1 | PREDICTED: uncharacterized protein LOC104884345 | 964 | 5.07E-47 | 65.6 |
| TRINITY_DN11973_c0_g1_i1 | disease resistance RGA3 | 280 | 4.64E-12 | 60.65 |
| TRINITY_DN11983_c0_g1_i1 | pseudouridine-5 -phosphatase | 1569 | 0 | 86.85 |
| TRINITY_DN11987_c0_g2_i1 | UDP-glycosyltransferase 76C2-like | 1478 | 1.71E-122 | 61.2 |
| TRINITY_DN11988_c0_g1_i1 | NRT1 PTR FAMILY -like | 1970 | 0 | 83.2 |
| TRINITY_DN1198_c0_g1_i1 | inositol 3-kinase | 426 | 8.08E-48 | 70.15 |
| TRINITY_DN1198_c0_g2_i1 | inositol 3-kinase | 426 | 5.25E-47 | 70.05 |
| TRINITY_DN12003_c0_g1_i1 | ---NA--- | 204 |  |  |
| TRINITY_DN12005_c0_g1_i1 | mediator of RNA polymerase II transcription subunit 12-like | 1850 | 2.23E-77 | 51 |
| TRINITY_DN12005_c0_g1_i2 | mediator of RNA polymerase II transcription subunit 12-like | 1975 | 1.49E-76 | 50.7 |
| TRINITY_DN12012_c0_g1_i1 | glycosyltransferase family 64 C4 | 1544 | 3.00E-179 | 82.2 |
| TRINITY_DN12025_c0_g1_i1 | WAS WASL-interacting family member 1 isoform X1 | 1350 | 3.11E-25 | 79.25 |
| TRINITY_DN12025_c0_g2_i1 | WAS WASL-interacting family member 1 isoform X2 | 1335 | 3.78E-20 | 79.4 |
| TRINITY_DN12025_c0_g3_i1 | WAS WASL-interacting family member 1 isoform X2 | 1362 | 3.88E-20 | 79.9 |
| TRINITY_DN12026_c0_g2_i1 | ---NA--- | 302 |  |  |
| TRINITY_DN12027_c0_g1_i1 | DNAJ JJJ1 homolog | 547 | 1.59E-71 | 85.75 |
| TRINITY_DN12027_c0_g2_i1 | DNAJ JJJ1 homolog | 548 | 2.33E-52 | 85.55 |
| TRINITY_DN12027_c1_g1_i1 | ---NA--- | 217 |  |  |
| TRINITY_DN12028_c0_g1_i1 | basic 7S globulin-like | 1412 | 8.63E-86 | 57.35 |
| TRINITY_DN12028_c0_g2_i1 | basic 7S globulin-like | 1415 | 8.89E-86 | 57.35 |
| TRINITY_DN12029_c0_g1_i1 | proline-rich receptor kinase PERK11 isoform X2 | 1009 | 1.45E-28 | 69.35 |
| TRINITY_DN12029_c0_g2_i1 | ---NA--- | 490 |  |  |
| TRINITY_DN12033_c0_g1_i1 | ---NA--- | 366 |  |  |
| TRINITY_DN12045_c0_g1_i1 | DETOXIFICATION 35 | 847 | 6.69E-12 | 69.7 |
| TRINITY_DN12047_c0_g1_i1 | ---NA--- | 648 |  |  |
| TRINITY_DN12047_c0_g2_i1 | ---NA--- | 649 |  |  |
| TRINITY_DN12048_c0_g1_i1 | polyadenylate-binding -interacting 11-like | 686 | 5.22E-123 | 95.25 |
| TRINITY_DN12048_c0_g2_i1 | polyadenylate-binding -interacting 11-like | 484 | 1.82E-76 | 95.55 |
| TRINITY_DN12052_c0_g1_i1 | probable GTP diphosphokinase chloroplastic | 2629 | 0 | 81.05 |
| TRINITY_DN12052_c0_g2_i1 | probable GTP diphosphokinase chloroplastic | 2629 | 0 | 81.9 |
| TRINITY_DN12053_c0_g1_i1 | ---NA--- | 315 |  |  |
| TRINITY_DN12068_c0_g1_i1 | lycopene epsilon chloroplastic | 2211 | 0 | 86.35 |
| TRINITY_DN12076_c0_g1_i1 | ---NA--- | 544 |  |  |
| TRINITY_DN12076_c0_g2_i1 | ---NA--- | 625 |  |  |
| TRINITY_DN12076_c1_g1_i1 | ---NA--- | 286 |  |  |
| TRINITY_DN12076_c1_g2_i1 | ---NA--- | 369 |  |  |
| TRINITY_DN12076_c1_g3_i1 | ---NA--- | 220 |  |  |
| TRINITY_DN12104_c0_g1_i1 | ---NA--- | 487 |  |  |
| TRINITY_DN12104_c0_g1_i2 | ---NA--- | 308 |  |  |
| TRINITY_DN12115_c0_g1_i1 | RETICULATA-RELATED chloroplastic-like | 1301 | 3.32E-103 | 83.6 |
| TRINITY_DN12134_c0_g1_i1 | rhomboid 19 | 1373 | 8.89E-135 | 82.65 |
| TRINITY_DN12144_c0_g1_i1 | type IV inositol polyphosphate 5-phosphatase 3 isoform X1 | 314 | 9.01E-15 | 77.15 |
| TRINITY_DN12144_c0_g1_i2 | type IV inositol polyphosphate 5-phosphatase 3-like isoform X2 | 447 | 1.21E-06 | 83 |
| TRINITY_DN12153_c0_g1_i1 | GATA transcription factor 5-like | 1439 | 5.88E-109 | 56.6 |
| TRINITY_DN12179_c0_g1_i1 | delta(7)-sterol-C5(6)-desaturase-like isoform X2 | 316 | 3.62E-21 | 74.75 |
| TRINITY_DN12179_c0_g2_i1 | delta(7)-sterol-C5(6)-desaturase-like | 338 | 2.40E-29 | 75.95 |
| TRINITY_DN12179_c1_g1_i1 | ---NA--- | 246 |  |  |
| TRINITY_DN12183_c0_g2_i1 | DUF4050 domain-containing | 982 | 1.07E-59 | 64.95 |
| TRINITY_DN12183_c0_g3_i1 | DUF4050 domain-containing | 986 | 3.96E-74 | 68.6 |
| TRINITY_DN12186_c0_g1_i1 | ---NA--- | 275 |  |  |
| TRINITY_DN12187_c0_g1_i1 | ---NA--- | 554 |  |  |
| TRINITY_DN12196_c0_g1_i1 | 60S ribosomal L9 | 661 | 3.11E-122 | 91.55 |
| TRINITY_DN12201_c0_g1_i1 | DNA-directed RNA polymerase III subunit RPC7 | 1052 | 2.46E-22 | 60.15 |
| TRINITY_DN12203_c0_g1_i1 | sugar carrier C | 1813 | 0 | 88.9 |
| TRINITY_DN12203_c0_g1_i2 | sugar carrier C | 1832 | 0 | 88.9 |
| TRINITY_DN12220_c0_g1_i1 | hypothetical protein SOVF_058340 | 1297 | 3.27E-111 | 60.05 |
| TRINITY_DN12220_c0_g2_i1 | hypothetical protein SOVF_058340 | 1287 | 1.26E-114 | 60.1 |
| TRINITY_DN12242_c0_g1_i1 | BAG family molecular chaperone regulator 1 | 1244 | 3.17E-133 | 72.45 |
| TRINITY_DN12242_c0_g1_i2 | BAG family molecular chaperone regulator 1 | 1070 | 4.79E-52 | 61.2 |
| TRINITY_DN12243_c0_g1_i1 | PREDICTED: uncharacterized protein LOC104890559 | 1369 | 8.37E-130 | 87.75 |
| TRINITY_DN12245_c0_g1_i2 | CBL-interacting serine threonine- kinase 14-like | 1072 | 2.88E-132 | 83.25 |
| TRINITY_DN12255_c0_g1_i1 | ---NA--- | 248 |  |  |
| TRINITY_DN12255_c0_g1_i2 | ---NA--- | 399 |  |  |
| TRINITY_DN12258_c0_g1_i1 | ---NA--- | 605 |  |  |
| TRINITY_DN12258_c0_g2_i1 | ---NA--- | 293 |  |  |
| TRINITY_DN12264_c0_g1_i1 | ribosome biogenesis BMS1 homolog | 2546 | 0 | 76.3 |
| TRINITY_DN12265_c0_g1_i1 | ---NA--- | 227 |  |  |
| TRINITY_DN12276_c0_g1_i1 | mitochondrial import inner membrane translocase subunit Tim17 Tim22 Tim23 family | 1082 | 2.57E-67 | 60.35 |
| TRINITY_DN12276_c0_g2_i1 | mitochondrial import inner membrane translocase subunit Tim17 Tim22 Tim23 family | 947 | 2.57E-76 | 78.7 |
| TRINITY_DN12280_c0_g1_i1 | Transcription factor CPC | 569 | 7.68E-21 | 78.1 |
| TRINITY_DN12282_c0_g1_i1 | ---NA--- | 359 |  |  |
| TRINITY_DN12282_c0_g4_i1 | ---NA--- | 364 |  |  |
| TRINITY_DN12283_c0_g1_i1 | ---NA--- | 776 |  |  |
| TRINITY_DN12283_c0_g2_i1 | ---NA--- | 776 |  |  |
| TRINITY_DN12292_c0_g1_i1 | ---NA--- | 330 |  |  |
| TRINITY_DN12294_c0_g1_i1 | ---NA--- | 239 |  |  |
| TRINITY_DN12294_c0_g2_i1 | ---NA--- | 239 |  |  |
| TRINITY_DN12294_c0_g3_i1 | ---NA--- | 239 |  |  |
| TRINITY_DN12304_c0_g1_i1 | serine threonine- kinase At5g01020 isoform X1 | 2028 | 0 | 80.5 |
| TRINITY_DN12306_c0_g1_i1 | ---NA--- | 804 |  |  |
| TRINITY_DN12309_c0_g1_i1 | probable cytochrome P450 556A1 | 984 | 4.41E-46 | 70.55 |
| TRINITY_DN12320_c0_g1_i1 | transcription factor bHLH110 | 890 | 8.99E-74 | 67.8 |
| TRINITY_DN12338_c0_g1_i1 | ---NA--- | 262 |  |  |
| TRINITY_DN12354_c0_g1_i1 | probable inactive histone-lysine N-methyltransferase SUVR2 isoform X1 | 1458 | 4.97E-89 | 57.2 |
| TRINITY_DN1235_c0_g1_i1 | ---NA--- | 288 |  |  |
| TRINITY_DN12360_c0_g1_i1 | phytoene synthase | 273 | 7.12E-09 | 78.7 |
| TRINITY_DN12361_c0_g1_i1 | hypothetical protein BVRB_001820 | 486 | 1.76E-13 | 85 |
| TRINITY_DN12361_c0_g2_i1 | hypothetical protein BVRB_001820 | 486 | 1.76E-13 | 85 |
| TRINITY_DN12361_c0_g3_i1 | PREDICTED: uncharacterized protein LOC104883554 | 481 | 3.52E-08 | 85 |
| TRINITY_DN12368_c0_g1_i1 | CBL-interacting kinase 2 | 1660 | 0 | 84.5 |
| TRINITY_DN12372_c0_g1_i1 | ascorbate chloroplastic-like | 796 | 1.65E-20 | 73.45 |
| TRINITY_DN12372_c0_g1_i2 | probable anion transporter chloroplastic | 809 | 1.47E-11 | 61.8 |
| TRINITY_DN12373_c0_g1_i1 | ---NA--- | 240 |  |  |
| TRINITY_DN12374_c0_g1_i1 | nitronate monooxygenase-like | 348 | 2.27E-15 | 91.75 |
| TRINITY_DN12374_c0_g2_i1 | probable nitronate monooxygenase | 1360 | 0 | 90.55 |
| TRINITY_DN12377_c0_g1_i1 | ---NA--- | 222 |  |  |
| TRINITY_DN12377_c0_g2_i1 | ---NA--- | 401 |  |  |
| TRINITY_DN12380_c0_g1_i1 | probable WRKY transcription factor 4 | 1912 | 0 | 66.9 |
| TRINITY_DN12388_c0_g1_i1 | G patch domain-containing 11 | 799 | 5.80E-10 | 84.5 |
| TRINITY_DN12388_c0_g1_i2 | G patch domain-containing 11 | 478 | 7.33E-11 | 84.5 |
| TRINITY_DN12388_c0_g1_i3 | G patch domain-containing 11 | 757 | 4.78E-10 | 84.5 |
| TRINITY_DN12388_c0_g1_i4 | G patch domain-containing 11 | 520 | 1.04E-10 | 84.5 |
| TRINITY_DN1238_c0_g2_i1 | ---NA--- | 220 |  |  |
| TRINITY_DN12395_c0_g1_i1 | probable disease resistance At4g27220 isoform X5 | 1771 | 2.42E-16 | 43.1 |
| TRINITY_DN123_c0_g1_i1 | calcium uptake 1 mitochondrial | 564 | 1.62E-12 | 61 |
| TRINITY_DN12411_c0_g3_i1 | ---NA--- | 250 |  |  |
| TRINITY_DN12411_c0_g4_i2 | ---NA--- | 789 |  |  |
| TRINITY_DN12434_c0_g1_i1 | cytochrome P450 CYP72A219-like | 425 | 3.10E-09 | 69.83 |
| TRINITY_DN12438_c0_g1_i1 | diacylglycerol O-acyltransferase cytosolic | 1624 | 9.47E-38 | 71.1 |
| TRINITY_DN12438_c0_g2_i1 | diacylglycerol O-acyltransferase cytosolic | 1623 | 3.58E-33 | 71.1 |
| TRINITY_DN12447_c0_g1_i1 | PREDICTED: uncharacterized protein LOC104883551 isoform X1 | 1731 | 8.33E-167 | 71.7 |
| TRINITY_DN12449_c0_g1_i1 | hypothetical protein SOVF_134270 | 1002 | 1.83E-10 | 48 |
| TRINITY_DN12451_c0_g2_i1 | ---NA--- | 358 |  |  |
| TRINITY_DN12453_c0_g1_i1 | ---NA--- | 307 |  |  |
| TRINITY_DN12459_c0_g1_i1 | centromere-associated E | 2679 | 4.46E-152 | 53.55 |
| TRINITY_DN12472_c0_g1_i1 | cold-regulated 413 plasma membrane 2-like | 1119 | 2.12E-93 | 80.9 |
| TRINITY_DN1247_c0_g1_i1 | chromo domain-containing LHP1 isoform X2 | 345 | 1.63E-36 | 91.95 |
| TRINITY_DN12490_c0_g1_i1 | beta-glucuronosyltransferase 14A | 807 | 1.29E-64 | 82.45 |
| TRINITY_DN12490_c0_g2_i1 | beta-glucuronosyltransferase 14A | 1815 | 0 | 84.25 |
| TRINITY_DN12496_c0_g1_i2 | ---NA--- | 811 |  |  |
| TRINITY_DN12503_c0_g1_i1 | 3-ketoacyl- synthase 6 | 1976 | 0 | 92.1 |
| TRINITY_DN12503_c0_g2_i1 | 3-ketoacyl- synthase 6 | 1976 | 0 | 92.15 |
| TRINITY_DN12505_c0_g1_i1 | ---NA--- | 743 |  |  |
| TRINITY_DN12509_c0_g1_i1 | pumilio homolog 3-like | 1487 | 6.18E-48 | 93.75 |
| TRINITY_DN12509_c0_g1_i2 | pumilio homolog 3-like | 1668 | 1.84E-47 | 93.75 |
| TRINITY_DN12513_c0_g1_i1 | Cyclin-dependent kinase 1 | 435 | 5.58E-17 | 82.8 |
| TRINITY_DN12517_c0_g1_i1 | homocysteine S-methyltransferase 2 | 1582 | 0 | 87.85 |
| TRINITY_DN12517_c0_g2_i1 | homocysteine S-methyltransferase 2 | 1432 | 0 | 87.85 |
| TRINITY_DN12523_c0_g2_i1 | NUCLEAR FUSION DEFECTIVE 4-like | 1977 | 0 | 76.55 |
| TRINITY_DN12537_c0_g1_i1 | ---NA--- | 614 |  |  |
| TRINITY_DN12537_c0_g1_i2 | ---NA--- | 469 |  |  |
| TRINITY_DN12538_c0_g1_i1 | extensin isoform X2 | 948 | 9.17E-18 | 93.2 |
| TRINITY_DN12538_c0_g2_i1 | extensin isoform X2 | 948 | 9.44E-18 | 93.2 |
| TRINITY_DN12558_c0_g1_i1 | ---NA--- | 237 |  |  |
| TRINITY_DN12564_c0_g4_i1 | transcription factor DIVARICATA-like | 2322 | 4.60E-117 | 78.9 |
| TRINITY_DN12568_c0_g1_i1 | DUF3511 domain | 526 | 2.77E-30 | 60.05 |
| TRINITY_DN12568_c0_g2_i1 | PREDICTED: uncharacterized protein LOC104886656 | 396 | 5.44E-17 | 56 |
| TRINITY_DN12578_c0_g1_i1 | AP2 ERF and B3 domain-containing transcription factor RAV1 | 751 | 2.98E-111 | 76.85 |
| TRINITY_DN12578_c0_g1_i2 | AP2 ERF and B3 domain-containing transcription factor RAV1 | 715 | 2.38E-105 | 76.7 |
| TRINITY_DN12584_c0_g1_i1 | lingerer isoform X1 | 3187 | 0 | 64.85 |
| TRINITY_DN12584_c0_g2_i1 | lingerer isoform X1 | 3188 | 0 | 64.85 |
| TRINITY_DN12595_c0_g1_i1 | tetraspanin-3-like | 1225 | 4.16E-167 | 89.3 |
| TRINITY_DN12596_c0_g1_i1 | transcription factor bHLH104 | 876 | 3.33E-87 | 67.65 |
| TRINITY_DN12596_c0_g1_i4 | transcription factor bHLH104 | 848 | 7.34E-67 | 73.7 |
| TRINITY_DN12600_c0_g1_i1 | hypothetical protein SOVF_135610 | 1556 | 3.09E-50 | 74.1 |
| TRINITY_DN12600_c0_g2_i1 | hypothetical protein SOVF_135610 | 1574 | 3.47E-50 | 74.1 |
| TRINITY_DN12603_c0_g1_i1 | PREDICTED: uncharacterized protein LOC104901373 | 1357 | 3.70E-134 | 68.4 |
| TRINITY_DN12603_c0_g1_i2 | PREDICTED: uncharacterized protein LOC104901373 | 1318 | 9.10E-123 | 65.65 |
| TRINITY_DN12614_c0_g2_i1 | cyclin-D4-2-like isoform X1 | 1697 | 8.15E-135 | 73.25 |
| TRINITY_DN12614_c0_g2_i2 | cyclin-D4-2-like isoform X1 | 1832 | 9.18E-138 | 73.2 |
| TRINITY_DN12619_c0_g1_i1 | hydroquinone glucosyltransferase-like | 1979 | 0 | 72.6 |
| TRINITY_DN12627_c0_g1_i1 | HKT1 2 transporter | 566 | 1.19E-44 | 81.75 |
| TRINITY_DN12627_c0_g2_i1 | HKT1 2 transporter | 438 | 6.84E-20 | 92.35 |
| TRINITY_DN12627_c1_g1_i1 | ---NA--- | 439 |  |  |
| TRINITY_DN12646_c0_g1_i1 | DUF1645 domain-containing | 871 | 2.47E-22 | 50 |
| TRINITY_DN12650_c0_g1_i1 | ---NA--- | 218 |  |  |
| TRINITY_DN12662_c0_g1_i1 | ethylene-responsive transcription factor 4-like | 1143 | 1.25E-28 | 74.9 |
| TRINITY_DN12671_c0_g1_i1 | endoplasmin homolog | 2975 | 0 | 92.75 |
| TRINITY_DN12677_c0_g1_i1 | chorismate mutase 2 isoform X2 | 1226 | 1.87E-119 | 72.9 |
| TRINITY_DN12679_c0_g1_i1 | probable serine threonine- kinase RLCKVII | 1046 | 2.06E-116 | 86.5 |
| TRINITY_DN12679_c0_g1_i2 | probable serine threonine- kinase RLCKVII | 1155 | 2.49E-116 | 86.25 |
| TRINITY_DN12697_c0_g1_i1 | ---NA--- | 274 |  |  |
| TRINITY_DN12698_c0_g1_i1 | ---NA--- | 528 |  |  |
| TRINITY_DN12698_c0_g2_i1 | uroporphyrinogen-III C-methyltransferase | 1845 | 3.65E-167 | 81.65 |
| TRINITY_DN12700_c0_g1_i1 | hypothetical protein BVRB_5g125510 | 692 | 2.32E-32 | 62.15 |
| TRINITY_DN12700_c0_g1_i2 | hypothetical protein BVRB_5g125510 | 647 | 1.11E-27 | 62.05 |
| TRINITY_DN12702_c0_g1_i1 | AT-hook motif nuclear-localized 5 | 1311 | 1.18E-99 | 73.55 |
| TRINITY_DN12702_c0_g2_i1 | AT-hook motif nuclear-localized 5 | 375 | 5.24E-16 | 77.7 |
| TRINITY_DN12704_c1_g1_i1 | PREDICTED: uncharacterized protein LOC104885201 | 866 | 2.32E-40 | 88.55 |
| TRINITY_DN12706_c0_g1_i1 | VHS domain-containing At3g16270 | 2661 | 0 | 73.6 |
| TRINITY_DN12711_c0_g1_i1 | CONSTANS-like 5 | 1551 | 1.19E-136 | 63.9 |
| TRINITY_DN12726_c0_g1_i1 | ---NA--- | 240 |  |  |
| TRINITY_DN12728_c1_g1_i1 | digalactosyldiacylglycerol synthase chloroplastic | 819 | 6.81E-67 | 68.55 |
| TRINITY_DN12728_c1_g2_i1 | digalactosyldiacylglycerol synthase chloroplastic | 2884 | 0 | 83.55 |
| TRINITY_DN12732_c0_g1_i1 | CBL-interacting serine threonine- kinase 12 | 1695 | 0 | 86.25 |
| TRINITY_DN12734_c0_g1_i1 | calvin cycle CP12- chloroplastic-like | 670 | 2.50E-49 | 78.75 |
| TRINITY_DN12740_c0_g1_i1 | methyltransferase 6 | 1126 | 4.11E-101 | 75.8 |
| TRINITY_DN12740_c0_g1_i2 | methyltransferase 6 | 1227 | 3.95E-148 | 77.35 |
| TRINITY_DN12744_c0_g1_i1 | ---NA--- | 505 |  |  |
| TRINITY_DN12750_c0_g1_i1 | zinc finger CCHC domain-containing 10-like | 1250 | 1.87E-51 | 80.1 |
| TRINITY_DN12758_c0_g1_i1 | ---NA--- | 417 |  |  |
| TRINITY_DN12758_c0_g2_i1 | ---NA--- | 224 |  |  |
| TRINITY_DN12758_c0_g3_i1 | ---NA--- | 806 |  |  |
| TRINITY_DN12758_c0_g3_i2 | ---NA--- | 826 |  |  |
| TRINITY_DN12758_c0_g3_i3 | ---NA--- | 909 |  |  |
| TRINITY_DN12766_c0_g1_i1 | probable pre-mRNA-splicing factor ATP-dependent RNA helicase DEAH9 isoform X1 | 2300 | 0 | 89.65 |
| TRINITY_DN12775_c0_g2_i1 | ---NA--- | 203 |  |  |
| TRINITY_DN12782_c0_g1_i1 | heavy metal-associated isoprenylated plant 30 | 709 | 2.34E-101 | 83.9 |
| TRINITY_DN12782_c0_g2_i1 | heavy metal-associated isoprenylated plant 30 | 612 | 8.73E-66 | 90.8 |
| TRINITY_DN12785_c0_g1_i1 | ---NA--- | 327 |  |  |
| TRINITY_DN12785_c0_g1_i2 | ---NA--- | 423 |  |  |
| TRINITY_DN12790_c0_g1_i2 | AT-hook motif nuclear-localized 1 | 653 | 1.83E-45 | 79.9 |
| TRINITY_DN1279_c0_g1_i1 | kDa proline-rich -like | 437 | 8.94E-29 | 89.65 |
| TRINITY_DN1279_c0_g2_i1 | kDa proline-rich -like | 437 | 3.51E-29 | 89.5 |
| TRINITY_DN127_c0_g1_i1 | probable L-type lectin-domain containing receptor kinase | 1225 | 7.83E-135 | 67.4 |
| TRINITY_DN127_c0_g2_i1 | probable L-type lectin-domain containing receptor kinase | 1109 | 9.67E-116 | 68.45 |
| TRINITY_DN12802_c0_g1_i1 | glutamine synthetase leaf chloroplastic | 2064 | 0 | 93.45 |
| TRINITY_DN12805_c0_g1_i1 | major allergen Pru ar 1 | 224 | 8.40E-24 | 70.3 |
| TRINITY_DN12805_c0_g2_i1 | major allergen Pru ar 1-like | 227 | 2.73E-27 | 73.65 |
| TRINITY_DN12808_c0_g1_i1 | probable leucine-rich repeat receptor kinase At2g33170 | 1023 | 3.45E-173 | 87.1 |
| TRINITY_DN1280_c0_g2_i1 | trihelix transcription factor ASR3-like | 617 | 3.97E-31 | 68.6 |
| TRINITY_DN12818_c0_g1_i1 | PREDICTED: uncharacterized protein DDB_G0283357 | 1139 | 8.18E-17 | 57 |
| TRINITY_DN12826_c0_g1_i1 | pentatricopeptide repeat-containing mitochondrial | 2069 | 0 | 76.4 |
| TRINITY_DN12827_c0_g1_i1 | telomere repeat-binding factor 4 | 1295 | 1.12E-78 | 62.75 |
| TRINITY_DN12832_c2_g1_i1 | lactoylglutathione lyase family | 928 | 1.67E-64 | 86.05 |
| TRINITY_DN12832_c2_g2_i1 | lactoylglutathione lyase family | 927 | 1.67E-64 | 86.05 |
| TRINITY_DN12832_c2_g3_i1 | lactoylglutathione lyase family | 767 | 2.76E-65 | 86.05 |
| TRINITY_DN12849_c0_g1_i1 | hypothetical protein SOVF_162080 | 766 | 2.18E-15 | 70.25 |
| TRINITY_DN12851_c0_g1_i1 | ---NA--- | 207 |  |  |
| TRINITY_DN12851_c0_g2_i1 | ---NA--- | 233 |  |  |
| TRINITY_DN12852_c0_g2_i1 | transmembrane (DUF3317) | 855 | 5.66E-08 | 95.15 |
| TRINITY_DN12862_c0_g1_i1 | mediator of RNA polymerase II transcription subunit 8 | 992 | 5.93E-152 | 79.6 |
| TRINITY_DN12865_c0_g1_i2 | NADPH-dependent 1-acyldihydroxyacetone phosphate reductase | 813 | 1.33E-94 | 83.8 |
| TRINITY_DN12866_c0_g1_i1 | lysM domain receptor-like kinase 3 | 1605 | 0 | 76 |
| TRINITY_DN12866_c0_g2_i1 | lysM domain receptor-like kinase 3 | 1705 | 0 | 76 |
| TRINITY_DN12867_c0_g1_i1 | membrane-anchored ubiquitin-fold 4 | 830 | 2.75E-46 | 83.25 |
| TRINITY_DN12871_c0_g1_i1 | PREDICTED: uncharacterized protein LOC109134285, partial | 761 | 2.36E-100 | 78.4 |
| TRINITY_DN12875_c1_g1_i1 | serine--glyoxylate aminotransferase-like | 4073 | 0 | 95.6 |
| TRINITY_DN12876_c0_g2_i1 | ---NA--- | 490 |  |  |
| TRINITY_DN12880_c0_g1_i1 | ---NA--- | 678 |  |  |
| TRINITY_DN12880_c0_g2_i1 | uncharacterized vacuolar membrane YML018C-like | 679 | 1.45E-61 | 94.75 |
| TRINITY_DN12880_c0_g2_i2 | uncharacterized vacuolar membrane YML018C-like | 1535 | 0 | 85 |
| TRINITY_DN12884_c0_g1_i1 | bfr2 isoform X2 | 1089 | 1.02E-14 | 71 |
| TRINITY_DN12884_c1_g1_i1 | hypothetical protein SOVF_055620 | 1012 | 1.12E-32 | 70.35 |
| TRINITY_DN12887_c0_g1_i1 | CASC3 Barentsz eIF4AIII isoform 1 | 531 | 9.85E-10 | 63.2 |
| TRINITY_DN12887_c0_g2_i1 | ---NA--- | 715 |  |  |
| TRINITY_DN12898_c0_g1_i1 | ---NA--- | 240 |  |  |
| TRINITY_DN1289_c0_g1_i1 | fasciclin-like arabinogalactan 21 | 499 | 6.14E-14 | 65.45 |
| TRINITY_DN128_c0_g1_i1 | WPP domain-interacting tail-anchored 1 | 1305 | 5.72E-139 | 63.75 |
| TRINITY_DN128_c0_g2_i1 | WPP domain-interacting tail-anchored 1 | 1302 | 9.00E-143 | 63.75 |
| TRINITY_DN12900_c0_g1_i1 | zinc finger CONSTANS-LIKE 13 | 1574 | 1.33E-144 | 62 |
| TRINITY_DN12902_c0_g1_i1 | UPF0057 membrane At4g30660-like | 546 | 3.13E-37 | 85.35 |
| TRINITY_DN12916_c0_g1_i1 | ---NA--- | 531 |  |  |
| TRINITY_DN12916_c0_g1_i2 | ---NA--- | 559 |  |  |
| TRINITY_DN12927_c0_g1_i1 | Aminoglycoside phosphotransferase | 410 | 1.28E-11 | 88 |
| TRINITY_DN12930_c0_g1_i1 | peptide deformylase chloroplastic | 1356 | 2.68E-88 | 89.05 |
| TRINITY_DN12931_c0_g1_i1 | glucan endo-1,3-beta-glucosidase-like | 599 | 3.38E-48 | 68.2 |
| TRINITY_DN12931_c0_g2_i1 | hypothetical protein SOVF_057490 | 276 | 7.14E-06 | 88 |
| TRINITY_DN12970_c0_g1_i1 | Retrovirus-related Pol poly from transposon TNT 1- | 466 | 1.92E-18 | 80.2 |
| TRINITY_DN12975_c0_g1_i1 | ---NA--- | 799 |  |  |
| TRINITY_DN12975_c0_g2_i1 | ---NA--- | 571 |  |  |
| TRINITY_DN12976_c0_g3_i1 | beta-hydroxyacyl-ACP dehydratase | 1036 | 2.34E-104 | 96.15 |
| TRINITY_DN1297_c0_g1_i1 | PREDICTED: uncharacterized protein LOC104897560 isoform X1 | 919 | 5.20E-103 | 76.4 |
| TRINITY_DN12986_c0_g1_i1 | ras-related Rab7 | 1028 | 4.00E-143 | 96.85 |
| TRINITY_DN12987_c0_g1_i1 | probable WRKY transcription factor 48 | 722 | 2.67E-40 | 54.55 |
| TRINITY_DN12987_c0_g1_i2 | probable WRKY transcription factor 48 | 1253 | 4.17E-57 | 61.1 |
| TRINITY_DN12987_c0_g2_i1 | probable WRKY transcription factor 23 | 393 | 5.24E-08 | 57.33 |
| TRINITY_DN12989_c0_g2_i1 | Nucleic acid isoform | 305 | 1.12E-12 | 82.95 |
| TRINITY_DN12989_c0_g3_i1 | ubiquitin system component Cue domain-containing family | 744 | 2.41E-96 | 77.45 |
| TRINITY_DN13000_c0_g1_i1 | ---NA--- | 418 |  |  |
| TRINITY_DN13000_c0_g3_i1 | ---NA--- | 402 |  |  |
| TRINITY_DN13005_c0_g1_i1 | ---NA--- | 975 |  |  |
| TRINITY_DN13010_c0_g1_i1 | regulatory NPR1 | 964 | 2.24E-75 | 76.8 |
| TRINITY_DN13010_c0_g2_i1 | Regulatory NPR1 | 576 | 1.68E-15 | 65.85 |
| TRINITY_DN13011_c0_g2_i1 | transcriptional corepressor SEUSS-like | 1034 | 2.18E-77 | 62.15 |
| TRINITY_DN13011_c0_g2_i2 | transcriptional corepressor SEUSS-like | 1048 | 5.55E-83 | 62.25 |
| TRINITY_DN13011_c1_g1_i1 | Photosystem II | 1077 | 5.57E-45 | 77.9 |
| TRINITY_DN13013_c0_g1_i1 | ---NA--- | 351 |  |  |
| TRINITY_DN1301_c0_g1_i1 | ---NA--- | 206 |  |  |
| TRINITY_DN1302_c0_g1_i1 | ---NA--- | 213 |  |  |
| TRINITY_DN13031_c0_g1_i1 | phospholipase D alpha 1 | 737 | 2.41E-83 | 79.5 |
| TRINITY_DN13036_c0_g1_i1 | 21 kDa | 790 | 4.01E-70 | 72.5 |
| TRINITY_DN13043_c0_g1_i1 | REF SRPP At3g05500 | 507 | 1.57E-50 | 76.8 |
| TRINITY_DN13043_c0_g2_i1 | REF SRPP At3g05500 | 1093 | 1.30E-126 | 80.9 |
| TRINITY_DN1304_c0_g1_i1 | ---NA--- | 243 |  |  |
| TRINITY_DN13056_c0_g1_i1 | transcription factor TCP14-like | 1408 | 4.66E-115 | 62.25 |
| TRINITY_DN13056_c0_g1_i2 | transcription factor TCP14-like | 1190 | 1.57E-104 | 61.6 |
| TRINITY_DN13056_c1_g1_i1 | LATERAL ROOT PRIMORDIUM 1-like | 548 | 3.20E-44 | 75.65 |
| TRINITY_DN13056_c2_g1_i1 | auxin response factor 18-like | 726 | 4.25E-69 | 69 |
| TRINITY_DN13062_c0_g1_i1 | 26S proteasome non-ATPase regulatory subunit 14 homolog | 1183 | 0 | 97.15 |
| TRINITY_DN13066_c0_g1_i1 | chloroplastic | 2780 | 0 | 94.35 |
| TRINITY_DN13066_c0_g2_i1 | chloroplastic | 2123 | 0 | 94.35 |
| TRINITY_DN13068_c0_g1_i1 | 50S ribosomal chloroplastic | 973 | 8.63E-90 | 93.9 |
| TRINITY_DN13070_c0_g1_i1 | CRM-domain containing factor chloroplastic mitochondrial | 2396 | 0 | 79.3 |
| TRINITY_DN13070_c0_g2_i1 | CRM-domain containing factor chloroplastic mitochondrial | 2463 | 0 | 79.3 |
| TRINITY_DN13070_c0_g3_i1 | CRM-domain containing factor chloroplastic mitochondrial | 2433 | 0 | 79.3 |
| TRINITY_DN13071_c0_g1_i1 | chlorophyll a-b binding chloroplastic | 1347 | 8.39E-145 | 88.05 |
| TRINITY_DN13076_c0_g1_i1 | ---NA--- | 2865 |  |  |
| TRINITY_DN13076_c0_g2_i1 | ---NA--- | 1754 |  |  |
| TRINITY_DN13077_c0_g1_i1 | 12-oxophytodienoate reductase 11 | 1562 | 0 | 89.35 |
| TRINITY_DN13078_c0_g1_i1 | 40S ribosomal S13 | 871 | 7.76E-102 | 98.4 |
| TRINITY_DN13078_c0_g3_i1 | 40S ribosomal S13 | 840 | 5.17E-102 | 98.4 |
| TRINITY_DN13087_c1_g1_i1 | ---NA--- | 443 |  |  |
| TRINITY_DN13091_c0_g1_i1 | haloacid dehalogenase-like hydrolase domain-containing At3g48420 isoform X1 | 1591 | 1.14E-151 | 78.65 |
| TRINITY_DN13092_c0_g1_i1 | ---NA--- | 563 |  |  |
| TRINITY_DN13092_c0_g2_i1 | ---NA--- | 562 |  |  |
| TRINITY_DN13114_c0_g1_i1 | pyrophosphate-energized vacuolar membrane proton pump | 522 | 1.26E-28 | 77 |
| TRINITY_DN13114_c0_g1_i2 | pyrophosphate-energized vacuolar membrane proton pump-like | 684 | 1.18E-110 | 88.7 |
| TRINITY_DN13116_c0_g1_i1 | HSP20-like chaperone | 1031 | 2.04E-53 | 85.45 |
| TRINITY_DN13120_c0_g1_i1 | axial regulator YABBY 5-like isoform X4 | 778 | 4.33E-57 | 69.75 |
| TRINITY_DN13129_c0_g10_i1 | flavanone 3- | 336 | 1.14E-44 | 79.6 |
| TRINITY_DN13129_c0_g11_i1 | ---NA--- | 203 |  |  |
| TRINITY_DN13129_c0_g12_i1 | naringenin,2-oxoglutarate 3-dioxygenase | 1293 | 0 | 80.15 |
| TRINITY_DN13129_c0_g13_i1 | flavanone 3- | 434 | 5.55E-44 | 79.9 |
| TRINITY_DN13129_c0_g14_i1 | flavanone 3- | 437 | 4.45E-44 | 79.9 |
| TRINITY_DN13129_c0_g15_i1 | flavanone 3- | 349 | 1.34E-44 | 79.6 |
| TRINITY_DN13129_c0_g16_i1 | flavanone 3- | 440 | 4.29E-44 | 79.9 |
| TRINITY_DN13129_c0_g1_i1 | ---NA--- | 208 |  |  |
| TRINITY_DN13129_c0_g2_i1 | flavanone 3- | 339 | 1.19E-44 | 79.6 |
| TRINITY_DN13129_c0_g3_i1 | flavanone 3- | 335 | 1.10E-44 | 79.6 |
| TRINITY_DN13129_c0_g4_i1 | flavanone 3- | 337 | 1.14E-44 | 79.6 |
| TRINITY_DN13129_c0_g5_i1 | flavanone 3- | 431 | 1.53E-47 | 79.9 |
| TRINITY_DN13129_c0_g6_i1 | flavanone 3- | 341 | 1.19E-44 | 79.6 |
| TRINITY_DN13129_c0_g7_i1 | flavanone 3- | 432 | 2.54E-47 | 79.85 |
| TRINITY_DN13129_c0_g8_i1 | flavanone 3- | 430 | 1.02E-43 | 79.85 |
| TRINITY_DN13129_c0_g9_i1 | flavanone 3- | 338 | 1.14E-44 | 79.6 |
| TRINITY_DN13130_c0_g1_i1 | photosystem II 22 kDa chloroplastic | 1366 | 1.77E-115 | 82.65 |
| TRINITY_DN13130_c0_g2_i1 | photosystem II 22 kDa chloroplastic | 1366 | 1.77E-115 | 82.65 |
| TRINITY_DN13138_c0_g1_i1 | ATP-dependent Clp protease proteolytic subunit chloroplastic | 1329 | 1.53E-140 | 81.85 |
| TRINITY_DN13160_c0_g1_i1 | transcription factor PCL1-like | 1814 | 5.84E-76 | 61.4 |
| TRINITY_DN13161_c0_g1_i1 | hypothetical protein SOVF_127000 | 1226 | 0 | 78.15 |
| TRINITY_DN13162_c0_g1_i1 | indeterminate-domain chloroplastic-like | 1376 | 4.16E-113 | 77.65 |
| TRINITY_DN13175_c0_g1_i1 | ferredoxin-thioredoxin reductase catalytic chloroplastic | 883 | 1.25E-86 | 91.15 |
| TRINITY_DN13175_c0_g2_i1 | ferredoxin-thioredoxin reductase catalytic chloroplastic | 874 | 8.87E-82 | 91.15 |
| TRINITY_DN13184_c0_g1_i1 | glycine-rich family | 1993 | 6.92E-107 | 66.65 |
| TRINITY_DN13191_c0_g1_i1 | probable sulfate transporter | 2245 | 0 | 87.4 |
| TRINITY_DN13198_c0_g1_i1 | UVR domain-containing DUF525 domain-containing | 1312 | 3.31E-132 | 91.3 |
| TRINITY_DN13215_c0_g1_i1 | hypothetical protein SOVF_085780 | 492 | 5.13E-14 | 80.6 |
| TRINITY_DN13220_c0_g1_i1 | ---NA--- | 545 |  |  |
| TRINITY_DN13225_c0_g1_i1 | rho-N domain-containing chloroplastic | 589 | 7.02E-18 | 73.5 |
| TRINITY_DN13225_c0_g2_i1 | rho-N domain-containing chloroplastic | 586 | 5.75E-16 | 82 |
| TRINITY_DN13232_c0_g1_i1 | NLP9-like | 3607 | 0 | 65.5 |
| TRINITY_DN13238_c0_g1_i1 | PREDICTED: uncharacterized protein LOC104889004 isoform X2 | 2261 | 0 | 64.55 |
| TRINITY_DN1323_c0_g1_i1 | ---NA--- | 300 |  |  |
| TRINITY_DN13261_c0_g1_i2 | Tricin synthase 1 | 1785 | 1.06E-115 | 88.3 |
| TRINITY_DN13265_c1_g2_i1 | ubiquitin-60S ribosomal L40 | 688 | 3.92E-88 | 98.85 |
| TRINITY_DN13267_c1_g3_i1 | PREDICTED: uncharacterized protein LOC104905839 | 1443 | 1.07E-35 | 60.6 |
| TRINITY_DN13273_c0_g1_i1 | hypothetical protein POPTR_0002s01800g | 482 | 1.39E-13 | 73.2 |
| TRINITY_DN13278_c0_g2_i1 | ---NA--- | 828 |  |  |
| TRINITY_DN13278_c0_g3_i1 | ---NA--- | 579 |  |  |
| TRINITY_DN13281_c0_g1_i1 | ubiquitin-conjugating enzyme E2-17 kDa | 671 | 1.63E-104 | 97.6 |
| TRINITY_DN13290_c0_g1_i1 | B-cell receptor-associated 31 | 792 | 1.87E-52 | 76.65 |
| TRINITY_DN13290_c0_g2_i1 | B-cell receptor-associated 31 | 786 | 2.51E-52 | 76.65 |
| TRINITY_DN13291_c0_g1_i1 | ---NA--- | 238 |  |  |
| TRINITY_DN13292_c0_g1_i1 | ethylene-responsive transcription factor 5 | 1207 | 3.52E-39 | 60.6 |
| TRINITY_DN13292_c0_g2_i1 | ethylene-responsive transcription factor 5 | 1174 | 5.87E-38 | 54.95 |
| TRINITY_DN13300_c0_g1_i1 | 2-oxoglutarate (2OG) and Fe(II)-dependent oxygenase superfamily isoform 1 | 2160 | 0 | 76.95 |
| TRINITY_DN13300_c0_g1_i2 | 2-oxoglutarate (2OG) and Fe(II)-dependent oxygenase superfamily isoform 1 | 2157 | 0 | 77.6 |
| TRINITY_DN13300_c0_g1_i3 | 2-oxoglutarate (2OG) and Fe(II)-dependent oxygenase superfamily isoform 1 | 2128 | 0 | 81.4 |
| TRINITY_DN13300_c0_g2_i1 | 2-oxoglutarate (2OG) and Fe(II)-dependent oxygenase superfamily isoform 1 | 849 | 7.32E-101 | 75.2 |
| TRINITY_DN13301_c0_g1_i1 | F-box kelch-repeat SKIP30 | 1500 | 0 | 84.95 |
| TRINITY_DN13303_c0_g1_i1 | glutathione S-transferase zeta class isoform X1 | 1168 | 1.48E-93 | 80.7 |
| TRINITY_DN13303_c0_g2_i1 | glutathione S-transferase 2 isoform X1 | 1302 | 1.94E-51 | 79.3 |
| TRINITY_DN13304_c0_g3_i1 | ---NA--- | 313 |  |  |
| TRINITY_DN13304_c0_g3_i2 | ---NA--- | 434 |  |  |
| TRINITY_DN13310_c0_g1_i1 | 60S ribosomal L23A | 987 | 9.55E-69 | 94.4 |
| TRINITY_DN13310_c0_g1_i2 | 60S ribosomal L23A | 1017 | 1.31E-68 | 94.4 |
| TRINITY_DN13311_c1_g1_i1 | ---NA--- | 483 |  |  |
| TRINITY_DN13318_c0_g1_i1 | histone-lysine N-methyltransferase setd3 | 1576 | 0 | 79.05 |
| TRINITY_DN13318_c0_g1_i2 | histone-lysine N-methyltransferase setd3 | 1627 | 0 | 76.3 |
| TRINITY_DN13320_c0_g1_i1 | hypothetical protein SOVF_158120 | 1503 | 1.72E-25 | 59.55 |
| TRINITY_DN13322_c0_g1_i1 | BTB POZ domain-containing At1g67900-like | 1625 | 0 | 79.65 |
| TRINITY_DN13342_c0_g1_i1 | kinesin identical | 1107 | 9.01E-55 | 68 |
| TRINITY_DN13353_c0_g1_i1 | ---NA--- | 281 |  |  |
| TRINITY_DN1335_c0_g1_i1 | probable inactive receptor kinase At4g23740 | 1310 | 0 | 88.3 |
| TRINITY_DN13361_c0_g1_i1 | 3Fe-4S ferredoxin | 1477 | 1.58E-178 | 81.2 |
| TRINITY_DN13379_c0_g1_i1 | S-formylglutathione hydrolase | 1246 | 0 | 90.8 |
| TRINITY_DN13383_c0_g1_i1 | nuclear transcription factor Y subunit B-3-like | 1518 | 2.68E-69 | 93.3 |
| TRINITY_DN13392_c0_g1_i1 | lysosomal Pro-X carboxypeptidase-like | 2139 | 0 | 83.15 |
| TRINITY_DN13395_c0_g1_i1 | ---NA--- | 969 |  |  |
| TRINITY_DN13395_c0_g2_i1 | ---NA--- | 1714 |  |  |
| TRINITY_DN13410_c0_g1_i1 | 29 kDa ribonucleo chloroplastic-like | 1532 | 2.83E-111 | 76.2 |
| TRINITY_DN13414_c0_g1_i1 | hypothetical protein SOVF_152260 | 636 | 1.75E-09 | 64 |
| TRINITY_DN13414_c1_g1_i1 | ---NA--- | 283 |  |  |
| TRINITY_DN13434_c0_g1_i1 | 40S ribosomal S16 | 824 | 3.21E-95 | 94.15 |
| TRINITY_DN13448_c0_g1_i1 | ---NA--- | 963 |  |  |
| TRINITY_DN13448_c0_g1_i2 | ---NA--- | 1007 |  |  |
| TRINITY_DN1344_c0_g1_i1 | subtilisin-like protease | 3107 | 0 | 91.5 |
| TRINITY_DN13461_c0_g1_i1 | SMAX1-LIKE 6-like | 3594 | 0 | 56.5 |
| TRINITY_DN13461_c0_g2_i1 | SMAX1-LIKE 6-like | 3599 | 0 | 60.85 |
| TRINITY_DN13462_c0_g1_i1 | tRNA wybutosine-synthesizing 2 3 4 isoform X1 | 3200 | 0 | 72.35 |
| TRINITY_DN13463_c0_g1_i1 | eukaryotic translation initiation factor 3 subunit E | 1666 | 0 | 94 |
| TRINITY_DN13464_c0_g10_i1 | DUF538 domain-containing | 1393 | 1.40E-67 | 76.35 |
| TRINITY_DN13464_c0_g11_i1 | DUF538 domain-containing | 1389 | 1.09E-71 | 76.35 |
| TRINITY_DN13464_c0_g12_i1 | DUF538 domain-containing | 1391 | 2.58E-67 | 76.35 |
| TRINITY_DN13464_c0_g1_i1 | DUF538 domain-containing | 1399 | 2.72E-67 | 76.35 |
| TRINITY_DN13464_c0_g2_i1 | DUF538 domain-containing | 1394 | 2.65E-67 | 76.35 |
| TRINITY_DN13464_c0_g3_i1 | DUF538 domain-containing | 1397 | 2.72E-67 | 76.35 |
| TRINITY_DN13464_c0_g4_i1 | DUF538 domain-containing | 1396 | 2.65E-67 | 76.35 |
| TRINITY_DN13464_c0_g5_i1 | DUF538 domain-containing | 1398 | 1.51E-71 | 76.35 |
| TRINITY_DN13464_c0_g6_i1 | DUF538 domain-containing | 1392 | 2.15E-67 | 76.35 |
| TRINITY_DN13464_c0_g7_i1 | DUF538 domain-containing | 848 | 1.06E-69 | 76.35 |
| TRINITY_DN13464_c0_g8_i1 | DUF538 domain-containing | 1390 | 1.36E-67 | 76.35 |
| TRINITY_DN13464_c0_g9_i1 | DUF538 domain-containing | 735 | 2.81E-70 | 76.35 |
| TRINITY_DN13468_c0_g1_i1 | ORF able to induce HR-like lesions | 751 | 2.29E-44 | 88.45 |
| TRINITY_DN13475_c0_g2_i1 | aspartic ase | 2156 | 0 | 85.1 |
| TRINITY_DN13483_c0_g2_i1 | SMAX1-LIKE 8 | 2192 | 8.93E-153 | 54.3 |
| TRINITY_DN13486_c0_g1_i1 | zf-C3HC4_3 domain-containing | 1764 | 1.44E-136 | 63.45 |
| TRINITY_DN13486_c0_g2_i1 | zf-C3HC4_3 domain-containing | 1911 | 3.10E-171 | 73.2 |
| TRINITY_DN1349_c0_g1_i1 | B3 domain-containing At5g42700 | 312 | 2.74E-08 | 86.67 |
| TRINITY_DN13502_c0_g1_i1 | hypothetical protein SOVF_133760 | 697 | 5.29E-15 | 90.79 |
| TRINITY_DN13502_c0_g2_i1 | hypothetical protein SOVF_133760 | 472 | 8.89E-16 | 90.79 |
| TRINITY_DN13505_c0_g2_i1 | yippee-like At4g27745 | 458 | 3.21E-38 | 86.75 |
| TRINITY_DN13509_c0_g1_i1 | rho GTPase-activating 5-like | 258 | 8.44E-30 | 84.2 |
| TRINITY_DN13511_c0_g1_i1 | ---NA--- | 961 |  |  |
| TRINITY_DN13524_c0_g1_i1 | probable purine permease 11 | 1597 | 2.56E-160 | 78.1 |
| TRINITY_DN13530_c0_g1_i1 | myb-related 308-like | 1244 | 2.70E-148 | 77.45 |
| TRINITY_DN13534_c0_g1_i1 | salicylate carboxymethyltransferase-like | 974 | 3.60E-144 | 66.85 |
| TRINITY_DN13549_c0_g1_i1 | late embryogenesis abundant | 1538 | 3.54E-107 | 53.3 |
| TRINITY_DN13551_c0_g2_i1 | ---NA--- | 317 |  |  |
| TRINITY_DN13560_c0_g1_i1 | 3-ketoacyl- synthase 12-like | 663 | 8.68E-84 | 87.55 |
| TRINITY_DN13576_c1_g1_i1 | ---NA--- | 633 |  |  |
| TRINITY_DN1357_c0_g1_i1 | ---NA--- | 250 |  |  |
| TRINITY_DN13590_c0_g1_i1 | transcription factor MYC2-like | 2246 | 3.97E-101 | 70.65 |
| TRINITY_DN13594_c0_g1_i1 | small multi-drug export | 1482 | 2.29E-49 | 92.6 |
| TRINITY_DN13594_c0_g1_i2 | small multi-drug export | 1382 | 2.27E-105 | 71.95 |
| TRINITY_DN13603_c0_g1_i1 | pentatricopeptide repeat-containing mitochondrial-like | 958 | 2.92E-103 | 58 |
| TRINITY_DN13603_c0_g2_i1 | pentatricopeptide repeat-containing mitochondrial-like | 1081 | 5.37E-127 | 66.15 |
| TRINITY_DN13604_c0_g1_i1 | hypothetical protein BVRB_2g045650 | 1065 | 4.11E-49 | 67.4 |
| TRINITY_DN13625_c0_g1_i1 | ---NA--- | 353 |  |  |
| TRINITY_DN13625_c0_g1_i2 | ---NA--- | 331 |  |  |
| TRINITY_DN13637_c0_g1_i1 | limonoid UDP-glucosyltransferase | 545 | 2.92E-80 | 82.65 |
| TRINITY_DN13637_c0_g2_i1 | limonoid UDP-glucosyltransferase | 1354 | 0 | 79.3 |
| TRINITY_DN13645_c0_g1_i1 | LOV domain-containing | 1913 | 0 | 90.15 |
| TRINITY_DN13645_c1_g1_i1 | pentatricopeptide repeat-containing At1g02150 | 1851 | 0 | 80.8 |
| TRINITY_DN13650_c0_g1_i1 | general transcription factor IIE subunit 2-like | 1481 | 5.31E-111 | 90.85 |
| TRINITY_DN13650_c0_g1_i2 | general transcription factor IIE subunit 2-like | 1509 | 1.84E-146 | 83.1 |
| TRINITY_DN13654_c0_g1_i1 | cytochrome c oxidase subunit 5b- mitochondrial | 1363 | 1.98E-46 | 85.95 |
| TRINITY_DN13655_c0_g1_i1 | ---NA--- | 303 |  |  |
| TRINITY_DN13659_c0_g1_i1 | DNA gyrase subunit chloroplastic mitochondrial | 2621 | 0 | 91.25 |
| TRINITY_DN1365_c0_g1_i1 | auxin-responsive SAUR40 | 717 | 1.63E-48 | 72.5 |
| TRINITY_DN1365_c0_g2_i1 | auxin-responsive SAUR40 | 716 | 1.12E-48 | 72.6 |
| TRINITY_DN13662_c0_g3_i1 | ---NA--- | 314 |  |  |
| TRINITY_DN13662_c0_g4_i1 | ---NA--- | 468 |  |  |
| TRINITY_DN13667_c0_g1_i1 | PLASMODESMATA CALLOSE-BINDING PROTEIN 3 | 701 | 1.05E-30 | 65.95 |
| TRINITY_DN13667_c0_g2_i1 | PLASMODESMATA CALLOSE-BINDING PROTEIN 3 | 689 | 9.37E-31 | 65.95 |
| TRINITY_DN1366_c0_g1_i1 | bifunctional UDP-glucose 4-epimerase and UDP-xylose 4-epimerase 1 | 238 | 7.05E-09 | 86.5 |
| TRINITY_DN13670_c0_g1_i1 | ---NA--- | 205 |  |  |
| TRINITY_DN13674_c0_g1_i1 | probable dolichyl pyrophosphate Man9 c2 alpha-1,3-glucosyltransferase | 2058 | 0 | 80.8 |
| TRINITY_DN13687_c0_g1_i1 | ---NA--- | 574 |  |  |
| TRINITY_DN13687_c0_g2_i1 | ---NA--- | 574 |  |  |
| TRINITY_DN13692_c0_g1_i1 | Nucleoporin seh1 | 500 | 3.30E-42 | 74.2 |
| TRINITY_DN13692_c0_g2_i1 | SEH1 | 1327 | 6.11E-174 | 79.65 |
| TRINITY_DN13694_c0_g1_i1 | transcription repressor MYB6-like | 1272 | 0 | 67.6 |
| TRINITY_DN13696_c0_g1_i1 | ---NA--- | 211 |  |  |
| TRINITY_DN13696_c0_g2_i1 | ---NA--- | 210 |  |  |
| TRINITY_DN13696_c0_g3_i1 | ---NA--- | 263 |  |  |
| TRINITY_DN13696_c0_g4_i1 | ---NA--- | 264 |  |  |
| TRINITY_DN1369_c0_g1_i1 | F-box kelch-repeat At3g23880 | 1416 | 7.04E-45 | 45.75 |
| TRINITY_DN1369_c0_g2_i1 | F-box kelch-repeat At3g23880 | 1311 | 3.31E-45 | 45.75 |
| TRINITY_DN13719_c0_g1_i1 | DNA-directed RNA polymerase V subunit 5A | 1169 | 7.64E-112 | 83.55 |
| TRINITY_DN13721_c0_g1_i1 | hypothetical protein BVRB_5g123110 | 933 | 6.61E-31 | 58.5 |
| TRINITY_DN13725_c0_g2_i1 | ---NA--- | 628 |  |  |
| TRINITY_DN13725_c0_g3_i1 | ---NA--- | 711 |  |  |
| TRINITY_DN13731_c0_g1_i1 | CAP-Gly domain-containing linker 1-like isoform X1 | 884 | 3.62E-38 | 80.55 |
| TRINITY_DN13731_c0_g2_i1 | CAP-Gly domain-containing linker 1-like isoform X1 | 884 | 6.79E-47 | 79.95 |
| TRINITY_DN13734_c0_g1_i1 | ---NA--- | 510 |  |  |
| TRINITY_DN13743_c0_g1_i2 | vacuolar sorting-associated 51 homolog | 796 | 2.37E-147 | 89.25 |
| TRINITY_DN1374_c0_g1_i1 | thymidine kinase a-like | 878 | 3.13E-120 | 89.05 |
| TRINITY_DN13756_c0_g1_i1 | ---NA--- | 778 |  |  |
| TRINITY_DN13766_c0_g1_i1 | ---NA--- | 312 |  |  |
| TRINITY_DN13766_c0_g2_i1 | ---NA--- | 312 |  |  |
| TRINITY_DN13769_c0_g1_i1 | endonuclease or glycosyl | 2735 | 0 | 81.8 |
| TRINITY_DN13769_c0_g2_i1 | Endonuclease or glycosyl hydrolase with C2H2-type zinc finger isoform 1 | 2984 | 0 | 64 |
| TRINITY_DN13782_c0_g1_i1 | probable BOI-related E3 ubiquitin- ligase 2 | 853 | 4.04E-50 | 62.95 |
| TRINITY_DN13782_c0_g2_i1 | probable BOI-related E3 ubiquitin- ligase 2 | 301 | 4.45E-08 | 84.11 |
| TRINITY_DN13784_c0_g1_i1 | myosin heavy striated muscle | 2258 | 3.08E-123 | 51.7 |
| TRINITY_DN13788_c0_g1_i1 | S-adenosylmethionine carrier chloroplastic mitochondrial | 2404 | 4.40E-164 | 85 |
| TRINITY_DN13789_c0_g1_i1 | transcription factor PCL1-like | 1150 | 5.09E-40 | 83.85 |
| TRINITY_DN13793_c0_g1_i2 | transcription factor bHLH62 | 1468 | 1.19E-141 | 67 |
| TRINITY_DN13794_c0_g1_i1 | zinc finger family | 1902 | 0 | 84.25 |
| TRINITY_DN13794_c0_g1_i2 | zinc finger family | 1914 | 0 | 83.45 |
| TRINITY_DN13796_c0_g1_i1 | lysM domain receptor-like kinase 3 | 962 | 1.92E-20 | 82.15 |
| TRINITY_DN13797_c0_g2_i1 | ---NA--- | 341 |  |  |
| TRINITY_DN13799_c0_g2_i1 | F-box LRR-repeat At5g02910-like isoform X3 | 907 | 1.01E-70 | 50.6 |
| TRINITY_DN13805_c0_g1_i1 | yippee-like At5g53940 | 718 | 1.18E-73 | 83.25 |
| TRINITY_DN13805_c0_g2_i1 | yippee-like At5g53940 | 659 | 2.97E-72 | 85.5 |
| TRINITY_DN13807_c0_g1_i1 | ---NA--- | 545 |  |  |
| TRINITY_DN13807_c0_g2_i1 | ---NA--- | 546 |  |  |
| TRINITY_DN13817_c0_g1_i1 | uncharacterized mitochondrial g00810-like | 410 | 8.44E-51 | 71.5 |
| TRINITY_DN13817_c0_g2_i1 | uncharacterized mitochondrial g00810-like | 268 | 1.85E-26 | 70.65 |
| TRINITY_DN1381_c0_g1_i1 | hypothetical protein SOVF_093970 | 770 | 1.29E-09 | 81.33 |
| TRINITY_DN1381_c0_g2_i1 | hypothetical protein SOVF_093970 | 761 | 1.25E-09 | 81.33 |
| TRINITY_DN13820_c0_g1_i1 | DUF946 domain-containing | 1820 | 0 | 73.55 |
| TRINITY_DN13841_c0_g1_i1 | glutaredoxin family | 1329 | 7.80E-127 | 70 |
| TRINITY_DN13842_c0_g1_i1 | pentatricopeptide repeat-containing chloroplastic | 1330 | 1.05E-142 | 79.75 |
| TRINITY_DN13842_c0_g1_i2 | pentatricopeptide repeat-containing chloroplastic | 1303 | 7.64E-143 | 79.75 |
| TRINITY_DN13842_c0_g1_i3 | pentatricopeptide repeat-containing chloroplastic | 1371 | 1.73E-142 | 79.75 |
| TRINITY_DN13842_c0_g2_i1 | pentatricopeptide repeat-containing chloroplastic | 1204 | 1.75E-137 | 81.1 |
| TRINITY_DN13858_c0_g1_i1 | mediator of RNA polymerase II transcription subunit 15 isoform X2 | 3058 | 0 | 67.65 |
| TRINITY_DN13862_c0_g1_i3 | reticulon B16 | 833 | 5.40E-70 | 78.5 |
| TRINITY_DN13866_c0_g1_i1 | 9-cis-epoxycarotenoid dioxygenase chloroplastic-like | 1962 | 0 | 86.4 |
| TRINITY_DN13869_c0_g1_i1 | ---NA--- | 683 |  |  |
| TRINITY_DN13884_c0_g1_i1 | ubiquitin extension | 1998 | 3.96E-27 | 80.65 |
| TRINITY_DN13885_c0_g1_i1 | serine carboxypeptidase-like 34 | 1762 | 0 | 83.8 |
| TRINITY_DN13893_c0_g1_i1 | transcription elongation factor SPT4 homolog 2 | 879 | 3.80E-74 | 93.75 |
| TRINITY_DN13893_c1_g1_i1 | serine threonine- phosphatase 6 regulatory subunit 3 isoform X2 | 2954 | 0 | 80.4 |
| TRINITY_DN13897_c0_g1_i1 | sanguinarine reductase | 1275 | 1.22E-145 | 92.65 |
| TRINITY_DN13899_c0_g1_i1 | cleavage and polyadenylation specificity factor subunit 3-I | 2496 | 0 | 94.1 |
| TRINITY_DN13900_c0_g1_i1 | galactinol synthase 1-like | 1270 | 0 | 86 |
| TRINITY_DN13900_c0_g1_i2 | galactinol synthase 2 | 1135 | 6.94E-151 | 72.55 |
| TRINITY_DN13906_c0_g1_i1 | PREDICTED: uncharacterized protein LOC107859630 | 396 | 2.78E-08 | 51.6 |
| TRINITY_DN13906_c0_g1_i2 | probable membrane-associated kinase regulator 1 | 806 | 5.65E-49 | 58.5 |
| TRINITY_DN13906_c0_g2_i1 | ---NA--- | 349 |  |  |
| TRINITY_DN13912_c0_g1_i1 | CBL-interacting serine threonine- kinase 11 | 1790 | 0 | 77.9 |
| TRINITY_DN13916_c0_g1_i1 | xyloglucan galactosyltransferase XLT2 | 1946 | 0 | 78.9 |
| TRINITY_DN13919_c0_g1_i1 | light-inducible CPRF2 | 1652 | 1.25E-130 | 65.35 |
| TRINITY_DN13919_c0_g1_i2 | light-inducible CPRF2 | 1636 | 5.25E-168 | 67.3 |
| TRINITY_DN13925_c0_g1_i1 | splicing factor U2af small subunit B-like | 2307 | 1.04E-125 | 80.75 |
| TRINITY_DN13925_c0_g1_i2 | splicing factor U2af small subunit B-like | 2294 | 7.83E-122 | 80.75 |
| TRINITY_DN13925_c0_g2_i1 | splicing factor U2af small subunit B-like | 1218 | 1.15E-126 | 80.75 |
| TRINITY_DN13927_c0_g1_i2 | hypothetical protein SOVF_085780 | 412 | 3.55E-12 | 87 |
| TRINITY_DN13927_c0_g1_i3 | hypothetical protein SOVF_085780 | 601 | 5.51E-08 | 92 |
| TRINITY_DN13930_c0_g1_i1 | DETOXIFICATION 35 | 1963 | 0 | 81.25 |
| TRINITY_DN13933_c0_g1_i1 | membrane-bound transcription factor site-2 protease homolog isoform X2 | 722 | 1.13E-84 | 75.3 |
| TRINITY_DN13933_c0_g1_i2 | membrane-bound transcription factor site-2 protease homolog | 707 | 4.47E-79 | 73 |
| TRINITY_DN13933_c0_g1_i3 | membrane-bound transcription factor site-2 protease homolog isoform X1 | 744 | 1.40E-64 | 69.85 |
| TRINITY_DN13933_c0_g1_i4 | membrane-bound transcription factor site-2 protease homolog isoform X1 | 858 | 3.66E-57 | 55.4 |
| TRINITY_DN13933_c0_g1_i5 | membrane-bound transcription factor site-2 protease homolog | 729 | 3.48E-60 | 67.25 |
| TRINITY_DN13933_c0_g1_i6 | membrane-bound transcription factor site-2 protease homolog isoform X1 | 836 | 1.84E-76 | 63.4 |
| TRINITY_DN13934_c0_g1_i1 | ---NA--- | 218 |  |  |
| TRINITY_DN13934_c0_g2_i1 | ---NA--- | 218 |  |  |
| TRINITY_DN1394_c0_g1_i1 | kelch repeat-containing At3g27220 | 1573 | 0 | 86.5 |
| TRINITY_DN13951_c0_g1_i1 | ---NA--- | 336 |  |  |
| TRINITY_DN13951_c0_g2_i1 | ---NA--- | 347 |  |  |
| TRINITY_DN13951_c1_g1_i1 | pentatricopeptide repeat-containing At5g48910 | 291 | 6.61E-48 | 87.6 |
| TRINITY_DN13951_c1_g2_i1 | pentatricopeptide repeat-containing At5g48910 | 1216 | 3.63E-163 | 83.65 |
| TRINITY_DN13954_c0_g1_i1 | heterogeneous nuclear ribonucleo R | 1680 | 1.20E-173 | 85.4 |
| TRINITY_DN13954_c0_g1_i2 | heterogeneous nuclear ribonucleo R | 1587 | 4.00E-174 | 85.4 |
| TRINITY_DN13954_c1_g1_i1 | ---NA--- | 311 |  |  |
| TRINITY_DN13955_c0_g1_i1 | FRIGIDA 3 | 2122 | 0 | 78.25 |
| TRINITY_DN13955_c0_g1_i2 | FRIGIDA 3 | 2165 | 0 | 78.25 |
| TRINITY_DN13956_c0_g1_i1 | ---NA--- | 1012 |  |  |
| TRINITY_DN13956_c0_g2_i1 | ---NA--- | 684 |  |  |
| TRINITY_DN13957_c0_g1_i1 | ---NA--- | 467 |  |  |
| TRINITY_DN13957_c0_g2_i1 | ---NA--- | 477 |  |  |
| TRINITY_DN13962_c0_g2_i1 | PREDICTED: uncharacterized protein LOC104908470 | 1194 | 1.64E-25 | 46.38 |
| TRINITY_DN13963_c0_g1_i1 | paladin isoform X1 | 443 | 5.47E-44 | 94.3 |
| TRINITY_DN13963_c0_g1_i2 | paladin isoform X1 | 431 | 4.71E-44 | 94.3 |
| TRINITY_DN13965_c0_g1_i1 | E3 ubiquitin- ligase CIP8 | 1261 | 2.29E-81 | 67 |
| TRINITY_DN13965_c1_g1_i1 | proteasome subunit beta type-3-A | 933 | 1.71E-140 | 96.4 |
| TRINITY_DN13965_c1_g2_i1 | proteasome subunit beta type-3-A | 933 | 9.05E-140 | 95.6 |
| TRINITY_DN13967_c0_g1_i1 | traB domain-containing | 1623 | 1.07E-151 | 77.25 |
| TRINITY_DN13971_c0_g1_i1 | RING U-box superfamily isoform 2 | 1495 | 2.49E-129 | 61.75 |
| TRINITY_DN13973_c0_g1_i1 | reticulon B5 | 1282 | 6.41E-98 | 85.35 |
| TRINITY_DN13973_c0_g2_i1 | peptide chain release factor chloroplastic | 1646 | 0 | 93.85 |
| TRINITY_DN13973_c0_g2_i2 | peptide chain release factor chloroplastic | 1580 | 0 | 88.45 |
| TRINITY_DN13973_c0_g2_i3 | peptide chain release factor chloroplastic | 1798 | 0 | 93.85 |
| TRINITY_DN13973_c0_g2_i4 | peptide chain release factor chloroplastic | 1732 | 0 | 88.35 |
| TRINITY_DN13973_c0_g3_i1 | reticulon B5 | 1434 | 2.94E-97 | 85.35 |
| TRINITY_DN13982_c0_g1_i1 | ammonium transporter 3 member 1-like | 1691 | 0 | 83.75 |
| TRINITY_DN13982_c0_g2_i1 | ammonium transporter 3 member 1 | 1692 | 0 | 86.4 |
| TRINITY_DN13986_c0_g1_i2 | monosaccharide-sensing 2 | 2784 | 0 | 82.5 |
| TRINITY_DN1398_c0_g1_i1 | PREDICTED: uncharacterized protein LOC104882923 | 866 | 1.22E-24 | 61.1 |
| TRINITY_DN1398_c0_g2_i1 | PREDICTED: uncharacterized protein LOC104882923 | 865 | 1.54E-29 | 61.1 |
| TRINITY_DN14009_c0_g1_i1 | 30S ribosomal S6 chloroplastic | 907 | 3.28E-72 | 87.65 |
| TRINITY_DN14009_c0_g2_i1 | ---NA--- | 223 |  |  |
| TRINITY_DN14010_c0_g1_i2 | probable GTP-binding OBGC2 | 2017 | 0 | 81.5 |
| TRINITY_DN14012_c0_g1_i1 | plant F14N23-31 | 1830 | 0 | 78.95 |
| TRINITY_DN14012_c0_g2_i1 | plant F14N23-31 | 1830 | 0 | 78.85 |
| TRINITY_DN14012_c1_g1_i1 | hypothetical protein SOVF_147640 | 794 | 5.56E-63 | 64.4 |
| TRINITY_DN14017_c0_g2_i1 | linoleate 13S-lipoxygenase 2- chloroplastic | 3055 | 0 | 81.65 |
| TRINITY_DN14018_c0_g1_i1 | 50S ribosomal chloroplastic | 1295 | 1.37E-101 | 82.7 |
| TRINITY_DN14018_c0_g1_i2 | 50S ribosomal chloroplastic | 1310 | 1.61E-101 | 82.7 |
| TRINITY_DN14020_c0_g1_i1 | zf-BED domain-containing DUF659 domain-containing Dimer_Tnp_hAT domain-containing | 2786 | 0 | 72.95 |
| TRINITY_DN14023_c0_g1_i1 | transcription elongation factor TFIIS-like | 1549 | 4.00E-154 | 72 |
| TRINITY_DN14025_c0_g2_i1 | aldehyde dehydrogenase family 2 member C4 | 1626 | 0 | 88.05 |
| TRINITY_DN14038_c0_g1_i1 | ---NA--- | 343 |  |  |
| TRINITY_DN14038_c0_g2_i1 | ---NA--- | 343 |  |  |
| TRINITY_DN14041_c0_g1_i1 | serine threonine- kinase D6PKL1 | 2441 | 0 | 82.9 |
| TRINITY_DN14041_c0_g2_i1 | serine threonine- kinase D6PKL1 | 2441 | 0 | 83 |
| TRINITY_DN14048_c0_g1_i1 | auxin-responsive IAA16 | 607 | 4.86E-12 | 79.6 |
| TRINITY_DN14051_c0_g1_i1 | cyclic dof factor 1 | 1613 | 2.21E-95 | 54.95 |
| TRINITY_DN14055_c0_g1_i1 | CBL-interacting serine threonine- kinase 6-like | 2138 | 0 | 79.8 |
| TRINITY_DN14066_c0_g1_i1 | beta-glucuronosyltransferase 14B-like | 1746 | 0 | 89.2 |
| TRINITY_DN14068_c0_g2_i1 | NHP2 1 | 726 | 1.82E-70 | 96.25 |
| TRINITY_DN14068_c0_g2_i2 | NHP2 1 | 830 | 6.00E-70 | 96.25 |
| TRINITY_DN14072_c0_g1_i1 | trichome birefringence-like 5 | 953 | 3.06E-58 | 88.8 |
| TRINITY_DN14072_c0_g2_i1 | trichome birefringence-like 5 | 1755 | 1.50E-174 | 86 |
| TRINITY_DN14072_c0_g2_i2 | trichome birefringence-like 5 | 1738 | 0 | 87.35 |
| TRINITY_DN1407_c0_g1_i1 | ribosomal S4 (plastid) | 562 | 1.05E-06 | 92 |
| TRINITY_DN1407_c0_g2_i1 | ribosomal S4 (plastid) | 555 | 2.29E-06 | 92 |
| TRINITY_DN14092_c0_g1_i1 | AT-hook motif nuclear-localized 6 | 1524 | 1.55E-100 | 69.45 |
| TRINITY_DN14095_c0_g1_i1 | G-type lectin S-receptor-like serine threonine- kinase At1g11330 | 281 | 1.56E-12 | 63.05 |
| TRINITY_DN14096_c0_g1_i1 | reticulon B2 | 1331 | 1.20E-119 | 83.75 |
| TRINITY_DN140_c0_g1_i1 | scarecrow 15 | 687 | 6.66E-13 | 64.5 |
| TRINITY_DN14107_c0_g1_i1 | 3beta-hydroxysteroid-dehydrogenase decarboxylase | 2190 | 0 | 70.4 |
| TRINITY_DN14113_c0_g1_i1 | ---NA--- | 884 |  |  |
| TRINITY_DN14114_c0_g1_i3 | ---NA--- | 251 |  |  |
| TRINITY_DN14115_c0_g1_i1 | peroxisomal and mitochondrial division factor 2-like | 965 | 6.23E-17 | 56.95 |
| TRINITY_DN14115_c0_g2_i1 | golgin subfamily A member 6 22 | 612 | 1.13E-10 | 67.64 |
| TRINITY_DN14119_c0_g1_i1 | translation initiation factor | 2678 | 0 | 81.75 |
| TRINITY_DN14119_c0_g1_i2 | translation initiation factor | 2753 | 0 | 85.05 |
| TRINITY_DN14119_c0_g2_i1 | translation initiation factor IF-2 | 723 | 2.97E-33 | 67.8 |
| TRINITY_DN14120_c0_g1_i1 | probable phosphatase 2C 24 | 1591 | 2.32E-159 | 81.75 |
| TRINITY_DN14124_c0_g1_i1 | boron transporter 1-like | 2757 | 0 | 90.6 |
| TRINITY_DN14126_c0_g2_i1 | ---NA--- | 236 |  |  |
| TRINITY_DN14130_c0_g2_i1 | polyadenylate-binding 2 | 2653 | 0 | 89.05 |
| TRINITY_DN14132_c0_g1_i1 | PREDICTED: uncharacterized protein LOC104883752 isoform X2 | 1384 | 4.13E-102 | 52.6 |
| TRINITY_DN14134_c0_g1_i1 | crossover junction endonuclease EME1B-like | 2371 | 2.06E-150 | 77.9 |
| TRINITY_DN14134_c0_g2_i1 | crossover junction endonuclease EME1B-like | 2534 | 7.24E-154 | 77.9 |
| TRINITY_DN14141_c0_g2_i1 | FAD-linked sulfhydryl oxidase ERV1 | 788 | 1.37E-72 | 72.95 |
| TRINITY_DN14141_c0_g3_i1 | FAD-linked sulfhydryl oxidase ERV1 | 774 | 7.76E-100 | 76 |
| TRINITY_DN14144_c0_g1_i1 | 4-coumarate-- ligase-like 5 | 2247 | 0 | 82.1 |
| TRINITY_DN14152_c0_g1_i1 | subtilisin-like protease | 2795 | 0 | 80.6 |
| TRINITY_DN14152_c0_g2_i1 | ---NA--- | 371 |  |  |
| TRINITY_DN14153_c0_g1_i1 | F-box only 6 | 1820 | 0 | 83.2 |
| TRINITY_DN14154_c0_g1_i1 | ---NA--- | 323 |  |  |
| TRINITY_DN14154_c1_g1_i1 | B2 -like | 1179 | 9.37E-100 | 95.9 |
| TRINITY_DN14159_c1_g1_i1 | PREDICTED: uncharacterized protein LOC104892221 | 2299 | 2.14E-134 | 55.95 |
| TRINITY_DN14162_c0_g1_i1 | epimerase family SDR39U1 chloroplastic | 1316 | 0 | 81.45 |
| TRINITY_DN14162_c0_g1_i2 | epimerase family SDR39U1 chloroplastic | 1265 | 0 | 76.65 |
| TRINITY_DN14162_c0_g1_i3 | epimerase family SDR39U1 chloroplastic | 1376 | 0 | 85.9 |
| TRINITY_DN14165_c0_g1_i1 | BRCT domain-containing DNA repair isoform 1 | 3266 | 0 | 72.85 |
| TRINITY_DN14165_c0_g2_i1 | BRCT domain-containing DNA repair isoform 1 | 3266 | 0 | 72.85 |
| TRINITY_DN14165_c0_g3_i1 | BRCT domain-containing DNA repair isoform 1 | 1462 | 1.61E-109 | 51.95 |
| TRINITY_DN14166_c0_g1_i1 | BEL1-like homeodomain 9 | 1976 | 6.03E-162 | 58.55 |
| TRINITY_DN14166_c0_g2_i1 | BEL1-like homeodomain 9 | 1068 | 3.26E-107 | 61.15 |
| TRINITY_DN14172_c0_g1_i1 | vesicle-associated 4-2-like | 1465 | 1.91E-110 | 79.7 |
| TRINITY_DN14173_c0_g1_i1 | ---NA--- | 589 |  |  |
| TRINITY_DN14195_c0_g1_i1 | WD repeat-containing 20-like | 1894 | 0 | 85.7 |
| TRINITY_DN14195_c0_g2_i1 | WD repeat-containing 20-like | 1900 | 0 | 84.95 |
| TRINITY_DN14203_c0_g1_i1 | UPF0481 At3g47200-like | 1589 | 1.35E-145 | 57.85 |
| TRINITY_DN14203_c0_g1_i2 | UPF0481 At3g47200-like | 1712 | 1.43E-141 | 57.85 |
| TRINITY_DN14210_c0_g1_i1 | origin of replication complex subunit 3 | 801 | 1.86E-82 | 79.7 |
| TRINITY_DN14210_c0_g2_i1 | origin of replication complex subunit 3 | 1967 | 0 | 70.25 |
| TRINITY_DN14211_c0_g1_i1 | 3 -N-debenzoyl-2 -deoxytaxol N-benzoyltransferase-like | 987 | 8.72E-80 | 80.05 |
| TRINITY_DN14220_c1_g1_i1 | ENHANCER OF AG-4 2 | 915 | 1.24E-29 | 56.5 |
| TRINITY_DN14224_c0_g1_i1 | Auxin response factor 19 | 1158 | 0 | 91.35 |
| TRINITY_DN14224_c0_g1_i2 | Auxin response factor 19 | 2012 | 0 | 92.2 |
| TRINITY_DN14226_c0_g1_i1 | villin-3 isoform X1 | 3629 | 0 | 84.35 |
| TRINITY_DN14229_c0_g1_i1 | UDP-glycosyltransferase 76C4-like | 725 | 8.42E-33 | 74.15 |
| TRINITY_DN14229_c0_g1_i2 | ---NA--- | 712 |  |  |
| TRINITY_DN1422_c0_g1_i1 | PREDICTED: uncharacterized protein LOC104889075 | 1356 | 1.34E-42 | 44.7 |
| TRINITY_DN1422_c0_g2_i1 | PREDICTED: uncharacterized protein LOC104889075 | 1357 | 1.34E-42 | 44.7 |
| TRINITY_DN14238_c0_g1_i1 | 60S ribosomal L27 | 752 | 3.18E-69 | 92.9 |
| TRINITY_DN14241_c0_g1_i1 | O-acyltransferase WSD1-like | 684 | 2.87E-55 | 66.35 |
| TRINITY_DN14248_c0_g1_i1 | hypersensitive-induced response 4 | 1356 | 0 | 93.45 |
| TRINITY_DN14252_c0_g1_i1 | general negative regulator of transcription subunit 3 isoform X1 | 3232 | 0 | 74.3 |
| TRINITY_DN14252_c0_g1_i2 | CCR4-NOT transcription complex subunit 3 isoform X2 | 3259 | 0 | 74.55 |
| TRINITY_DN14252_c0_g1_i3 | CCR4-NOT transcription complex subunit 3 isoform X2 | 3238 | 0 | 73.8 |
| TRINITY_DN14252_c0_g1_i4 | general negative regulator of transcription subunit 3 isoform X1 | 3211 | 0 | 73.6 |
| TRINITY_DN14256_c0_g1_i1 | polygalacturonase 1 beta 3 | 2205 | 0 | 77.85 |
| TRINITY_DN14260_c0_g1_i1 | transcription factor HY5 | 945 | 2.54E-78 | 88.5 |
| TRINITY_DN14267_c0_g2_i1 | RNA polymerase II C-terminal domain phosphatase-like 3 | 655 | 2.11E-26 | 71.95 |
| TRINITY_DN14269_c0_g1_i1 | CW-type Zinc isoform 1 | 4783 | 0 | 70.4 |
| TRINITY_DN14273_c0_g1_i1 | importin beta-like SAD2 | 3967 | 0 | 90.6 |
| TRINITY_DN14276_c1_g1_i1 | ---NA--- | 242 |  |  |
| TRINITY_DN14279_c0_g1_i1 | hypothetical protein BVRB_7g166960 | 816 | 1.86E-07 | 59.33 |
| TRINITY_DN14289_c0_g1_i1 | plastid division PDV2 | 1418 | 2.46E-88 | 60.45 |
| TRINITY_DN14292_c0_g1_i1 | UPF0503 chloroplastic | 1964 | 1.27E-123 | 64.2 |
| TRINITY_DN14292_c0_g1_i2 | UPF0503 chloroplastic-like | 820 | 5.18E-40 | 64.6 |
| TRINITY_DN14294_c0_g1_i1 | vacuolar cation proton exchanger 3-like | 741 | 3.32E-54 | 88.4 |
| TRINITY_DN14294_c0_g1_i2 | vacuolar cation proton exchanger 3 | 1826 | 0 | 85.7 |
| TRINITY_DN14294_c0_g2_i1 | ---NA--- | 281 |  |  |
| TRINITY_DN14312_c0_g2_i1 | triacylglycerol lipase 1 | 1889 | 0 | 84.45 |
| TRINITY_DN14317_c0_g1_i1 | 4-hydroxyphenylpyruvate dioxygenase | 319 | 6.40E-16 | 76.95 |
| TRINITY_DN14317_c0_g1_i2 | 4-hydroxyphenylpyruvate dioxygenase | 755 | 1.66E-69 | 80.75 |
| TRINITY_DN14317_c0_g1_i3 | 4-hydroxyphenylpyruvate dioxygenase | 750 | 1.16E-70 | 82.2 |
| TRINITY_DN14318_c0_g1_i1 | ---NA--- | 480 |  |  |
| TRINITY_DN14318_c0_g2_i1 | ---NA--- | 487 |  |  |
| TRINITY_DN1432_c0_g1_i1 | ---NA--- | 253 |  |  |
| TRINITY_DN1432_c0_g2_i1 | ---NA--- | 238 |  |  |
| TRINITY_DN14338_c1_g1_i1 | NAC domain-containing 83 | 1162 | 2.13E-109 | 74.75 |
| TRINITY_DN14357_c0_g1_i1 | receptor-like kinase TMK4 | 2491 | 0 | 80.55 |
| TRINITY_DN14358_c0_g1_i1 | zf-BED domain-containing DUF659 domain-containing Dimer_Tnp_hAT domain-containing | 692 | 1.89E-43 | 75.1 |
| TRINITY_DN14358_c0_g1_i2 | zf-BED domain-containing DUF659 domain-containing Dimer_Tnp_hAT domain-containing | 693 | 4.07E-49 | 74.3 |
| TRINITY_DN14366_c0_g1_i1 | ---NA--- | 981 |  |  |
| TRINITY_DN14367_c0_g1_i1 | chloroplast sensor chloroplastic | 2430 | 0 | 77.25 |
| TRINITY_DN14371_c0_g1_i1 | dentin sialophospho -like | 2069 | 4.37E-87 | 52.45 |
| TRINITY_DN14384_c0_g2_i1 | outer envelope pore 16- chloroplastic | 909 | 6.97E-48 | 72.85 |
| TRINITY_DN14385_c0_g1_i1 | ribonuclease chloroplastic mitochondrial isoform X1 | 2904 | 0 | 88.4 |
| TRINITY_DN14388_c0_g1_i1 | ethylene-responsive transcription factor ERF054-like | 1641 | 2.00E-72 | 55.35 |
| TRINITY_DN14389_c0_g1_i1 | heme-binding 2 | 1515 | 4.34E-102 | 85.25 |
| TRINITY_DN14389_c0_g2_i1 | heme-binding 2 | 1782 | 1.89E-108 | 85.25 |
| TRINITY_DN14389_c0_g2_i2 | heme-binding 2 | 1815 | 2.60E-108 | 85.25 |
| TRINITY_DN14394_c0_g1_i1 | leukocyte receptor cluster member 1 | 1239 | 8.28E-49 | 63.55 |
| TRINITY_DN14405_c0_g1_i1 | PREDICTED: uncharacterized protein LOC106752479 | 864 | 1.72E-23 | 56.35 |
| TRINITY_DN14405_c0_g1_i2 | unnamed protein product | 850 | 7.61E-26 | 56.1 |
| TRINITY_DN14411_c0_g3_i1 | SPOC domain Transcription elongation factor S-II isoform 1 | 3228 | 1.54E-124 | 72.3 |
| TRINITY_DN14414_c0_g1_i1 | trichome birefringence-like 10 | 1724 | 0 | 77.5 |
| TRINITY_DN14415_c0_g1_i1 | ---NA--- | 201 |  |  |
| TRINITY_DN14415_c1_g1_i1 | formin 6 | 2019 | 0 | 88.6 |
| TRINITY_DN14416_c0_g1_i1 | E3 ubiquitin- ligase RING1-like | 1250 | 5.76E-68 | 59 |
| TRINITY_DN14417_c0_g1_i1 | oxidoreductase family | 1128 | 1.71E-114 | 66.3 |
| TRINITY_DN14425_c0_g1_i3 | heme-binding chloroplastic isoform X2 | 1178 | 5.18E-134 | 79.8 |
| TRINITY_DN14425_c0_g1_i4 | heme-binding chloroplastic isoform X2 | 1293 | 7.26E-101 | 79 |
| TRINITY_DN14434_c0_g2_i1 | ---NA--- | 373 |  |  |
| TRINITY_DN14434_c0_g2_i2 | biogenesis of lysosome-related organelles complex 1 subunit 2-like isoform X1 | 719 | 9.53E-07 | 57.5 |
| TRINITY_DN14435_c0_g1_i1 | mediator of RNA polymerase II transcription subunit 24 | 662 | 2.08E-16 | 48.65 |
| TRINITY_DN14435_c0_g1_i2 | PREDICTED: uncharacterized protein LOC104903066 | 638 | 2.92E-13 | 49.2 |
| TRINITY_DN14436_c0_g1_i1 | BPI LBP family At1g04970 | 2097 | 0 | 75.9 |
| TRINITY_DN14451_c0_g1_i1 | PREDICTED: uncharacterized protein LOC104883274 | 1003 | 8.74E-24 | 63.1 |
| TRINITY_DN14458_c0_g1_i1 | pentatricopeptide repeat-containing At5g10690 isoform X1 | 1920 | 0 | 78.1 |
| TRINITY_DN14458_c0_g2_i1 | pentatricopeptide repeat-containing At5g10690 | 2137 | 0 | 76.45 |
| TRINITY_DN14461_c0_g1_i1 | ---NA--- | 859 |  |  |
| TRINITY_DN1446_c0_g1_i1 | E3 ubiquitin- ligase UPL3-like | 302 | 7.44E-11 | 84.36 |
| TRINITY_DN14473_c0_g1_i1 | bZIP transcription factor 53-like | 1053 | 1.29E-38 | 55.6 |
| TRINITY_DN14474_c0_g1_i1 | IQ-DOMAIN 1 isoform X1 | 1511 | 1.10E-109 | 64.05 |
| TRINITY_DN14474_c0_g2_i1 | IQ-DOMAIN 1 | 1000 | 7.32E-91 | 59.9 |
| TRINITY_DN14478_c0_g1_i1 | allantoinase | 2086 | 0 | 85.2 |
| TRINITY_DN14487_c0_g1_i1 | cyclin-dependent kinase C-2-like | 1856 | 0 | 75.55 |
| TRINITY_DN14492_c0_g1_i1 | transmembrane 53-A-like | 1496 | 0 | 78.55 |
| TRINITY_DN14492_c0_g2_i1 | transmembrane 53-A-like | 1903 | 0 | 79.9 |
| TRINITY_DN14496_c0_g1_i1 | ---NA--- | 285 |  |  |
| TRINITY_DN14508_c0_g2_i1 | glutaminyl-peptide cyclotransferase | 1387 | 9.99E-142 | 76.55 |
| TRINITY_DN14514_c0_g1_i1 | NAC domain-containing 35 | 471 | 1.36E-23 | 62.45 |
| TRINITY_DN14514_c0_g2_i2 | NAC domain-containing 35 | 733 | 2.81E-123 | 82.3 |
| TRINITY_DN14514_c0_g2_i3 | NAC domain-containing 35 | 746 | 5.47E-92 | 79.5 |
| TRINITY_DN14521_c0_g1_i1 | dof zinc finger | 1520 | 4.25E-80 | 51.45 |
| TRINITY_DN14521_c0_g2_i1 | dof zinc finger -like | 442 | 4.93E-32 | 95.1 |
| TRINITY_DN14522_c0_g1_i1 | serine-rich adhesin for platelets-like | 1062 | 1.31E-34 | 53.5 |
| TRINITY_DN1452_c0_g1_i1 | probable membrane-associated kinase regulator 1 | 734 | 2.59E-22 | 64.55 |
| TRINITY_DN1452_c0_g2_i1 | probable membrane-associated kinase regulator 1 | 735 | 1.41E-21 | 64.25 |
| TRINITY_DN14530_c0_g1_i1 | geranylgeranyl pyrophosphate chloroplastic | 1465 | 1.99E-154 | 87.45 |
| TRINITY_DN14533_c0_g1_i1 | PREDICTED: uncharacterized protein LOC104902408 | 1386 | 4.61E-09 | 53.75 |
| TRINITY_DN14535_c0_g1_i1 | 28 kDa chloroplastic | 1549 | 4.03E-80 | 75.25 |
| TRINITY_DN14536_c0_g1_i2 | Transport SEC31 | 1767 | 3.97E-58 | 68.9 |
| TRINITY_DN14552_c0_g2_i1 | ethylene-responsive transcription factor ERF011-like | 1053 | 1.04E-63 | 77.85 |
| TRINITY_DN14554_c0_g2_i1 | BRO1 domain-containing BROX | 1771 | 0 | 89.6 |
| TRINITY_DN14562_c0_g1_i1 | type-2 histone deacetylase 1 | 1653 | 4.98E-13 | 61.5 |
| TRINITY_DN14565_c0_g1_i1 | adenine phosphoribosyltransferase 5 | 975 | 1.06E-123 | 91.95 |
| TRINITY_DN14565_c0_g2_i1 | adenine phosphoribosyltransferase 5 | 864 | 3.01E-86 | 75.55 |
| TRINITY_DN14566_c0_g1_i1 | RING-H2 finger ATL56-like | 1096 | 3.38E-51 | 57.45 |
| TRINITY_DN14567_c0_g1_i1 | DNA-directed RNA polymerase III subunit RPC5 isoform X1 | 2454 | 0 | 61.25 |
| TRINITY_DN14577_c0_g1_i1 | pleckstrin homology domain-containing 1 | 943 | 8.15E-89 | 87.95 |
| TRINITY_DN14577_c0_g2_i1 | pleckstrin homology domain-containing 1 | 1278 | 3.30E-87 | 87.95 |
| TRINITY_DN14587_c0_g1_i1 | ---NA--- | 338 |  |  |
| TRINITY_DN14587_c0_g2_i3 | Proline-rich receptor kinase PERK1 | 1674 | 0 | 91.4 |
| TRINITY_DN14592_c0_g1_i1 | hypothetical protein SOVF_036200 | 1299 | 5.16E-64 | 85.55 |
| TRINITY_DN14592_c0_g1_i2 | hypothetical protein SOVF_036200 | 1264 | 3.73E-64 | 85.55 |
| TRINITY_DN14601_c0_g1_i1 | translocase subunit chloroplastic isoform X2 | 2682 | 0 | 59.6 |
| TRINITY_DN14601_c0_g2_i1 | translocase subunit chloroplastic isoform X2 | 2980 | 0 | 59.9 |
| TRINITY_DN14605_c0_g1_i1 | probable Histone-lysine N-methyltransferase ATXR5 | 667 | 2.04E-46 | 75.85 |
| TRINITY_DN14620_c0_g1_i1 | kinase PINOID 2 | 878 | 1.24E-136 | 82.45 |
| TRINITY_DN14620_c0_g2_i1 | kinase PINOID 2 | 228 | 8.70E-28 | 97.6 |
| TRINITY_DN14621_c0_g1_i1 | ---NA--- | 373 |  |  |
| TRINITY_DN14621_c0_g2_i1 | ---NA--- | 310 |  |  |
| TRINITY_DN14626_c0_g1_i1 | ---NA--- | 586 |  |  |
| TRINITY_DN14626_c0_g1_i2 | ---NA--- | 563 |  |  |
| TRINITY_DN14641_c0_g1_i1 | IAA-amino acid hydrolase ILR1-like 3 | 1715 | 0 | 78.2 |
| TRINITY_DN14641_c0_g2_i1 | IAA-amino acid hydrolase ILR1-like 3 | 1715 | 0 | 78.2 |
| TRINITY_DN14641_c0_g3_i1 | IAA-amino acid hydrolase ILR1-like 3 | 1674 | 0 | 78.2 |
| TRINITY_DN14652_c0_g1_i1 | probable receptor kinase At2g42960 | 2396 | 0 | 87.9 |
| TRINITY_DN14653_c2_g1_i1 | ---NA--- | 235 |  |  |
| TRINITY_DN14653_c2_g2_i1 | ---NA--- | 343 |  |  |
| TRINITY_DN14659_c0_g1_i1 | probable arabinosyltransferase ARAD1 | 1780 | 0 | 84.9 |
| TRINITY_DN14661_c0_g1_i1 | target of Myb 1 | 2231 | 3.73E-134 | 80.5 |
| TRINITY_DN14665_c0_g1_i1 | PREDICTED: uncharacterized protein LOC104900040 | 1571 | 1.36E-111 | 71 |
| TRINITY_DN14665_c0_g1_i2 | PREDICTED: uncharacterized protein LOC104900040 | 1519 | 9.92E-112 | 71 |
| TRINITY_DN14665_c0_g2_i1 | ---NA--- | 448 |  |  |
| TRINITY_DN14669_c0_g1_i1 | mediator of RNA polymerase II transcription subunit 15a isoform X1 | 1475 | 1.72E-30 | 77.45 |
| TRINITY_DN14673_c0_g1_i1 | ---NA--- | 446 |  |  |
| TRINITY_DN14673_c0_g2_i1 | ---NA--- | 820 |  |  |
| TRINITY_DN14677_c0_g1_i1 | aldehyde dehydrogenase family 3 member H1 isoform X2 | 1924 | 0 | 85.8 |
| TRINITY_DN14677_c0_g1_i2 | aldehyde dehydrogenase family 3 member H1 isoform X2 | 1935 | 0 | 83.45 |
| TRINITY_DN14688_c0_g1_i2 | ---NA--- | 315 |  |  |
| TRINITY_DN14688_c0_g2_i1 | ---NA--- | 331 |  |  |
| TRINITY_DN14690_c0_g1_i1 | ---NA--- | 248 |  |  |
| TRINITY_DN1470_c0_g1_i1 | disease resistance RGA3 | 2891 | 0 | 63 |
| TRINITY_DN14718_c0_g1_i1 | transmembrane 120 homolog | 1118 | 2.43E-127 | 86.85 |
| TRINITY_DN14718_c0_g2_i1 | transmembrane 120 homolog isoform X2 | 891 | 3.15E-58 | 83.8 |
| TRINITY_DN1471_c0_g1_i1 | photosystem II phospho (chloroplast) | 704 | 3.52E-45 | 97.4 |
| TRINITY_DN14725_c0_g2_i1 | Actin-depolymerizing factor 2 | 272 | 3.36E-22 | 87.8 |
| TRINITY_DN14725_c0_g3_i2 | Actin-depolymerizing factor 2 | 361 | 1.95E-21 | 88.85 |
| TRINITY_DN14729_c0_g1_i1 | ---NA--- | 429 |  |  |
| TRINITY_DN14729_c0_g1_i2 | ---NA--- | 988 |  |  |
| TRINITY_DN14729_c0_g1_i3 | ---NA--- | 204 |  |  |
| TRINITY_DN14733_c0_g1_i1 | 3-oxoacyl-[acyl-carrier- ] reductase | 603 | 1.97E-64 | 77.25 |
| TRINITY_DN14733_c0_g1_i2 | 3-oxoacyl-[acyl-carrier- ] reductase | 1138 | 5.85E-132 | 81.4 |
| TRINITY_DN14734_c0_g1_i1 | ---NA--- | 375 |  |  |
| TRINITY_DN14734_c0_g1_i2 | ---NA--- | 389 |  |  |
| TRINITY_DN14734_c1_g1_i1 | Transmembrane like | 1735 | 0 | 87.25 |
| TRINITY_DN14734_c1_g2_i1 | Transmembrane like | 1720 | 0 | 86.63 |
| TRINITY_DN14735_c0_g1_i1 | basic proline-rich | 1343 | 1.77E-34 | 75.8 |
| TRINITY_DN14735_c0_g1_i2 | formin 5 | 1920 | 2.83E-65 | 92.79 |
| TRINITY_DN14743_c0_g1_i1 | D-3-phosphoglycerate dehydrogenase chloroplastic-like | 2256 | 0 | 94 |
| TRINITY_DN14746_c0_g1_i1 | probable phosphatase 2C 49 | 2574 | 7.03E-137 | 85.95 |
| TRINITY_DN14746_c0_g2_i1 | probable phosphatase 2C 49 | 2526 | 4.59E-137 | 85.95 |
| TRINITY_DN14752_c0_g1_i2 | NRT1 PTR FAMILY isoform X1 | 2038 | 0 | 85.25 |
| TRINITY_DN14754_c0_g2_i1 | starmaker | 2845 | 4.77E-55 | 74.45 |
| TRINITY_DN14756_c0_g1_i1 | ---NA--- | 316 |  |  |
| TRINITY_DN14756_c0_g2_i1 | FT-interacting 1-like | 2483 | 0 | 85.3 |
| TRINITY_DN14759_c0_g1_i1 | regulation of nuclear pre-mRNA domain-containing 1B | 1281 | 3.35E-180 | 79.8 |
| TRINITY_DN14759_c1_g1_i1 | ---NA--- | 669 |  |  |
| TRINITY_DN14761_c0_g1_i1 | probable 1-deoxy-D-xylulose-5-phosphate chloroplastic | 2388 | 0 | 91.9 |
| TRINITY_DN14767_c0_g1_i1 | ---NA--- | 283 |  |  |
| TRINITY_DN14773_c0_g1_i1 | casein kinase I-like isoform X1 | 2743 | 0 | 85.3 |
| TRINITY_DN14773_c0_g1_i2 | casein kinase I-like isoform X1 | 2731 | 0 | 85.3 |
| TRINITY_DN14773_c0_g2_i1 | casein kinase I-like isoform X1 | 2575 | 0 | 85.3 |
| TRINITY_DN14774_c0_g1_i1 | ---NA--- | 805 |  |  |
| TRINITY_DN14774_c0_g1_i2 | ---NA--- | 972 |  |  |
| TRINITY_DN14774_c0_g1_i3 | ---NA--- | 896 |  |  |
| TRINITY_DN14790_c0_g1_i1 | root UVB sensitive 3 | 1039 | 1.81E-43 | 77.3 |
| TRINITY_DN14793_c0_g1_i1 | S-adenosyl-L-methionine-dependent methyltransferases superfamily | 1546 | 5.27E-151 | 74.1 |
| TRINITY_DN14795_c0_g1_i2 | PREDICTED: uncharacterized protein At5g41620 | 1892 | 0 | 59.2 |
| TRINITY_DN14796_c0_g1_i1 | ---NA--- | 1153 |  |  |
| TRINITY_DN14798_c0_g1_i1 | NBR1 homolog | 1217 | 6.82E-25 | 66.4 |
| TRINITY_DN14804_c0_g1_i1 | pentatricopeptide repeat-containing mitochondrial-like | 2422 | 0 | 79.8 |
| TRINITY_DN14814_c1_g1_i1 | 60S ribosomal L13a-2-like | 616 | 8.54E-111 | 96.35 |
| TRINITY_DN14814_c1_g1_i2 | 60S ribosomal L13a-4 | 952 | 9.68E-128 | 95.3 |
| TRINITY_DN14818_c0_g1_i1 | ATP-dependent Clp protease proteolytic subunit mitochondrial | 1138 | 1.15E-127 | 95.65 |
| TRINITY_DN14819_c0_g1_i1 | F-box SKIP28 | 1282 | 7.50E-71 | 60.5 |
| TRINITY_DN1481_c0_g1_i1 | IRK-interacting isoform X2 | 1423 | 1.20E-165 | 80.95 |
| TRINITY_DN14823_c0_g1_i1 | phosphate chloroplastic | 3219 | 0 | 90.55 |
| TRINITY_DN14823_c0_g1_i2 | phosphate chloroplastic | 3113 | 0 | 90.55 |
| TRINITY_DN14831_c0_g1_i1 | CBL-interacting serine threonine- kinase 23 | 2579 | 0 | 92.15 |
| TRINITY_DN14831_c0_g1_i2 | CBL-interacting serine threonine- kinase 23 | 2580 | 0 | 91.2 |
| TRINITY_DN14831_c0_g1_i3 | CBL-interacting serine threonine- kinase 23 | 1940 | 0 | 92.15 |
| TRINITY_DN14831_c0_g1_i4 | CBL-interacting serine threonine- kinase 23 | 1941 | 0 | 91.2 |
| TRINITY_DN14839_c0_g1_i1 | CDPK-related kinase 3 | 1119 | 5.22E-102 | 76.9 |
| TRINITY_DN14839_c0_g2_i1 | CDPK-related kinase 3 | 2378 | 0 | 84.85 |
| TRINITY_DN14847_c1_g1_i1 | strawberry notch | 4175 | 0 | 86 |
| TRINITY_DN14847_c2_g1_i1 | 50S ribosomal chloroplastic | 901 | 1.25E-80 | 90.95 |
| TRINITY_DN14855_c0_g1_i3 | ---NA--- | 442 |  |  |
| TRINITY_DN14862_c0_g1_i1 | auxin response factor 18-like | 1762 | 0 | 85.4 |
| TRINITY_DN14862_c0_g1_i2 | auxin response factor 18-like | 612 | 2.56E-111 | 87.7 |
| TRINITY_DN14865_c0_g1_i1 | Sn1-specific diacylglycerol lipase alpha | 2535 | 0 | 81.95 |
| TRINITY_DN1486_c0_g1_i1 | ---NA--- | 252 |  |  |
| TRINITY_DN14875_c0_g1_i1 | proline--tRNA chloroplastic mitochondrial | 2096 | 0 | 92.05 |
| TRINITY_DN14882_c0_g1_i1 | exocyst complex component EXO70A1-like | 2440 | 0 | 88.3 |
| TRINITY_DN14885_c0_g1_i1 | farnesyltransferase geranylgeranyltransferase type-1 subunit alpha | 1260 | 0 | 81.75 |
| TRINITY_DN14885_c0_g1_i2 | farnesyltransferase geranylgeranyltransferase type-1 subunit alpha | 1271 | 3.31E-149 | 81.4 |
| TRINITY_DN14886_c0_g2_i1 | PREDICTED: uncharacterized protein LOC104901901 | 1816 | 0 | 80.9 |
| TRINITY_DN14887_c0_g1_i1 | E3 ubiquitin- ligase MBR2 isoform X2 | 1233 | 8.73E-119 | 64.75 |
| TRINITY_DN14888_c0_g3_i1 | B3 domain-containing Os01g0234100-like isoform X2 | 535 | 1.85E-20 | 68.75 |
| TRINITY_DN14906_c0_g1_i1 | ---NA--- | 1103 |  |  |
| TRINITY_DN14906_c0_g1_i2 | ---NA--- | 1087 |  |  |
| TRINITY_DN14912_c0_g1_i1 | staphylococcal-like nuclease CAN2 | 1511 | 0 | 86.65 |
| TRINITY_DN14915_c0_g1_i1 | epidermal growth factor receptor substrate 15-like 1 | 4117 | 0 | 61.35 |
| TRINITY_DN14918_c0_g1_i1 | myb family transcription factor family | 1089 | 5.13E-111 | 76.35 |
| TRINITY_DN14919_c0_g1_i1 | trihelix transcription factor ASIL2 | 1272 | 1.59E-45 | 54.7 |
| TRINITY_DN14919_c0_g1_i2 | trihelix transcription factor ASIL2 | 1258 | 1.59E-45 | 54.95 |
| TRINITY_DN14919_c0_g1_i3 | trihelix transcription factor ASIL2 | 1088 | 4.90E-46 | 54.95 |
| TRINITY_DN14922_c0_g1_i1 | ATP synthase delta chloroplastic | 927 | 6.16E-26 | 73.05 |
| TRINITY_DN14922_c0_g2_i1 | ATP synthase delta chloroplastic | 691 | 5.51E-27 | 72.9 |
| TRINITY_DN14922_c0_g3_i1 | ATP synthase delta chloroplastic | 905 | 8.47E-24 | 73.25 |
| TRINITY_DN14922_c0_g4_i1 | ATP synthase delta chloroplastic | 905 | 9.21E-24 | 73 |
| TRINITY_DN14922_c0_g5_i1 | ---NA--- | 446 |  |  |
| TRINITY_DN14922_c0_g6_i1 | ATP synthase delta chloroplastic | 691 | 1.05E-26 | 73.2 |
| TRINITY_DN14922_c0_g7_i1 | ATP synthase delta chloroplastic | 927 | 3.29E-26 | 72.05 |
| TRINITY_DN14930_c0_g1_i1 | probable sulfate transporter | 2630 | 0 | 84.4 |
| TRINITY_DN14933_c0_g1_i1 | probable sodium-coupled neutral amino acid transporter 6 | 2133 | 0 | 89.55 |
| TRINITY_DN14933_c0_g2_i1 | probable sodium-coupled neutral amino acid transporter 6 | 2133 | 0 | 89.9 |
| TRINITY_DN14944_c0_g2_i1 | Plasma membrane isoform 1 | 1988 | 0 | 67.75 |
| TRINITY_DN14947_c0_g1_i1 | Beta-galactosidase 9 isoform 1 | 1796 | 0 | 85.05 |
| TRINITY_DN14949_c0_g2_i1 | ribonuclease 1 | 1533 | 1.79E-125 | 72.5 |
| TRINITY_DN14949_c1_g1_i1 | scarecrow 14 | 429 | 3.58E-17 | 69.63 |
| TRINITY_DN14962_c0_g1_i1 | GPI ethanolamine phosphate transferase 1 | 3158 | 0 | 85.8 |
| TRINITY_DN14963_c0_g1_i2 | CASP 4A3 | 1108 | 1.15E-80 | 83.7 |
| TRINITY_DN14966_c1_g1_i1 | ---NA--- | 214 |  |  |
| TRINITY_DN14969_c0_g1_i1 | serine arginine-rich splicing factor RS40 isoform X1 | 1512 | 6.71E-133 | 68.8 |
| TRINITY_DN14969_c0_g2_i1 | serine arginine-rich splicing factor RS40 isoform X1 | 398 | 5.75E-15 | 77.9 |
| TRINITY_DN14975_c0_g1_i1 | GPI transamidase component PIG-T | 2206 | 0 | 75.7 |
| TRINITY_DN14976_c0_g1_i1 | PREDICTED: uncharacterized protein LOC104888549 | 792 | 6.18E-43 | 95.35 |
| TRINITY_DN14979_c0_g1_i1 | ---NA--- | 630 |  |  |
| TRINITY_DN14979_c0_g1_i2 | ---NA--- | 700 |  |  |
| TRINITY_DN14983_c0_g1_i1 | MRG1 isoform X2 | 1333 | 0 | 85.4 |
| TRINITY_DN14983_c0_g1_i2 | MRG1 isoform X2 | 1369 | 1.60E-177 | 82 |
| TRINITY_DN14992_c0_g1_i1 | CBL-interacting serine threonine- kinase 1 | 1683 | 0 | 88.1 |
| TRINITY_DN14992_c0_g2_i1 | CBL-interacting serine threonine- kinase 1 | 947 | 5.37E-123 | 83.8 |
| TRINITY_DN14994_c0_g1_i1 | transcription termination factor chloroplastic | 2374 | 0 | 80.9 |
| TRINITY_DN14994_c0_g1_i2 | transcription termination factor chloroplastic | 2043 | 0 | 80.9 |
| TRINITY_DN15008_c0_g1_i1 | zinc finger 4-like | 822 | 3.18E-50 | 52.85 |
| TRINITY_DN15008_c0_g1_i2 | zinc finger 4-like | 923 | 2.39E-50 | 51.5 |
| TRINITY_DN15026_c0_g1_i1 | acidic leucine-rich nuclear phospho 32-related | 664 | 1.69E-76 | 91.3 |
| TRINITY_DN15026_c1_g1_i1 | E3 ubiquitin- ligase SINAT5 | 1629 | 0 | 89.1 |
| TRINITY_DN15035_c0_g1_i1 | plant cysteine oxidase 4 | 1087 | 1.29E-138 | 84 |
| TRINITY_DN15035_c1_g1_i1 | ---NA--- | 220 |  |  |
| TRINITY_DN15038_c0_g1_i1 | ---NA--- | 524 |  |  |
| TRINITY_DN15038_c0_g3_i1 | ---NA--- | 308 |  |  |
| TRINITY_DN15057_c0_g1_i1 | ---NA--- | 265 |  |  |
| TRINITY_DN15057_c0_g2_i1 | ---NA--- | 268 |  |  |
| TRINITY_DN15057_c1_g1_i1 | transcription factor TCP2 | 422 | 2.42E-24 | 93.85 |
| TRINITY_DN15057_c1_g2_i1 | transcription factor TCP2 | 1508 | 9.70E-43 | 89.9 |
| TRINITY_DN15057_c1_g2_i2 | transcription factor TCP2 | 1497 | 8.99E-43 | 89.9 |
| TRINITY_DN15060_c0_g1_i1 | scarecrow 1 | 2012 | 0 | 86.45 |
| TRINITY_DN15060_c0_g1_i2 | scarecrow 1 | 2094 | 0 | 77.1 |
| TRINITY_DN15065_c0_g1_i1 | C2-DOMAIN ABA-RELATED 11 | 817 | 4.50E-84 | 86.15 |
| TRINITY_DN15067_c0_g1_i1 | ROTUNDIFOLIA like 2 | 346 | 6.48E-17 | 93 |
| TRINITY_DN15067_c0_g2_i1 | ROTUNDIFOLIA like 2 | 537 | 2.54E-16 | 93 |
| TRINITY_DN15068_c0_g1_i1 | ribulose-1,5 bisphosphate carboxylase oxygenase large subunit N- chloroplastic | 1953 | 0 | 85.8 |
| TRINITY_DN15068_c0_g2_i1 | ribulose-1,5 bisphosphate carboxylase oxygenase large subunit N- chloroplastic | 1952 | 0 | 85.8 |
| TRINITY_DN15068_c1_g1_i1 | hypothetical protein SOVF_154000 | 1326 | 0 | 85.15 |
| TRINITY_DN15068_c2_g1_i1 | ---NA--- | 479 |  |  |
| TRINITY_DN1506_c0_g1_i1 | F-box kelch-repeat At1g57790-like | 1559 | 0 | 85.75 |
| TRINITY_DN15072_c0_g1_i1 | serine threonine- phosphatase 2A 65 kDa regulatory subunit A beta isoform-like | 2140 | 0 | 96.1 |
| TRINITY_DN15073_c0_g1_i1 | heat shock binding | 1209 | 2.33E-101 | 66.1 |
| TRINITY_DN15078_c0_g2_i1 | ---NA--- | 551 |  |  |
| TRINITY_DN15079_c0_g1_i1 | RTF1 homolog | 2525 | 0 | 76 |
| TRINITY_DN15080_c0_g1_i1 | small RNA degrading nuclease 5 | 1979 | 0 | 81.45 |
| TRINITY_DN15086_c0_g1_i1 | transcriptional corepressor SEUSS-like | 878 | 2.23E-17 | 68.85 |
| TRINITY_DN15088_c0_g2_i1 | ---NA--- | 409 |  |  |
| TRINITY_DN15097_c0_g1_i1 | zf-LITAF-like domain-containing | 754 | 3.42E-56 | 74.6 |
| TRINITY_DN15097_c1_g1_i1 | lactoylglutathione lyase isoform X1 | 1195 | 3.04E-113 | 93.3 |
| TRINITY_DN15097_c1_g2_i1 | lactoylglutathione lyase isoform X1 | 1174 | 2.88E-113 | 93.3 |
| TRINITY_DN15097_c1_g3_i1 | lactoylglutathione lyase isoform X1 | 1186 | 7.77E-117 | 93.3 |
| TRINITY_DN15097_c1_g4_i1 | indeterminate-domain 7 | 285 | 1.25E-06 | 63 |
| TRINITY_DN15100_c0_g1_i1 | F-box FBD LRR-repeat At4g26340-like | 914 | 3.65E-21 | 52.45 |
| TRINITY_DN15100_c0_g1_i2 | F-box FBD LRR-repeat At4g26340-like | 1002 | 6.36E-21 | 52.45 |
| TRINITY_DN15105_c0_g1_i1 | ---NA--- | 392 |  |  |
| TRINITY_DN15106_c0_g1_i1 | #NAME? | 2070 | 0 | 77.2 |
| TRINITY_DN15106_c0_g1_i2 | #NAME? | 2084 | 0 | 78.3 |
| TRINITY_DN15106_c0_g2_i1 | #NAME? | 922 | 6.53E-112 | 87.9 |
| TRINITY_DN15115_c0_g1_i1 | mitogen-activated kinase homolog NTF6 | 1606 | 0 | 91.5 |
| TRINITY_DN15119_c0_g1_i1 | thioredoxin F- chloroplastic | 995 | 7.52E-62 | 83.4 |
| TRINITY_DN15126_c0_g1_i1 | homogentisate phytyltransferase chloroplastic isoform X1 | 738 | 2.44E-80 | 85.8 |
| TRINITY_DN15126_c0_g1_i2 | homogentisate phytyltransferase chloroplastic isoform X1 | 1562 | 0 | 83.8 |
| TRINITY_DN15130_c0_g1_i1 | calcium uptake mitochondrial | 1929 | 0 | 82.9 |
| TRINITY_DN15130_c0_g1_i2 | calcium uptake mitochondrial | 2069 | 0 | 87.32 |
| TRINITY_DN15130_c0_g1_i3 | calcium uptake mitochondrial | 2175 | 0 | 87.3 |
| TRINITY_DN15131_c0_g1_i1 | F-box At5g46170-like | 781 | 1.01E-95 | 73.75 |
| TRINITY_DN15137_c0_g1_i1 | PREDICTED: uncharacterized protein LOC104898146 | 853 | 2.59E-84 | 74.9 |
| TRINITY_DN15143_c0_g1_i1 | hypothetical protein SOVF_189540 | 1519 | 2.49E-167 | 67 |
| TRINITY_DN15143_c0_g1_i4 | hypothetical protein SOVF_189540 | 1502 | 8.08E-144 | 66.55 |
| TRINITY_DN15143_c0_g2_i1 | hypothetical protein SOVF_189540 | 557 | 1.22E-53 | 76.2 |
| TRINITY_DN15165_c0_g1_i1 | PREDICTED: uncharacterized protein LOC104901896 | 537 | 1.06E-31 | 60.53 |
| TRINITY_DN15167_c0_g1_i1 | probable phosphatase 2C 5 | 463 | 1.60E-64 | 91.3 |
| TRINITY_DN15167_c0_g2_i1 | probable phosphatase 2C 5 | 1677 | 0 | 91.15 |
| TRINITY_DN15167_c0_g3_i1 | probable phosphatase 2C 5 | 520 | 1.90E-76 | 92.05 |
| TRINITY_DN15179_c0_g2_i1 | actin cytoskeleton-regulatory complex pan1 | 1571 | 2.10E-158 | 76.35 |
| TRINITY_DN15179_c0_g2_i2 | actin cytoskeleton-regulatory complex pan1 | 1860 | 5.66E-161 | 76.35 |
| TRINITY_DN15184_c0_g1_i1 | ---NA--- | 228 |  |  |
| TRINITY_DN15189_c0_g1_i1 | ATP-dependent Clp protease proteolytic subunit chloroplastic | 1450 | 6.73E-173 | 84.6 |
| TRINITY_DN15206_c0_g1_i1 | gag and pol identical | 617 | 3.60E-81 | 69 |
| TRINITY_DN15216_c0_g1_i1 | PAF1 homolog | 2398 | 0 | 77.95 |
| TRINITY_DN15217_c0_g1_i1 | LOW PSII ACCUMULATION chloroplastic | 1383 | 0 | 90.05 |
| TRINITY_DN15217_c0_g1_i2 | LOW PSII ACCUMULATION chloroplastic | 1181 | 0 | 89.85 |
| TRINITY_DN15218_c0_g3_i2 | ---NA--- | 490 |  |  |
| TRINITY_DN15233_c0_g1_i1 | homeotic female sterile-like | 958 | 7.38E-75 | 49.3 |
| TRINITY_DN15233_c0_g2_i1 | homeotic female sterile-like | 954 | 2.05E-76 | 49.7 |
| TRINITY_DN15233_c1_g1_i2 | methyl- -binding domain-containing 2 | 1745 | 5.38E-119 | 72.05 |
| TRINITY_DN15233_c1_g2_i1 | ---NA--- | 374 |  |  |
| TRINITY_DN15257_c0_g1_i1 | thylakoid lumenal kDa chloroplastic | 1043 | 6.93E-61 | 73.25 |
| TRINITY_DN1525_c0_g1_i1 | B2 | 1467 | 6.17E-154 | 84.55 |
| TRINITY_DN15269_c0_g1_i1 | peter Pan | 1157 | 1.83E-172 | 87.95 |
| TRINITY_DN15269_c1_g1_i1 | ---NA--- | 310 |  |  |
| TRINITY_DN15275_c0_g1_i1 | probable aquaporin TIP2-2 | 1012 | 2.80E-130 | 92.1 |
| TRINITY_DN15278_c0_g1_i1 | CBL-interacting serine threonine- kinase 6-like | 959 | 1.07E-86 | 72.55 |
| TRINITY_DN15293_c0_g1_i1 | serine threonine- phosphatase PP-Z1 | 666 | 3.33E-25 | 52.9 |
| TRINITY_DN15293_c0_g1_i2 | serine threonine- phosphatase PP-Z1 | 650 | 2.15E-22 | 52.35 |
| TRINITY_DN15293_c0_g1_i3 | serine threonine- phosphatase PP-Z1 | 921 | 7.69E-37 | 55.35 |
| TRINITY_DN15299_c0_g1_i1 | ---NA--- | 1213 |  |  |
| TRINITY_DN1529_c0_g1_i1 | PREDICTED: uncharacterized protein LOC104902996 | 619 | 2.61E-41 | 67.25 |
| TRINITY_DN1529_c0_g2_i1 | PREDICTED: uncharacterized protein LOC104902996 | 619 | 6.49E-38 | 67.15 |
| TRINITY_DN15303_c0_g1_i1 | ---NA--- | 207 |  |  |
| TRINITY_DN15313_c0_g1_i1 | ---NA--- | 529 |  |  |
| TRINITY_DN15319_c0_g1_i1 | shugoshin-1 isoform X2 | 708 | 9.16E-26 | 60.35 |
| TRINITY_DN1531_c0_g1_i1 | ---NA--- | 248 |  |  |
| TRINITY_DN15324_c0_g1_i1 | DUF581 domain-containing | 820 | 2.29E-46 | 74.85 |
| TRINITY_DN15332_c0_g1_i2 | ---NA--- | 429 |  |  |
| TRINITY_DN15337_c0_g1_i1 | histone-lysine N-methyltransferase ASHH1 isoform X1 | 1815 | 0 | 69.55 |
| TRINITY_DN15337_c0_g2_i1 | histone-lysine N-methyltransferase ASHH1 isoform X1 | 1829 | 0 | 73.8 |
| TRINITY_DN15345_c0_g2_i1 | fructose-1,6- chloroplastic | 1842 | 0 | 86.6 |
| TRINITY_DN15349_c0_g1_i1 | RETICULATA-RELATED chloroplastic-like | 741 | 2.41E-65 | 84.65 |
| TRINITY_DN15350_c0_g1_i1 | la-related 1-like | 1393 | 2.74E-124 | 72.15 |
| TRINITY_DN15357_c0_g1_i1 | PHD finger ALFIN-LIKE 3-like isoform X2 | 1241 | 9.87E-120 | 91.5 |
| TRINITY_DN15358_c0_g1_i1 | chalcone synthase | 1301 | 1.28E-157 | 77.4 |
| TRINITY_DN1535_c0_g1_i1 | 1-aminocyclopropane-1-carboxylate oxidase homolog 4-like | 1041 | 1.98E-103 | 66 |
| TRINITY_DN15360_c0_g1_i1 | receptor-like serine threonine- kinase ALE2 | 1847 | 0 | 79.4 |
| TRINITY_DN15360_c0_g1_i2 | receptor-like serine threonine- kinase ALE2 | 1698 | 0 | 80.05 |
| TRINITY_DN15364_c0_g1_i1 | germin subfamily 2 member 4 | 956 | 8.07E-121 | 88.05 |
| TRINITY_DN15371_c0_g1_i1 | ---NA--- | 424 |  |  |
| TRINITY_DN15371_c0_g2_i1 | ---NA--- | 510 |  |  |
| TRINITY_DN15373_c0_g2_i1 | ---NA--- | 637 |  |  |
| TRINITY_DN15377_c0_g1_i1 | ---NA--- | 617 |  |  |
| TRINITY_DN15383_c0_g1_i1 | ---NA--- | 341 |  |  |
| TRINITY_DN15387_c0_g1_i1 | reticulon-4-interacting mitochondrial | 1562 | 0 | 86.8 |
| TRINITY_DN15395_c0_g1_i1 | ---NA--- | 571 |  |  |
| TRINITY_DN15395_c0_g1_i2 | ---NA--- | 550 |  |  |
| TRINITY_DN15401_c0_g1_i1 | acyl- -binding domain-containing 4 | 2437 | 0 | 80.75 |
| TRINITY_DN15402_c0_g1_i1 | high chlorophyll fluorescent 107 | 2030 | 0 | 82.2 |
| TRINITY_DN15406_c0_g1_i1 | cell wall RBR3 | 2269 | 3.43E-117 | 82.1 |
| TRINITY_DN15406_c0_g2_i1 | mucin-5AC-like isoform X1 | 918 | 2.31E-39 | 62.4 |
| TRINITY_DN15407_c0_g1_i1 | SAC3 family C isoform X1 | 1632 | 7.76E-133 | 70.65 |
| TRINITY_DN15407_c0_g1_i2 | SAC3 family C isoform X1 | 1695 | 7.49E-157 | 77.85 |
| TRINITY_DN15407_c0_g1_i3 | SAC3 family C isoform X1 | 1725 | 1.05E-156 | 77.85 |
| TRINITY_DN15407_c0_g1_i4 | SAC3 family C isoform X1 | 1602 | 5.58E-133 | 70.65 |
| TRINITY_DN15409_c0_g1_i1 | LRR receptor-like serine threonine- kinase EFR | 1486 | 3.91E-101 | 77.4 |
| TRINITY_DN15442_c0_g1_i1 | Agenet domain-containing | 1320 | 1.23E-70 | 59.3 |
| TRINITY_DN15442_c0_g1_i2 | Agenet domain-containing | 1221 | 1.92E-71 | 59.3 |
| TRINITY_DN15446_c0_g1_i1 | ---NA--- | 308 |  |  |
| TRINITY_DN15449_c0_g1_i1 | synaptotagmin-3 isoform X1 | 2245 | 0 | 80.5 |
| TRINITY_DN15460_c0_g1_i1 | F-box kelch-repeat At3g06240-like isoform X1 | 245 | 3.99E-15 | 72.6 |
| TRINITY_DN15472_c0_g1_i1 | trihelix transcription factor ASIL2 | 777 | 1.09E-43 | 74.35 |
| TRINITY_DN15475_c0_g1_i1 | TPRXL isoform X2 | 1053 | 1.20E-62 | 59 |
| TRINITY_DN15480_c0_g1_i1 | receptor kinase At4g00960 | 972 | 3.12E-116 | 76.8 |
| TRINITY_DN15480_c0_g2_i1 | receptor kinase At4g00960 | 1576 | 0 | 69.55 |
| TRINITY_DN15480_c0_g2_i2 | cysteine-rich receptor kinase 10 | 2129 | 0 | 67.25 |
| TRINITY_DN15480_c0_g3_i1 | receptor kinase At4g00960 | 799 | 2.68E-111 | 77.25 |
| TRINITY_DN15485_c0_g1_i1 | ---NA--- | 670 |  |  |
| TRINITY_DN15485_c0_g2_i1 | ---NA--- | 1268 |  |  |
| TRINITY_DN15485_c0_g3_i1 | ---NA--- | 635 |  |  |
| TRINITY_DN15496_c0_g1_i1 | ---NA--- | 228 |  |  |
| TRINITY_DN1549_c0_g1_i1 | E3 ubiquitin- ligase RGLG2 | 1676 | 0 | 77.2 |
| TRINITY_DN15507_c0_g2_i1 | ---NA--- | 302 |  |  |
| TRINITY_DN15522_c0_g1_i1 | PREDICTED: uncharacterized protein LOC104894091 | 310 | 2.80E-22 | 70.9 |
| TRINITY_DN15522_c0_g2_i1 | PREDICTED: uncharacterized protein LOC108331079 | 1772 | 2.97E-92 | 61.8 |
| TRINITY_DN15526_c0_g1_i1 | serine threonine- kinase HT1-like | 1226 | 0 | 88.8 |
| TRINITY_DN15535_c0_g1_i1 | abscisic acid 8 -hydroxylase 1-like | 1021 | 5.76E-135 | 90.55 |
| TRINITY_DN15538_c0_g1_i1 | cytokinin riboside 5 -monophosphate phosphoribohydrolase LOG3 | 1172 | 3.89E-134 | 92.25 |
| TRINITY_DN15538_c1_g1_i1 | indeterminate-domain 12 | 1059 | 7.78E-08 | 70.5 |
| TRINITY_DN15539_c0_g1_i1 | delta(7)-sterol-C5(6)-desaturase-like | 773 | 1.41E-57 | 74.05 |
| TRINITY_DN15539_c0_g2_i1 | delta(7)-sterol-C5(6)-desaturase 1-like | 1327 | 7.56E-147 | 81 |
| TRINITY_DN15540_c0_g1_i1 | transcription factor TCP20-like | 1485 | 1.78E-55 | 58 |
| TRINITY_DN15540_c0_g1_i2 | transcription factor TCP20-like | 1353 | 7.35E-47 | 59.55 |
| TRINITY_DN15540_c0_g1_i3 | transcription factor TCP20-like | 1111 | 2.28E-51 | 59.55 |
| TRINITY_DN15540_c0_g1_i4 | transcription factor TCP20-like | 1243 | 2.45E-56 | 58 |
| TRINITY_DN15544_c0_g1_i1 | ---NA--- | 894 |  |  |
| TRINITY_DN15544_c0_g2_i1 | ---NA--- | 825 |  |  |
| TRINITY_DN15548_c0_g1_i1 | Pentatricopeptide repeat-containing | 2557 | 1.59E-129 | 87.5 |
| TRINITY_DN15548_c0_g1_i2 | PUTATIVE TYPE 1 MEMBRANE family | 1763 | 3.46E-133 | 66.05 |
| TRINITY_DN15548_c0_g2_i1 | Pentatricopeptide repeat-containing | 2969 | 0 | 87 |
| TRINITY_DN1554_c0_g1_i1 | ---NA--- | 1025 |  |  |
| TRINITY_DN1554_c0_g1_i2 | ---NA--- | 994 |  |  |
| TRINITY_DN15554_c0_g1_i1 | RNA polymerase II transcriptional coactivator KELP | 870 | 5.84E-65 | 76.4 |
| TRINITY_DN15555_c0_g1_i1 | MAM33 domain-containing | 1222 | 3.74E-96 | 70.55 |
| TRINITY_DN15564_c0_g1_i1 | enolase-phosphatase E1 | 1678 | 1.50E-16 | 57.33 |
| TRINITY_DN15570_c0_g1_i1 | trihelix transcription factor ASIL1 isoform X3 | 1003 | 1.97E-63 | 86.5 |
| TRINITY_DN15578_c0_g1_i1 | peptidyl-prolyl cis-trans isomerase | 1109 | 5.18E-101 | 95.3 |
| TRINITY_DN15580_c0_g1_i1 | zinc finger CCCH domain-containing 3-like isoform X1 | 1765 | 5.64E-139 | 65.55 |
| TRINITY_DN15589_c0_g1_i1 | hypothetical protein SOVF_117440 | 888 | 6.10E-127 | 91.45 |
| TRINITY_DN15589_c0_g2_i1 | hypothetical protein SOVF_117440 | 888 | 8.61E-128 | 91.45 |
| TRINITY_DN15593_c0_g1_i1 | GUCD1 isoform X1 | 1627 | 3.39E-105 | 82.4 |
| TRINITY_DN15593_c0_g1_i2 | GUCD1 isoform X1 | 1515 | 4.15E-139 | 77 |
| TRINITY_DN15593_c0_g1_i3 | GUCD1 isoform X1 | 1657 | 2.82E-103 | 78.45 |
| TRINITY_DN15593_c0_g1_i4 | GUCD1 isoform X1 | 1545 | 2.99E-136 | 74.4 |
| TRINITY_DN15593_c0_g2_i1 | GUCD1 isoform X1 | 752 | 2.52E-36 | 67.35 |
| TRINITY_DN15599_c0_g1_i1 | GATA transcription factor 8-like | 616 | 4.35E-38 | 72.15 |
| TRINITY_DN15599_c0_g1_i2 | GATA transcription factor 8-like | 622 | 4.64E-38 | 72.15 |
| TRINITY_DN15604_c0_g1_i1 | E3 SUMO- ligase MMS21 | 947 | 1.13E-98 | 74.3 |
| TRINITY_DN15604_c0_g1_i2 | E3 SUMO- ligase MMS21 | 1158 | 2.64E-107 | 72.5 |
| TRINITY_DN15604_c0_g1_i3 | E3 SUMO- ligase MMS21 | 544 | 3.63E-51 | 77.8 |
| TRINITY_DN15604_c0_g1_i4 | E3 SUMO- ligase MMS21 | 755 | 8.30E-61 | 74.5 |
| TRINITY_DN15609_c0_g1_i1 | methyltransferase 2 isoform X1 | 995 | 1.13E-51 | 57.9 |
| TRINITY_DN15609_c0_g1_i2 | ---NA--- | 215 |  |  |
| TRINITY_DN15612_c0_g1_i1 | ---NA--- | 862 |  |  |
| TRINITY_DN15612_c1_g2_i1 | ---NA--- | 361 |  |  |
| TRINITY_DN15612_c1_g2_i2 | ---NA--- | 278 |  |  |
| TRINITY_DN15612_c1_g2_i3 | ---NA--- | 357 |  |  |
| TRINITY_DN15613_c0_g1_i1 | probable plastid-lipid-associated chloroplastic | 2809 | 0 | 79.6 |
| TRINITY_DN15613_c0_g1_i2 | probable plastid-lipid-associated chloroplastic | 2840 | 0 | 79.75 |
| TRINITY_DN15613_c0_g2_i1 | probable plastid-lipid-associated chloroplastic | 978 | 4.58E-43 | 82.75 |
| TRINITY_DN15614_c0_g2_i1 | ---NA--- | 236 |  |  |
| TRINITY_DN15622_c0_g1_i1 | DEHYDRATION-INDUCED 19 homolog 3-like | 1289 | 1.94E-57 | 65.35 |
| TRINITY_DN15629_c0_g1_i1 | rubredoxin family | 1051 | 2.58E-54 | 73.05 |
| TRINITY_DN15631_c2_g1_i1 | ---NA--- | 621 |  |  |
| TRINITY_DN15633_c0_g1_i1 | O-fucosyltransferase family isoform 1 | 1694 | 0 | 87.4 |
| TRINITY_DN15633_c0_g2_i1 | O-fucosyltransferase family isoform 1 | 1694 | 0 | 87.55 |
| TRINITY_DN15633_c2_g1_i1 | hypothetical protein SOVF_149050 | 597 | 9.20E-63 | 80.55 |
| TRINITY_DN15635_c0_g1_i1 | BI1 | 941 | 2.27E-128 | 86.1 |
| TRINITY_DN15635_c0_g1_i2 | BI1 | 941 | 2.55E-133 | 86.7 |
| TRINITY_DN15640_c0_g1_i1 | transmembrane (DUF1218) | 978 | 1.62E-18 | 61.1 |
| TRINITY_DN15640_c0_g1_i2 | transmembrane (DUF1218) | 887 | 1.72E-28 | 56.45 |
| TRINITY_DN15642_c0_g1_i1 | IQ-DOMAIN 14 | 1784 | 5.66E-153 | 65.25 |
| TRINITY_DN15648_c0_g2_i1 | RNA-binding 5 | 671 | 6.55E-11 | 61.32 |
| TRINITY_DN15650_c0_g1_i1 | uridine 5 -monophosphate synthase | 1913 | 0 | 90.35 |
| TRINITY_DN15653_c0_g1_i3 | disease resistance RGA3 | 1651 | 0 | 63.8 |
| TRINITY_DN15659_c0_g1_i1 | vesicle-associated membrane 722 | 1224 | 7.85E-141 | 97 |
| TRINITY_DN15663_c0_g1_i1 | pentatricopeptide repeat-containing chloroplastic | 3078 | 0 | 89.35 |
| TRINITY_DN15663_c0_g1_i2 | pentatricopeptide repeat-containing chloroplastic | 3074 | 0 | 87.6 |
| TRINITY_DN15667_c0_g1_i1 | sn1-specific diacylglycerol lipase alpha | 706 | 1.10E-35 | 68.75 |
| TRINITY_DN15667_c0_g2_i1 | sn1-specific diacylglycerol lipase alpha | 1823 | 0 | 83.4 |
| TRINITY_DN15667_c0_g2_i2 | sn1-specific diacylglycerol lipase alpha | 1372 | 0 | 83.4 |
| TRINITY_DN15669_c0_g1_i1 | ---NA--- | 330 |  |  |
| TRINITY_DN15674_c1_g1_i1 | acyl carrier chloroplastic-like | 774 | 1.20E-37 | 77.65 |
| TRINITY_DN15677_c0_g1_i1 | F-box At3g23970 | 1182 | 1.50E-16 | 42.95 |
| TRINITY_DN15700_c1_g1_i1 | splicing factor U2af small subunit B-like | 1364 | 4.38E-111 | 96.85 |
| TRINITY_DN15706_c0_g1_i1 | FREE1 isoform X2 | 1908 | 1.03E-176 | 82.6 |
| TRINITY_DN15710_c0_g1_i1 | ABC transporter B family member chloroplastic | 641 | 7.23E-96 | 87.25 |
| TRINITY_DN15719_c0_g1_i1 | histone H2A | 873 | 9.70E-59 | 97.95 |
| TRINITY_DN15720_c0_g1_i1 | transport Sec61 subunit gamma-1-like | 564 | 1.05E-27 | 97.75 |
| TRINITY_DN15723_c0_g1_i1 | NEDD8 ultimate buster 1 | 2132 | 0 | 71.75 |
| TRINITY_DN15724_c0_g1_i1 | calponin homology domain-containing DDB_G0272472 | 1508 | 4.24E-113 | 79.1 |
| TRINITY_DN15724_c0_g1_i2 | dnaJ homolog subfamily C member 5B-like | 519 | 1.85E-06 | 94 |
| TRINITY_DN15726_c0_g1_i1 | probable methyltransferase PMT15 | 2291 | 0 | 80.6 |
| TRINITY_DN15726_c0_g2_i1 | S-adenosyl-L-methionine-dependent methyltransferase | 370 | 9.48E-09 | 63.33 |
| TRINITY_DN15729_c0_g1_i1 | scarecrow 4 | 1455 | 0 | 80.4 |
| TRINITY_DN15729_c0_g1_i2 | scarecrow 4 | 766 | 9.11E-93 | 82.2 |
| TRINITY_DN15734_c1_g1_i1 | ---NA--- | 218 |  |  |
| TRINITY_DN15735_c0_g1_i2 | transcription factor bHLH35 | 996 | 8.37E-99 | 76.4 |
| TRINITY_DN15741_c0_g1_i1 | cytochrome b561 and DOMON domain-containing At3g25290-like | 928 | 4.33E-49 | 65.8 |
| TRINITY_DN15741_c0_g1_i2 | cytochrome b561 and DOMON domain-containing At3g25290-like | 887 | 2.84E-49 | 65.8 |
| TRINITY_DN15742_c0_g1_i1 | GEM 1 | 845 | 3.85E-94 | 71.75 |
| TRINITY_DN15745_c0_g1_i1 | Replicase poly 1ab | 1372 | 3.16E-114 | 77.55 |
| TRINITY_DN15757_c0_g1_i1 | IMPACT family member in pol 5 region | 1082 | 2.73E-130 | 88.45 |
| TRINITY_DN15757_c0_g1_i2 | IMPACT family member in pol 5 region | 1094 | 1.56E-125 | 88.45 |
| TRINITY_DN15770_c0_g1_i1 | pre-mRNA cleavage factor Im 25 kDa subunit 2 | 1263 | 7.75E-136 | 95.7 |
| TRINITY_DN15771_c0_g1_i1 | cysteine ase inhibitor A-like | 1185 | 1.36E-53 | 86.45 |
| TRINITY_DN15785_c0_g1_i1 | molybdate-anion transporter-like | 1982 | 0 | 94.9 |
| TRINITY_DN15820_c0_g2_i1 | 40S ribosomal SA-like | 1323 | 9.90E-148 | 84.2 |
| TRINITY_DN15820_c0_g2_i2 | 40S ribosomal SA-like | 1559 | 1.04E-140 | 85.65 |
| TRINITY_DN15823_c0_g1_i1 | serine arginine-rich splicing factor RSZ21 | 1748 | 1.33E-48 | 88.7 |
| TRINITY_DN15824_c0_g1_i1 | probable inactive ATP-dependent zinc metalloprotease FTSHI chloroplastic | 2978 | 0 | 92.7 |
| TRINITY_DN15824_c0_g1_i2 | probable inactive ATP-dependent zinc metalloprotease FTSHI chloroplastic | 2733 | 0 | 92.7 |
| TRINITY_DN15824_c0_g1_i3 | probable inactive ATP-dependent zinc metalloprotease FTSHI chloroplastic | 2732 | 0 | 91.5 |
| TRINITY_DN15824_c0_g1_i4 | probable inactive ATP-dependent zinc metalloprotease FTSHI chloroplastic | 2977 | 0 | 91.5 |
| TRINITY_DN15828_c0_g2_i1 | glycerol kinase | 2023 | 0 | 91.35 |
| TRINITY_DN15839_c0_g1_i1 | transcription factor MYB44-like | 1236 | 3.50E-89 | 61.6 |
| TRINITY_DN15842_c0_g1_i2 | F-box LRR-repeat 3 | 2881 | 0 | 83.65 |
| TRINITY_DN15845_c0_g2_i1 | dnaJ homolog subfamily B member 1-like | 890 | 1.03E-92 | 88.1 |
| TRINITY_DN15864_c0_g1_i1 | PREDICTED: uncharacterized protein LOC107851668 | 1044 | 5.85E-53 | 71.8 |
| TRINITY_DN15864_c1_g1_i1 | ---NA--- | 305 |  |  |
| TRINITY_DN15871_c0_g1_i1 | NDR1 HIN1 12 | 1427 | 6.92E-80 | 75 |
| TRINITY_DN15874_c0_g1_i1 | DUF150 domain-containing | 1085 | 4.94E-124 | 88.1 |
| TRINITY_DN15874_c1_g1_i1 | ---NA--- | 321 |  |  |
| TRINITY_DN15876_c0_g1_i1 | casein kinase 1 HD16 | 2697 | 0 | 94.45 |
| TRINITY_DN15885_c0_g1_i1 | homeobox-leucine zipper ATHB-40 | 811 | 8.80E-83 | 67.95 |
| TRINITY_DN15885_c0_g1_i2 | homeobox-leucine zipper ATHB-40-like | 476 | 1.11E-31 | 59.45 |
| TRINITY_DN15899_c0_g1_i1 | ---NA--- | 333 |  |  |
| TRINITY_DN15902_c0_g4_i1 | ---NA--- | 552 |  |  |
| TRINITY_DN15902_c0_g4_i2 | ---NA--- | 559 |  |  |
| TRINITY_DN15902_c0_g4_i3 | ---NA--- | 758 |  |  |
| TRINITY_DN15902_c0_g4_i4 | ---NA--- | 789 |  |  |
| TRINITY_DN15906_c0_g1_i1 | ---NA--- | 417 |  |  |
| TRINITY_DN15906_c0_g2_i1 | PREDICTED: uncharacterized protein LOC104908168 | 601 | 2.15E-44 | 87 |
| TRINITY_DN15906_c0_g2_i2 | PREDICTED: uncharacterized protein LOC109015916, partial | 593 | 1.65E-34 | 86.65 |
| TRINITY_DN15906_c0_g2_i3 | PREDICTED: uncharacterized protein LOC104908168 | 499 | 2.27E-33 | 86.4 |
| TRINITY_DN15907_c0_g1_i1 | MO25 At5g47540 | 1785 | 0 | 90.35 |
| TRINITY_DN15911_c0_g2_i1 | cullin-1 isoform X1 | 1343 | 0 | 91.65 |
| TRINITY_DN15915_c0_g1_i1 | probable membrane-associated kinase regulator 1 | 667 | 4.97E-39 | 65.4 |
| TRINITY_DN15915_c1_g1_i1 | ---NA--- | 274 |  |  |
| TRINITY_DN15921_c0_g1_i1 | ---NA--- | 422 |  |  |
| TRINITY_DN15927_c0_g1_i1 | probable magnesium transporter NIPA1 | 1120 | 1.31E-119 | 89.35 |
| TRINITY_DN15928_c0_g1_i1 | thiol-disulfide oxidoreductase DCC | 1902 | 8.03E-72 | 87.8 |
| TRINITY_DN15928_c0_g1_i2 | thiol-disulfide oxidoreductase DCC | 2048 | 1.36E-65 | 85.4 |
| TRINITY_DN15928_c0_g1_i3 | thiol-disulfide oxidoreductase DCC | 2028 | 1.21E-106 | 76.9 |
| TRINITY_DN15951_c0_g1_i1 | bet1-like SNARE 1-1 | 843 | 6.79E-58 | 89.6 |
| TRINITY_DN15951_c0_g2_i1 | bet1-like SNARE 1-1 | 774 | 5.35E-57 | 88.2 |
| TRINITY_DN15951_c0_g3_i1 | bet1-like SNARE 1-1 | 774 | 5.35E-57 | 88.2 |
| TRINITY_DN15963_c0_g1_i1 | alpha 1,4-glycosyltransferase family | 1804 | 0 | 69.25 |
| TRINITY_DN15963_c0_g1_i2 | alpha 1,4-glycosyltransferase family | 1770 | 0 | 69.25 |
| TRINITY_DN15963_c1_g1_i1 | ---NA--- | 257 |  |  |
| TRINITY_DN15963_c2_g1_i1 | ---NA--- | 257 |  |  |
| TRINITY_DN15974_c0_g1_i1 | ---NA--- | 492 |  |  |
| TRINITY_DN15974_c0_g1_i2 | ---NA--- | 509 |  |  |
| TRINITY_DN15988_c0_g1_i1 | ---NA--- | 349 |  |  |
| TRINITY_DN15988_c0_g1_i2 | ---NA--- | 367 |  |  |
| TRINITY_DN15991_c1_g1_i1 | PREDICTED: uncharacterized protein At5g39570 | 656 | 6.76E-18 | 75.7 |
| TRINITY_DN15996_c0_g1_i1 | hypothetical protein SOVF_205960 | 1225 | 7.59E-88 | 75.25 |
| TRINITY_DN1599_c0_g1_i1 | la-related 1C-like | 2446 | 1.32E-85 | 55.4 |
| TRINITY_DN16014_c0_g1_i1 | PREDICTED: uncharacterized protein LOC104897978 | 1268 | 0 | 82.75 |
| TRINITY_DN16019_c0_g1_i1 | ---NA--- | 377 |  |  |
| TRINITY_DN1601_c0_g1_i1 | ---NA--- | 634 |  |  |
| TRINITY_DN1601_c0_g2_i1 | ---NA--- | 640 |  |  |
| TRINITY_DN16026_c1_g1_i1 | lisH domain-containing -like | 560 | 2.67E-43 | 63.3 |
| TRINITY_DN16027_c0_g1_i1 | serine threonine- kinase CTR1 | 2838 | 0 | 77.9 |
| TRINITY_DN16027_c0_g1_i2 | serine threonine- kinase CTR1 | 2437 | 0 | 77.15 |
| TRINITY_DN16027_c0_g2_i1 | serine threonine- kinase CTR1-like | 2182 | 0 | 75.1 |
| TRINITY_DN16030_c0_g1_i1 | U3 snoRNP-associated -like EMB2271 | 1087 | 5.83E-161 | 78.05 |
| TRINITY_DN16030_c0_g2_i1 | U3 snoRNP-associated -like EMB2271 | 1821 | 0 | 78.1 |
| TRINITY_DN16044_c0_g1_i1 | ---NA--- | 288 |  |  |
| TRINITY_DN16044_c0_g1_i2 | ---NA--- | 308 |  |  |
| TRINITY_DN16044_c0_g1_i3 | ---NA--- | 218 |  |  |
| TRINITY_DN16044_c0_g2_i1 | ---NA--- | 233 |  |  |
| TRINITY_DN16045_c0_g1_i1 | MICOS complex subunit Mic60 | 2584 | 4.07E-178 | 62.65 |
| TRINITY_DN16045_c0_g1_i2 | MICOS complex subunit mic60 isoform X1 | 2583 | 3.71E-97 | 80.7 |
| TRINITY_DN16051_c0_g1_i1 | ---NA--- | 886 |  |  |
| TRINITY_DN16054_c0_g1_i1 | V-type proton ATPase subunit E-like | 1277 | 1.66E-131 | 89.35 |
| TRINITY_DN16055_c0_g1_i1 | ---NA--- | 209 |  |  |
| TRINITY_DN16061_c0_g1_i1 | RNA-binding BRN1 | 1737 | 0 | 79.75 |
| TRINITY_DN16068_c0_g1_i1 | RNA-binding 8A-like | 1117 | 2.82E-72 | 83.25 |
| TRINITY_DN16068_c0_g1_i2 | RNA-binding 8A-like | 1147 | 3.83E-72 | 83.25 |
| TRINITY_DN16070_c0_g1_i1 | ---NA--- | 225 |  |  |
| TRINITY_DN16070_c0_g1_i2 | ---NA--- | 245 |  |  |
| TRINITY_DN16076_c0_g1_i1 | Transmembrane Fragile-X-F-associated | 1668 | 1.85E-153 | 85.65 |
| TRINITY_DN16076_c0_g1_i2 | zinc finger family | 1756 | 0 | 85.25 |
| TRINITY_DN16076_c0_g1_i3 | Transmembrane Fragile-X-F-associated | 1444 | 1.38E-141 | 84 |
| TRINITY_DN16076_c0_g1_i4 | RING finger B | 1532 | 0 | 85.4 |
| TRINITY_DN16080_c0_g1_i1 | actin-related 3 | 1735 | 0 | 96.2 |
| TRINITY_DN16082_c0_g1_i1 | hypothetical protein BVRB_5g105900 | 1141 | 1.25E-20 | 55.45 |
| TRINITY_DN16086_c0_g1_i1 | zinc finger CCCH domain-containing 29-like | 1868 | 0 | 63.65 |
| TRINITY_DN16086_c0_g2_i1 | zinc finger CCCH domain-containing 29-like | 2267 | 0 | 62.2 |
| TRINITY_DN1608_c0_g1_i1 | ---NA--- | 533 |  |  |
| TRINITY_DN16099_c0_g1_i1 | ---NA--- | 315 |  |  |
| TRINITY_DN1609_c0_g1_i1 | ---NA--- | 548 |  |  |
| TRINITY_DN16100_c0_g1_i1 | homeobox knotted-1-like 3 isoform X1 | 1725 | 1.31E-172 | 89.6 |
| TRINITY_DN16100_c0_g1_i4 | homeobox knotted-1-like 3 isoform X1 | 1958 | 7.35E-167 | 89.55 |
| TRINITY_DN16101_c0_g2_i1 | AP-1 complex subunit mu-2 | 284 | 4.02E-36 | 95.45 |
| TRINITY_DN16113_c1_g1_i1 | ---NA--- | 492 |  |  |
| TRINITY_DN16113_c3_g1_i1 | ---NA--- | 221 |  |  |
| TRINITY_DN16121_c0_g2_i1 | probable inactive receptor kinase At5g58300 | 2140 | 0 | 75.85 |
| TRINITY_DN16124_c0_g1_i1 | hypothetical protein BVRB_2g046580 | 351 | 9.48E-12 | 66.5 |
| TRINITY_DN16124_c1_g1_i1 | ---NA--- | 386 |  |  |
| TRINITY_DN1612_c0_g1_i1 | auxin response factor 7 isoform X2 | 1354 | 9.28E-139 | 75.85 |
| TRINITY_DN16131_c0_g1_i1 | G-type lectin S-receptor-like serine threonine- kinase At4g27290 | 363 | 2.03E-06 | 84 |
| TRINITY_DN16131_c0_g3_i1 | ---NA--- | 209 |  |  |
| TRINITY_DN16133_c0_g1_i1 | V-type proton ATPase subunit H | 1982 | 0 | 89.3 |
| TRINITY_DN16135_c0_g1_i1 | GDP-fucose O-fucosyltransferase | 1964 | 0 | 84.55 |
| TRINITY_DN16136_c0_g1_i1 | ---NA--- | 271 |  |  |
| TRINITY_DN16136_c0_g2_i1 | ---NA--- | 313 |  |  |
| TRINITY_DN16142_c0_g1_i1 | ---NA--- | 253 |  |  |
| TRINITY_DN16151_c0_g1_i1 | PREDICTED: uncharacterized protein LOC104906633 | 1440 | 2.07E-74 | 60.9 |
| TRINITY_DN16154_c0_g1_i1 | ribulose bisphosphate carboxylase oxygenase chloroplastic | 1368 | 5.77E-105 | 91.15 |
| TRINITY_DN16154_c0_g2_i1 | ribulose bisphosphate carboxylase oxygenase chloroplastic | 1822 | 0 | 88.2 |
| TRINITY_DN16154_c0_g2_i2 | ribulose bisphosphate carboxylase oxygenase chloroplastic | 1783 | 0 | 91.95 |
| TRINITY_DN16167_c0_g1_i1 | probable sugar phosphate phosphate translocator At2g25520 | 1736 | 0 | 92.85 |
| TRINITY_DN16167_c0_g2_i1 | probable sugar phosphate phosphate translocator At2g25520 | 1127 | 5.67E-124 | 91.2 |
| TRINITY_DN16185_c0_g1_i1 | Lactoylglutathione lyase glyoxalase I family | 771 | 2.44E-59 | 82.85 |
| TRINITY_DN16190_c0_g1_i1 | MAK16 homolog | 1294 | 1.74E-113 | 89.6 |
| TRINITY_DN16193_c0_g1_i1 | BTB POZ domain-containing At1g67900-like | 1039 | 8.04E-108 | 87.6 |
| TRINITY_DN16193_c0_g2_i1 | BTB POZ domain-containing At1g67900 | 539 | 6.98E-99 | 85.55 |
| TRINITY_DN16195_c0_g1_i1 | probable UDP-N-acetylglucosamine--peptide N-acetylglucosaminyltransferase SEC | 4319 | 0 | 90.25 |
| TRINITY_DN16197_c0_g1_i1 | ---NA--- | 321 |  |  |
| TRINITY_DN16197_c0_g2_i1 | ---NA--- | 324 |  |  |
| TRINITY_DN16200_c0_g1_i1 | cytosolic Fe-S cluster assembly factor narfl | 530 | 5.59E-09 | 93.35 |
| TRINITY_DN16201_c0_g1_i1 | transcription factor GTE6 | 1511 | 3.46E-172 | 82.2 |
| TRINITY_DN16201_c0_g2_i1 | transcription factor GTE6 | 545 | 1.29E-58 | 82.75 |
| TRINITY_DN16211_c0_g1_i1 | OBERON 4 | 679 | 8.50E-22 | 76.35 |
| TRINITY_DN16211_c0_g1_i2 | OBERON 4 | 824 | 9.94E-17 | 64.5 |
| TRINITY_DN16212_c0_g1_i1 | ---NA--- | 295 |  |  |
| TRINITY_DN16263_c0_g2_i1 | F-box kelch-repeat SKIP6 | 936 | 4.03E-06 | 91.5 |
| TRINITY_DN1626_c0_g1_i1 | CBL-interacting serine threonine- kinase 7-like | 1174 | 0 | 75.75 |
| TRINITY_DN16273_c1_g1_i1 | pentatricopeptide repeat-containing mitochondrial | 624 | 1.47E-70 | 70.15 |
| TRINITY_DN16278_c0_g2_i1 | cytidine deaminase 1-like | 972 | 2.55E-110 | 71.8 |
| TRINITY_DN1627_c0_g1_i1 | ninja-family mc410 | 1910 | 2.30E-147 | 55.05 |
| TRINITY_DN16298_c0_g1_i1 | probable aminotransferase TAT2 | 314 | 2.69E-38 | 82.05 |
| TRINITY_DN16298_c0_g2_i1 | probable aminotransferase TAT2 | 1370 | 0 | 85.8 |
| TRINITY_DN16298_c1_g1_i1 | GATA transcription factor 15-like | 367 | 1.75E-25 | 91.4 |
| TRINITY_DN16307_c0_g2_i1 | kDa class VI heat shock | 596 | 1.72E-39 | 71 |
| TRINITY_DN16307_c0_g2_i2 | kDa class VI heat shock | 763 | 4.43E-102 | 75.95 |
| TRINITY_DN16309_c0_g1_i1 | ATP-dependent Clp protease ATP-binding subunit chloroplastic | 1052 | 2.10E-85 | 80.95 |
| TRINITY_DN16316_c0_g1_i1 | metacaspase-1 | 720 | 9.59E-92 | 83.3 |
| TRINITY_DN16316_c0_g2_i1 | metacaspase-1 | 1812 | 0 | 84.8 |
| TRINITY_DN16325_c0_g1_i1 | UTP:RNA uridylyltransferase 1 | 2049 | 0 | 81 |
| TRINITY_DN16325_c0_g1_i2 | UTP:RNA uridylyltransferase 1 | 2037 | 0 | 80.35 |
| TRINITY_DN16329_c1_g1_i1 | DUF724 domain-containing 3 | 1336 | 8.04E-135 | 70.25 |
| TRINITY_DN16329_c1_g2_i1 | DUF724 domain-containing 3 | 1336 | 6.97E-129 | 69.5 |
| TRINITY_DN16330_c0_g1_i1 | transferase mitochondrial | 1314 | 1.71E-60 | 51.8 |
| TRINITY_DN16342_c0_g1_i2 | zinc finger CCHC domain-containing 7 | 1496 | 4.45E-72 | 58.8 |
| TRINITY_DN16342_c0_g1_i3 | zinc finger CCHC domain-containing 7-like | 1417 | 1.01E-71 | 58.65 |
| TRINITY_DN16344_c0_g1_i1 | ABC transporter G family member 22 isoform X1 | 2979 | 0 | 87.75 |
| TRINITY_DN16345_c0_g1_i1 | ---NA--- | 342 |  |  |
| TRINITY_DN16345_c0_g2_i1 | ---NA--- | 464 |  |  |
| TRINITY_DN16359_c1_g2_i1 | ---NA--- | 334 |  |  |
| TRINITY_DN16359_c1_g3_i1 | ---NA--- | 1096 |  |  |
| TRINITY_DN16359_c1_g4_i1 | tricalbin-3 | 2431 | 0 | 77.25 |
| TRINITY_DN16362_c0_g1_i1 | BTB POZ domain-containing At1g63850 | 2145 | 0 | 83.2 |
| TRINITY_DN16362_c0_g1_i2 | BTB POZ domain-containing At1g63850 | 1932 | 0 | 83.2 |
| TRINITY_DN16365_c0_g1_i1 | probable fructokinase-7 | 1297 | 0 | 89.25 |
| TRINITY_DN16365_c0_g1_i2 | probable fructokinase-7 | 1109 | 1.00E-163 | 89 |
| TRINITY_DN16367_c0_g1_i1 | ---NA--- | 216 |  |  |
| TRINITY_DN16367_c0_g2_i1 | ---NA--- | 222 |  |  |
| TRINITY_DN16375_c0_g1_i1 | trihelix transcription factor ASIL2 | 1190 | 6.81E-28 | 93 |
| TRINITY_DN16376_c0_g1_i2 | ---NA--- | 617 |  |  |
| TRINITY_DN16376_c0_g1_i3 | ---NA--- | 403 |  |  |
| TRINITY_DN16376_c0_g2_i1 | ---NA--- | 205 |  |  |
| TRINITY_DN16378_c0_g1_i1 | LOV domain-containing | 2852 | 0 | 93.8 |
| TRINITY_DN16378_c0_g1_i2 | LOV domain-containing | 2852 | 0 | 93.6 |
| TRINITY_DN16378_c1_g1_i1 | LOV domain-containing | 528 | 1.99E-38 | 93.95 |
| TRINITY_DN16382_c0_g1_i1 | PREDICTED: uncharacterized protein LOC104904439 | 457 | 1.20E-07 | 84.25 |
| TRINITY_DN16388_c0_g1_i1 | PREDICTED: uncharacterized protein LOC104886668 isoform X2 | 1622 | 4.75E-140 | 79.65 |
| TRINITY_DN1640_c0_g1_i1 | EID1-like F-box 2 | 1325 | 2.39E-163 | 94.2 |
| TRINITY_DN16410_c0_g1_i1 | uracil phosphoribosyltransferase | 1095 | 1.37E-143 | 90.65 |
| TRINITY_DN16418_c0_g1_i1 | cyclic dof factor 2 | 1635 | 1.80E-150 | 61.75 |
| TRINITY_DN16418_c0_g2_i1 | cyclic dof factor 2 | 1264 | 1.66E-110 | 58.7 |
| TRINITY_DN16426_c0_g1_i1 | pentatricopeptide repeat-containing mitochondrial | 1237 | 0 | 82.9 |
| TRINITY_DN16426_c0_g2_i1 | pentatricopeptide repeat-containing mitochondrial | 713 | 6.68E-31 | 59.65 |
| TRINITY_DN16426_c0_g3_i1 | pentatricopeptide repeat-containing mitochondrial | 712 | 9.97E-35 | 61.1 |
| TRINITY_DN16432_c0_g1_i2 | chaperone dnaJ 10 | 1840 | 0 | 86.95 |
| TRINITY_DN16437_c0_g1_i1 | ---NA--- | 1732 |  |  |
| TRINITY_DN1643_c0_g1_i1 | PREDICTED: uncharacterized protein LOC104891289 | 424 | 1.35E-07 | 46 |
| TRINITY_DN16444_c0_g1_i1 | probable E3 ubiquitin- ligase MARCH10 | 1912 | 6.94E-158 | 86.55 |
| TRINITY_DN16444_c0_g1_i2 | probable E3 ubiquitin- ligase MARCH10 | 1925 | 0 | 73.95 |
| TRINITY_DN16444_c0_g1_i3 | probable E3 ubiquitin- ligase MARCH10 | 1928 | 7.01E-158 | 86.55 |
| TRINITY_DN16444_c0_g1_i4 | probable E3 ubiquitin- ligase MARCH10 | 1941 | 0 | 74.2 |
| TRINITY_DN16457_c0_g1_i1 | CBL-interacting kinase 18-like | 2066 | 0 | 81.65 |
| TRINITY_DN16459_c0_g1_i1 | serine threonine- kinase SRK2H | 941 | 5.80E-162 | 88.45 |
| TRINITY_DN1645_c0_g1_i1 | exocyst complex component EXO70B1-like | 2289 | 0 | 63.5 |
| TRINITY_DN16466_c0_g1_i1 | ---NA--- | 403 |  |  |
| TRINITY_DN16466_c0_g1_i2 | ---NA--- | 392 |  |  |
| TRINITY_DN16469_c0_g1_i1 | inactive LRR receptor-like serine threonine- kinase BIR2 | 946 | 7.91E-80 | 79.85 |
| TRINITY_DN16472_c0_g1_i1 | thylakoid membrane slr0575 | 1441 | 1.52E-121 | 87.1 |
| TRINITY_DN16477_c0_g1_i1 | ---NA--- | 480 |  |  |
| TRINITY_DN16484_c0_g1_i1 | ---NA--- | 339 |  |  |
| TRINITY_DN16484_c0_g2_i1 | calcium-dependent kinase 10-like | 1153 | 2.43E-174 | 92.75 |
| TRINITY_DN16484_c0_g3_i1 | calcium-dependent kinase 10 | 2048 | 0 | 89.55 |
| TRINITY_DN16490_c0_g1_i1 | ---NA--- | 939 |  |  |
| TRINITY_DN16491_c0_g1_i1 | ---NA--- | 536 |  |  |
| TRINITY_DN16491_c0_g1_i2 | ---NA--- | 1250 |  |  |
| TRINITY_DN16492_c0_g1_i2 | zinc finger A20 and AN1 domain-containing stress-associated 8 | 992 | 5.80E-90 | 80.1 |
| TRINITY_DN16493_c0_g1_i2 | DETOXIFICATION 12 | 1766 | 0 | 84.3 |
| TRINITY_DN16498_c0_g1_i1 | calnexin homolog | 2036 | 0 | 87.95 |
| TRINITY_DN16499_c1_g2_i1 | ---NA--- | 320 |  |  |
| TRINITY_DN16499_c1_g3_i1 | hypothetical protein BVRB_5g098810 | 971 | 5.81E-26 | 62.2 |
| TRINITY_DN16506_c0_g1_i1 | ---NA--- | 405 |  |  |
| TRINITY_DN16507_c0_g1_i1 | 60S acidic ribosomal P0 | 1454 | 2.23E-168 | 93.45 |
| TRINITY_DN16509_c0_g1_i1 | PREDICTED: uncharacterized protein LOC104894701 | 797 | 1.52E-44 | 55.95 |
| TRINITY_DN16509_c0_g1_i2 | PREDICTED: uncharacterized protein LOC104894701 | 974 | 2.99E-52 | 56.25 |
| TRINITY_DN16510_c0_g1_i1 | thioredoxin chloroplastic | 1680 | 4.75E-136 | 91.8 |
| TRINITY_DN16513_c0_g1_i1 | zinc transporter chloroplastic isoform X2 | 2132 | 1.95E-80 | 87.95 |
| TRINITY_DN16515_c0_g1_i1 | ATP-dependent zinc metalloprotease FTSH chloroplastic | 2710 | 0 | 89.1 |
| TRINITY_DN16517_c1_g1_i1 | psoralen synthase-like | 402 | 6.13E-12 | 79.7 |
| TRINITY_DN1651_c0_g1_i1 | NAC domain-containing 94 | 977 | 5.68E-97 | 64.55 |
| TRINITY_DN1651_c0_g2_i1 | NAC domain-containing 94 | 965 | 1.50E-97 | 64.6 |
| TRINITY_DN16521_c0_g1_i1 | beta-galactosidase 10 | 2624 | 0 | 83.6 |
| TRINITY_DN16521_c0_g3_i1 | beta-galactosidase 10 | 2625 | 0 | 81.15 |
| TRINITY_DN16524_c0_g1_i1 | zinc finger 1 | 877 | 2.04E-39 | 57.2 |
| TRINITY_DN16524_c0_g1_i2 | zinc finger 1 | 679 | 2.83E-30 | 60.2 |
| TRINITY_DN16527_c0_g1_i2 | transcription initiation factor TFIID subunit 11-like | 1185 | 6.40E-45 | 61.7 |
| TRINITY_DN16527_c0_g1_i3 | transcription initiation factor TFIID subunit 11-like | 923 | 3.28E-37 | 62.4 |
| TRINITY_DN16530_c0_g1_i1 | probable inactive purple acid phosphatase 27 | 4007 | 0 | 87.4 |
| TRINITY_DN16533_c0_g1_i1 | Ypt Rab-GAP domain of gyp1p superfamily isoform 1 | 2621 | 0 | 69.7 |
| TRINITY_DN16533_c0_g1_i2 | Ypt Rab-GAP domain of gyp1p superfamily isoform 1 | 2606 | 0 | 69.7 |
| TRINITY_DN16534_c0_g1_i1 | probable receptor kinase At5g15080 | 1646 | 0 | 87.8 |
| TRINITY_DN16534_c0_g1_i2 | receptor-like serine threonine- kinase At3g01300 | 1986 | 4.75E-168 | 89.75 |
| TRINITY_DN16535_c0_g1_i1 | peptidyl-prolyl cis-trans isomerase CYP63 isoform X2 | 2745 | 2.75E-72 | 85.75 |
| TRINITY_DN16535_c0_g1_i2 | peptidyl-prolyl cis-trans isomerase CYP63 isoform X2 | 2763 | 9.17E-76 | 85.75 |
| TRINITY_DN16548_c0_g1_i1 | probable 6-phosphogluconolactonase chloroplastic | 1329 | 2.00E-154 | 82.7 |
| TRINITY_DN16550_c0_g1_i1 | scarecrow 3 | 1627 | 0 | 83.5 |
| TRINITY_DN16550_c0_g2_i1 | scarecrow 3 | 464 | 5.75E-30 | 87.85 |
| TRINITY_DN16552_c0_g1_i1 | vacuolar cation proton exchanger 3 | 1669 | 4.52E-173 | 81.3 |
| TRINITY_DN16552_c0_g1_i2 | vacuolar cation proton exchanger 3 | 1669 | 3.47E-146 | 80.45 |
| TRINITY_DN16554_c0_g1_i2 | ---NA--- | 853 |  |  |
| TRINITY_DN16561_c0_g1_i1 | flowering time control FPA | 3186 | 0 | 59.25 |
| TRINITY_DN16561_c0_g1_i2 | flowering time control FPA | 3741 | 0 | 59.25 |
| TRINITY_DN16564_c0_g1_i1 | ---NA--- | 2670 |  |  |
| TRINITY_DN16564_c0_g1_i2 | ---NA--- | 2280 |  |  |
| TRINITY_DN16566_c0_g1_i1 | chloride channel CLC-d isoform X1 | 2931 | 0 | 91.75 |
| TRINITY_DN16566_c0_g1_i2 | Chloride channel CLC-d | 2912 | 0 | 92 |
| TRINITY_DN16568_c0_g1_i1 | leucine-rich repeat-containing DDB_G0290503 isoform X2 | 1655 | 4.40E-170 | 68.6 |
| TRINITY_DN16568_c0_g1_i2 | leucine-rich repeat-containing DDB_G0290503 isoform X2 | 3510 | 0 | 62.75 |
| TRINITY_DN16570_c0_g1_i1 | KINESIN LIGHT CHAIN-RELATED 3 | 2394 | 0 | 79.55 |
| TRINITY_DN16570_c0_g1_i2 | KINESIN LIGHT CHAIN-RELATED 3 | 870 | 1.46E-145 | 92.35 |
| TRINITY_DN16570_c0_g2_i1 | KINESIN LIGHT CHAIN-RELATED 3 | 531 | 4.47E-69 | 87.7 |
| TRINITY_DN16578_c0_g1_i1 | phosphatidylinositol phosphatidylcholine transfer SFH8 isoform X2 | 2308 | 0 | 87.15 |
| TRINITY_DN16578_c0_g1_i2 | phosphatidylinositol phosphatidylcholine transfer SFH8 isoform X2 | 2308 | 0 | 87.25 |
| TRINITY_DN16579_c0_g1_i1 | acetate butyrate-- ligase peroxisomal | 2133 | 0 | 89 |
| TRINITY_DN16583_c0_g2_i1 | U3 small nucleolar RNA-associated 4 | 2969 | 0 | 78.25 |
| TRINITY_DN16583_c0_g3_i1 | U3 small nucleolar RNA-associated 4 | 2810 | 0 | 78.25 |
| TRINITY_DN16583_c0_g4_i1 | U3 small nucleolar RNA-associated 4 | 2811 | 0 | 78.25 |
| TRINITY_DN16583_c0_g5_i1 | U3 small nucleolar RNA-associated 4 | 3009 | 0 | 78.25 |
| TRINITY_DN16598_c0_g1_i1 | membrane of ER body isoform X3 | 2761 | 3.94E-144 | 65.5 |
| TRINITY_DN16609_c0_g1_i1 | ---NA--- | 201 |  |  |
| TRINITY_DN16609_c0_g2_i1 | ---NA--- | 450 |  |  |
| TRINITY_DN16609_c0_g2_i2 | ---NA--- | 847 |  |  |
| TRINITY_DN16624_c0_g1_i1 | chloride channel CLC-f | 2567 | 0 | 83.65 |
| TRINITY_DN16629_c0_g1_i1 | U11 U12 small nuclear ribonucleo 25 kDa | 1014 | 1.42E-77 | 90.75 |
| TRINITY_DN16629_c0_g1_i2 | U11 U12 small nuclear ribonucleo 25 kDa | 912 | 4.53E-78 | 90.75 |
| TRINITY_DN16633_c0_g1_i1 | ---NA--- | 385 |  |  |
| TRINITY_DN16633_c0_g2_i1 | ---NA--- | 708 |  |  |
| TRINITY_DN16636_c0_g1_i1 | AP2 ERF domain-containing transcription factor | 399 | 8.64E-21 | 81.4 |
| TRINITY_DN16638_c0_g1_i1 | auxin response factor 1 | 3040 | 0 | 82.55 |
| TRINITY_DN16663_c0_g2_i1 | NDR1 HIN1 12 | 975 | 1.43E-83 | 71.95 |
| TRINITY_DN16667_c0_g1_i1 | ---NA--- | 278 |  |  |
| TRINITY_DN16667_c0_g1_i2 | ---NA--- | 351 |  |  |
| TRINITY_DN1666_c0_g1_i1 | ---NA--- | 268 |  |  |
| TRINITY_DN16679_c0_g1_i1 | hypothetical protein SOVF_087310 | 802 | 6.60E-21 | 55 |
| TRINITY_DN16680_c0_g1_i1 | PREDICTED: uncharacterized protein LOC104904665 | 1974 | 0 | 82.65 |
| TRINITY_DN16687_c0_g1_i1 | Damaged dna-binding isoform 1 | 1089 | 1.61E-44 | 78.25 |
| TRINITY_DN16687_c1_g1_i1 | UPSTREAM OF FLC | 409 | 4.20E-20 | 59.95 |
| TRINITY_DN16687_c2_g1_i1 | serine arginine repetitive matrix 2 | 1111 | 2.31E-59 | 61.85 |
| TRINITY_DN16689_c1_g1_i1 | transcription factor MYB1R1 | 1234 | 1.05E-55 | 68.9 |
| TRINITY_DN16689_c1_g1_i2 | transcription factor MYB1R1 | 1417 | 3.50E-57 | 58.05 |
| TRINITY_DN16690_c1_g1_i1 | palmitoyl- thioesterase 1-like | 1216 | 3.67E-171 | 84.2 |
| TRINITY_DN16690_c1_g2_i1 | palmitoyl- thioesterase 1-like | 1219 | 1.25E-167 | 85.1 |
| TRINITY_DN16696_c0_g1_i1 | SWI SNF complex subunit SWI3C | 2453 | 0 | 71.85 |
| TRINITY_DN16697_c0_g1_i1 | Myb O | 800 | 1.08E-65 | 62.4 |
| TRINITY_DN16697_c0_g2_i1 | Homeodomain-like superfamily isoform 1 | 3609 | 0 | 56.35 |
| TRINITY_DN16698_c0_g1_i1 | NAC domain-containing 72 | 822 | 2.17E-71 | 62.45 |
| TRINITY_DN16702_c0_g1_i1 | accelerated cell death 11-like | 900 | 5.52E-107 | 85 |
| TRINITY_DN16702_c0_g2_i1 | 5 -3 exoribonuclease 4 | 3807 | 0 | 79.8 |
| TRINITY_DN16718_c0_g1_i1 | MND1-interacting 1 | 2846 | 0 | 75.55 |
| TRINITY_DN16718_c0_g2_i1 | MND1-interacting 1 | 2021 | 0 | 74.6 |
| TRINITY_DN16721_c0_g1_i1 | ---NA--- | 552 |  |  |
| TRINITY_DN16721_c1_g1_i1 | PREDICTED: uncharacterized protein LOC104888475 | 1221 | 5.28E-46 | 66.3 |
| TRINITY_DN16724_c0_g1_i1 | Fra a 1 associated | 985 | 2.37E-66 | 72.2 |
| TRINITY_DN16730_c0_g1_i1 | ---NA--- | 935 |  |  |
| TRINITY_DN16730_c0_g1_i2 | ---NA--- | 308 |  |  |
| TRINITY_DN16730_c0_g1_i3 | ---NA--- | 698 |  |  |
| TRINITY_DN16734_c0_g1_i1 | AP2-like ethylene-responsive transcription factor At2g41710 | 1679 | 0 | 81.55 |
| TRINITY_DN16737_c0_g1_i1 | B3 domain-containing Os01g0234100-like isoform X2 | 1616 | 3.87E-129 | 60.2 |
| TRINITY_DN16737_c0_g1_i2 | B3 domain-containing Os01g0234100-like isoform X1 | 1720 | 1.49E-101 | 59.5 |
| TRINITY_DN16742_c0_g1_i1 | zinc finger CCHC domain-containing 7 | 1827 | 7.51E-147 | 65.35 |
| TRINITY_DN16752_c0_g2_i1 | probable inactive poly [ADP-ribose] polymerase SRO5 | 995 | 7.88E-95 | 64.65 |
| TRINITY_DN16752_c0_g3_i1 | probable inactive poly [ADP-ribose] polymerase SRO5 | 1429 | 2.90E-107 | 66.1 |
| TRINITY_DN16752_c0_g4_i1 | probable inactive poly [ADP-ribose] polymerase SRO5 | 1005 | 2.92E-94 | 63.7 |
| TRINITY_DN16755_c0_g1_i1 | ---NA--- | 218 |  |  |
| TRINITY_DN16755_c0_g2_i1 | mitotic-spindle organizing 1A-like | 747 | 1.29E-30 | 90.8 |
| TRINITY_DN16756_c0_g2_i1 | high mobility group B 15 isoform X1 | 1833 | 5.08E-144 | 75.3 |
| TRINITY_DN16757_c0_g1_i1 | DNA damage-binding 1 | 3694 | 0 | 96.35 |
| TRINITY_DN16760_c0_g1_i1 | ---NA--- | 249 |  |  |
| TRINITY_DN16760_c0_g2_i1 | tRNA (guanine(26)-N(2))-dimethyltransferase | 1903 | 0 | 77.55 |
| TRINITY_DN16760_c0_g2_i2 | tRNA (guanine(26)-N(2))-dimethyltransferase | 1813 | 0 | 83.35 |
| TRINITY_DN16760_c0_g2_i3 | tRNA (guanine(26)-N(2))-dimethyltransferase | 1876 | 0 | 79.9 |
| TRINITY_DN16766_c0_g1_i2 | probable BOI-related E3 ubiquitin- ligase 2 | 1507 | 8.29E-37 | 64.2 |
| TRINITY_DN16769_c0_g1_i1 | F-box kelch-repeat At3g23880-like | 989 | 1.75E-46 | 49.4 |
| TRINITY_DN16779_c0_g1_i1 | CCA-adding enzyme isoform X1 | 2041 | 0 | 70.15 |
| TRINITY_DN16784_c0_g2_i1 | eukaryotic translation initiation factor 3 subunit C-like | 347 | 2.01E-19 | 69.85 |
| TRINITY_DN16786_c0_g1_i1 | hypothetical protein SOVF_054870 | 1807 | 1.01E-125 | 77.05 |
| TRINITY_DN16786_c0_g1_i2 | hypothetical protein SOVF_054870 | 1670 | 0 | 79.6 |
| TRINITY_DN16795_c0_g2_i3 | probable phosphatase 2C 9 isoform X1 | 1245 | 5.00E-167 | 88.6 |
| TRINITY_DN16797_c0_g1_i1 | PLASMODESMATA CALLOSE-BINDING PROTEIN 3-like isoform X2 | 1140 | 3.67E-32 | 65.4 |
| TRINITY_DN16799_c3_g1_i1 | ---NA--- | 213 |  |  |
| TRINITY_DN16803_c0_g1_i1 | neuroguidin | 714 | 5.11E-75 | 67.05 |
| TRINITY_DN16803_c0_g2_i1 | ---NA--- | 539 |  |  |
| TRINITY_DN16803_c0_g3_i1 | neuroguidin | 714 | 4.42E-80 | 68.1 |
| TRINITY_DN16804_c0_g1_i1 | ---NA--- | 479 |  |  |
| TRINITY_DN16806_c0_g1_i1 | ---NA--- | 270 |  |  |
| TRINITY_DN16806_c0_g3_i1 | ---NA--- | 270 |  |  |
| TRINITY_DN16808_c0_g3_i1 | ---NA--- | 1348 |  |  |
| TRINITY_DN16813_c0_g1_i1 | geraniol 8-hydroxylase | 1744 | 0 | 75.9 |
| TRINITY_DN16814_c0_g1_i1 | trichome birefringence-like 4 | 508 | 2.19E-50 | 79.15 |
| TRINITY_DN16814_c0_g1_i2 | trichome birefringence-like 4 | 1181 | 2.01E-168 | 72.85 |
| TRINITY_DN16819_c0_g1_i1 | Synergin gamma | 2776 | 2.34E-176 | 60.05 |
| TRINITY_DN16820_c0_g1_i1 | serine arginine-rich splicing factor RSZ22A-like | 824 | 1.45E-53 | 86.05 |
| TRINITY_DN16820_c0_g2_i1 | serine arginine-rich splicing factor RSZ22A-like | 1393 | 2.52E-51 | 86.05 |
| TRINITY_DN16827_c0_g1_i1 | heat shock cognate 80-like | 720 | 2.47E-17 | 82.65 |
| TRINITY_DN16827_c0_g2_i1 | Heat shock 90-1 | 883 | 1.81E-16 | 80.05 |
| TRINITY_DN16831_c0_g1_i1 | ---NA--- | 758 |  |  |
| TRINITY_DN16852_c0_g1_i1 | ---NA--- | 506 |  |  |
| TRINITY_DN16852_c0_g2_i1 | ---NA--- | 381 |  |  |
| TRINITY_DN16852_c0_g3_i1 | ---NA--- | 381 |  |  |
| TRINITY_DN16853_c0_g1_i1 | DUF639 domain-containing | 981 | 1.79E-109 | 81.65 |
| TRINITY_DN16853_c0_g2_i1 | DUF639 domain-containing | 2965 | 0 | 82.75 |
| TRINITY_DN16873_c0_g1_i2 | F-box kelch-repeat At3g06240-like | 1675 | 1.20E-116 | 64.85 |
| TRINITY_DN16874_c0_g2_i1 | RNA-binding 24-A-like isoform X1 | 1843 | 3.45E-114 | 70.8 |
| TRINITY_DN16877_c1_g1_i1 | transcriptional corepressor LEUNIG isoform X1 | 2547 | 0 | 84.6 |
| TRINITY_DN16882_c0_g1_i1 | probable WRKY transcription factor 12 | 989 | 6.81E-69 | 76.2 |
| TRINITY_DN16886_c1_g3_i1 | probable cadmium zinc-transporting ATPase chloroplastic | 2848 | 0 | 85.25 |
| TRINITY_DN16889_c0_g1_i1 | gamma-interferon-inducible lysosomal thiol reductase-like | 1501 | 3.99E-91 | 76.35 |
| TRINITY_DN16891_c0_g1_i1 | microfibrillar-associated 1-like | 1176 | 5.10E-61 | 68.6 |
| TRINITY_DN16897_c0_g1_i1 | histone deacetylase 6 | 667 | 5.03E-76 | 83.35 |
| TRINITY_DN16897_c0_g2_i1 | histone deacetylase 6 | 783 | 8.01E-76 | 83.45 |
| TRINITY_DN16905_c0_g1_i1 | two-component response regulator-like APRR2 isoform X1 | 1858 | 0 | 70.5 |
| TRINITY_DN16911_c0_g1_i1 | ---NA--- | 370 |  |  |
| TRINITY_DN16911_c0_g2_i1 | ---NA--- | 351 |  |  |
| TRINITY_DN16924_c0_g1_i1 | 6-phosphogluconate decarboxylating chloroplastic | 2006 | 0 | 93.45 |
| TRINITY_DN16925_c0_g1_i1 | probable mediator of RNA polymerase II transcription subunit 26b | 1130 | 1.06E-143 | 72.2 |
| TRINITY_DN16925_c0_g2_i1 | probable mediator of RNA polymerase II transcription subunit 26b | 1933 | 5.89E-140 | 72.25 |
| TRINITY_DN16925_c0_g2_i2 | probable mediator of RNA polymerase II transcription subunit 26b | 1863 | 2.84E-140 | 72.25 |
| TRINITY_DN16926_c0_g1_i1 | methyltransferase 7A isoform X1 | 1084 | 6.34E-122 | 74.65 |
| TRINITY_DN16926_c0_g1_i2 | methyltransferase 7A isoform X1 | 1031 | 9.86E-88 | 79.6 |
| TRINITY_DN16926_c0_g1_i3 | methyltransferase 7A isoform X1 | 1066 | 5.10E-122 | 74.65 |
| TRINITY_DN16926_c0_g1_i4 | methyltransferase 7A isoform X1 | 1049 | 1.01E-87 | 79.6 |
| TRINITY_DN16929_c0_g1_i1 | auxilin 1 | 2512 | 6.58E-145 | 72.5 |
| TRINITY_DN16931_c0_g1_i1 | poly(rC)-binding 4-like isoform X1 | 1555 | 8.50E-153 | 71.3 |
| TRINITY_DN16931_c0_g2_i1 | poly(rC)-binding 4-like isoform X1 | 1468 | 1.05E-158 | 77.7 |
| TRINITY_DN16931_c2_g1_i1 | E3 ubiquitin- ligase RBBP6 isoform X1 | 1562 | 2.97E-33 | 64.1 |
| TRINITY_DN16931_c2_g2_i1 | E3 ubiquitin- ligase RBBP6 isoform X1 | 914 | 3.24E-38 | 63.95 |
| TRINITY_DN16935_c0_g1_i1 | ---NA--- | 379 |  |  |
| TRINITY_DN16935_c0_g1_i3 | ---NA--- | 277 |  |  |
| TRINITY_DN16938_c0_g1_i1 | VQ motif-containing 4-like | 725 | 2.46E-07 | 63.45 |
| TRINITY_DN16938_c2_g1_i1 | VQ motif-containing 4 | 411 | 2.08E-15 | 92.1 |
| TRINITY_DN16959_c0_g1_i1 | ---NA--- | 1092 |  |  |
| TRINITY_DN16959_c0_g1_i2 | ---NA--- | 1006 |  |  |
| TRINITY_DN16960_c1_g1_i1 | ---NA--- | 745 |  |  |
| TRINITY_DN16964_c0_g3_i1 | ---NA--- | 304 |  |  |
| TRINITY_DN16964_c0_g4_i1 | ---NA--- | 486 |  |  |
| TRINITY_DN16966_c0_g1_i1 | Glycoside family 17 | 1313 | 2.83E-55 | 68.1 |
| TRINITY_DN16970_c0_g1_i1 | AMSH-like ubiquitin thioesterase 1 | 1980 | 0 | 74.4 |
| TRINITY_DN16972_c0_g1_i1 | U1 small nuclear ribonucleo 70 kDa-like [Erythranthe guttata] | 837 | 7.30E-35 | 57.8 |
| TRINITY_DN16981_c0_g1_i1 | SHOOT GRAVITROPISM 5-like | 1665 | 6.34E-165 | 89.5 |
| TRINITY_DN16981_c0_g2_i1 | mediator of RNA polymerase II transcription subunit 14 | 5798 | 0 | 76.9 |
| TRINITY_DN16984_c0_g1_i1 | ---NA--- | 300 |  |  |
| TRINITY_DN16989_c0_g1_i1 | probable receptor kinase At5g47070 | 1052 | 7.88E-123 | 83.55 |
| TRINITY_DN16989_c0_g1_i2 | probable receptor kinase At5g47070 | 1080 | 1.14E-122 | 83.55 |
| TRINITY_DN1698_c0_g1_i1 | molybdate transporter 1 | 609 | 4.99E-40 | 85.75 |
| TRINITY_DN16992_c0_g1_i1 | ---NA--- | 462 |  |  |
| TRINITY_DN16993_c1_g1_i1 | 3-phosphoinositide-dependent kinase 2 | 1837 | 0 | 89.05 |
| TRINITY_DN16993_c1_g2_i1 | 3-phosphoinositide-dependent kinase 2 | 557 | 5.82E-103 | 90 |
| TRINITY_DN16993_c1_g3_i1 | 3-phosphoinositide-dependent kinase | 529 | 1.48E-12 | 88.75 |
| TRINITY_DN16994_c0_g1_i1 | double-stranded RNA-binding 4-like isoform X1 | 1882 | 7.50E-79 | 66.45 |
| TRINITY_DN16994_c0_g1_i2 | double-stranded RNA-binding 4-like isoform X1 | 1847 | 5.63E-79 | 66.45 |
| TRINITY_DN16994_c0_g1_i3 | double-stranded RNA-binding 4 isoform X1 | 1492 | 7.27E-37 | 69.05 |
| TRINITY_DN16994_c0_g1_i4 | double-stranded RNA-binding 4 isoform X1 | 1527 | 8.75E-37 | 69.05 |
| TRINITY_DN16999_c0_g1_i2 | serine carboxypeptidase-like 27 | 1796 | 0 | 87.1 |
| TRINITY_DN16999_c0_g1_i4 | serine carboxypeptidase-like 27 | 1766 | 0 | 87.85 |
| TRINITY_DN16999_c0_g4_i1 | serine carboxypeptidase-like 27 | 715 | 6.00E-68 | 84 |
| TRINITY_DN17003_c0_g2_i1 | myb family transcription factor EFM | 702 | 2.82E-39 | 83.3 |
| TRINITY_DN17008_c0_g1_i1 | monosaccharide-sensing 2 | 2709 | 0 | 80.1 |
| TRINITY_DN17009_c0_g1_i1 | transcription initiation factor TFIID subunit 10 | 844 | 6.92E-67 | 91.8 |
| TRINITY_DN17009_c0_g2_i1 | transcription initiation factor TFIID subunit 10 | 891 | 1.63E-71 | 91.8 |
| TRINITY_DN17012_c0_g1_i1 | histone-lysine N-methyltransferase SUVR3 | 1254 | 4.31E-136 | 67.75 |
| TRINITY_DN17014_c0_g1_i2 | BRCA1-A complex subunit Abraxas | 1077 | 2.02E-50 | 64.9 |
| TRINITY_DN17014_c0_g1_i3 | BRISC complex subunit Abro1 | 544 | 5.90E-59 | 68.1 |
| TRINITY_DN17014_c0_g2_i1 | ---NA--- | 519 |  |  |
| TRINITY_DN17016_c0_g1_i1 | E3 ubiquitin- ligase ORTHRUS 2 | 1879 | 5.01E-175 | 78.85 |
| TRINITY_DN17016_c0_g3_i1 | myosin-4 isoform X1 | 632 | 2.21E-59 | 70.2 |
| TRINITY_DN17017_c0_g1_i1 | ---NA--- | 525 |  |  |
| TRINITY_DN17017_c0_g4_i1 | ---NA--- | 1155 |  |  |
| TRINITY_DN17036_c1_g1_i1 | ATP synthase subunit mitochondrial | 373 | 1.87E-17 | 97.75 |
| TRINITY_DN17037_c0_g1_i1 | F-box PP2-B12 | 1046 | 4.62E-126 | 66.85 |
| TRINITY_DN17037_c0_g1_i2 | F-box PP2-B12 | 1402 | 2.55E-124 | 66.85 |
| TRINITY_DN17043_c0_g1_i1 | ---NA--- | 899 |  |  |
| TRINITY_DN17043_c0_g1_i2 | homeobox BEL1 homolog | 2325 | 0 | 63.05 |
| TRINITY_DN17043_c0_g1_i3 | homeobox BEL1 homolog | 2438 | 0 | 68.55 |
| TRINITY_DN17048_c0_g1_i1 | ---NA--- | 447 |  |  |
| TRINITY_DN17048_c0_g1_i2 | ---NA--- | 212 |  |  |
| TRINITY_DN17048_c0_g1_i3 | ---NA--- | 613 |  |  |
| TRINITY_DN1704_c0_g2_i1 | phosphatidate cytidylyltransferase 1 | 851 | 1.40E-105 | 89.05 |
| TRINITY_DN17057_c0_g1_i1 | ---NA--- | 450 |  |  |
| TRINITY_DN17058_c1_g1_i1 | psbP domain-containing chloroplastic | 1305 | 1.42E-148 | 83.25 |
| TRINITY_DN17066_c0_g1_i1 | Formate--tetrahydrofolate ligase | 4018 | 0 | 54.15 |
| TRINITY_DN17075_c0_g1_i1 | probable WRKY transcription factor 31 | 530 | 1.04E-42 | 67.55 |
| TRINITY_DN17075_c1_g1_i1 | probable WRKY transcription factor 31 | 1502 | 4.67E-133 | 72.3 |
| TRINITY_DN17075_c1_g2_i1 | probable WRKY transcription factor 31 | 1484 | 3.57E-130 | 71.65 |
| TRINITY_DN17082_c0_g1_i2 | glucan endo-1,3-beta-glucosidase 14-like | 2831 | 0 | 82 |
| TRINITY_DN17083_c0_g1_i1 | ---NA--- | 355 |  |  |
| TRINITY_DN17083_c0_g1_i2 | ---NA--- | 448 |  |  |
| TRINITY_DN1708_c0_g1_i1 | PREDICTED: uncharacterized protein LOC104890422 | 415 | 1.08E-23 | 86.35 |
| TRINITY_DN17091_c0_g1_i1 | SNF2 domain-containing CLASSY 4-like | 2131 | 1.19E-25 | 58.85 |
| TRINITY_DN17091_c0_g1_i3 | SNF2 domain-containing CLASSY 4-like | 2097 | 1.34E-20 | 74 |
| TRINITY_DN17096_c1_g1_i1 | zinc finger CONSTANS-LIKE 5 | 1676 | 8.35E-106 | 54.2 |
| TRINITY_DN17102_c0_g1_i1 | ABC transporter G family member 25 | 1007 | 1.12E-165 | 83.2 |
| TRINITY_DN17102_c1_g1_i1 | ABC transporter G family member 25 | 1174 | 7.92E-168 | 80.65 |
| TRINITY_DN17103_c0_g1_i1 | uncharacterized protein LOC109836348 | 2658 | 3.42E-114 | 65.65 |
| TRINITY_DN17104_c0_g1_i1 | extra-large guanine nucleotide-binding 3 | 1314 | 5.45E-103 | 68.75 |
| TRINITY_DN17104_c0_g1_i2 | extra-large guanine nucleotide-binding 3 | 820 | 1.54E-73 | 75.35 |
| TRINITY_DN17104_c0_g1_i3 | extra-large guanine nucleotide-binding 3 | 777 | 3.26E-70 | 75.35 |
| TRINITY_DN17109_c0_g1_i1 | Retrotransposon gag | 581 | 1.10E-65 | 81.2 |
| TRINITY_DN17115_c0_g1_i1 | ---NA--- | 259 |  |  |
| TRINITY_DN17115_c1_g2_i1 | hypothetical protein SOVF_197930 | 950 | 2.94E-75 | 83.1 |
| TRINITY_DN17117_c0_g1_i1 | sterol C-5 desaturase | 788 | 1.94E-70 | 71.6 |
| TRINITY_DN17118_c0_g1_i1 | Complex1_LYR domain-containing | 749 | 1.63E-50 | 93.5 |
| TRINITY_DN17118_c0_g1_i2 | Complex1_LYR domain-containing | 709 | 7.26E-56 | 94.2 |
| TRINITY_DN17131_c0_g1_i1 | BIG GRAIN 1-like B | 1568 | 1.03E-50 | 59 |
| TRINITY_DN17139_c0_g1_i1 | DNA ligase 1-like | 1292 | 1.90E-25 | 50.6 |
| TRINITY_DN17139_c0_g1_i2 | DNA ligase 1-like | 1067 | 6.04E-26 | 50.6 |
| TRINITY_DN17145_c0_g1_i1 | O-fucosyltransferase family | 2695 | 0 | 81.95 |
| TRINITY_DN17147_c0_g2_i1 | probable WRKY transcription factor 23 | 584 | 5.26E-54 | 85.75 |
| TRINITY_DN17147_c0_g2_i2 | probable WRKY transcription factor 23 | 309 | 9.96E-27 | 86 |
| TRINITY_DN17153_c0_g1_i1 | ---NA--- | 251 |  |  |
| TRINITY_DN17165_c0_g1_i1 | ethylene-responsive transcription factor RAP2-7-like isoform X4 | 2085 | 5.86E-128 | 76.05 |
| TRINITY_DN17165_c0_g1_i2 | ethylene-responsive transcription factor RAP2-7 isoform X2 | 1649 | 3.08E-134 | 72.9 |
| TRINITY_DN17165_c0_g1_i3 | ethylene-responsive transcription factor RAP2-7 isoform X2 | 1643 | 2.89E-134 | 72.9 |
| TRINITY_DN17165_c0_g1_i4 | ethylene-responsive transcription factor RAP2-7-like isoform X4 | 2079 | 1.35E-124 | 76.05 |
| TRINITY_DN17169_c0_g1_i1 | serine arginine repetitive matrix 1 | 358 | 1.59E-21 | 76.33 |
| TRINITY_DN17169_c0_g3_i1 | serine arginine repetitive matrix 1 | 3719 | 0 | 54.5 |
| TRINITY_DN17172_c0_g1_i2 | serine arginine-rich SC35-like splicing factor SCL33 | 1405 | 1.26E-47 | 94.4 |
| TRINITY_DN17180_c0_g1_i1 | sugar carrier A-like | 2065 | 0 | 89.65 |
| TRINITY_DN17185_c0_g1_i1 | probable serine threonine kinase IREH1 isoform X1 | 3632 | 0 | 89.95 |
| TRINITY_DN17185_c0_g1_i2 | probable serine threonine kinase IREH1 isoform X1 | 3805 | 0 | 79.95 |
| TRINITY_DN17188_c0_g2_i1 | ---NA--- | 388 |  |  |
| TRINITY_DN17193_c0_g1_i1 | AT-rich interactive domain-containing 2-like | 1411 | 5.60E-90 | 63.65 |
| TRINITY_DN17195_c0_g2_i1 | stomatal closure-related actin-binding 3-like | 1886 | 0 | 86.5 |
| TRINITY_DN17197_c0_g2_i1 | LOB domain-containing 1-like | 289 | 7.49E-19 | 96.5 |
| TRINITY_DN17201_c0_g1_i1 | two-pore potassium channel 5-like | 1181 | 3.66E-133 | 80.6 |
| TRINITY_DN17201_c0_g2_i1 | two-pore potassium channel 5 isoform X1 | 502 | 6.11E-19 | 94 |
| TRINITY_DN17204_c0_g1_i1 | O-acyltransferase WSD1-like | 1850 | 0 | 77.05 |
| TRINITY_DN17206_c0_g1_i1 | ---NA--- | 371 |  |  |
| TRINITY_DN17206_c0_g2_i4 | ---NA--- | 372 |  |  |
| TRINITY_DN17208_c0_g1_i1 | palmitoyl- thioesterase 1-like isoform X1 | 1242 | 3.17E-90 | 70.25 |
| TRINITY_DN17208_c0_g1_i2 | palmitoyl- thioesterase 1 isoform X1 | 1179 | 4.54E-86 | 65.45 |
| TRINITY_DN17214_c0_g1_i1 | ---NA--- | 555 |  |  |
| TRINITY_DN17214_c0_g1_i2 | ---NA--- | 460 |  |  |
| TRINITY_DN17219_c0_g1_i1 | probable polyamine transporter At3g13620 | 1926 | 0 | 90.2 |
| TRINITY_DN17219_c0_g2_i1 | probable polyamine transporter At3g13620 | 1927 | 0 | 83.7 |
| TRINITY_DN17222_c0_g1_i2 | PLATZ transcription factor family | 1350 | 1.82E-126 | 85.8 |
| TRINITY_DN17222_c0_g1_i3 | PREDICTED: uncharacterized protein LOC106774881 | 1389 | 5.67E-118 | 84.45 |
| TRINITY_DN17222_c0_g1_i4 | PLATZ transcription factor family | 1350 | 3.42E-126 | 85.8 |
| TRINITY_DN17222_c0_g1_i5 | PREDICTED: uncharacterized protein LOC108319199 | 1501 | 9.23E-117 | 85 |
| TRINITY_DN17225_c0_g2_i2 | tobamovirus multiplication 1-like isoform X1 | 1008 | 2.51E-105 | 77.5 |
| TRINITY_DN17227_c0_g1_i1 | thiamine biosynthetic bifunctional enzyme chloroplastic isoform X1 | 1842 | 1.38E-141 | 88.95 |
| TRINITY_DN17227_c0_g1_i2 | thiamine biosynthetic bifunctional enzyme chloroplastic isoform X1 | 1888 | 1.69E-112 | 88.2 |
| TRINITY_DN17227_c0_g1_i3 | thiamine biosynthetic bifunctional enzyme chloroplastic | 2057 | 0 | 84.6 |
| TRINITY_DN17227_c0_g1_i4 | thiamine biosynthetic bifunctional enzyme chloroplastic isoform X1 | 2011 | 0 | 85.95 |
| TRINITY_DN17228_c0_g1_i1 | probable ribonuclease P MRP subunit POP5 | 1203 | 4.06E-41 | 78 |
| TRINITY_DN17228_c0_g1_i2 | probable ribonuclease P MRP subunit POP5 | 1243 | 1.15E-71 | 77.75 |
| TRINITY_DN17229_c0_g1_i1 | receptor kinase HAIKU2 | 3517 | 0 | 79.4 |
| TRINITY_DN1722_c0_g1_i1 | peroxisomal adenine nucleotide carrier 1-like | 1440 | 2.20E-172 | 86.75 |
| TRINITY_DN17238_c0_g1_i1 | calcium-dependent kinase 1-like | 2518 | 0 | 86.7 |
| TRINITY_DN17244_c0_g2_i1 | U-box domain-containing 11 | 1220 | 1.75E-86 | 67.95 |
| TRINITY_DN17253_c0_g1_i1 | ---NA--- | 250 |  |  |
| TRINITY_DN17253_c0_g1_i2 | ---NA--- | 306 |  |  |
| TRINITY_DN17253_c0_g2_i1 | ---NA--- | 306 |  |  |
| TRINITY_DN17253_c0_g2_i2 | ---NA--- | 250 |  |  |
| TRINITY_DN1725_c0_g1_i1 | (DL)-glycerol-3-phosphatase 2 | 1108 | 5.47E-150 | 89.7 |
| TRINITY_DN1725_c0_g2_i1 | (DL)-glycerol-3-phosphatase 2 | 1109 | 1.54E-113 | 91.65 |
| TRINITY_DN17270_c0_g1_i1 | LIKE COV 2 | 1255 | 1.29E-156 | 90.8 |
| TRINITY_DN17271_c0_g1_i1 | PREDICTED: uncharacterized protein LOC104898638 | 1851 | 6.16E-134 | 59.75 |
| TRINITY_DN17271_c1_g1_i1 | DNA-damage-repair toleration chloroplastic | 1048 | 1.29E-69 | 85.3 |
| TRINITY_DN17273_c0_g1_i1 | ATG8-interacting 2 | 1583 | 1.37E-110 | 63.3 |
| TRINITY_DN17273_c0_g1_i2 | ATG8-interacting 2 | 1656 | 2.90E-110 | 63.3 |
| TRINITY_DN17276_c0_g1_i1 | transport and Golgi organization 2 homolog | 2057 | 1.45E-138 | 76.55 |
| TRINITY_DN17276_c0_g1_i3 | transport and Golgi organization 2 homolog | 2200 | 5.84E-138 | 76.65 |
| TRINITY_DN17280_c0_g1_i1 | molybdate-anion transporter-like | 1982 | 0 | 92.5 |
| TRINITY_DN17280_c1_g1_i1 | ---NA--- | 202 |  |  |
| TRINITY_DN17282_c0_g1_i2 | calcium-dependent kinase 11 | 2025 | 0 | 91.95 |
| TRINITY_DN17284_c0_g1_i1 | PREDICTED: uncharacterized protein LOC104886487 | 1166 | 5.21E-161 | 85.05 |
| TRINITY_DN17290_c0_g1_i1 | acyl-coenzyme A oxidase peroxisomal isoform X2 | 2310 | 0 | 87.45 |
| TRINITY_DN17295_c0_g1_i1 | F-box At1g65770 | 697 | 1.20E-38 | 52.5 |
| TRINITY_DN17295_c0_g2_i1 | F-box At1g65770 | 696 | 2.74E-50 | 54.45 |
| TRINITY_DN17296_c0_g1_i1 | ---NA--- | 505 |  |  |
| TRINITY_DN17300_c0_g1_i1 | ---NA--- | 242 |  |  |
| TRINITY_DN17305_c0_g1_i1 | 2,3-bisphosphoglycerate-independent phosphoglycerate mutase | 2099 | 0 | 93.55 |
| TRINITY_DN17305_c0_g1_i2 | 2,3-bisphosphoglycerate-independent phosphoglycerate mutase | 1938 | 0 | 93.8 |
| TRINITY_DN17307_c0_g1_i1 | PREDICTED: uncharacterized protein LOC104895648 isoform X2 | 1292 | 1.40E-150 | 81.95 |
| TRINITY_DN17307_c0_g1_i2 | PREDICTED: uncharacterized protein LOC104895648 isoform X2 | 1358 | 7.07E-146 | 77.7 |
| TRINITY_DN17307_c0_g1_i3 | PREDICTED: uncharacterized protein LOC104895648 isoform X2 | 1307 | 2.97E-148 | 80.7 |
| TRINITY_DN17307_c0_g1_i4 | PREDICTED: uncharacterized protein LOC104895648 isoform X2 | 1373 | 3.17E-143 | 76.5 |
| TRINITY_DN1730_c0_g1_i1 | PREDICTED: uncharacterized protein LOC8271118 isoform X2 | 605 | 8.32E-31 | 70.5 |
| TRINITY_DN17314_c0_g1_i1 | ---NA--- | 249 |  |  |
| TRINITY_DN17314_c0_g1_i2 | ---NA--- | 252 |  |  |
| TRINITY_DN17324_c0_g1_i1 | NEP1-interacting -like 2 | 1274 | 1.81E-95 | 77.8 |
| TRINITY_DN17327_c0_g1_i1 | formin 14 | 2600 | 0 | 84 |
| TRINITY_DN17327_c0_g2_i1 | polyphenol oxidase | 988 | 1.33E-133 | 67.05 |
| TRINITY_DN17337_c0_g1_i2 | zinc finger CCHC domain-containing 8 isoform X3 | 2319 | 1.41E-175 | 68.4 |
| TRINITY_DN17340_c0_g1_i1 | pentatricopeptide repeat-containing At1g62350 | 1120 | 6.59E-116 | 91.2 |
| TRINITY_DN17340_c1_g1_i1 | xyloglucan 6-xylosyltransferase 2-like | 1639 | 0 | 86.75 |
| TRINITY_DN17340_c1_g1_i2 | xyloglucan 6-xylosyltransferase 2-like | 1711 | 0 | 87 |
| TRINITY_DN17340_c1_g1_i3 | xyloglucan 6-xylosyltransferase 2-like | 1232 | 0 | 93.1 |
| TRINITY_DN17340_c1_g1_i4 | xyloglucan 6-xylosyltransferase 2-like | 1304 | 0 | 93.6 |
| TRINITY_DN17348_c0_g1_i3 | phosphatase 2C 56 | 1578 | 1.49E-157 | 79.5 |
| TRINITY_DN17350_c0_g1_i1 | E3 ubiquitin- ligase UPL4 | 5317 | 0 | 68.8 |
| TRINITY_DN17350_c0_g2_i1 | E3 ubiquitin- ligase UPL4 isoform X1 | 5318 | 0 | 65.05 |
| TRINITY_DN17357_c0_g1_i1 | probable 2-carboxy-D-arabinitol-1-phosphatase | 1318 | 1.79E-164 | 81.75 |
| TRINITY_DN17357_c0_g1_i2 | probable 2-carboxy-D-arabinitol-1-phosphatase | 1331 | 1.36E-124 | 81.2 |
| TRINITY_DN17357_c0_g2_i1 | probable 2-carboxy-D-arabinitol-1-phosphatase | 923 | 2.45E-65 | 78.35 |
| TRINITY_DN17360_c0_g1_i1 | kDa class I heat shock | 839 | 4.08E-26 | 51.65 |
| TRINITY_DN17363_c0_g1_i1 | ---NA--- | 227 |  |  |
| TRINITY_DN17363_c0_g2_i1 | probable sarcosine oxidase | 1610 | 0 | 77.95 |
| TRINITY_DN17367_c0_g1_i1 | hypothetical protein BVRB_7g158300 | 787 | 2.09E-24 | 79 |
| TRINITY_DN17368_c0_g1_i1 | CASP 4C2 | 908 | 1.91E-57 | 88.7 |
| TRINITY_DN17368_c0_g1_i2 | CASP 4C1 | 934 | 8.35E-85 | 79.7 |
| TRINITY_DN17369_c0_g1_i1 | methyltransferase 22 isoform X2 | 1134 | 1.20E-144 | 73.75 |
| TRINITY_DN17369_c0_g1_i2 | methyltransferase 22 isoform X1 | 1069 | 6.13E-111 | 76.75 |
| TRINITY_DN17369_c0_g1_i3 | methyltransferase 22 isoform X1 | 1005 | 2.58E-86 | 75.55 |
| TRINITY_DN17369_c0_g1_i4 | methyltransferase 22 isoform X1 | 1070 | 3.82E-98 | 76.15 |
| TRINITY_DN17376_c0_g1_i1 | ---NA--- | 1030 |  |  |
| TRINITY_DN17379_c0_g1_i1 | PREDICTED: uncharacterized protein LOC104906572 isoform X2 | 612 | 4.88E-48 | 92.45 |
| TRINITY_DN1737_c0_g1_i1 | ---NA--- | 302 |  |  |
| TRINITY_DN17384_c0_g1_i1 | F-box family | 1329 | 2.45E-106 | 76.3 |
| TRINITY_DN17384_c0_g1_i2 | F-box family | 1341 | 2.79E-106 | 76.3 |
| TRINITY_DN17399_c0_g1_i1 | SUPPRESSOR OF GENE SILENCING 3-like | 2247 | 0 | 72.3 |
| TRINITY_DN17405_c1_g1_i1 | transcription factor PIF7 isoform X1 | 1475 | 1.81E-138 | 63.25 |
| TRINITY_DN17405_c1_g2_i1 | transcription factor PIF7 isoform X2 | 1604 | 5.53E-132 | 62.05 |
| TRINITY_DN17405_c1_g2_i2 | transcription factor PIF7 isoform X1 | 1454 | 1.33E-136 | 62.25 |
| TRINITY_DN1740_c1_g1_i1 | enhancer of mRNA-decapping 4-like | 4306 | 0 | 75.35 |
| TRINITY_DN17419_c0_g1_i1 | nascent polypeptide-associated complex subunit alpha 2 | 419 | 3.16E-16 | 74.05 |
| TRINITY_DN17427_c0_g1_i1 | PREDICTED: uncharacterized protein LOC104889078 | 554 | 9.45E-22 | 52.5 |
| TRINITY_DN17427_c0_g1_i2 | PREDICTED: uncharacterized protein LOC104889078 | 668 | 1.01E-25 | 54.5 |
| TRINITY_DN17428_c1_g1_i1 | ---NA--- | 251 |  |  |
| TRINITY_DN17442_c0_g1_i1 | F-box At5g67140 | 984 | 2.52E-113 | 83.55 |
| TRINITY_DN17442_c0_g1_i2 | F-box At5g67140 | 973 | 2.68E-126 | 84.85 |
| TRINITY_DN17442_c0_g2_i2 | ubiquitin 12 | 314 | 2.03E-06 | 99.3 |
| TRINITY_DN17450_c0_g2_i1 | NUCLEAR FUSION DEFECTIVE 4-like | 2385 | 0 | 83.6 |
| TRINITY_DN17450_c0_g2_i2 | RNA-binding MEX3B-like isoform X1 | 856 | 1.54E-32 | 59.25 |
| TRINITY_DN17450_c0_g2_i3 | NUCLEAR FUSION DEFECTIVE 4-like | 2561 | 0 | 83.6 |
| TRINITY_DN17450_c0_g2_i4 | RNA-binding MEX3B-like isoform X1 | 1032 | 5.96E-32 | 60.75 |
| TRINITY_DN17450_c0_g2_i5 | NUCLEAR FUSION DEFECTIVE 4-like | 1912 | 0 | 83.6 |
| TRINITY_DN17460_c0_g1_i2 | chloroplastic isoform X1 | 1189 | 7.15E-113 | 84.4 |
| TRINITY_DN17460_c0_g2_i1 | chloroplastic isoform X1 | 1141 | 2.39E-113 | 80.85 |
| TRINITY_DN17470_c1_g1_i1 | oxygen-evolving enhancer 3- chloroplastic | 1263 | 9.32E-100 | 85.5 |
| TRINITY_DN17470_c1_g1_i2 | oxygen-evolving enhancer 3- chloroplastic | 1122 | 4.47E-101 | 85.55 |
| TRINITY_DN17470_c1_g2_i1 | oxygen-evolving enhancer chloroplastic | 1263 | 1.15E-104 | 84.65 |
| TRINITY_DN17470_c1_g2_i2 | oxygen-evolving enhancer chloroplastic | 1122 | 3.97E-106 | 84.9 |
| TRINITY_DN17472_c0_g1_i1 | coiled-coil domain-containing SCD2-like isoform X1 | 1505 | 2.70E-152 | 78.1 |
| TRINITY_DN17472_c0_g1_i3 | coiled-coil domain-containing SCD2 | 1570 | 0 | 80.8 |
| TRINITY_DN17473_c0_g1_i1 | psbP domain-containing chloroplastic | 1320 | 1.43E-78 | 87.15 |
| TRINITY_DN17473_c0_g1_i2 | psbP domain-containing chloroplastic | 993 | 1.64E-24 | 86.85 |
| TRINITY_DN17479_c0_g1_i1 | phosphatase chloroplastic | 1373 | 0 | 81.3 |
| TRINITY_DN17479_c1_g1_i1 | 4-hydroxyphenylpyruvate dioxygenase | 1084 | 3.52E-150 | 88.55 |
| TRINITY_DN17479_c1_g1_i2 | 4-hydroxyphenylpyruvate dioxygenase | 1102 | 3.59E-149 | 87 |
| TRINITY_DN17482_c0_g1_i1 | Antigenic heat-stable 120 kDa | 1154 | 3.03E-71 | 86.3 |
| TRINITY_DN17489_c0_g1_i1 | MOB kinase activator-like 1A | 1348 | 3.88E-156 | 97.55 |
| TRINITY_DN17489_c0_g2_i1 | MOB kinase activator-like 1A | 510 | 1.26E-31 | 94.75 |
| TRINITY_DN17493_c0_g2_i1 | Pentatricopeptide repeat (PPR) superfamily isoform 2 | 2143 | 0 | 76.15 |
| TRINITY_DN17493_c0_g2_i2 | hypothetical protein BVRB_5g098730 | 2129 | 0 | 84.3 |
| TRINITY_DN17493_c0_g2_i3 | Pentatricopeptide repeat (PPR) superfamily isoform 2 | 2107 | 0 | 80.95 |
| TRINITY_DN17493_c0_g2_i4 | hypothetical protein BVRB_5g098730 | 2121 | 0 | 84.6 |
| TRINITY_DN174_c0_g1_i1 | ---NA--- | 863 |  |  |
| TRINITY_DN174_c0_g2_i1 | ---NA--- | 881 |  |  |
| TRINITY_DN17501_c0_g1_i1 | pentatricopeptide repeat-containing chloroplastic | 2143 | 0 | 79.7 |
| TRINITY_DN17501_c0_g1_i2 | pentatricopeptide repeat-containing chloroplastic | 1995 | 0 | 74.05 |
| TRINITY_DN17509_c0_g1_i1 | peroxisomal membrane PEX14 | 3377 | 0 | 79.5 |
| TRINITY_DN17512_c0_g1_i1 | glutamate--cysteine chloroplastic-like | 1966 | 0 | 89.7 |
| TRINITY_DN17512_c1_g1_i1 | ---NA--- | 306 |  |  |
| TRINITY_DN17517_c0_g1_i1 | pectinesterase 31 | 1382 | 0 | 92.3 |
| TRINITY_DN17521_c0_g1_i1 | plant F9H3-4 | 1343 | 3.79E-159 | 80.9 |
| TRINITY_DN17521_c0_g1_i2 | plant F9H3-4 | 996 | 7.79E-97 | 80.35 |
| TRINITY_DN17528_c0_g2_i1 | pentatricopeptide repeat-containing At2g37230 | 2296 | 0 | 85.35 |
| TRINITY_DN17533_c0_g1_i1 | 3-oxoacyl-[acyl-carrier- ] synthase chloroplastic | 1150 | 1.37E-159 | 92.2 |
| TRINITY_DN17533_c0_g2_i1 | 3-oxoacyl-[acyl-carrier- ] synthase 3 chloroplastic | 1506 | 4.83E-154 | 88.55 |
| TRINITY_DN17533_c0_g2_i2 | 3-oxoacyl-[acyl-carrier- ] synthase 3 chloroplastic-like | 1643 | 0 | 89 |
| TRINITY_DN17535_c0_g1_i1 | histone deacetylase HDT1-like | 1254 | 8.05E-44 | 77 |
| TRINITY_DN17536_c0_g1_i1 | NADPH:quinone oxidoreductase | 835 | 3.85E-104 | 86.4 |
| TRINITY_DN17540_c0_g1_i1 | AUGMIN subunit 5 | 2856 | 0 | 82.7 |
| TRINITY_DN17542_c0_g1_i1 | PREDICTED: uncharacterized protein LOC104884071 isoform X2 | 2240 | 0 | 75.6 |
| TRINITY_DN17542_c0_g1_i2 | PREDICTED: uncharacterized protein LOC104884071 isoform X2 | 2130 | 0 | 75.6 |
| TRINITY_DN17547_c0_g1_i1 | ---NA--- | 276 |  |  |
| TRINITY_DN17549_c0_g1_i1 | ---NA--- | 741 |  |  |
| TRINITY_DN17549_c0_g1_i2 | ---NA--- | 642 |  |  |
| TRINITY_DN17549_c0_g1_i3 | ---NA--- | 619 |  |  |
| TRINITY_DN17549_c0_g2_i1 | probable E3 ubiquitin- ligase HIP1 | 2022 | 0 | 68.55 |
| TRINITY_DN17552_c0_g1_i1 | prolyl endopeptidase-like | 2500 | 0 | 84.1 |
| TRINITY_DN17558_c0_g1_i1 | ABC transporter C family member 10-like | 324 | 3.70E-09 | 99 |
| TRINITY_DN17558_c0_g2_i1 | PREDICTED: uncharacterized protein LOC105109055 | 1101 | 4.92E-54 | 94.1 |
| TRINITY_DN17559_c0_g1_i1 | alcohol dehydrogenase-like | 562 | 6.06E-29 | 91.35 |
| TRINITY_DN17559_c0_g2_i2 | alcohol dehydrogenase-like | 2148 | 0 | 90.95 |
| TRINITY_DN17559_c0_g2_i3 | alcohol dehydrogenase 1-like | 2273 | 1.69E-135 | 88.6 |
| TRINITY_DN17560_c0_g1_i1 | kinesin KIN- chloroplastic | 3227 | 0 | 83.45 |
| TRINITY_DN17560_c0_g1_i2 | kinesin KIN- chloroplastic | 3246 | 0 | 86.3 |
| TRINITY_DN17562_c0_g1_i1 | CDK5RAP1 | 2241 | 0 | 88.05 |
| TRINITY_DN17562_c0_g1_i2 | CDK5RAP1 | 2118 | 0 | 88.05 |
| TRINITY_DN17562_c0_g1_i3 | CDK5RAP1 | 2369 | 0 | 88.05 |
| TRINITY_DN17565_c1_g1_i1 | ---NA--- | 493 |  |  |
| TRINITY_DN17569_c0_g1_i1 | 60S ribosomal L9 | 487 | 9.78E-79 | 91.65 |
| TRINITY_DN1756_c0_g1_i1 | ---NA--- | 689 |  |  |
| TRINITY_DN1756_c0_g2_i1 | ---NA--- | 689 |  |  |
| TRINITY_DN17578_c1_g1_i1 | ADP-ribosylation factor GTPase-activating AGD12 | 1592 | 0 | 89.5 |
| TRINITY_DN17582_c0_g1_i1 | KH domain-containing At2g38610 | 1126 | 7.00E-132 | 77.15 |
| TRINITY_DN17582_c0_g1_i2 | KH domain-containing At2g38610 | 1372 | 2.22E-160 | 74.65 |
| TRINITY_DN17582_c0_g1_i3 | KH domain-containing At2g38610 | 1243 | 2.92E-168 | 89.55 |
| TRINITY_DN17585_c0_g1_i2 | U3 small nucleolar RNA-associated 10 and NUC211 domain-containing isoform 1 | 5153 | 0 | 67.9 |
| TRINITY_DN17585_c0_g1_i3 | ---NA--- | 505 |  |  |
| TRINITY_DN17585_c0_g1_i4 | U3 small nucleolar RNA-associated 10 and NUC211 domain-containing isoform 1 | 4814 | 0 | 67.9 |
| TRINITY_DN17588_c0_g1_i1 | glycine dehydrogenase (decarboxylating) mitochondrial | 3821 | 0 | 89.95 |
| TRINITY_DN17592_c0_g1_i1 | G3BP isoform X2 | 1780 | 0 | 65.65 |
| TRINITY_DN17592_c0_g2_i1 | ---NA--- | 297 |  |  |
| TRINITY_DN17595_c0_g1_i1 | C2H2-like zinc finger isoform 2 | 2457 | 3.53E-156 | 78.9 |
| TRINITY_DN17599_c0_g2_i1 | ---NA--- | 980 |  |  |
| TRINITY_DN17602_c0_g1_i1 | hsp70-Hsp90 organizing 3 | 2244 | 0 | 84.75 |
| TRINITY_DN17602_c0_g1_i2 | hsp70-Hsp90 organizing 3 | 2250 | 0 | 84.75 |
| TRINITY_DN17602_c0_g1_i3 | ---NA--- | 427 |  |  |
| TRINITY_DN17604_c0_g1_i1 | DUF246 domain-containing At1g04910 family | 1805 | 0 | 77.8 |
| TRINITY_DN17604_c0_g2_i1 | GDP-fucose O-fucosyltransferase | 1817 | 0 | 78.9 |
| TRINITY_DN17611_c0_g1_i1 | pheromone-processing carboxypeptidase KEX1-like | 556 | 1.02E-15 | 65.9 |
| TRINITY_DN17611_c2_g1_i1 | serine threonine- kinase 2 19-like | 769 | 1.95E-164 | 94.1 |
| TRINITY_DN17624_c0_g2_i1 | F-box PP2-A12 | 270 | 3.19E-35 | 81.7 |
| TRINITY_DN17626_c0_g1_i1 | F-box At4g00755 | 670 | 4.66E-17 | 88 |
| TRINITY_DN17629_c0_g1_i1 | ABC transporter G family member 15-like | 2410 | 0 | 86.35 |
| TRINITY_DN17629_c0_g2_i1 | rubisco activase | 1840 | 0 | 87.9 |
| TRINITY_DN17629_c0_g3_i1 | ribulose bisphosphate carboxylase oxygenase chloroplastic isoform X1 | 1839 | 0 | 85.6 |
| TRINITY_DN17629_c0_g4_i1 | ribulose bisphosphate carboxylase oxygenase chloroplastic isoform X1 | 1806 | 0 | 87.6 |
| TRINITY_DN17631_c1_g1_i1 | cyclin-T1-3-like | 722 | 8.01E-19 | 79 |
| TRINITY_DN17631_c1_g2_i1 | ---NA--- | 669 |  |  |
| TRINITY_DN17631_c1_g2_i2 | cyclin-T1-3-like | 1024 | 2.32E-17 | 82.5 |
| TRINITY_DN17631_c1_g2_i3 | ---NA--- | 700 |  |  |
| TRINITY_DN17631_c1_g2_i4 | cyclin-T1-3-like | 916 | 1.53E-13 | 81.4 |
| TRINITY_DN17631_c1_g2_i5 | ---NA--- | 812 |  |  |
| TRINITY_DN17632_c0_g1_i1 | nuclear transcription factor Y subunit A-10-like | 1154 | 1.42E-119 | 60.15 |
| TRINITY_DN17634_c0_g1_i1 | helicase-like transcription factor CHR28 isoform X1 | 3814 | 0 | 79.9 |
| TRINITY_DN17634_c0_g1_i2 | helicase-like transcription factor CHR28 isoform X2 | 3980 | 0 | 73 |
| TRINITY_DN17636_c0_g1_i1 | probable proteasome inhibitor | 1374 | 1.82E-115 | 70.05 |
| TRINITY_DN17636_c1_g1_i1 | ---NA--- | 230 |  |  |
| TRINITY_DN17639_c0_g1_i1 | NADH dehydrogenase [ubiquinone] 1 beta subcomplex subunit 10-B-like | 693 | 2.31E-50 | 90.1 |
| TRINITY_DN17641_c0_g1_i1 | ---NA--- | 458 |  |  |
| TRINITY_DN17641_c0_g2_i1 | ---NA--- | 259 |  |  |
| TRINITY_DN17641_c0_g3_i1 | ---NA--- | 259 |  |  |
| TRINITY_DN17641_c0_g4_i1 | ---NA--- | 258 |  |  |
| TRINITY_DN17641_c0_g5_i1 | ---NA--- | 452 |  |  |
| TRINITY_DN17641_c0_g6_i1 | AChain The Angstroms Structure Of Pokeweed Antiviral | 1548 | 2.43E-45 | 54.05 |
| TRINITY_DN17641_c0_g6_i2 | AChain The Angstroms Structure Of Pokeweed Antiviral | 1536 | 2.26E-45 | 54.05 |
| TRINITY_DN17641_c0_g7_i1 | ---NA--- | 454 |  |  |
| TRINITY_DN17641_c0_g8_i1 | ---NA--- | 434 |  |  |
| TRINITY_DN17641_c0_g9_i1 | ---NA--- | 438 |  |  |
| TRINITY_DN17643_c0_g1_i1 | zinc finger CCCH domain-containing 18 | 2032 | 0 | 74.25 |
| TRINITY_DN17643_c0_g1_i2 | Zinc finger CCCH domain-containing 18 | 1997 | 1.64E-109 | 70.15 |
| TRINITY_DN17643_c0_g1_i3 | Zinc finger CCCH domain-containing 18 | 2058 | 4.54E-142 | 74.75 |
| TRINITY_DN17643_c0_g1_i4 | zinc finger CCCH domain-containing 18 | 1971 | 1.90E-140 | 72.35 |
| TRINITY_DN17648_c0_g1_i1 | bark storage A | 1352 | 1.25E-110 | 55.85 |
| TRINITY_DN17648_c0_g1_i2 | bark storage A | 1349 | 5.34E-96 | 54.55 |
| TRINITY_DN1764_c0_g1_i1 | calcium-dependent kinase 17-like | 656 | 1.33E-70 | 78.6 |
| TRINITY_DN17660_c1_g1_i1 | 3-dehydroquinate chloroplastic | 1829 | 0 | 93 |
| TRINITY_DN17660_c1_g2_i1 | 3-dehydroquinate chloroplastic | 1820 | 0 | 93 |
| TRINITY_DN17660_c2_g1_i1 | LOW PSII ACCUMULATION chloroplastic | 1431 | 0 | 89.95 |
| TRINITY_DN17660_c2_g1_i2 | LOW PSII ACCUMULATION chloroplastic | 1419 | 0 | 90.85 |
| TRINITY_DN17661_c1_g1_i2 | plant UBX domain-containing 4 | 1197 | 5.70E-140 | 77.75 |
| TRINITY_DN17662_c0_g1_i1 | polyadenylate-binding -interacting 12 | 782 | 5.83E-38 | 69 |
| TRINITY_DN17662_c0_g1_i2 | polyadenylate-binding -interacting 12 | 800 | 1.57E-39 | 65.95 |
| TRINITY_DN17680_c0_g1_i1 | importin subunit beta-1-like | 3188 | 0 | 85.15 |
| TRINITY_DN17692_c0_g1_i1 | ribulose bisphosphate carboxylase small chloroplastic | 772 | 3.60E-97 | 83.4 |
| TRINITY_DN17692_c0_g1_i2 | ribulose bisphosphate carboxylase small chloroplastic | 846 | 8.34E-97 | 83.4 |
| TRINITY_DN17706_c0_g1_i1 | hypothetical protein SOVF_175690 | 807 | 5.06E-88 | 77.1 |
| TRINITY_DN17709_c0_g1_i1 | enoyl- hydratase peroxisomal | 1360 | 1.76E-175 | 85.25 |
| TRINITY_DN17710_c0_g1_i1 | 3-oxoacyl-[acyl-carrier- ] synthase chloroplastic-like isoform X1 | 833 | 1.37E-61 | 95.15 |
| TRINITY_DN17711_c0_g1_i1 | cytosolic sulfotransferase 15-like | 1187 | 0 | 71.75 |
| TRINITY_DN17713_c0_g1_i1 | cytochrome P450 90B1 | 1828 | 0 | 82.45 |
| TRINITY_DN17713_c0_g1_i2 | cytochrome P450 90B1 | 2053 | 0 | 86.9 |
| TRINITY_DN17715_c0_g1_i1 | glutamyl-tRNA(Gln) amidotransferase subunit chloroplastic mitochondrial | 945 | 1.44E-58 | 84.7 |
| TRINITY_DN17715_c0_g1_i2 | glutamyl-tRNA(Gln) amidotransferase subunit chloroplastic mitochondrial | 852 | 3.08E-58 | 84.4 |
| TRINITY_DN17715_c0_g1_i3 | glutamyl-tRNA(Gln) amidotransferase subunit chloroplastic mitochondrial | 649 | 5.31E-60 | 84.7 |
| TRINITY_DN17721_c0_g1_i1 | 3-oxo-5-alpha-steroid 4-dehydrogenase 1 | 1164 | 1.95E-166 | 89.95 |
| TRINITY_DN17721_c1_g1_i1 | ---NA--- | 258 |  |  |
| TRINITY_DN17721_c1_g2_i1 | ---NA--- | 262 |  |  |
| TRINITY_DN17721_c1_g3_i1 | ---NA--- | 260 |  |  |
| TRINITY_DN17721_c1_g4_i1 | ---NA--- | 261 |  |  |
| TRINITY_DN17721_c1_g5_i1 | ---NA--- | 259 |  |  |
| TRINITY_DN1772_c0_g1_i1 | ---NA--- | 579 |  |  |
| TRINITY_DN1772_c1_g1_i1 | ---NA--- | 231 |  |  |
| TRINITY_DN17732_c0_g1_i1 | cell wall vacuolar inhibitor of fructosidase 1 | 792 | 1.35E-73 | 63.55 |
| TRINITY_DN17736_c0_g1_i1 | TRANSPORT INHIBITOR RESPONSE 1 | 2257 | 0 | 84.35 |
| TRINITY_DN17736_c0_g2_i1 | TRANSPORT INHIBITOR RESPONSE 1 | 2437 | 0 | 84.4 |
| TRINITY_DN17737_c0_g1_i1 | ---NA--- | 361 |  |  |
| TRINITY_DN17737_c0_g1_i2 | ---NA--- | 434 |  |  |
| TRINITY_DN17737_c0_g1_i3 | ---NA--- | 647 |  |  |
| TRINITY_DN17737_c0_g2_i1 | hypothetical protein CICLE_v10031676mg | 1405 | 4.52E-135 | 79.5 |
| TRINITY_DN17740_c0_g1_i5 | ADP-ribosylation factor 2 | 1203 | 8.62E-130 | 98.6 |
| TRINITY_DN17743_c0_g1_i1 | mitochondrial adenine nucleotide transporter ADNT1 | 1563 | 0 | 93 |
| TRINITY_DN17744_c1_g1_i1 | casein kinase II subunit alpha | 1638 | 0 | 95.5 |
| TRINITY_DN17744_c1_g2_i1 | casein kinase II subunit alpha | 1638 | 0 | 97.35 |
| TRINITY_DN17744_c1_g3_i1 | ---NA--- | 304 |  |  |
| TRINITY_DN17747_c0_g1_i1 | HGH1 homolog | 1654 | 3.81E-125 | 86.2 |
| TRINITY_DN17753_c0_g1_i1 | ---NA--- | 1176 |  |  |
| TRINITY_DN17753_c0_g1_i3 | ---NA--- | 646 |  |  |
| TRINITY_DN17753_c0_g1_i4 | ---NA--- | 657 |  |  |
| TRINITY_DN17753_c0_g1_i6 | ---NA--- | 1077 |  |  |
| TRINITY_DN17753_c0_g1_i7 | ---NA--- | 1161 |  |  |
| TRINITY_DN17753_c0_g1_i8 | ---NA--- | 1092 |  |  |
| TRINITY_DN17756_c0_g1_i1 | zinc finger 511 | 1417 | 3.71E-119 | 81.6 |
| TRINITY_DN17756_c0_g1_i3 | zinc finger 511 | 1306 | 1.06E-119 | 81.63 |
| TRINITY_DN17759_c0_g1_i1 | photosystem II repair PSB27- chloroplastic | 800 | 4.23E-71 | 86.1 |
| TRINITY_DN17759_c0_g1_i2 | ---NA--- | 362 |  |  |
| TRINITY_DN17759_c0_g1_i3 | photosystem II repair PSB27- chloroplastic | 812 | 2.24E-68 | 86 |
| TRINITY_DN17764_c0_g1_i1 | heavy metal-associated isoprenylated plant 33 | 1606 | 1.49E-08 | 72.33 |
| TRINITY_DN17765_c0_g1_i1 | probable aquaporin SIP2-1 | 203 | 8.79E-13 | 76.9 |
| TRINITY_DN17765_c0_g2_i1 | ---NA--- | 224 |  |  |
| TRINITY_DN17768_c0_g1_i1 | ubiquitin receptor RAD23d | 1891 | 2.27E-161 | 81.75 |
| TRINITY_DN17768_c1_g1_i1 | ubiquitin receptor RAD23d | 660 | 7.01E-95 | 82.25 |
| TRINITY_DN17774_c0_g1_i1 | trans-cinnamate 4-monooxygenase | 2130 | 0 | 95.25 |
| TRINITY_DN17777_c0_g1_i1 | glutaredoxin domain-containing cysteine-rich CG12206 | 1687 | 2.41E-135 | 64.75 |
| TRINITY_DN17782_c0_g1_i1 | armadillo repeat-containing 6 | 658 | 8.99E-38 | 63.4 |
| TRINITY_DN17787_c1_g1_i1 | ---NA--- | 743 |  |  |
| TRINITY_DN17787_c2_g1_i1 | kish-like | 754 | 2.89E-40 | 95.3 |
| TRINITY_DN17792_c0_g10_i1 | ATP-dependent zinc metalloprotease FTSH chloroplastic | 2406 | 0 | 92.75 |
| TRINITY_DN17792_c0_g11_i1 | ATP-dependent zinc metalloprotease FTSH chloroplastic | 827 | 8.60E-95 | 81.55 |
| TRINITY_DN17792_c0_g12_i1 | ATP-dependent zinc metalloprotease FTSH chloroplastic | 837 | 9.97E-95 | 81.55 |
| TRINITY_DN17792_c0_g13_i1 | ATP-dependent zinc metalloprotease FTSH chloroplastic | 845 | 1.07E-94 | 81.55 |
| TRINITY_DN17792_c0_g14_i1 | ATP-dependent zinc metalloprotease FTSH chloroplastic | 861 | 1.34E-94 | 81.55 |
| TRINITY_DN17792_c0_g15_i1 | ATP-dependent zinc metalloprotease FTSH chloroplastic | 831 | 9.26E-95 | 81.55 |
| TRINITY_DN17792_c0_g16_i1 | ATP-dependent zinc metalloprotease FTSH chloroplastic | 823 | 8.28E-95 | 81.55 |
| TRINITY_DN17792_c0_g17_i1 | ATP-dependent zinc metalloprotease FTSH chloroplastic | 839 | 9.97E-95 | 81.55 |
| TRINITY_DN17792_c0_g18_i1 | ATP-dependent zinc metalloprotease FTSH chloroplastic | 833 | 9.26E-95 | 81.55 |
| TRINITY_DN17792_c0_g19_i1 | ATP-dependent zinc metalloprotease FTSH chloroplastic | 841 | 1.04E-94 | 81.55 |
| TRINITY_DN17792_c0_g1_i1 | ATP-dependent zinc metalloprotease FTSH chloroplastic | 843 | 1.07E-94 | 81.55 |
| TRINITY_DN17792_c0_g20_i1 | ATP-dependent zinc metalloprotease FTSH chloroplastic | 849 | 1.16E-94 | 81.55 |
| TRINITY_DN17792_c0_g2_i1 | ATP-dependent zinc metalloprotease FTSH chloroplastic | 859 | 1.29E-94 | 81.55 |
| TRINITY_DN17792_c0_g3_i1 | ATP-dependent zinc metalloprotease FTSH chloroplastic | 835 | 9.61E-95 | 81.55 |
| TRINITY_DN17792_c0_g4_i1 | ATP-dependent zinc metalloprotease FTSH chloroplastic | 857 | 1.25E-94 | 81.55 |
| TRINITY_DN17792_c0_g5_i1 | ATP-dependent zinc metalloprotease FTSH chloroplastic | 703 | 1.97E-99 | 81.55 |
| TRINITY_DN17792_c0_g6_i1 | ATP-dependent zinc metalloprotease FTSH chloroplastic | 825 | 8.60E-95 | 81.55 |
| TRINITY_DN17792_c0_g7_i1 | ---NA--- | 242 |  |  |
| TRINITY_DN17792_c0_g8_i1 | ATP-dependent zinc metalloprotease FTSH chloroplastic | 851 | 1.16E-94 | 81.55 |
| TRINITY_DN17792_c0_g9_i1 | ATP-dependent zinc metalloprotease FTSH chloroplastic | 829 | 9.63E-99 | 81.55 |
| TRINITY_DN17800_c0_g1_i1 | ethylene-responsive transcription factor RAP2-4-like | 994 | 2.05E-61 | 66.8 |
| TRINITY_DN17806_c0_g1_i1 | DEAD-box ATP-dependent RNA helicase 21 | 2558 | 0 | 96.1 |
| TRINITY_DN17806_c0_g1_i2 | DEAD-box ATP-dependent RNA helicase 21 | 2801 | 0 | 96.1 |
| TRINITY_DN17815_c0_g1_i1 | L-galactose dehydrogenase | 1679 | 0 | 88.45 |
| TRINITY_DN17815_c0_g2_i1 | f-box family | 310 | 1.18E-06 | 57 |
| TRINITY_DN17818_c0_g2_i1 | 26S proteasome non-ATPase regulatory subunit 11 homolog | 1470 | 0 | 94.9 |
| TRINITY_DN17818_c0_g2_i2 | 26S proteasome non-ATPase regulatory subunit 11 homolog | 1330 | 0 | 94.9 |
| TRINITY_DN17819_c0_g1_i1 | photosystem I reaction center subunit chloroplastic-like | 2250 | 2.36E-78 | 85.55 |
| TRINITY_DN17820_c0_g1_i1 | hypothetical protein BVRB_6g151040 | 480 | 5.19E-43 | 53.9 |
| TRINITY_DN17820_c1_g1_i1 | ---NA--- | 536 |  |  |
| TRINITY_DN17820_c1_g2_i1 | triphosphate tunel metalloenzyme 3 isoform X1 | 1524 | 3.10E-110 | 77.75 |
| TRINITY_DN17820_c1_g3_i1 | triphosphate tunel metalloenzyme 3 isoform X1 | 1457 | 4.00E-111 | 77.8 |
| TRINITY_DN17832_c2_g1_i1 | glucose-6-phosphate 1- cytoplasmic isoform | 2107 | 0 | 90.9 |
| TRINITY_DN17837_c0_g1_i1 | Transcriptional coactivator Hfi1 Transcriptional adapter 1 | 1618 | 1.67E-167 | 68.95 |
| TRINITY_DN17838_c0_g2_i1 | tRNA (guanine(10)-N2)-methyltransferase homolog | 1839 | 0 | 87.75 |
| TRINITY_DN17838_c0_g3_i1 | tRNA (guanine(10)-N2)-methyltransferase homolog | 908 | 6.95E-150 | 86.1 |
| TRINITY_DN17849_c0_g1_i1 | Histone | 897 | 2.56E-98 | 96.6 |
| TRINITY_DN1784_c0_g1_i1 | ---NA--- | 223 |  |  |
| TRINITY_DN17859_c1_g1_i1 | homeobox-leucine zipper HAT5 | 735 | 4.68E-13 | 79.8 |
| TRINITY_DN17862_c0_g1_i1 | serine threonine- kinase HT1-like | 2062 | 0 | 71.95 |
| TRINITY_DN17862_c0_g1_i2 | serine threonine- kinase HT1 | 1365 | 0 | 82.5 |
| TRINITY_DN17862_c0_g2_i1 | serine threonine- kinase HT1-like | 1406 | 7.82E-105 | 58.95 |
| TRINITY_DN17867_c0_g1_i1 | Zinc finger (C3HC4-type RING finger) family | 2520 | 0 | 74.65 |
| TRINITY_DN17871_c0_g1_i1 | defensin Ec-AMP-D2-like | 600 | 4.49E-23 | 76.8 |
| TRINITY_DN17874_c0_g1_i1 | CRS2-associated factor chloroplastic | 2006 | 0 | 75.6 |
| TRINITY_DN17879_c0_g1_i1 | transcription factor TCP20-like | 998 | 9.81E-73 | 65.55 |
| TRINITY_DN17880_c0_g1_i1 | bifunctional phosphatase chloroplastic | 1150 | 1.81E-106 | 70.9 |
| TRINITY_DN17880_c0_g1_i2 | bifunctional phosphatase chloroplastic | 1084 | 3.48E-107 | 69.65 |
| TRINITY_DN17882_c0_g1_i1 | PHD finger ALFIN-LIKE 1-like | 1375 | 3.91E-123 | 93.7 |
| TRINITY_DN17882_c0_g1_i2 | PHD finger ALFIN-LIKE 1-like | 1375 | 9.80E-124 | 93.75 |
| TRINITY_DN17888_c0_g1_i1 | Histone-lysine N-methyltransferase ATX1 | 4137 | 0 | 65.6 |
| TRINITY_DN17888_c0_g1_i2 | Histone-lysine N-methyltransferase ATX1 | 4094 | 0 | 66.2 |
| TRINITY_DN17899_c0_g1_i1 | GDT1 3 | 1476 | 1.95E-147 | 86.5 |
| TRINITY_DN178_c0_g1_i1 | aspartyl protease family At5g10770-like | 1956 | 0 | 74.6 |
| TRINITY_DN17900_c0_g1_i1 | probable methyltransferase PMT2 | 2586 | 0 | 89.25 |
| TRINITY_DN17900_c0_g1_i2 | probable methyltransferase PMT2 | 2586 | 0 | 89.1 |
| TRINITY_DN17905_c0_g1_i1 | F-box SKIP5 | 1694 | 3.23E-66 | 91.8 |
| TRINITY_DN17905_c0_g1_i2 | F-box SKIP5 | 1239 | 8.00E-143 | 86.6 |
| TRINITY_DN17905_c0_g1_i3 | F-box SKIP5 | 1663 | 7.86E-57 | 83.25 |
| TRINITY_DN17905_c0_g1_i4 | F-box SKIP5 | 1208 | 1.10E-116 | 86.7 |
| TRINITY_DN17914_c0_g1_i1 | ---NA--- | 418 |  |  |
| TRINITY_DN17914_c0_g1_i2 | serine arginine repetitive matrix 1-like | 530 | 7.41E-18 | 63.9 |
| TRINITY_DN17917_c0_g1_i1 | stress enhanced chloroplastic | 1352 | 3.31E-65 | 63.7 |
| TRINITY_DN17918_c0_g2_i1 | ---NA--- | 694 |  |  |
| TRINITY_DN17920_c0_g1_i1 | probable serine threonine- kinase At5g41260 | 2128 | 0 | 90.15 |
| TRINITY_DN17924_c0_g1_i1 | ---NA--- | 489 |  |  |
| TRINITY_DN17924_c0_g1_i2 | mediator of RNA polymerase II transcription subunit 25 isoform X1 | 525 | 1.43E-14 | 79.85 |
| TRINITY_DN17951_c0_g1_i1 | transcription factor Pur-alpha 1-like isoform X2 | 1569 | 3.65E-165 | 88.3 |
| TRINITY_DN17955_c1_g3_i1 | ubiquitin-NEDD8 RUB2 | 497 | 1.64E-61 | 99.55 |
| TRINITY_DN17955_c1_g4_i1 | ubiquitin-NEDD8 RUB2 | 568 | 1.49E-79 | 98.95 |
| TRINITY_DN17961_c0_g1_i1 | ribonuclease J isoform X1 | 2976 | 0 | 84.55 |
| TRINITY_DN17965_c0_g1_i1 | 26S proteasome non-ATPase regulatory subunit 13 homolog A | 1570 | 0 | 95.05 |
| TRINITY_DN17965_c0_g1_i4 | 26S proteasome non-ATPase regulatory subunit 13 homolog A | 1727 | 0 | 95.05 |
| TRINITY_DN17967_c0_g1_i1 | ---NA--- | 799 |  |  |
| TRINITY_DN17967_c0_g1_i2 | ---NA--- | 861 |  |  |
| TRINITY_DN17975_c0_g1_i1 | IQ domain-containing IQM1 | 2033 | 0 | 75.35 |
| TRINITY_DN17975_c0_g1_i2 | IQ domain-containing IQM1 | 1169 | 4.28E-160 | 79.25 |
| TRINITY_DN17982_c0_g2_i1 | PREDICTED: uncharacterized protein At3g17950 isoform X1 | 932 | 6.63E-90 | 72.75 |
| TRINITY_DN17983_c0_g2_i1 | hypothetical protein SOVF_011560 | 2380 | 0 | 76.55 |
| TRINITY_DN17985_c0_g1_i1 | calcium-dependent kinase 1 | 2250 | 0 | 92.7 |
| TRINITY_DN17986_c0_g1_i1 | ORF able to induce HR-like lesions | 842 | 1.88E-76 | 91.1 |
| TRINITY_DN17988_c0_g2_i1 | phenylalanine ammonia-lyase | 2629 | 0 | 91.85 |
| TRINITY_DN17990_c0_g2_i2 | probable kinetochore SPC25 | 1183 | 3.52E-117 | 69.4 |
| TRINITY_DN17990_c0_g2_i3 | probable kinetochore SPC25 | 1556 | 4.64E-115 | 69.2 |
| TRINITY_DN17990_c0_g2_i4 | probable kinetochore SPC25 | 1441 | 6.06E-118 | 67.95 |
| TRINITY_DN17993_c0_g2_i2 | PLANT CADMIUM RESISTANCE 12 | 1199 | 1.57E-119 | 80.65 |
| TRINITY_DN17995_c0_g1_i1 | probable phosphatase 2C 60 isoform X1 | 1485 | 0 | 93.7 |
| TRINITY_DN17995_c0_g1_i2 | probable phosphatase 2C 60 | 1485 | 0 | 94.15 |
| TRINITY_DN17997_c1_g1_i1 | ---NA--- | 311 |  |  |
| TRINITY_DN17998_c0_g1_i1 | transcription factor bHLH93 | 982 | 4.03E-99 | 80.7 |
| TRINITY_DN17998_c0_g2_i1 | rab escort 1 | 906 | 1.87E-53 | 64.65 |
| TRINITY_DN17998_c0_g2_i2 | rab escort 1 | 935 | 4.00E-62 | 68.15 |
| TRINITY_DN17998_c0_g2_i3 | rab escort 1 | 936 | 5.67E-59 | 67.1 |
| TRINITY_DN17998_c0_g2_i4 | rab escort 1 | 907 | 5.24E-51 | 63.15 |
| TRINITY_DN17_c0_g1_i1 | rRNA methyltransferase | 3002 | 0 | 72.65 |
| TRINITY_DN17_c0_g2_i1 | rRNA methyltransferase | 3001 | 0 | 73.65 |
| TRINITY_DN18002_c0_g1_i1 | puromycin-sensitive aminopeptidase isoform X1 | 3325 | 0 | 88.55 |
| TRINITY_DN18002_c0_g1_i2 | puromycin-sensitive aminopeptidase isoform X1 | 3313 | 0 | 88.53 |
| TRINITY_DN18003_c0_g2_i1 | probable phosphatase 2C 12 | 1369 | 8.85E-135 | 88.25 |
| TRINITY_DN18010_c0_g1_i1 | ---NA--- | 677 |  |  |
| TRINITY_DN18010_c0_g1_i4 | DDB1- and CUL4-associated factor 8 isoform X1 | 250 | 1.43E-11 | 77 |
| TRINITY_DN18016_c0_g1_i1 | ---NA--- | 512 |  |  |
| TRINITY_DN18020_c0_g1_i1 | probable ubiquitin-like-specific protease 2A isoform X1 | 1383 | 1.36E-118 | 66 |
| TRINITY_DN18020_c0_g1_i2 | probable ubiquitin-like-specific protease 2A isoform X1 | 1410 | 2.40E-129 | 68.5 |
| TRINITY_DN18025_c0_g1_i1 | ---NA--- | 630 |  |  |
| TRINITY_DN18028_c0_g1_i1 | ---NA--- | 1040 |  |  |
| TRINITY_DN18028_c0_g1_i2 | ---NA--- | 933 |  |  |
| TRINITY_DN18028_c0_g1_i3 | ---NA--- | 958 |  |  |
| TRINITY_DN18031_c0_g2_i1 | peroxisome biogenesis 6 | 2718 | 0 | 77.95 |
| TRINITY_DN18031_c0_g2_i2 | peroxisome biogenesis 6 | 2770 | 0 | 77.35 |
| TRINITY_DN18035_c0_g1_i1 | probable serine threonine- kinase WNK11 | 1171 | 1.24E-166 | 90.45 |
| TRINITY_DN18035_c0_g1_i2 | probable serine threonine- kinase WNK11 | 614 | 9.30E-65 | 92.45 |
| TRINITY_DN18038_c0_g1_i1 | serine threonine- kinase PBS1 | 1977 | 0 | 87.2 |
| TRINITY_DN18038_c0_g2_i1 | cyclin-dependent kinase inhibitor 7-like | 965 | 2.93E-23 | 75.95 |
| TRINITY_DN18044_c0_g1_i1 | LYK5 | 2332 | 0 | 68.65 |
| TRINITY_DN18044_c0_g1_i2 | LYK5 | 2414 | 0 | 68.35 |
| TRINITY_DN18046_c0_g1_i1 | ---NA--- | 280 |  |  |
| TRINITY_DN18046_c0_g2_i1 | probable receptor kinase At4g10390 | 1487 | 4.60E-165 | 71.4 |
| TRINITY_DN18051_c0_g1_i1 | decapping 5 | 1496 | 6.93E-156 | 64.1 |
| TRINITY_DN18052_c0_g1_i1 | probable transcriptional regulator SLK2 isoform X1 | 3218 | 0 | 74 |
| TRINITY_DN18052_c0_g2_i1 | probable transcriptional regulator SLK2 isoform X1 | 3062 | 0 | 74.9 |
| TRINITY_DN18052_c0_g2_i2 | probable transcriptional regulator SLK2 | 3065 | 0 | 75 |
| TRINITY_DN18052_c0_g3_i1 | zinc-finger homeodomain 9-like | 820 | 2.13E-30 | 69.75 |
| TRINITY_DN18052_c0_g4_i1 | zinc-finger homeodomain 9-like | 1551 | 1.15E-65 | 60.21 |
| TRINITY_DN18054_c0_g1_i1 | ubiquitin-conjugating enzyme E2 28-like | 420 | 3.86E-14 | 99.8 |
| TRINITY_DN18054_c2_g1_i1 | ubiquitin-conjugating enzyme E2 28-like | 295 | 1.36E-18 | 99.85 |
| TRINITY_DN18055_c0_g1_i1 | Phox Bem1p | 3139 | 0 | 70.25 |
| TRINITY_DN18055_c0_g1_i2 | NLP6-like isoform X1 | 1111 | 8.12E-139 | 74.6 |
| TRINITY_DN18055_c0_g1_i3 | Phox Bem1p | 3233 | 0 | 72.6 |
| TRINITY_DN18062_c0_g1_i1 | early nodulin 2 | 1265 | 3.24E-49 | 66.65 |
| TRINITY_DN18062_c0_g1_i2 | early nodulin 2 | 706 | 4.13E-55 | 67.6 |
| TRINITY_DN18079_c0_g1_i1 | PREDICTED: uncharacterized protein LOC104889993 | 880 | 1.67E-44 | 68.75 |
| TRINITY_DN18079_c0_g2_i1 | PREDICTED: uncharacterized protein LOC104889993 | 881 | 1.73E-44 | 68.89 |
| TRINITY_DN18079_c1_g1_i1 | senescence-associated family | 1044 | 2.66E-19 | 65.1 |
| TRINITY_DN18079_c1_g1_i2 | senescence-associated family | 1098 | 1.56E-14 | 53.2 |
| TRINITY_DN18079_c2_g1_i1 | ---NA--- | 246 |  |  |
| TRINITY_DN18084_c0_g1_i1 | septum-promoting GTP-binding 1 | 1216 | 6.17E-136 | 78.35 |
| TRINITY_DN18088_c0_g1_i1 | 28 kDa chloroplastic | 380 | 1.13E-10 | 61.5 |
| TRINITY_DN18088_c1_g1_i1 | PREDICTED: uncharacterized protein LOC104115157 | 1451 | 1.82E-45 | 82.6 |
| TRINITY_DN18088_c1_g1_i2 | PREDICTED: uncharacterized protein LOC104115157 | 1454 | 3.23E-49 | 82.63 |
| TRINITY_DN18088_c1_g1_i3 | PREDICTED: uncharacterized protein LOC104115157 | 952 | 3.10E-47 | 82.9 |
| TRINITY_DN18088_c1_g1_i4 | unnamed protein product | 1457 | 1.66E-45 | 82.45 |
| TRINITY_DN18093_c0_g1_i1 | calvin cycle CP12- chloroplastic-like | 704 | 9.29E-38 | 70.9 |
| TRINITY_DN18093_c0_g1_i2 | calvin cycle CP12- chloroplastic-like | 522 | 1.27E-38 | 70.9 |
| TRINITY_DN18096_c1_g1_i2 | shaggy-related kinase eta isoform X1 | 1818 | 0 | 94.8 |
| TRINITY_DN18096_c1_g1_i4 | shaggy-related kinase eta-like | 1729 | 0 | 94.9 |
| TRINITY_DN18099_c0_g1_i1 | Peptidoglycan-binding domain-containing | 922 | 3.08E-36 | 71.35 |
| TRINITY_DN18106_c0_g3_i1 | probable transcription factor 21 | 1627 | 1.65E-136 | 76.75 |
| TRINITY_DN18111_c0_g1_i1 | peptidyl-prolyl cis-trans isomerase CYP21-1 | 1170 | 4.74E-116 | 86.85 |
| TRINITY_DN18112_c0_g1_i1 | PREDICTED: uncharacterized protein LOC104898794 | 1008 | 5.04E-15 | 60.25 |
| TRINITY_DN18113_c0_g1_i1 | PREDICTED: uncharacterized protein LOC104900026 | 798 | 3.26E-41 | 75.15 |
| TRINITY_DN18113_c0_g1_i2 | PREDICTED: uncharacterized protein LOC104900026 | 824 | 1.75E-41 | 76.6 |
| TRINITY_DN18113_c1_g1_i1 | U4 small nuclear ribonucleo 27 kDa | 1010 | 1.18E-23 | 73.35 |
| TRINITY_DN18117_c0_g1_i1 | EXGT1 family | 1261 | 0 | 93.4 |
| TRINITY_DN18117_c0_g2_i1 | probable xyloglucan endotransglucosylase hydrolase 5 | 491 | 3.24E-46 | 91.95 |
| TRINITY_DN1812_c0_g1_i1 | hypothetical protein SOVF_001020 | 238 | 1.26E-14 | 67.33 |
| TRINITY_DN18131_c0_g1_i1 | alpha beta-gliadin A-III | 2190 | 1.83E-97 | 65.35 |
| TRINITY_DN18131_c0_g1_i4 | alpha beta-gliadin A-III | 1494 | 3.11E-100 | 65.35 |
| TRINITY_DN18131_c0_g2_i1 | SWIB MDM2 domain | 651 | 2.73E-53 | 54.95 |
| TRINITY_DN18137_c0_g1_i1 | E3 ubiquitin- ligase XBAT31 | 2016 | 0 | 81.4 |
| TRINITY_DN18138_c0_g1_i1 | mitochondrial Rho GTPase 1-like | 996 | 3.34E-90 | 73.05 |
| TRINITY_DN18138_c0_g2_i1 | mitochondrial Rho GTPase 1-like | 996 | 1.16E-90 | 72.9 |
| TRINITY_DN18143_c0_g1_i1 | zinc finger CCCH domain-containing 18 isoform X1 | 2356 | 0 | 70.1 |
| TRINITY_DN18143_c0_g1_i4 | zinc finger CCCH domain-containing 18 isoform X1 | 2406 | 0 | 70.1 |
| TRINITY_DN18163_c0_g2_i1 | microtubule-associated 70-2 | 1155 | 1.78E-38 | 85.9 |
| TRINITY_DN18163_c0_g2_i2 | microtubule-associated 70-3-like [Tarenaya hassleriana] | 1324 | 1.40E-39 | 81.85 |
| TRINITY_DN18163_c0_g2_i3 | microtubule-associated 70-2 | 1182 | 2.16E-38 | 85.9 |
| TRINITY_DN18181_c0_g1_i2 | uncharacterized CRM domain-containing chloroplastic-like | 1558 | 1.75E-118 | 80.3 |
| TRINITY_DN18191_c0_g1_i1 | calpain-type cysteine protease DEK1 | 5608 | 0 | 87.8 |
| TRINITY_DN18191_c0_g2_i1 | calpain-type cysteine protease DEK1 | 7199 | 0 | 89.25 |
| TRINITY_DN18192_c0_g1_i2 | cell number regulator 1-like | 884 | 8.35E-84 | 78.65 |
| TRINITY_DN18199_c0_g1_i1 | Tetratricopeptide-like helical | 1332 | 8.49E-150 | 73.45 |
| TRINITY_DN1819_c0_g1_i1 | basic 7S globulin | 572 | 2.58E-38 | 60.8 |
| TRINITY_DN18207_c0_g1_i1 | trihelix transcription factor ASIL2 | 1089 | 1.22E-48 | 82.75 |
| TRINITY_DN18207_c0_g2_i1 | trihelix transcription factor ASIL2 | 1089 | 8.91E-49 | 82.75 |
| TRINITY_DN18209_c0_g2_i1 | ---NA--- | 808 |  |  |
| TRINITY_DN18209_c1_g1_i1 | ---NA--- | 566 |  |  |
| TRINITY_DN18209_c1_g1_i2 | ---NA--- | 581 |  |  |
| TRINITY_DN18225_c0_g1_i1 | SIP5-like isoform X1 | 1888 | 2.74E-141 | 69.2 |
| TRINITY_DN18226_c0_g1_i2 | probable ribose-5-phosphate isomerase 2 | 1219 | 1.10E-144 | 90.25 |
| TRINITY_DN18238_c0_g1_i1 | ---NA--- | 340 |  |  |
| TRINITY_DN18241_c1_g1_i2 | probable methyltransferase PMT20 | 2435 | 0 | 87.45 |
| TRINITY_DN18241_c1_g1_i3 | probable methyltransferase PMT20 | 2435 | 0 | 86.95 |
| TRINITY_DN18242_c0_g1_i1 | LRR receptor-like serine threonine- kinase ERL1 | 1356 | 0 | 83.7 |
| TRINITY_DN18242_c1_g1_i1 | ---NA--- | 514 |  |  |
| TRINITY_DN18242_c1_g1_i2 | ---NA--- | 361 |  |  |
| TRINITY_DN18250_c0_g1_i1 | thiamine biosynthesis oxidoreductase | 1735 | 0 | 77.4 |
| TRINITY_DN18250_c0_g1_i2 | hypothetical protein BVRB_8g192520 | 1809 | 1.60E-144 | 78.8 |
| TRINITY_DN18256_c0_g1_i1 | E3 ubiquitin- ligase BOI-like | 1049 | 1.47E-87 | 70 |
| TRINITY_DN18257_c0_g1_i1 | ---NA--- | 270 |  |  |
| TRINITY_DN18257_c0_g2_i1 | ---NA--- | 273 |  |  |
| TRINITY_DN18257_c1_g2_i1 | trihelix transcription factor ASR3 | 1314 | 4.26E-93 | 61.6 |
| TRINITY_DN18257_c1_g2_i2 | trihelix transcription factor ASR3 | 1257 | 6.38E-94 | 64.05 |
| TRINITY_DN18259_c0_g1_i1 | transcription factor MYB1R1 | 1353 | 6.64E-106 | 66.55 |
| TRINITY_DN18265_c0_g1_i1 | ---NA--- | 1119 |  |  |
| TRINITY_DN18273_c0_g1_i1 | probable trehalose-phosphate phosphatase J | 1539 | 2.28E-176 | 76.4 |
| TRINITY_DN18276_c0_g2_i1 | hypothetical protein SOVF_079490 | 1588 | 1.37E-34 | 85.55 |
| TRINITY_DN18276_c0_g2_i2 | hypothetical protein SOVF_079490 | 1052 | 6.33E-36 | 85.55 |
| TRINITY_DN18276_c0_g2_i3 | corepressor interacting with RBPJ 1 isoform X1 | 1614 | 2.64E-51 | 76.85 |
| TRINITY_DN18276_c0_g2_i4 | corepressor interacting with RBPJ 1 isoform X1 | 1078 | 1.67E-57 | 76.85 |
| TRINITY_DN18277_c0_g2_i1 | peroxygenase | 1078 | 2.38E-151 | 85.25 |
| TRINITY_DN18279_c0_g1_i1 | suppressor of mec-8 and unc-52 homolog 1 | 1859 | 0 | 95.25 |
| TRINITY_DN18292_c0_g1_i2 | probable acyl-activating enzyme peroxisomal | 1873 | 0 | 81.3 |
| TRINITY_DN18292_c0_g1_i3 | probable acyl-activating enzyme peroxisomal | 1122 | 8.81E-141 | 77.9 |
| TRINITY_DN18292_c0_g1_i4 | probable acyl-activating enzyme peroxisomal | 2522 | 0 | 81.65 |
| TRINITY_DN18298_c0_g1_i1 | transcription factor LHW-like | 1352 | 0 | 76.35 |
| TRINITY_DN18298_c0_g1_i4 | basic helix-loop-helix | 1201 | 1.11E-71 | 82.65 |
| TRINITY_DN18300_c0_g1_i2 | PREDICTED: uncharacterized protein LOC104899180 | 1134 | 5.09E-56 | 64.4 |
| TRINITY_DN18303_c0_g1_i1 | SAWADEE HOMEODOMAIN HOMOLOG 2 | 1575 | 1.75E-108 | 82.75 |
| TRINITY_DN18304_c0_g2_i1 | Zinc U1-type | 1766 | 4.09E-96 | 54.25 |
| TRINITY_DN18304_c0_g3_i1 | Zinc U1-type | 2026 | 6.89E-90 | 54.6 |
| TRINITY_DN18305_c0_g1_i1 | serine threonine phosphatase 2A 57 kDa regulatory subunit B iota isoform | 2393 | 0 | 85.15 |
| TRINITY_DN18305_c0_g1_i2 | serine threonine phosphatase 2A 57 kDa regulatory subunit B iota isoform | 666 | 1.90E-35 | 78.35 |
| TRINITY_DN18305_c0_g2_i1 | ---NA--- | 220 |  |  |
| TRINITY_DN18311_c0_g1_i1 | glucose-1-phosphate adenylyltransferase small subunit chloroplastic | 4827 | 0 | 92.6 |
| TRINITY_DN18312_c0_g1_i1 | deSI At4g17486 isoform X1 | 513 | 6.13E-06 | 96 |
| TRINITY_DN18312_c0_g2_i1 | deSI At4g17486 isoform X2 | 1208 | 6.81E-109 | 84.2 |
| TRINITY_DN18314_c0_g1_i1 | PREDICTED: uncharacterized protein LOC104883003 | 821 | 8.63E-37 | 56.55 |
| TRINITY_DN18314_c0_g1_i2 | PREDICTED: uncharacterized protein LOC104883003 | 862 | 6.26E-34 | 53 |
| TRINITY_DN18315_c0_g1_i1 | arginine decarboxylase | 2915 | 0 | 80.5 |
| TRINITY_DN18327_c0_g3_i1 | ---NA--- | 468 |  |  |
| TRINITY_DN18337_c0_g1_i1 | syntaxin-32 | 1234 | 2.65E-129 | 82.45 |
| TRINITY_DN18342_c0_g2_i1 | RNA polymerase II degradation factor 1 | 939 | 7.11E-27 | 62.25 |
| TRINITY_DN18344_c0_g1_i1 | Glycosyl family 14 | 1931 | 5.27E-127 | 88.9 |
| TRINITY_DN18344_c0_g1_i2 | Glycosyl family 14 | 1962 | 2.08E-166 | 84.55 |
| TRINITY_DN18348_c0_g2_i1 | 30-kDa cleavage and polyadenylation specificity factor 30 | 2313 | 0 | 76.6 |
| TRINITY_DN18352_c0_g1_i1 | NLRC3 isoform X1 | 2168 | 0 | 84.8 |
| TRINITY_DN18352_c0_g1_i2 | NLRC3 isoform X1 | 2326 | 0 | 84.2 |
| TRINITY_DN18368_c0_g1_i1 | isovaleryl- mitochondrial | 1576 | 0 | 93.45 |
| TRINITY_DN18373_c0_g1_i1 | transmembrane 64 | 1528 | 7.39E-128 | 82.2 |
| TRINITY_DN18374_c0_g2_i1 | dynamin-related 3A-like | 2423 | 0 | 81.8 |
| TRINITY_DN18382_c0_g2_i1 | ---NA--- | 211 |  |  |
| TRINITY_DN18389_c0_g1_i1 | general transcription factor 3C polypeptide 5-like | 2127 | 0 | 65.85 |
| TRINITY_DN18389_c0_g1_i2 | general transcription factor 3C polypeptide 5-like | 2173 | 0 | 69.3 |
| TRINITY_DN18389_c0_g1_i3 | general transcription factor 3C polypeptide 5-like | 2085 | 0 | 65.9 |
| TRINITY_DN18389_c0_g1_i4 | general transcription factor 3C polypeptide 5-like | 2131 | 0 | 69.4 |
| TRINITY_DN18394_c0_g2_i1 | endoplasmic reticulum-Golgi intermediate compartment 3-like | 1508 | 0 | 92.95 |
| TRINITY_DN18409_c0_g2_i1 | phosphatidylinositol glycan anchor biosynthesis class U | 1941 | 0 | 79.85 |
| TRINITY_DN18409_c0_g2_i2 | phosphatidylinositol glycan anchor biosynthesis class U | 1921 | 0 | 79.9 |
| TRINITY_DN18418_c0_g1_i1 | hypothetical protein SOVF_004900 | 1644 | 0 | 78.3 |
| TRINITY_DN18419_c0_g1_i1 | transcription repressor OFP8-like | 1292 | 1.08E-39 | 86.55 |
| TRINITY_DN18422_c0_g1_i1 | pentatricopeptide repeat-containing chloroplastic | 2960 | 0 | 81.35 |
| TRINITY_DN18428_c0_g1_i2 | Fiber Fb34 | 970 | 1.03E-65 | 82.25 |
| TRINITY_DN18428_c0_g1_i3 | Fiber Fb34 | 986 | 8.69E-81 | 80.85 |
| TRINITY_DN1842_c0_g1_i1 | thylakoid lumenal 15 kDa chloroplastic | 883 | 4.69E-96 | 94.1 |
| TRINITY_DN1842_c0_g2_i1 | thylakoid lumenal 15 kDa chloroplastic | 886 | 4.51E-96 | 94.1 |
| TRINITY_DN18430_c0_g1_i1 | RNA pseudouridine synthase 1 | 1254 | 1.79E-97 | 80.6 |
| TRINITY_DN18430_c0_g1_i5 | RNA pseudouridine synthase 1 | 1468 | 8.12E-89 | 78.6 |
| TRINITY_DN18430_c0_g1_i6 | RNA pseudouridine synthase 1 | 1544 | 2.51E-122 | 77.8 |
| TRINITY_DN18436_c1_g1_i1 | ---NA--- | 304 |  |  |
| TRINITY_DN18439_c0_g1_i1 | kinase dsk1 | 1999 | 0 | 83 |
| TRINITY_DN18439_c0_g1_i2 | kinase dsk1 | 2120 | 0 | 83 |
| TRINITY_DN1843_c0_g1_i1 | trihelix transcription factor GT-2 | 317 | 1.02E-16 | 86.45 |
| TRINITY_DN18441_c0_g2_i1 | type 2C phosphatase | 290 | 9.51E-12 | 82 |
| TRINITY_DN18441_c0_g3_i1 | ---NA--- | 444 |  |  |
| TRINITY_DN18441_c0_g4_i1 | ---NA--- | 304 |  |  |
| TRINITY_DN18452_c0_g1_i2 | mitochondrial inner membrane protease subunit 1 | 768 | 2.05E-81 | 78.6 |
| TRINITY_DN18456_c0_g1_i1 | proton pump-interactor 1-like | 2315 | 2.80E-158 | 68.85 |
| TRINITY_DN18460_c0_g2_i1 | probable membrane-associated kinase regulator 2 | 1287 | 2.96E-60 | 49.35 |
| TRINITY_DN18464_c0_g1_i1 | SODIUM POTASSIUM ROOT DEFECTIVE 2-like | 1259 | 5.92E-74 | 59.25 |
| TRINITY_DN18468_c0_g1_i1 | UDP-glucuronic acid decarboxylase 6-like | 1177 | 3.80E-12 | 80.1 |
| TRINITY_DN18468_c0_g1_i2 | UDP-glucuronic acid decarboxylase 6-like | 1148 | 4.21E-12 | 78.05 |
| TRINITY_DN18468_c0_g1_i5 | UDP-glucuronic acid decarboxylase 6-like | 1151 | 3.66E-12 | 80.1 |
| TRINITY_DN18468_c0_g1_i6 | UDP-glucuronic acid decarboxylase 6-like | 1122 | 3.94E-12 | 78.05 |
| TRINITY_DN18474_c0_g1_i1 | ---NA--- | 878 |  |  |
| TRINITY_DN18480_c0_g2_i3 | Ankyrin repeat domain-containing 50 isoform 3 | 816 | 7.76E-79 | 79.8 |
| TRINITY_DN18480_c0_g2_i4 | Ankyrin repeat domain-containing 50 isoform 3 | 854 | 2.50E-66 | 75.3 |
| TRINITY_DN18486_c0_g1_i1 | DNA-directed RNA polymerase II subunit RPB2 | 4085 | 0 | 97.45 |
| TRINITY_DN18488_c0_g1_i1 | probable calcium-binding CML41 | 764 | 6.23E-84 | 76.45 |
| TRINITY_DN18488_c0_g1_i2 | probable calcium-binding CML41 | 749 | 7.42E-81 | 77.1 |
| TRINITY_DN184_c0_g2_i1 | transport Sec24-like At3g07100 | 582 | 1.65E-07 | 90.4 |
| TRINITY_DN18509_c0_g1_i1 | PREDICTED: uncharacterized protein LOC104896639 | 1121 | 1.44E-143 | 71.95 |
| TRINITY_DN18509_c0_g2_i1 | PREDICTED: uncharacterized protein LOC104896639 | 1293 | 8.90E-171 | 72.9 |
| TRINITY_DN18524_c0_g1_i1 | probable glycosyltransferase At5g03795 | 1787 | 0 | 93.95 |
| TRINITY_DN18524_c0_g2_i1 | ---NA--- | 441 |  |  |
| TRINITY_DN18525_c0_g1_i1 | GDSL esterase lipase 5 | 1314 | 1.61E-168 | 73.6 |
| TRINITY_DN18525_c0_g1_i3 | GDSL esterase lipase 5 | 1273 | 1.04E-122 | 72.4 |
| TRINITY_DN18533_c0_g1_i1 | 5-methyltetrahydropteroyltriglutamate--homocysteine methyltransferase | 2875 | 0 | 94.4 |
| TRINITY_DN18533_c0_g1_i2 | 5-methyltetrahydropteroyltriglutamate--homocysteine methyltransferase | 2791 | 0 | 94.25 |
| TRINITY_DN18535_c0_g1_i1 | ---NA--- | 1299 |  |  |
| TRINITY_DN18535_c0_g1_i2 | ---NA--- | 734 |  |  |
| TRINITY_DN18540_c0_g1_i1 | hypothetical protein glysoja_028539 | 1727 | 5.71E-114 | 82 |
| TRINITY_DN18540_c0_g1_i2 | hypothetical protein glysoja_028539 | 2011 | 6.87E-114 | 82 |
| TRINITY_DN18540_c0_g1_i3 | Zinc knuckle family isoform 1 | 1620 | 0 | 76.35 |
| TRINITY_DN18540_c0_g1_i4 | hypothetical protein glysoja_028539 | 1832 | 6.13E-114 | 82 |
| TRINITY_DN18540_c0_g1_i5 | Zinc knuckle family isoform 1 | 1799 | 0 | 76.35 |
| TRINITY_DN18540_c0_g1_i6 | Zinc knuckle family isoform 1 | 1515 | 0 | 76.35 |
| TRINITY_DN18543_c0_g1_i1 | MID1-COMPLEMENTING ACTIVITY 1-like | 526 | 1.28E-57 | 95.4 |
| TRINITY_DN18546_c0_g1_i1 | ---NA--- | 401 |  |  |
| TRINITY_DN1854_c0_g1_i1 | cysteine ase | 1401 | 0 | 86.75 |
| TRINITY_DN18553_c0_g2_i1 | zinc finger CCCH domain-containing 62 | 1616 | 1.79E-118 | 73.05 |
| TRINITY_DN18563_c0_g1_i1 | histone-lysine N-methyltransferase CLF isoform X1 | 2062 | 0 | 77.75 |
| TRINITY_DN18563_c0_g2_i1 | Histone-lysine N-methyltransferase CLF | 382 | 6.59E-32 | 92.2 |
| TRINITY_DN18563_c1_g1_i1 | ---NA--- | 268 |  |  |
| TRINITY_DN18569_c0_g1_i1 | 5 -3 exoribonuclease 3-like isoform X2 | 2063 | 0 | 71.85 |
| TRINITY_DN1856_c0_g1_i1 | PREDICTED: uncharacterized protein LOC104888374 | 250 | 3.63E-06 | 97.5 |
| TRINITY_DN1856_c0_g2_i1 | ---NA--- | 433 |  |  |
| TRINITY_DN18573_c1_g1_i2 | WRKY transcription factor 22 | 361 | 4.62E-44 | 91.65 |
| TRINITY_DN18573_c1_g2_i1 | WRKY transcription factor 22-like | 836 | 5.79E-80 | 71.6 |
| TRINITY_DN18578_c0_g1_i1 | NAC domain-containing 2-like | 1282 | 2.45E-176 | 78.25 |
| TRINITY_DN18578_c0_g1_i2 | NAC domain-containing 2-like | 1086 | 8.43E-153 | 74.95 |
| TRINITY_DN18578_c0_g2_i1 | NAC domain-containing 2-like | 630 | 3.83E-103 | 94.25 |
| TRINITY_DN18585_c0_g1_i1 | MALE DISCOVERER 1-like isoform X1 | 2767 | 0 | 67.3 |
| TRINITY_DN18589_c0_g1_i1 | glyoxylate hydroxypyruvate reductase HPR3-like | 1392 | 3.18E-142 | 74.8 |
| TRINITY_DN18590_c0_g1_i1 | U2 small nuclear ribonucleo B isoform X1 | 1463 | 1.23E-105 | 73.35 |
| TRINITY_DN18600_c0_g1_i1 | glycine-rich RNA-binding mitochondrial-like | 840 | 4.74E-55 | 82.1 |
| TRINITY_DN18601_c0_g1_i1 | molybdenum cofactor sulfurase | 1108 | 1.46E-140 | 72.15 |
| TRINITY_DN18602_c0_g1_i1 | Phospholipid:diacylglycerol acyltransferase 1 | 679 | 9.14E-07 | 68.57 |
| TRINITY_DN18602_c0_g1_i2 | Phospholipid:diacylglycerol acyltransferase 1 | 548 | 5.50E-07 | 70.05 |
| TRINITY_DN18602_c0_g2_i1 | ---NA--- | 245 |  |  |
| TRINITY_DN18618_c0_g1_i1 | DNA-directed RNA polymerase II subunit 1 | 5069 | 0 | 94.25 |
| TRINITY_DN18620_c0_g1_i1 | ---NA--- | 335 |  |  |
| TRINITY_DN18620_c0_g2_i1 | ---NA--- | 343 |  |  |
| TRINITY_DN18627_c0_g1_i2 | hypothetical protein BVRB_6g128440 | 800 | 3.43E-16 | 79.65 |
| TRINITY_DN18634_c0_g1_i1 | PREDICTED: uncharacterized protein LOC104888860 isoform X3 | 890 | 5.47E-101 | 83.05 |
| TRINITY_DN18634_c0_g1_i2 | ZZ-type zinc finger-containing 3 | 933 | 3.65E-77 | 78.1 |
| TRINITY_DN18634_c0_g1_i3 | histone H2A deubiquitinase (DUF3755) | 348 | 2.08E-11 | 89.9 |
| TRINITY_DN18634_c1_g1_i1 | serine threonine- kinase CDL1 | 2355 | 0 | 83.85 |
| TRINITY_DN18638_c0_g1_i1 | ferredoxin-dependent glutamate chloroplastic | 1734 | 6.61E-118 | 82.4 |
| TRINITY_DN18638_c0_g2_i1 | ferredoxin-dependent glutamate chloroplastic isoform X1 | 1735 | 8.26E-73 | 84.05 |
| TRINITY_DN18639_c0_g1_i1 | Myosin-H heavy chain isoform 1 | 1405 | 4.57E-142 | 79.3 |
| TRINITY_DN18639_c0_g1_i2 | Myosin-H heavy chain isoform 1 | 1366 | 5.25E-129 | 79.4 |
| TRINITY_DN18641_c0_g1_i1 | UTP--glucose-1-phosphate uridylyltransferase chloroplastic | 3229 | 0 | 80.8 |
| TRINITY_DN18641_c0_g1_i2 | UTP--glucose-1-phosphate uridylyltransferase chloroplastic | 3232 | 0 | 80.75 |
| TRINITY_DN18646_c0_g2_i1 | myb X isoform X1 | 2943 | 1.51E-15 | 62.3 |
| TRINITY_DN18646_c0_g2_i2 | myb X isoform X1 | 2962 | 1.52E-15 | 62.3 |
| TRINITY_DN18648_c0_g1_i1 | ---NA--- | 461 |  |  |
| TRINITY_DN18648_c0_g3_i1 | ---NA--- | 449 |  |  |
| TRINITY_DN18648_c0_g4_i1 | ---NA--- | 331 |  |  |
| TRINITY_DN18658_c0_g1_i1 | auxin transporter 4 | 1835 | 0 | 93.9 |
| TRINITY_DN18660_c0_g1_i1 | ABC transporter B family member 20 | 4752 | 0 | 91.65 |
| TRINITY_DN18675_c0_g1_i1 | bifunctional epoxide hydrolase 2-like | 1736 | 0 | 76.6 |
| TRINITY_DN18677_c0_g2_i1 | AMSH-like ubiquitin thioesterase 3 | 2055 | 0 | 72.95 |
| TRINITY_DN18677_c0_g2_i2 | AMSH-like ubiquitin thioesterase 3 | 2086 | 0 | 75.5 |
| TRINITY_DN18677_c1_g1_i1 | ---NA--- | 314 |  |  |
| TRINITY_DN18689_c0_g1_i1 | transcriptional corepressor SEUSS | 1242 | 3.19E-49 | 63.25 |
| TRINITY_DN18689_c0_g2_i1 | transcriptional corepressor SEUSS | 1274 | 3.33E-42 | 68.1 |
| TRINITY_DN18696_c0_g1_i1 | probable carboxylesterase 11 | 1777 | 0 | 78 |
| TRINITY_DN18696_c0_g1_i2 | probable carboxylesterase 11 | 1736 | 5.14E-174 | 97.15 |
| TRINITY_DN18696_c1_g1_i1 | probable E3 ubiquitin- ligase rbrA | 1944 | 0 | 77.65 |
| TRINITY_DN18696_c2_g1_i1 | ---NA--- | 201 |  |  |
| TRINITY_DN18697_c0_g1_i1 | phospholipid-transporting ATPase 2 | 359 | 5.24E-11 | 81.2 |
| TRINITY_DN18697_c0_g2_i1 | phospholipid-transporting ATPase 2 | 3616 | 0 | 92.7 |
| TRINITY_DN18699_c0_g1_i1 | ---NA--- | 446 |  |  |
| TRINITY_DN18699_c0_g2_i1 | probable apyrase 7 | 2977 | 0 | 76.05 |
| TRINITY_DN18699_c0_g2_i2 | probable apyrase 7 | 2789 | 0 | 76.05 |
| TRINITY_DN18700_c0_g2_i1 | ---NA--- | 735 |  |  |
| TRINITY_DN18700_c0_g3_i1 | iron-sulfur assembly -like mitochondrial | 904 | 1.02E-66 | 94.4 |
| TRINITY_DN18709_c0_g1_i2 | SPX domain-containing 3 | 2073 | 3.78E-129 | 82.35 |
| TRINITY_DN18717_c0_g2_i1 | scarecrow 15 | 1475 | 0 | 71.7 |
| TRINITY_DN18718_c0_g1_i1 | Aquaporin PIP1- | 1257 | 0 | 95 |
| TRINITY_DN18718_c0_g1_i3 | probable aquaporin PIP1-4 | 1569 | 0 | 95.1 |
| TRINITY_DN18719_c0_g1_i2 | PREDICTED: uncharacterized protein LOC104902199 | 792 | 5.43E-60 | 52.4 |
| TRINITY_DN18730_c0_g1_i1 | probable beta-1,4-xylosyltransferase IRX14 | 1988 | 0 | 76.85 |
| TRINITY_DN18732_c0_g2_i1 | ---NA--- | 233 |  |  |
| TRINITY_DN18732_c1_g2_i1 | cytochrome c | 701 | 3.72E-68 | 94.4 |
| TRINITY_DN18732_c1_g2_i2 | cytochrome c | 879 | 1.61E-62 | 94.4 |
| TRINITY_DN18738_c1_g1_i1 | FAR-RED ELONGATED HYPOCOTYL 3-like | 921 | 1.26E-33 | 53.45 |
| TRINITY_DN18738_c1_g2_i1 | FAR-RED ELONGATED HYPOCOTYL 3-like | 948 | 1.47E-33 | 53.4 |
| TRINITY_DN18740_c0_g1_i1 | PREDICTED: uncharacterized protein LOC104906018 | 1083 | 1.39E-68 | 67.45 |
| TRINITY_DN18751_c1_g4_i1 | ---NA--- | 504 |  |  |
| TRINITY_DN18751_c1_g7_i1 | haloacid dehalogenase-like hydrolase domain-containing At2g33255 | 1385 | 1.48E-144 | 89 |
| TRINITY_DN18752_c0_g1_i1 | probable ATP-dependent DNA helicase CHR12 | 3286 | 0 | 86.35 |
| TRINITY_DN18761_c0_g2_i1 | chromo domain-containing LHP1 | 1409 | 5.55E-136 | 61.35 |
| TRINITY_DN18769_c0_g1_i1 | NAC domain-containing 2-like | 1571 | 1.40E-178 | 78.55 |
| TRINITY_DN18775_c0_g1_i2 | flowering time control FCA isoform X1 | 2022 | 1.09E-138 | 55.4 |
| TRINITY_DN18777_c0_g1_i2 | sigma non-opioid intracellular receptor 1 | 470 | 9.74E-20 | 58.3 |
| TRINITY_DN18782_c1_g1_i1 | ubiquitin domain-containing DSK2a-like isoform X1 | 2258 | 0 | 72.7 |
| TRINITY_DN18787_c1_g1_i1 | leucine-rich repeat-containing DDB_G0290503 isoform X2 | 2965 | 0 | 63.05 |
| TRINITY_DN18787_c1_g1_i2 | myosin-11-like isoform X1 | 3329 | 0 | 66.5 |
| TRINITY_DN18787_c1_g1_i3 | myosin-11-like isoform X1 | 3688 | 0 | 67.05 |
| TRINITY_DN18787_c1_g1_i4 | transport family | 690 | 2.25E-57 | 62.8 |
| TRINITY_DN18789_c0_g1_i1 | hydroxyphenylpyruvate reductase | 1760 | 0 | 88.9 |
| TRINITY_DN1879_c0_g1_i1 | ---NA--- | 238 |  |  |
| TRINITY_DN18800_c0_g1_i1 | PREDICTED: uncharacterized protein LOC104889952 | 2662 | 0 | 81.35 |
| TRINITY_DN18803_c0_g1_i1 | WD repeat domain-containing 83 | 1424 | 0 | 92.4 |
| TRINITY_DN18804_c0_g1_i1 | f-box family | 907 | 1.36E-37 | 54.93 |
| TRINITY_DN18804_c0_g2_i1 | F-box At1g49610 | 501 | 3.81E-11 | 77.86 |
| TRINITY_DN18804_c0_g2_i2 | F-box At1g49610 | 520 | 4.47E-11 | 78 |
| TRINITY_DN18804_c0_g5_i1 | F-box At1g49610 | 1861 | 2.33E-101 | 51.65 |
| TRINITY_DN18819_c0_g1_i1 | DNA polymerase beta-like isoform X1 | 2310 | 3.31E-152 | 65.1 |
| TRINITY_DN18819_c0_g1_i2 | DNA polymerase beta isoform X1 | 2200 | 0 | 68.25 |
| TRINITY_DN18820_c0_g1_i1 | rho GDP-dissociation inhibitor 1 | 890 | 4.62E-98 | 92.3 |
| TRINITY_DN18835_c0_g1_i2 | F-box LRR-repeat At4g14103 isoform X1 | 593 | 4.23E-11 | 55.2 |
| TRINITY_DN18835_c0_g1_i3 | F-box LRR-repeat At4g14103 isoform X1 | 872 | 1.73E-20 | 48.88 |
| TRINITY_DN18837_c0_g1_i1 | DNA-directed RNA polymerases IV and V subunit 3 | 800 | 1.55E-95 | 93.4 |
| TRINITY_DN18837_c0_g2_i1 | DNA-directed RNA polymerases IV and V subunit 3 | 1356 | 0 | 92.7 |
| TRINITY_DN18837_c0_g2_i2 | DNA-directed RNA polymerases IV and V subunit 3 | 1410 | 0 | 92.55 |
| TRINITY_DN18838_c0_g1_i1 | hypothetical protein SOVF_037520 | 539 | 2.47E-83 | 88.95 |
| TRINITY_DN18842_c0_g1_i1 | S-acyltransferase 24 | 2655 | 0 | 85.3 |
| TRINITY_DN18842_c0_g1_i2 | S-acyltransferase 24 | 2676 | 0 | 84.65 |
| TRINITY_DN1884_c0_g1_i1 | ---NA--- | 311 |  |  |
| TRINITY_DN1884_c0_g2_i1 | ---NA--- | 311 |  |  |
| TRINITY_DN18855_c0_g2_i3 | zinc finger CONSTANS-LIKE 7 isoform X1 | 1407 | 2.16E-125 | 71.95 |
| TRINITY_DN18855_c0_g2_i4 | zinc finger CONSTANS-LIKE 7 isoform X1 | 1455 | 6.26E-132 | 74.95 |
| TRINITY_DN18863_c0_g1_i1 | F-box At1g49610 isoform X1 | 530 | 1.77E-19 | 64.67 |
| TRINITY_DN18868_c0_g1_i1 | ---NA--- | 290 |  |  |
| TRINITY_DN18868_c0_g2_i1 | ---NA--- | 290 |  |  |
| TRINITY_DN18868_c0_g3_i1 | ---NA--- | 290 |  |  |
| TRINITY_DN18868_c0_g4_i1 | ---NA--- | 290 |  |  |
| TRINITY_DN1886_c0_g1_i1 | NUCLEAR FUSION DEFECTIVE 2 | 916 | 6.97E-86 | 78.8 |
| TRINITY_DN18874_c0_g1_i1 | ---NA--- | 591 |  |  |
| TRINITY_DN18874_c0_g1_i2 | ---NA--- | 1100 |  |  |
| TRINITY_DN18874_c0_g1_i3 | ---NA--- | 520 |  |  |
| TRINITY_DN18874_c0_g1_i4 | ---NA--- | 1171 |  |  |
| TRINITY_DN18874_c0_g1_i5 | ---NA--- | 535 |  |  |
| TRINITY_DN18874_c0_g1_i6 | ---NA--- | 1115 |  |  |
| TRINITY_DN18874_c0_g2_i1 | ---NA--- | 751 |  |  |
| TRINITY_DN18878_c0_g1_i1 | dihydroxy-acid chloroplastic | 965 | 6.06E-159 | 93.1 |
| TRINITY_DN18882_c0_g1_i1 | transcription factor BIM2-like isoform X1 | 1181 | 1.05E-111 | 63.4 |
| TRINITY_DN18882_c0_g1_i3 | transcription factor BIM2 isoform X2 | 518 | 1.83E-35 | 88.05 |
| TRINITY_DN18882_c0_g1_i4 | transcription factor BIM2 isoform X1 | 533 | 3.02E-15 | 66.5 |
| TRINITY_DN18883_c0_g1_i1 | rho GTPase-activating gacHH isoform X1 | 3058 | 0 | 73.7 |
| TRINITY_DN18883_c0_g1_i2 | rho GTPase-activating gacHH isoform X1 | 3057 | 0 | 73.7 |
| TRINITY_DN18889_c0_g1_i1 | probable carboxylesterase 2 | 1253 | 9.62E-140 | 72.4 |
| TRINITY_DN18892_c0_g1_i2 | carbonic anhydrase 2 isoform X1 | 1143 | 2.37E-145 | 85.25 |
| TRINITY_DN18901_c0_g1_i1 | serine arginine-rich-splicing factor SR34 isoform X2 | 702 | 6.19E-124 | 93.45 |
| TRINITY_DN18901_c0_g1_i2 | serine arginine-rich-splicing factor SR34 isoform X2 | 791 | 1.15E-123 | 93.45 |
| TRINITY_DN18901_c0_g2_i1 | 2,3-dimethylmalate lyase isoform X1 | 1623 | 0 | 81.55 |
| TRINITY_DN18901_c2_g1_i1 | serine arginine-rich-splicing factor SR34 isoform X1 | 211 | 2.20E-26 | 85.55 |
| TRINITY_DN18903_c0_g1_i1 | UDP-glucuronate:xylan alpha-glucuronosyltransferase 3 | 2420 | 0 | 84.1 |
| TRINITY_DN18903_c0_g1_i2 | UDP-glucuronate:xylan alpha-glucuronosyltransferase 3 | 2408 | 0 | 84.1 |
| TRINITY_DN18905_c0_g1_i1 | NAC domain-containing 78-like | 1815 | 0 | 70.9 |
| TRINITY_DN18905_c0_g1_i2 | NAC domain-containing 78-like | 1782 | 0 | 70.9 |
| TRINITY_DN18913_c0_g1_i1 | hypothetical protein SOVF_187600 | 1771 | 1.15E-29 | 44.85 |
| TRINITY_DN18913_c2_g1_i3 | serine threonine phosphatase 2A 57 kDa regulatory subunit B theta isoform-like | 2138 | 0 | 89.75 |
| TRINITY_DN18914_c0_g1_i1 | mitochondrial intermembrane space import and assembly 40 | 1068 | 4.35E-50 | 79.7 |
| TRINITY_DN1891_c0_g1_i1 | ---NA--- | 261 |  |  |
| TRINITY_DN18925_c0_g1_i1 | zinc finger 4-like | 1053 | 4.15E-46 | 58.6 |
| TRINITY_DN18932_c0_g1_i1 | transcription factor PIF1-like isoform X1 | 494 | 8.25E-42 | 84.25 |
| TRINITY_DN18932_c0_g1_i2 | transcription factor PIF1-like isoform X1 | 383 | 7.87E-43 | 84.95 |
| TRINITY_DN18932_c0_g1_i3 | transcription factor PIF1-like isoform X1 | 544 | 4.22E-42 | 85.3 |
| TRINITY_DN18932_c0_g3_i1 | transcription factor PIF1-like | 269 | 4.22E-25 | 85.65 |
| TRINITY_DN18933_c0_g2_i1 | heparanase 1 | 2117 | 0 | 80.95 |
| TRINITY_DN18937_c1_g1_i1 | hypothetical protein POPTR_0001s13670g | 1022 | 1.41E-26 | 71.75 |
| TRINITY_DN18938_c0_g1_i1 | ---NA--- | 1389 |  |  |
| TRINITY_DN18938_c0_g1_i2 | ---NA--- | 1112 |  |  |
| TRINITY_DN18938_c0_g1_i3 | ---NA--- | 997 |  |  |
| TRINITY_DN18938_c0_g1_i4 | ---NA--- | 720 |  |  |
| TRINITY_DN18940_c0_g1_i1 | ---NA--- | 469 |  |  |
| TRINITY_DN18940_c0_g1_i2 | ---NA--- | 406 |  |  |
| TRINITY_DN18940_c0_g2_i1 | ---NA--- | 403 |  |  |
| TRINITY_DN18945_c0_g1_i1 | myb family transcription factor APL isoform X1 | 1406 | 4.83E-150 | 75.55 |
| TRINITY_DN18945_c0_g2_i1 | myb family transcription factor APL isoform X1 | 1409 | 1.30E-151 | 75.1 |
| TRINITY_DN18946_c0_g1_i1 | chloroplastic isoform X1 | 3260 | 0 | 83.1 |
| TRINITY_DN18952_c0_g1_i1 | WAT1-related At4g08300-like | 1556 | 0 | 80.3 |
| TRINITY_DN18953_c0_g1_i1 | ---NA--- | 288 |  |  |
| TRINITY_DN18953_c0_g2_i1 | pentatricopeptide repeat-containing mitochondrial | 468 | 5.56E-18 | 69.25 |
| TRINITY_DN18953_c0_g2_i2 | pentatricopeptide repeat-containing mitochondrial | 482 | 9.08E-27 | 72 |
| TRINITY_DN18953_c0_g2_i3 | pentatricopeptide repeat-containing mitochondrial | 617 | 2.31E-46 | 72.25 |
| TRINITY_DN18957_c0_g2_i1 | ---NA--- | 1201 |  |  |
| TRINITY_DN1895_c0_g1_i1 | H ACA ribonucleo complex subunit 2 | 840 | 4.75E-74 | 91.85 |
| TRINITY_DN1895_c0_g2_i1 | H ACA ribonucleo complex subunit 2 | 839 | 4.71E-89 | 90.15 |
| TRINITY_DN1895_c1_g1_i1 | enhancer of rudimentary homolog | 973 | 5.64E-57 | 90.1 |
| TRINITY_DN18960_c0_g1_i1 | pentatricopeptide repeat-containing At5g02860 | 4003 | 0 | 81.65 |
| TRINITY_DN18960_c0_g2_i1 | 3-oxo-Delta(4,5)-steroid 5-beta-reductase-like | 3802 | 1.02E-174 | 72.2 |
| TRINITY_DN18961_c0_g1_i1 | ERAD-associated E3 ubiquitin- ligase component HRD3A | 2564 | 0 | 87.55 |
| TRINITY_DN18971_c0_g1_i1 | ---NA--- | 283 |  |  |
| TRINITY_DN18971_c0_g2_i1 | mitogen-activated kinase kinase kinase 13 | 904 | 1.01E-104 | 67.9 |
| TRINITY_DN18973_c0_g1_i1 | pentatricopeptide repeat-containing mitochondrial isoform X2 | 2031 | 9.68E-10 | 65 |
| TRINITY_DN18985_c0_g1_i1 | U11 U12 small nuclear ribonucleo 65 kDa | 1658 | 0 | 76.25 |
| TRINITY_DN18985_c0_g1_i3 | RNA recognition motif | 619 | 2.15E-60 | 73.75 |
| TRINITY_DN18985_c0_g1_i5 | U11 U12 small nuclear ribonucleo 65 kDa | 1690 | 2.25E-158 | 77.6 |
| TRINITY_DN18985_c0_g1_i6 | U11 U12 small nuclear ribonucleo 65 kDa | 1635 | 8.12E-154 | 78.2 |
| TRINITY_DN18985_c0_g1_i7 | U11 U12 small nuclear ribonucleo 65 kDa | 1603 | 1.65E-159 | 75.1 |
| TRINITY_DN18985_c0_g1_i8 | U11 U12 small nuclear ribonucleo 65 kDa -like | 587 | 8.61E-81 | 75.95 |
| TRINITY_DN18990_c0_g1_i1 | mitogen-activated kinase kinase 9-like | 1181 | 1.62E-141 | 82.15 |
| TRINITY_DN18993_c0_g1_i1 | 2 -deoxymugineic-acid 2 -dioxygenase | 1127 | 9.32E-91 | 71.95 |
| TRINITY_DN18993_c0_g1_i2 | hyoscyamine 6-dioxygenase-like | 811 | 5.26E-34 | 68.05 |
| TRINITY_DN19004_c0_g1_i1 | ---NA--- | 402 |  |  |
| TRINITY_DN19004_c0_g2_i1 | ---NA--- | 395 |  |  |
| TRINITY_DN19004_c1_g3_i1 | ---NA--- | 454 |  |  |
| TRINITY_DN19006_c0_g1_i2 | phosphate transporter PHO1 homolog 10 | 768 | 1.96E-65 | 82.1 |
| TRINITY_DN19006_c0_g1_i3 | phosphate transporter PHO1 homolog 10 | 812 | 3.34E-68 | 77.6 |
| TRINITY_DN19006_c0_g1_i4 | phosphate transporter PHO1 homolog 10 | 1053 | 6.38E-72 | 76.05 |
| TRINITY_DN19006_c0_g1_i5 | phosphate transporter PHO1 homolog 10 | 1009 | 3.88E-69 | 79.25 |
| TRINITY_DN19008_c0_g1_i1 | YTH domain-containing 1 isoform X2 | 706 | 4.19E-13 | 85.65 |
| TRINITY_DN19008_c0_g1_i2 | upstream activation factor subunit spp27-like | 885 | 5.01E-95 | 75.6 |
| TRINITY_DN19009_c0_g1_i1 | DNA-binding bromodomain-containing family | 2139 | 1.74E-112 | 61.7 |
| TRINITY_DN19009_c0_g1_i2 | DNA-binding bromodomain-containing family | 2225 | 1.35E-149 | 58.05 |
| TRINITY_DN19009_c0_g1_i3 | DNA-binding bromodomain-containing family | 2096 | 1.25E-112 | 61.7 |
| TRINITY_DN19009_c0_g1_i4 | DNA-binding bromodomain-containing family | 2206 | 1.11E-149 | 57.4 |
| TRINITY_DN19009_c0_g1_i5 | DNA-binding bromodomain-containing family | 2249 | 1.72E-149 | 57.4 |
| TRINITY_DN19009_c0_g1_i6 | DNA-binding bromodomain-containing family | 2115 | 1.40E-112 | 61.7 |
| TRINITY_DN19011_c0_g1_i1 | ---NA--- | 2058 |  |  |
| TRINITY_DN19015_c0_g1_i1 | short integuments mitochondrial | 1452 | 8.76E-146 | 83 |
| TRINITY_DN19015_c0_g1_i2 | short integuments mitochondrial | 1441 | 0 | 83.95 |
| TRINITY_DN19016_c0_g1_i1 | ---NA--- | 405 |  |  |
| TRINITY_DN19016_c0_g1_i2 | ---NA--- | 415 |  |  |
| TRINITY_DN19016_c0_g1_i3 | ---NA--- | 447 |  |  |
| TRINITY_DN19016_c0_g1_i8 | ---NA--- | 350 |  |  |
| TRINITY_DN19021_c0_g2_i2 | Hop-interacting THI113 | 1286 | 1.31E-76 | 76.4 |
| TRINITY_DN19021_c0_g2_i4 | Hop-interacting THI113 | 1300 | 1.52E-76 | 76.4 |
| TRINITY_DN19038_c0_g1_i1 | RNA polymerase II degradation factor 1 | 2584 | 4.26E-95 | 60.75 |
| TRINITY_DN19040_c0_g1_i1 | LEO1 homolog isoform X1 | 2497 | 0 | 79.95 |
| TRINITY_DN19040_c0_g1_i2 | LEO1 homolog isoform X1 | 2509 | 0 | 76.95 |
| TRINITY_DN19040_c0_g1_i3 | LEO1 homolog isoform X1 | 2497 | 0 | 78.3 |
| TRINITY_DN19040_c0_g1_i4 | LEO1 homolog isoform X1 | 2485 | 0 | 81.5 |
| TRINITY_DN19041_c0_g1_i1 | monofunctional riboflavin biosynthesis RIBA chloroplastic | 1760 | 0 | 88 |
| TRINITY_DN19041_c0_g1_i2 | monofunctional riboflavin biosynthesis RIBA chloroplastic | 1776 | 0 | 84.15 |
| TRINITY_DN19046_c0_g1_i1 | scarecrow 14 | 1733 | 0 | 72.15 |
| TRINITY_DN19057_c0_g1_i2 | alpha-glucosidase 2 | 3301 | 0 | 86.3 |
| TRINITY_DN19059_c0_g1_i1 | serine threonine- kinase 19 isoform X1 | 446 | 4.45E-64 | 87.55 |
| TRINITY_DN19059_c0_g1_i3 | serine threonine- kinase 19 isoform X1 | 1239 | 1.17E-124 | 82.3 |
| TRINITY_DN19060_c0_g1_i1 | hypothetical protein | 3143 | 3.34E-46 | 49.15 |
| TRINITY_DN19063_c0_g1_i1 | hypothetical protein SOVF_134120 | 1259 | 6.90E-58 | 83.45 |
| TRINITY_DN19064_c0_g1_i1 | pollen-specific leucine-rich repeat extensin 1 | 728 | 3.76E-28 | 84.55 |
| TRINITY_DN19064_c0_g1_i2 | pollen-specific leucine-rich repeat extensin 1 | 1165 | 1.14E-26 | 84.55 |
| TRINITY_DN19064_c0_g1_i3 | pollen-specific leucine-rich repeat extensin 1 | 1177 | 1.22E-26 | 84.55 |
| TRINITY_DN19064_c0_g1_i4 | pollen-specific leucine-rich repeat extensin 1 | 1093 | 7.60E-27 | 84.55 |
| TRINITY_DN19064_c0_g1_i5 | pollen-specific leucine-rich repeat extensin 1 | 1081 | 7.07E-27 | 84.55 |
| TRINITY_DN19075_c0_g1_i1 | auxin response factor 9 | 1334 | 6.94E-137 | 60.65 |
| TRINITY_DN19075_c0_g1_i2 | auxin response factor 9 | 2262 | 0 | 74.1 |
| TRINITY_DN19075_c0_g2_i1 | auxin response factor 9 | 804 | 9.00E-54 | 88.55 |
| TRINITY_DN19078_c0_g1_i1 | serine arginine-rich splicing factor SC35-like | 1284 | 1.66E-70 | 96.65 |
| TRINITY_DN19078_c0_g1_i2 | serine arginine-rich splicing factor SC35-like | 1287 | 2.59E-71 | 95.45 |
| TRINITY_DN19078_c0_g1_i3 | serine arginine-rich splicing factor SC35-like | 1255 | 2.24E-74 | 96.75 |
| TRINITY_DN19082_c0_g1_i1 | U-box domain-containing 4 | 2173 | 1.16E-180 | 76.55 |
| TRINITY_DN19084_c0_g1_i1 | oligopeptide transporter 3 | 2679 | 0 | 92.3 |
| TRINITY_DN1908_c0_g1_i1 | ---NA--- | 244 |  |  |
| TRINITY_DN19090_c0_g1_i1 | MLO 11 isoform X1 | 2193 | 0 | 86.25 |
| TRINITY_DN19090_c0_g1_i2 | MLO 11 isoform X1 | 2031 | 0 | 85.65 |
| TRINITY_DN19092_c0_g1_i1 | AE7-like 1 | 739 | 1.90E-83 | 91.6 |
| TRINITY_DN19095_c0_g2_i1 | Zinc RING-type | 1808 | 1.05E-51 | 43.85 |
| TRINITY_DN19097_c0_g1_i1 | maturase K (chloroplast) | 2369 | 0 | 94.8 |
| TRINITY_DN19100_c0_g1_i1 | thioredoxin reductase NTRC | 2059 | 0 | 94.05 |
| TRINITY_DN19100_c0_g1_i2 | thioredoxin reductase NTRC | 2036 | 0 | 92.65 |
| TRINITY_DN19102_c0_g1_i1 | S-acyltransferase 10-like | 868 | 1.07E-105 | 73.3 |
| TRINITY_DN19102_c0_g1_i2 | S-acyltransferase 10-like | 1509 | 3.58E-154 | 77.05 |
| TRINITY_DN19102_c0_g1_i3 | S-acyltransferase 10-like | 1489 | 1.31E-115 | 73.75 |
| TRINITY_DN19102_c0_g2_i1 | S-acyltransferase 10-like | 439 | 2.92E-33 | 65.85 |
| TRINITY_DN19103_c0_g1_i3 | tRNA (adenine(58)-N(1))-methyltransferase catalytic subunit TRMT61A | 1332 | 4.11E-178 | 85.75 |
| TRINITY_DN19103_c0_g1_i4 | tRNA (adenine(58)-N(1))-methyltransferase catalytic subunit TRMT61A | 1558 | 4.11E-177 | 85.75 |
| TRINITY_DN19103_c0_g1_i5 | tRNA (adenine(58)-N(1))-methyltransferase catalytic subunit TRMT61A | 1359 | 3.70E-178 | 85.75 |
| TRINITY_DN19107_c0_g1_i2 | TRIGALACTOSYLDIACYLGLYCEROL chloroplastic | 1892 | 0 | 74.65 |
| TRINITY_DN1910_c0_g2_i1 | ribosome biogenesis BMS1 homolog | 1727 | 0 | 78.95 |
| TRINITY_DN19114_c0_g1_i1 | probable purine permease 4 | 464 | 1.03E-40 | 79.35 |
| TRINITY_DN19114_c0_g2_i1 | probable purine permease 4 | 1279 | 4.32E-162 | 81.55 |
| TRINITY_DN19118_c0_g1_i1 | ---NA--- | 1208 |  |  |
| TRINITY_DN1911_c0_g1_i1 | cellulose synthase E6 | 388 | 5.93E-59 | 86.35 |
| TRINITY_DN19124_c0_g1_i1 | transcriptional corepressor LEUNIG-like isoform X2 | 2760 | 0 | 73.5 |
| TRINITY_DN19124_c1_g1_i1 | transcriptional corepressor LEUNIG | 437 | 4.51E-59 | 99.9 |
| TRINITY_DN19125_c0_g1_i1 | SPX domain-containing 1 | 1239 | 2.13E-142 | 78.95 |
| TRINITY_DN19125_c0_g2_i1 | SPX domain-containing 1 | 1132 | 8.46E-136 | 76.95 |
| TRINITY_DN19125_c2_g1_i1 | ---NA--- | 230 |  |  |
| TRINITY_DN19126_c0_g1_i1 | adenylyl-sulfate kinase chloroplastic isoform X7 | 1877 | 3.39E-128 | 86.95 |
| TRINITY_DN19126_c0_g2_i1 | ---NA--- | 583 |  |  |
| TRINITY_DN19126_c0_g3_i2 | adenylyl-sulfate kinase chloroplastic isoform X7 | 1048 | 7.27E-129 | 89.45 |
| TRINITY_DN19126_c0_g3_i3 | adenylyl-sulfate kinase 3 isoform X1 | 1065 | 5.86E-133 | 88.85 |
| TRINITY_DN19131_c0_g1_i1 | ATP-dependent zinc metalloprotease FTSH chloroplastic mitochondrial | 2904 | 0 | 90.35 |
| TRINITY_DN19135_c1_g2_i1 | ---NA--- | 1199 |  |  |
| TRINITY_DN19139_c0_g1_i1 | serine threonine- kinase HT1 | 1878 | 0 | 88.65 |
| TRINITY_DN19139_c0_g1_i3 | serine threonine- kinase HT1 | 1185 | 9.87E-89 | 88.7 |
| TRINITY_DN19139_c1_g1_i1 | serine threonine- kinase HT1-like | 2150 | 0 | 78.1 |
| TRINITY_DN1913_c0_g1_i2 | ---NA--- | 424 |  |  |
| TRINITY_DN19144_c1_g3_i1 | vacuolar-sorting receptor 6-like | 2372 | 0 | 85.3 |
| TRINITY_DN19145_c0_g1_i1 | fatty acyl- reductase 2 | 2181 | 0 | 78.25 |
| TRINITY_DN19145_c0_g1_i2 | fatty acyl- reductase 2 | 2154 | 0 | 77.85 |
| TRINITY_DN19146_c0_g1_i1 | 39S ribosomal mitochondrial | 1021 | 1.50E-80 | 85.65 |
| TRINITY_DN19149_c0_g1_i2 | apoptosis inhibitor 5 API5 | 1825 | 0 | 85.35 |
| TRINITY_DN19149_c0_g2_i1 | apoptosis inhibitor 5 API5 isoform X1 | 2240 | 0 | 85.8 |
| TRINITY_DN19150_c0_g1_i1 | cytochrome P450 family | 1640 | 3.64E-107 | 74.35 |
| TRINITY_DN19150_c1_g1_i1 | F-box At3g12350 | 2105 | 3.56E-108 | 72.9 |
| TRINITY_DN19150_c1_g1_i2 | F-box At3g12350 | 2203 | 2.57E-105 | 76.35 |
| TRINITY_DN19156_c0_g3_i3 | far upstream element-binding 2-like | 2958 | 3.63E-22 | 57.45 |
| TRINITY_DN19157_c0_g1_i1 | rhomboid 20 | 1328 | 2.60E-151 | 80.75 |
| TRINITY_DN19161_c0_g1_i1 | ---NA--- | 259 |  |  |
| TRINITY_DN19161_c0_g1_i2 | ---NA--- | 391 |  |  |
| TRINITY_DN19161_c0_g1_i3 | aquaporin NIP1-1-like | 1243 | 9.25E-165 | 86.6 |
| TRINITY_DN19162_c0_g1_i1 | Homeobox-leucine zipper HAT4 | 1429 | 9.17E-132 | 77.5 |
| TRINITY_DN19174_c0_g1_i1 | hypothetical protein SOVF_038670 isoform B | 3124 | 0 | 68.25 |
| TRINITY_DN19182_c0_g1_i1 | APO mitochondrial | 1829 | 6.95E-148 | 74.6 |
| TRINITY_DN19182_c0_g2_i1 | APO mitochondrial | 618 | 3.47E-60 | 74.95 |
| TRINITY_DN19186_c0_g1_i1 | LRR repeats and ubiquitin-like domain-containing At2g30105 | 1587 | 2.01E-136 | 72.75 |
| TRINITY_DN19186_c0_g1_i2 | LRR repeats and ubiquitin-like domain-containing At2g30105 | 1671 | 8.29E-138 | 73.05 |
| TRINITY_DN19186_c0_g1_i3 | LRR repeats and ubiquitin-like domain-containing At2g30105 | 1667 | 6.84E-104 | 68.5 |
| TRINITY_DN19186_c0_g1_i4 | LRR repeats and ubiquitin-like domain-containing At2g30105 | 1751 | 9.53E-106 | 68.3 |
| TRINITY_DN19188_c0_g1_i1 | Kinase superfamily with octicosapeptide Phox Bem1p isoform 1 | 4468 | 0 | 60.65 |
| TRINITY_DN19192_c0_g1_i3 | Alpha beta-Hydrolases superfamily isoform 1 | 1084 | 3.13E-69 | 59.5 |
| TRINITY_DN19196_c0_g1_i1 | translation initiation factor eIF-2B subunit gamma | 1537 | 0 | 86.85 |
| TRINITY_DN19196_c0_g1_i2 | translation initiation factor eIF-2B subunit gamma | 1642 | 0 | 81.1 |
| TRINITY_DN19196_c0_g1_i3 | translation initiation factor eIF-2B subunit gamma | 1524 | 0 | 87.3 |
| TRINITY_DN19196_c1_g1_i1 | ---NA--- | 243 |  |  |
| TRINITY_DN19215_c0_g1_i1 | expansin-A13 | 1003 | 2.45E-123 | 80.45 |
| TRINITY_DN19215_c0_g1_i2 | expansin-A13-like | 1015 | 4.83E-127 | 82.1 |
| TRINITY_DN1921_c0_g1_i1 | type 1 phosphatases regulator ypi1 | 717 | 1.47E-36 | 72.45 |
| TRINITY_DN19234_c0_g1_i1 | ---NA--- | 872 |  |  |
| TRINITY_DN19235_c0_g1_i1 | BAR domain-containing | 872 | 6.53E-95 | 82.15 |
| TRINITY_DN19237_c0_g1_i1 | E3 ubiquitin- ligase RNF8-A-like isoform X2 | 963 | 4.62E-160 | 84.55 |
| TRINITY_DN19237_c1_g2_i1 | receptor homology transmembrane domain- and RING domain-containing 2-like | 1033 | 2.64E-71 | 59.55 |
| TRINITY_DN19238_c1_g1_i1 | ---NA--- | 1282 |  |  |
| TRINITY_DN19238_c1_g1_i2 | ---NA--- | 1249 |  |  |
| TRINITY_DN19238_c1_g1_i3 | ---NA--- | 1064 |  |  |
| TRINITY_DN19238_c1_g1_i4 | ---NA--- | 1118 |  |  |
| TRINITY_DN19238_c1_g1_i5 | ---NA--- | 1467 |  |  |
| TRINITY_DN19238_c1_g1_i6 | ---NA--- | 1336 |  |  |
| TRINITY_DN19247_c0_g1_i1 | PREDICTED: uncharacterized protein LOC104908518 | 703 | 1.68E-67 | 78.15 |
| TRINITY_DN1924_c0_g1_i1 | GPR107-like | 1920 | 0 | 90.95 |
| TRINITY_DN19264_c0_g1_i1 | Mpv17 isoform X2 | 1688 | 1.24E-124 | 87.7 |
| TRINITY_DN19264_c0_g1_i2 | Mpv17 isoform X2 | 1566 | 4.56E-101 | 87.65 |
| TRINITY_DN19264_c0_g1_i3 | Mpv17 isoform X2 | 1998 | 1.06E-123 | 85.7 |
| TRINITY_DN19269_c0_g1_i1 | RRC1 isoform X1 | 3221 | 0 | 84.2 |
| TRINITY_DN19273_c1_g1_i1 | elongation factor G- chloroplastic | 2703 | 0 | 94.7 |
| TRINITY_DN19279_c0_g1_i1 | F-box CPR30 | 497 | 6.99E-17 | 48.5 |
| TRINITY_DN19279_c0_g1_i2 | F-box CPR30 | 620 | 7.63E-12 | 48.5 |
| TRINITY_DN19284_c0_g2_i1 | 4-alpha- chloroplastic amyloplastic | 1427 | 0 | 83 |
| TRINITY_DN19284_c0_g2_i2 | 4-alpha- chloroplastic amyloplastic | 1408 | 9.67E-170 | 81.5 |
| TRINITY_DN19284_c0_g2_i3 | 4-alpha- chloroplastic amyloplastic | 1318 | 8.20E-174 | 90.1 |
| TRINITY_DN19284_c0_g2_i4 | 4-alpha- chloroplastic amyloplastic | 1337 | 0 | 89.75 |
| TRINITY_DN19285_c0_g1_i1 | ---NA--- | 392 |  |  |
| TRINITY_DN19285_c0_g1_i2 | ---NA--- | 698 |  |  |
| TRINITY_DN19285_c0_g1_i3 | ---NA--- | 740 |  |  |
| TRINITY_DN19294_c0_g1_i1 | heat shock 70 kDa 15-like | 3095 | 0 | 84.4 |
| TRINITY_DN19294_c0_g1_i2 | heat shock 70 kDa 15-like | 3098 | 0 | 84.8 |
| TRINITY_DN19298_c0_g1_i1 | ---NA--- | 253 |  |  |
| TRINITY_DN19298_c0_g2_i1 | diacylglycerol O-acyltransferase 1 | 1757 | 0 | 89.35 |
| TRINITY_DN1929_c0_g1_i1 | trihelix transcription factor GT-1 isoform X2 | 516 | 1.37E-40 | 95 |
| TRINITY_DN19302_c0_g1_i1 | syntaxin-132-like | 1373 | 9.14E-171 | 89.5 |
| TRINITY_DN19304_c0_g1_i1 | Mog1 alpha beta alpha sandwich | 1246 | 1.78E-105 | 80.2 |
| TRINITY_DN19305_c0_g1_i1 | UPSTREAM OF FLC | 1627 | 7.36E-109 | 60.55 |
| TRINITY_DN19305_c0_g1_i2 | UPSTREAM OF FLC | 1642 | 4.18E-111 | 60.8 |
| TRINITY_DN19309_c0_g1_i1 | ---NA--- | 592 |  |  |
| TRINITY_DN19309_c0_g1_i2 | ---NA--- | 400 |  |  |
| TRINITY_DN19309_c0_g1_i3 | ---NA--- | 461 |  |  |
| TRINITY_DN19309_c0_g1_i4 | ---NA--- | 538 |  |  |
| TRINITY_DN19317_c0_g1_i1 | ABHD11 isoform X1 | 1638 | 0 | 85.95 |
| TRINITY_DN19318_c0_g1_i1 | acetate butyrate-- ligase peroxisomal | 2014 | 0 | 89.05 |
| TRINITY_DN19318_c0_g1_i2 | ---NA--- | 298 |  |  |
| TRINITY_DN19318_c0_g1_i3 | acetate butyrate-- ligase peroxisomal | 2197 | 0 | 89.05 |
| TRINITY_DN19319_c0_g1_i1 | Translation initiation factor 3 family isoform 1 | 1636 | 1.14E-78 | 59 |
| TRINITY_DN19319_c0_g1_i2 | Translation initiation factor 3 family isoform 1 | 1558 | 5.10E-80 | 64.1 |
| TRINITY_DN19327_c0_g1_i1 | programmed cell death 2 | 1611 | 0 | 76.6 |
| TRINITY_DN19328_c0_g1_i1 | meiotic recombination DMC1 homolog | 1443 | 0 | 96.1 |
| TRINITY_DN19328_c0_g1_i2 | meiotic recombination DMC1 homolog | 1465 | 0 | 95.5 |
| TRINITY_DN19328_c0_g1_i3 | meiotic recombination DMC1 homolog | 1553 | 0 | 95.15 |
| TRINITY_DN19333_c0_g1_i3 | succinate-- ligase [ADP-forming] subunit alpha- mitochondrial | 1433 | 0 | 91.2 |
| TRINITY_DN19342_c0_g1_i2 | ---NA--- | 276 |  |  |
| TRINITY_DN19350_c0_g1_i1 | nuclear poly(A) polymerase 1 | 2678 | 0 | 76.3 |
| TRINITY_DN1935_c0_g1_i1 | ---NA--- | 220 |  |  |
| TRINITY_DN19363_c0_g1_i1 | cation H(+) antiporter 20 | 1411 | 0 | 75.25 |
| TRINITY_DN19364_c0_g1_i1 | transcription factor bHLH123-like isoform X2 | 550 | 2.73E-71 | 78.9 |
| TRINITY_DN19364_c0_g2_i1 | transcription factor bHLH123-like isoform X2 | 262 | 2.45E-09 | 63.41 |
| TRINITY_DN19369_c0_g1_i1 | elicitor-responsive 1 | 712 | 1.38E-66 | 73.9 |
| TRINITY_DN19369_c0_g1_i2 | elicitor-responsive 1-like | 576 | 8.23E-44 | 74.45 |
| TRINITY_DN19372_c0_g1_i3 | U-box domain-containing 8 | 1025 | 3.82E-61 | 58.05 |
| TRINITY_DN19376_c0_g1_i1 | GPR107 | 1813 | 0 | 88.45 |
| TRINITY_DN19377_c0_g1_i1 | uncharacterized aarF domain-containing kinase chloroplastic isoform X1 | 2902 | 0 | 90.3 |
| TRINITY_DN19385_c0_g1_i1 | katanin p80 WD40 repeat-containing subunit B1 homolog isoform X1 | 3121 | 0 | 79.8 |
| TRINITY_DN19389_c0_g1_i1 | molybdate-anion transporter | 3329 | 0 | 75.15 |
| TRINITY_DN19389_c0_g1_i2 | molybdate-anion transporter | 3402 | 0 | 75.15 |
| TRINITY_DN19390_c0_g1_i1 | pentatricopeptide repeat-containing mitochondrial | 573 | 3.71E-29 | 48.1 |
| TRINITY_DN19390_c0_g1_i2 | pentatricopeptide repeat-containing mitochondrial | 552 | 9.36E-27 | 48.25 |
| TRINITY_DN19391_c0_g1_i1 | ---NA--- | 648 |  |  |
| TRINITY_DN19407_c0_g1_i1 | dehydrodolichyl diphosphate synthase 2 | 1223 | 1.61E-151 | 83.05 |
| TRINITY_DN19408_c2_g2_i1 | SKP1 1B | 712 | 1.59E-47 | 76.65 |
| TRINITY_DN19410_c0_g1_i3 | ---NA--- | 655 |  |  |
| TRINITY_DN19410_c0_g1_i5 | ---NA--- | 551 |  |  |
| TRINITY_DN19415_c0_g1_i1 | trichome berefringence-like 7 | 1728 | 0 | 78.05 |
| TRINITY_DN1941_c0_g1_i1 | GPI-anchored LORELEI | 656 | 6.73E-44 | 76.1 |
| TRINITY_DN19428_c0_g2_i1 | TBC1 domain family member 13 | 1748 | 0 | 89.75 |
| TRINITY_DN19436_c0_g1_i1 | Retrotransposable element Tf2 | 434 | 4.28E-19 | 65.5 |
| TRINITY_DN1943_c0_g1_i1 | biotin carboxylase chloroplastic | 2177 | 0 | 92.95 |
| TRINITY_DN1943_c0_g1_i2 | biotin carboxylase chloroplastic | 2189 | 0 | 92.95 |
| TRINITY_DN19449_c0_g1_i1 | methylcrotonoyl- carboxylase subunit mitochondrial isoform X2 | 2946 | 0 | 83.8 |
| TRINITY_DN19459_c0_g1_i2 | ---NA--- | 1122 |  |  |
| TRINITY_DN19459_c0_g1_i3 | ---NA--- | 1142 |  |  |
| TRINITY_DN19459_c0_g1_i4 | ---NA--- | 1033 |  |  |
| TRINITY_DN19459_c0_g1_i6 | flavin-containing monooxygenase FMO GS-OX-like 9 | 977 | 3.21E-08 | 91.77 |
| TRINITY_DN19468_c0_g1_i2 | 60S ribosomal L7-1 | 963 | 3.97E-134 | 76.9 |
| TRINITY_DN19470_c0_g2_i1 | ---NA--- | 759 |  |  |
| TRINITY_DN19470_c0_g2_i2 | ---NA--- | 717 |  |  |
| TRINITY_DN19474_c0_g4_i1 | ---NA--- | 474 |  |  |
| TRINITY_DN19476_c0_g1_i1 | proline-rich receptor kinase PERK8 | 2383 | 0 | 83.1 |
| TRINITY_DN19476_c0_g1_i2 | proline-rich receptor kinase PERK8 | 2221 | 0 | 71.45 |
| TRINITY_DN19483_c0_g1_i1 | LOV domain-containing | 1471 | 1.05E-150 | 66.65 |
| TRINITY_DN19483_c0_g1_i2 | LOV domain-containing | 1588 | 0 | 77.55 |
| TRINITY_DN19483_c0_g1_i3 | LOV domain-containing | 1563 | 6.02E-147 | 76.25 |
| TRINITY_DN19483_c0_g1_i4 | LOV domain-containing | 1496 | 7.74E-140 | 78.1 |
| TRINITY_DN19486_c1_g1_i1 | ---NA--- | 357 |  |  |
| TRINITY_DN1949_c0_g1_i1 | myb family transcription factor EFM-like | 1361 | 4.49E-49 | 55.65 |
| TRINITY_DN1949_c0_g2_i1 | myb family transcription factor EFM-like | 1493 | 9.70E-87 | 54.4 |
| TRINITY_DN19501_c0_g1_i1 | inner centromere isoform X3 | 1520 | 4.19E-120 | 63 |
| TRINITY_DN19502_c0_g1_i1 | NKAP [Tarenaya hassleriana] | 788 | 6.06E-87 | 92.75 |
| TRINITY_DN19509_c0_g1_i1 | indeterminate-domain chloroplastic | 746 | 1.50E-15 | 52.6 |
| TRINITY_DN19509_c0_g1_i2 | indeterminate-domain chloroplastic | 747 | 2.68E-15 | 52.9 |
| TRINITY_DN19509_c0_g2_i1 | indeterminate-domain chloroplastic-like | 1044 | 6.01E-24 | 53.75 |
| TRINITY_DN19509_c0_g4_i1 | F-box SKIP14 | 1992 | 5.28E-95 | 70.05 |
| TRINITY_DN19515_c0_g1_i1 | serine threonine- phosphatase BSL1 | 3210 | 0 | 90.9 |
| TRINITY_DN19524_c0_g1_i1 | 2-C-methyl-D-erythritol 4-phosphate chloroplastic | 1480 | 1.45E-155 | 80.85 |
| TRINITY_DN19524_c0_g1_i3 | 2-C-methyl-D-erythritol 4-phosphate chloroplastic | 1472 | 1.36E-155 | 80.85 |
| TRINITY_DN19524_c0_g1_i4 | 2-C-methyl-D-erythritol 4-phosphate chloroplastic | 1577 | 4.61E-155 | 80.85 |
| TRINITY_DN19524_c0_g1_i5 | 2-C-methyl-D-erythritol 4-phosphate chloroplastic | 1569 | 4.16E-155 | 80.85 |
| TRINITY_DN19525_c1_g1_i2 | Leucine-rich cysteine-containing subtype | 2050 | 4.71E-168 | 58.4 |
| TRINITY_DN19525_c1_g2_i1 | Leucine-rich cysteine-containing subtype | 1561 | 1.45E-122 | 56.1 |
| TRINITY_DN19528_c0_g1_i1 | homeobox cut | 1149 | 2.53E-44 | 68.65 |
| TRINITY_DN19537_c0_g2_i1 | PREDICTED: uncharacterized protein LOC104896483 | 637 | 9.62E-37 | 74.85 |
| TRINITY_DN19537_c0_g2_i2 | PREDICTED: uncharacterized protein LOC101264582 isoform X2 | 685 | 9.88E-22 | 71.15 |
| TRINITY_DN19538_c0_g1_i1 | J domain-containing required for chloroplast accumulation response 1 isoform X2 | 2418 | 7.91E-123 | 48.25 |
| TRINITY_DN19538_c0_g1_i2 | J domain-containing required for chloroplast accumulation response 1 | 2133 | 9.07E-74 | 52.75 |
| TRINITY_DN19544_c0_g1_i1 | probable methyltransferase PMT23 | 1824 | 0 | 82.7 |
| TRINITY_DN19544_c0_g1_i2 | probable methyltransferase PMT23 | 1853 | 0 | 86.4 |
| TRINITY_DN19544_c0_g1_i3 | probable methyltransferase PMT23 | 1836 | 0 | 82.7 |
| TRINITY_DN19544_c0_g1_i4 | probable methyltransferase PMT23 | 1865 | 0 | 86.05 |
| TRINITY_DN19564_c0_g1_i1 | 7-hydroxymethyl chlorophyll a chloroplastic | 1924 | 0 | 93.05 |
| TRINITY_DN19564_c0_g1_i2 | 7-hydroxymethyl chlorophyll a chloroplastic | 1940 | 0 | 93.85 |
| TRINITY_DN19564_c0_g1_i3 | 7-hydroxymethyl chlorophyll a chloroplastic | 1055 | 3.25E-113 | 89.45 |
| TRINITY_DN19567_c0_g2_i1 | RST1 isoform X3 | 3319 | 0 | 67.35 |
| TRINITY_DN19570_c0_g1_i1 | ---NA--- | 588 |  |  |
| TRINITY_DN19570_c0_g2_i1 | probable transcription factor At4g00390 | 894 | 5.07E-07 | 51.83 |
| TRINITY_DN19570_c0_g2_i2 | probable transcription factor At1g61730 | 882 | 1.20E-12 | 53.23 |
| TRINITY_DN19579_c0_g1_i1 | ---NA--- | 425 |  |  |
| TRINITY_DN1957_c0_g1_i1 | hypothetical protein SOVF_074290 | 953 | 6.61E-45 | 68.1 |
| TRINITY_DN1957_c0_g2_i1 | hypothetical protein SOVF_074290 | 954 | 6.56E-40 | 68.1 |
| TRINITY_DN19581_c0_g1_i1 | ADP,ATP carrier mitochondrial | 1558 | 0 | 88.85 |
| TRINITY_DN19581_c0_g1_i2 | ADP,ATP carrier mitochondrial | 1663 | 0 | 88.85 |
| TRINITY_DN19581_c1_g2_i1 | ---NA--- | 368 |  |  |
| TRINITY_DN19581_c1_g3_i1 | ---NA--- | 270 |  |  |
| TRINITY_DN19582_c0_g1_i1 | serine threonine- kinase EDR1-like | 970 | 7.46E-85 | 90.65 |
| TRINITY_DN19582_c0_g1_i2 | serine threonine- kinase EDR1 | 3427 | 0 | 74.2 |
| TRINITY_DN19583_c0_g1_i1 | ninja-family AFP3-like | 2549 | 1.43E-123 | 67 |
| TRINITY_DN19583_c0_g1_i2 | ninja-family AFP3-like | 2266 | 1.74E-128 | 67 |
| TRINITY_DN19585_c0_g3_i1 | chromatin modification-related MEAF6 | 1264 | 8.87E-10 | 71.71 |
| TRINITY_DN19586_c0_g1_i1 | ---NA--- | 443 |  |  |
| TRINITY_DN19586_c0_g1_i2 | PREDICTED: uncharacterized protein LOC100241465 | 1776 | 2.30E-55 | 49.55 |
| TRINITY_DN19586_c0_g1_i3 | pore-forming toxin Hfr-2 | 1073 | 1.21E-27 | 53.4 |
| TRINITY_DN19589_c0_g1_i1 | BTB POZ domain-containing At1g21780 | 1649 | 4.50E-164 | 69.95 |
| TRINITY_DN19589_c0_g1_i2 | BTB POZ domain-containing At1g21780 | 1556 | 8.45E-168 | 74.9 |
| TRINITY_DN19589_c0_g1_i3 | BTB POZ domain-containing At1g21780 | 1454 | 8.43E-176 | 82.65 |
| TRINITY_DN19591_c1_g1_i1 | ras-related RABD2a | 1078 | 6.44E-145 | 96.15 |
| TRINITY_DN19591_c1_g1_i2 | ras-related RABD2a | 1078 | 1.87E-145 | 96.2 |
| TRINITY_DN19592_c1_g1_i1 | pyridoxal 5 -phosphate synthase subunit | 424 | 2.33E-77 | 88.25 |
| TRINITY_DN19592_c1_g1_i2 | probable pyridoxal 5 -phosphate synthase subunit PDX1 | 486 | 3.31E-66 | 88.35 |
| TRINITY_DN19593_c0_g1_i1 | mRNA-decapping enzyme | 1700 | 6.72E-116 | 70.4 |
| TRINITY_DN19593_c1_g1_i1 | ---NA--- | 298 |  |  |
| TRINITY_DN19597_c0_g1_i1 | microtubule-associated TORTIFOLIA1 | 2402 | 0 | 74.2 |
| TRINITY_DN19604_c0_g1_i1 | STRUBBELIG-RECEPTOR FAMILY 8 | 2434 | 0 | 84.55 |
| TRINITY_DN19609_c0_g1_i1 | auxilin-related 2-like | 3362 | 1.79E-109 | 86.85 |
| TRINITY_DN19609_c0_g1_i2 | auxilin-related 2-like | 3359 | 1.52E-90 | 85.5 |
| TRINITY_DN19612_c0_g1_i1 | transmembrane DDB_G0273707 DDB_G0273361 | 1172 | 4.84E-93 | 88.25 |
| TRINITY_DN19616_c0_g1_i1 | Chaperone -domain superfamily | 1339 | 1.15E-73 | 49.7 |
| TRINITY_DN19621_c0_g1_i1 | ---NA--- | 256 |  |  |
| TRINITY_DN19621_c1_g1_i1 | ---NA--- | 209 |  |  |
| TRINITY_DN19621_c1_g2_i1 | auxilin 1 | 3298 | 3.76E-76 | 54.05 |
| TRINITY_DN19623_c1_g1_i1 | ---NA--- | 827 |  |  |
| TRINITY_DN19623_c1_g2_i1 | RNA-binding (RRM RBD RNP motifs) family isoform 1 | 981 | 5.20E-08 | 52.83 |
| TRINITY_DN19630_c0_g1_i1 | glyceraldehyde-3-phosphate dehydrogenase | 1689 | 0 | 96.1 |
| TRINITY_DN19630_c0_g1_i2 | glyceraldehyde-3-phosphate cytosolic-like | 1689 | 0 | 96.35 |
| TRINITY_DN19630_c0_g1_i3 | glyceraldehyde-3-phosphate dehydrogenase | 1689 | 0 | 96.15 |
| TRINITY_DN19630_c0_g1_i4 | glyceraldehyde-3-phosphate cytosolic-like | 1689 | 0 | 96.3 |
| TRINITY_DN19630_c0_g1_i5 | glyceraldehyde-3-phosphate cytosolic-like | 1689 | 0 | 96.35 |
| TRINITY_DN19630_c0_g1_i6 | glyceraldehyde-3-phosphate cytosolic-like | 1689 | 0 | 96.3 |
| TRINITY_DN19631_c0_g1_i1 | eukaryotic initiation factor 4A-3 | 806 | 2.02E-152 | 92.95 |
| TRINITY_DN19631_c0_g2_i1 | eukaryotic initiation factor 4A-3 | 1859 | 0 | 94.8 |
| TRINITY_DN19631_c0_g2_i2 | eukaryotic initiation factor 4A-3 | 1861 | 0 | 95.3 |
| TRINITY_DN19631_c0_g2_i3 | eukaryotic initiation factor 4A-3 | 1861 | 0 | 95.25 |
| TRINITY_DN19642_c0_g1_i1 | DUF21 domain-containing At2g14520-like | 1846 | 0 | 86.35 |
| TRINITY_DN19644_c0_g3_i1 | ---NA--- | 661 |  |  |
| TRINITY_DN19645_c1_g1_i1 | probable 1-deoxy-D-xylulose-5-phosphate chloroplastic isoform X2 | 1168 | 1.43E-19 | 92.25 |
| TRINITY_DN19646_c0_g1_i1 | SAND family | 1779 | 0 | 90.6 |
| TRINITY_DN19646_c0_g1_i2 | SAND family | 1549 | 0 | 90.6 |
| TRINITY_DN19646_c1_g1_i1 | ---NA--- | 515 |  |  |
| TRINITY_DN19647_c0_g1_i1 | F-box LRR-repeat At5g38386 | 2055 | 1.62E-49 | 48.45 |
| TRINITY_DN19647_c0_g1_i2 | F-box LRR-repeat At5g38386 | 2343 | 4.22E-49 | 48.2 |
| TRINITY_DN19647_c0_g3_i1 | ribosomal L20 (chloroplast) | 1867 | 5.74E-42 | 91.65 |
| TRINITY_DN19649_c0_g1_i1 | probable E3 ubiquitin ligase SUD1 | 1790 | 0 | 87.5 |
| TRINITY_DN19649_c0_g2_i1 | probable E3 ubiquitin ligase SUD1 | 1784 | 0 | 87.5 |
| TRINITY_DN19649_c0_g3_i1 | probable E3 ubiquitin ligase SUD1 | 1786 | 0 | 87.5 |
| TRINITY_DN19649_c0_g4_i1 | probable E3 ubiquitin ligase SUD1 | 1788 | 0 | 87.5 |
| TRINITY_DN19649_c0_g5_i1 | probable E3 ubiquitin ligase SUD1 | 1785 | 0 | 87.5 |
| TRINITY_DN19649_c0_g6_i1 | probable E3 ubiquitin ligase SUD1 | 1788 | 0 | 87.5 |
| TRINITY_DN19649_c0_g7_i1 | probable E3 ubiquitin ligase SUD1 | 1781 | 0 | 87.5 |
| TRINITY_DN19649_c0_g8_i1 | probable E3 ubiquitin ligase SUD1 | 1786 | 0 | 87.5 |
| TRINITY_DN19650_c0_g1_i1 | RNA polymerase II-associated 3 isoform X1 | 1998 | 1.19E-166 | 66.55 |
| TRINITY_DN19652_c0_g1_i1 | transportin-1 isoform X1 | 3401 | 0 | 88.7 |
| TRINITY_DN19652_c0_g1_i2 | transportin-1 isoform X1 | 3144 | 0 | 88.7 |
| TRINITY_DN19652_c0_g2_i1 | transportin-1 | 1726 | 0 | 88.2 |
| TRINITY_DN19652_c0_g3_i1 | transportin-1 | 1720 | 0 | 88.2 |
| TRINITY_DN19655_c0_g1_i1 | DEAD-box ATP-dependent RNA helicase 50 | 2123 | 0 | 74.4 |
| TRINITY_DN19658_c0_g1_i1 | probable histone H2A variant 3 | 696 | 1.19E-84 | 93.9 |
| TRINITY_DN19658_c0_g2_i1 | probable histone H2A variant 3 | 692 | 1.10E-84 | 93.9 |
| TRINITY_DN19658_c1_g1_i1 | ---NA--- | 212 |  |  |
| TRINITY_DN19659_c0_g2_i1 | ---NA--- | 396 |  |  |
| TRINITY_DN19659_c0_g3_i2 | jasmonic acid-amido synthetase JAR1 | 2340 | 0 | 85 |
| TRINITY_DN19659_c0_g3_i3 | jasmonic acid-amido synthetase JAR1 | 2362 | 0 | 78.05 |
| TRINITY_DN19659_c0_g3_i4 | jasmonic acid-amido synthetase JAR1 | 2509 | 0 | 82.7 |
| TRINITY_DN19666_c0_g1_i1 | VACUOLELESS1 | 2926 | 0 | 87.5 |
| TRINITY_DN19671_c0_g2_i1 | ubinuclein-1-like isoform X3 | 2243 | 0 | 62.65 |
| TRINITY_DN19671_c0_g4_i1 | ubinuclein-1-like isoform X1 | 278 | 2.41E-22 | 85.75 |
| TRINITY_DN19675_c0_g2_i1 | ATP synthase mitochondrial F1 complex assembly factor 2 | 1357 | 3.90E-133 | 87.95 |
| TRINITY_DN19679_c0_g1_i1 | leucine-rich repeat receptor kinase TDR | 1070 | 0 | 92 |
| TRINITY_DN19685_c0_g1_i1 | CD2 antigen cytoplasmic tail-binding 2 | 1537 | 1.34E-144 | 68.7 |
| TRINITY_DN19685_c0_g1_i2 | CD2 antigen cytoplasmic tail-binding 2 | 1686 | 1.82E-139 | 69.25 |
| TRINITY_DN19689_c0_g2_i1 | serine-threonine plant- | 1757 | 1.48E-178 | 79.3 |
| TRINITY_DN19702_c0_g1_i1 | ARM repeat superfamily isoform 1 | 674 | 1.81E-32 | 60.3 |
| TRINITY_DN19702_c0_g2_i1 | SIEL isoform X1 | 2692 | 0 | 59.65 |
| TRINITY_DN19702_c0_g2_i4 | SIEL isoform X1 | 2538 | 0 | 60.35 |
| TRINITY_DN19702_c0_g2_i5 | SIEL isoform X1 | 2576 | 0 | 56.85 |
| TRINITY_DN19702_c0_g2_i6 | SIEL isoform X1 | 2422 | 1.25E-156 | 58.8 |
| TRINITY_DN19704_c0_g1_i1 | ---NA--- | 309 |  |  |
| TRINITY_DN19704_c0_g2_i1 | ---NA--- | 255 |  |  |
| TRINITY_DN19704_c0_g2_i2 | ---NA--- | 250 |  |  |
| TRINITY_DN19704_c0_g3_i1 | ---NA--- | 258 |  |  |
| TRINITY_DN19709_c0_g1_i1 | ---NA--- | 283 |  |  |
| TRINITY_DN19710_c0_g1_i1 | tetraspanin-2-like | 972 | 2.06E-145 | 84.75 |
| TRINITY_DN19710_c0_g1_i2 | tetraspanin-2-like | 1128 | 1.46E-144 | 84.75 |
| TRINITY_DN19710_c1_g1_i1 | IQ-DOMAIN 32 isoform X2 | 2799 | 7.09E-167 | 51.2 |
| TRINITY_DN19712_c0_g3_i1 | phototropin-2-like isoform X1 | 604 | 4.46E-13 | 96.6 |
| TRINITY_DN19712_c0_g4_i1 | LOV domain-containing | 645 | 7.78E-13 | 94.45 |
| TRINITY_DN19713_c0_g1_i1 | probable very-long-chain enoyl- reductase art-1 | 769 | 9.97E-07 | 78 |
| TRINITY_DN19713_c0_g1_i3 | probable very-long-chain enoyl- reductase art-1 | 783 | 1.04E-06 | 78 |
| TRINITY_DN19713_c0_g1_i5 | probable very-long-chain enoyl- reductase art-1 | 864 | 1.36E-06 | 78 |
| TRINITY_DN19715_c0_g1_i1 | ribosomal L1 domain-containing 1-like | 1401 | 2.69E-101 | 72 |
| TRINITY_DN19715_c0_g2_i1 | serine arginine-rich splicing factor RS2Z33-like isoform X1 | 1489 | 1.83E-90 | 90.4 |
| TRINITY_DN19715_c0_g2_i3 | serine arginine-rich splicing factor RS2Z33-like isoform X1 | 1726 | 1.41E-56 | 84.45 |
| TRINITY_DN19716_c0_g1_i1 | Retrovirus-related Pol poly from transposon TNT 1-94 | 1080 | 2.86E-58 | 59.25 |
| TRINITY_DN19720_c0_g1_i1 | homeobox-leucine zipper HOX11 | 722 | 1.51E-18 | 51.14 |
| TRINITY_DN19720_c0_g2_i1 | homeobox-leucine zipper HOX11 | 248 | 1.43E-14 | 74 |
| TRINITY_DN19724_c1_g1_i1 | E3 ubiquitin- ligase SINAT5-like | 1427 | 0 | 87.05 |
| TRINITY_DN19725_c0_g1_i1 | DNA topoisomerase 2-binding 1 | 3358 | 0 | 69.1 |
| TRINITY_DN1972_c0_g1_i1 | PXR1-like isoform X2 | 1099 | 6.67E-51 | 64.15 |
| TRINITY_DN19736_c0_g1_i2 | probable LRR receptor-like serine threonine- kinase At4g37250 | 2589 | 0 | 68.7 |
| TRINITY_DN19736_c0_g2_i1 | probable LRR receptor-like serine threonine- kinase At4g37250 | 536 | 3.40E-36 | 77.6 |
| TRINITY_DN19743_c0_g1_i1 | glucose-6-phosphate 1- chloroplastic | 1942 | 0 | 96.1 |
| TRINITY_DN19743_c0_g1_i2 | glucose-6-phosphate 1- chloroplastic | 2635 | 0 | 92.05 |
| TRINITY_DN19743_c0_g1_i3 | glucose-6-phosphate 1- chloroplastic | 2037 | 0 | 96.15 |
| TRINITY_DN19744_c0_g1_i1 | RRP6-like 2 | 3098 | 0 | 68 |
| TRINITY_DN19744_c0_g1_i2 | RRP6-like 2 | 3376 | 0 | 81.75 |
| TRINITY_DN19744_c0_g1_i3 | RRP6-like 2 | 3185 | 0 | 65.35 |
| TRINITY_DN19744_c0_g1_i4 | RRP6-like 2 | 3037 | 0 | 68 |
| TRINITY_DN19745_c0_g1_i1 | PREDICTED: uncharacterized protein LOC109177684 | 243 | 1.13E-18 | 83.3 |
| TRINITY_DN19753_c0_g2_i1 | ---NA--- | 276 |  |  |
| TRINITY_DN19761_c0_g2_i1 | ---NA--- | 286 |  |  |
| TRINITY_DN19762_c0_g1_i4 | tubulin alpha-2 chain | 1485 | 0 | 98.2 |
| TRINITY_DN19762_c0_g1_i5 | tubulin alpha-1 chain | 1786 | 0 | 98.55 |
| TRINITY_DN19764_c0_g1_i1 | RING-box 1a | 760 | 5.12E-71 | 87.2 |
| TRINITY_DN19764_c0_g2_i2 | RING-box 1a | 795 | 2.25E-64 | 87.2 |
| TRINITY_DN19779_c0_g1_i1 | long-chain-alcohol oxidase FAO2 | 2769 | 0 | 78.9 |
| TRINITY_DN19779_c0_g2_i1 | long-chain-alcohol oxidase FAO2-like | 775 | 5.63E-15 | 58.5 |
| TRINITY_DN19779_c0_g3_i1 | long-chain-alcohol oxidase FAO2-like | 629 | 1.54E-11 | 60.6 |
| TRINITY_DN19780_c0_g2_i1 | hypothetical protein BVRB_8g182580 | 1673 | 2.71E-10 | 61 |
| TRINITY_DN19780_c0_g3_i1 | E6-like isoform X2 | 659 | 8.93E-16 | 55.5 |
| TRINITY_DN19786_c0_g1_i1 | NADH dehydrogenase [ubiquinone] 1 beta subcomplex subunit 3-A | 243 | 1.38E-25 | 91.5 |
| TRINITY_DN19786_c0_g2_i1 | NADH dehydrogenase [ubiquinone] 1 beta subcomplex subunit 3-A | 305 | 1.94E-26 | 86.25 |
| TRINITY_DN19786_c2_g1_i1 | glycine-rich cell wall structural -like | 1160 | 8.52E-13 | 88.35 |
| TRINITY_DN19786_c3_g1_i1 | ---NA--- | 444 |  |  |
| TRINITY_DN19786_c4_g1_i1 | ---NA--- | 302 |  |  |
| TRINITY_DN19789_c0_g1_i1 | RING-H2 finger ATL5-like | 996 | 7.34E-64 | 76.1 |
| TRINITY_DN19789_c0_g1_i2 | RING-H2 finger ATL5-like | 904 | 1.66E-32 | 76.2 |
| TRINITY_DN19814_c0_g1_i1 | PREDICTED: uncharacterized protein LOC104893517 | 1120 | 1.24E-47 | 56.35 |
| TRINITY_DN19814_c0_g1_i2 | PREDICTED: uncharacterized protein LOC104893517 | 1165 | 6.34E-17 | 67 |
| TRINITY_DN19814_c0_g1_i3 | PREDICTED: uncharacterized protein LOC104893517 | 1319 | 3.23E-47 | 56.9 |
| TRINITY_DN19814_c0_g1_i4 | PREDICTED: uncharacterized protein LOC104893517 | 1890 | 1.21E-81 | 57.65 |
| TRINITY_DN19814_c0_g2_i1 | ---NA--- | 265 |  |  |
| TRINITY_DN19816_c0_g1_i1 | DNA-directed RNA polymerase IV subunit 1 isoform X1 | 4815 | 0 | 71.8 |
| TRINITY_DN19817_c0_g1_i1 | E3 ubiquitin- ligase ORTHRUS 2-like | 2744 | 0 | 72.85 |
| TRINITY_DN19834_c0_g1_i1 | phospholipase A1-IIdelta | 1537 | 0 | 76.25 |
| TRINITY_DN19834_c0_g2_i2 | phospholipase A1-IIdelta | 1537 | 0 | 76.25 |
| TRINITY_DN1983_c0_g1_i1 | tRNA wybutosine-synthesizing 4 | 1498 | 0 | 87.65 |
| TRINITY_DN19841_c0_g1_i1 | COFACTOR ASSEMBLY OF COMPLEX C SUBUNIT B chloroplastic | 882 | 3.24E-72 | 74.9 |
| TRINITY_DN1984_c0_g2_i1 | transcription factor bHLH13-like | 1001 | 5.36E-113 | 80.65 |
| TRINITY_DN1984_c1_g1_i1 | transcription factor bHLH13 | 772 | 1.07E-42 | 62.85 |
| TRINITY_DN19854_c1_g1_i1 | UV radiation resistance-associated gene isoform X1 | 626 | 1.81E-55 | 81.1 |
| TRINITY_DN19854_c1_g2_i1 | UV radiation resistance-associated gene isoform X1 | 1702 | 9.56E-176 | 78.6 |
| TRINITY_DN19858_c0_g1_i1 | stromal 70 kDa heat shock-related chloroplastic | 3482 | 0 | 93.15 |
| TRINITY_DN19858_c0_g1_i2 | stromal 70 kDa heat shock-related chloroplastic | 3476 | 0 | 93.45 |
| TRINITY_DN19863_c0_g1_i1 | microtubule-associated 70-5 | 2310 | 0 | 72.6 |
| TRINITY_DN19863_c0_g1_i2 | microtubule-associated 70-5 | 2547 | 0 | 72.55 |
| TRINITY_DN19863_c0_g1_i3 | microtubule-associated 70-5 | 2449 | 0 | 72.25 |
| TRINITY_DN19863_c0_g1_i4 | microtubule-associated 70-5 | 2212 | 0 | 72.3 |
| TRINITY_DN19882_c0_g1_i1 | guanine nucleotide exchange factor SPIKE 1 | 5965 | 0 | 89.5 |
| TRINITY_DN19888_c0_g1_i1 | pentatricopeptide repeat-containing At1g02150 | 2226 | 0 | 77.1 |
| TRINITY_DN19890_c0_g1_i1 | DA1-related 2 isoform X1 | 2083 | 0 | 85.1 |
| TRINITY_DN19891_c0_g1_i1 | ---NA--- | 1032 |  |  |
| TRINITY_DN19893_c2_g1_i1 | aquaporin PIP2-1-like | 775 | 1.32E-108 | 95.4 |
| TRINITY_DN19893_c2_g2_i1 | aquaporin PIP2-1-like | 775 | 1.32E-108 | 95.4 |
| TRINITY_DN19893_c3_g1_i1 | probable aquaporin PIP2-8 | 1018 | 6.36E-143 | 96.8 |
| TRINITY_DN19894_c0_g1_i1 | serine hydroxymethyltransferase 7-like | 2994 | 0 | 83.45 |
| TRINITY_DN19894_c0_g1_i2 | serine hydroxymethyltransferase 6 | 2105 | 0 | 87.9 |
| TRINITY_DN19894_c1_g1_i1 | ---NA--- | 247 |  |  |
| TRINITY_DN19896_c0_g1_i1 | dof zinc finger -like | 907 | 2.28E-33 | 81.15 |
| TRINITY_DN19899_c0_g2_i1 | MICAL C-terminal | 1311 | 1.04E-34 | 54.95 |
| TRINITY_DN19904_c0_g1_i1 | dnaJ homolog subfamily C GRV2 isoform X2 | 8283 | 0 | 87.15 |
| TRINITY_DN19904_c0_g1_i2 | dnaJ homolog subfamily C GRV2 isoform X1 | 8282 | 0 | 87.5 |
| TRINITY_DN19910_c0_g2_i1 | PREDICTED: uncharacterized protein LOC104901744 | 1836 | 0 | 70.85 |
| TRINITY_DN19914_c0_g1_i1 | mannosylglyco endo-beta-mannosidase | 3188 | 0 | 83.4 |
| TRINITY_DN19920_c0_g1_i1 | ethylene-responsive transcription factor ERF034 | 1223 | 3.01E-41 | 78.55 |
| TRINITY_DN19921_c0_g1_i1 | nardilysin-like isoform X1 | 3258 | 0 | 83.8 |
| TRINITY_DN19921_c0_g1_i2 | nardilysin-like isoform X1 | 3031 | 0 | 83.8 |
| TRINITY_DN19925_c0_g1_i1 | ---NA--- | 1537 |  |  |
| TRINITY_DN19925_c0_g1_i2 | ---NA--- | 1609 |  |  |
| TRINITY_DN19925_c0_g1_i3 | ---NA--- | 1693 |  |  |
| TRINITY_DN19928_c0_g2_i1 | ---NA--- | 1246 |  |  |
| TRINITY_DN19930_c0_g1_i1 | probable folate-biopterin transporter 2 | 1945 | 2.85E-115 | 80 |
| TRINITY_DN19930_c0_g1_i2 | probable folate-biopterin transporter 2 | 1691 | 0 | 81.6 |
| TRINITY_DN19930_c0_g1_i3 | probable folate-biopterin transporter 2 | 1549 | 3.38E-142 | 81.4 |
| TRINITY_DN19930_c0_g1_i4 | probable folate-biopterin transporter 2 | 1609 | 6.54E-174 | 82.2 |
| TRINITY_DN19931_c0_g1_i1 | E3 ubiquitin- ligase ATL31 | 1277 | 1.90E-63 | 61.75 |
| TRINITY_DN19931_c0_g1_i2 | ---NA--- | 357 |  |  |
| TRINITY_DN19932_c0_g1_i1 | ---NA--- | 1325 |  |  |
| TRINITY_DN19934_c0_g1_i1 | zinc finger CCCH domain-containing 1 | 1447 | 6.12E-155 | 82.7 |
| TRINITY_DN19935_c0_g1_i1 | Embryo defective isoform 2 | 537 | 3.11E-47 | 70.3 |
| TRINITY_DN19944_c0_g1_i1 | ---NA--- | 284 |  |  |
| TRINITY_DN19944_c0_g2_i1 | ---NA--- | 284 |  |  |
| TRINITY_DN19944_c1_g1_i1 | ---NA--- | 390 |  |  |
| TRINITY_DN19944_c1_g1_i2 | ---NA--- | 465 |  |  |
| TRINITY_DN19944_c2_g1_i1 | beta-hexosaminidase 3 | 1989 | 0 | 88.3 |
| TRINITY_DN19944_c2_g1_i2 | beta-hexosaminidase 3 isoform X3 | 1793 | 0 | 85.5 |
| TRINITY_DN19944_c3_g1_i1 | ---NA--- | 373 |  |  |
| TRINITY_DN19945_c0_g1_i3 | ---NA--- | 302 |  |  |
| TRINITY_DN19945_c0_g1_i4 | ---NA--- | 386 |  |  |
| TRINITY_DN19945_c0_g1_i6 | heparan-alpha-glucosaminide N-acetyltransferase-like | 575 | 2.99E-33 | 80.3 |
| TRINITY_DN19945_c0_g3_i1 | ---NA--- | 240 |  |  |
| TRINITY_DN19965_c0_g1_i1 | ---NA--- | 243 |  |  |
| TRINITY_DN19965_c0_g1_i2 | ---NA--- | 311 |  |  |
| TRINITY_DN19965_c0_g2_i1 | ---NA--- | 217 |  |  |
| TRINITY_DN19969_c0_g1_i1 | B-zip transcription isoform 2 | 1722 | 1.50E-88 | 72.55 |
| TRINITY_DN19969_c0_g1_i2 | B-zip transcription isoform 2 | 1775 | 1.10E-94 | 68.95 |
| TRINITY_DN19977_c0_g1_i1 | probable galacturonosyltransferase-like 7 | 1948 | 0 | 83.95 |
| TRINITY_DN19978_c0_g1_i1 | probable ADP,ATP carrier At5g56450 | 1894 | 5.74E-142 | 86.95 |
| TRINITY_DN19978_c0_g1_i2 | probable ADP,ATP carrier At5g56450 | 1790 | 0 | 85.9 |
| TRINITY_DN19980_c0_g1_i1 | probable arabinosyltransferase ARAD1 | 627 | 3.31E-86 | 89.1 |
| TRINITY_DN19980_c0_g2_i1 | probable arabinosyltransferase ARAD1 | 1895 | 0 | 81.25 |
| TRINITY_DN19994_c0_g1_i1 | mediator of RNA polymerase II transcription subunit 27 | 1635 | 6.88E-175 | 75.7 |
| TRINITY_DN19994_c0_g1_i2 | mediator of RNA polymerase II transcription subunit 27 | 1648 | 3.35E-147 | 78.4 |
| TRINITY_DN19994_c0_g1_i3 | mediator of RNA polymerase II transcription subunit 27 | 1622 | 0 | 77 |
| TRINITY_DN19994_c0_g1_i4 | mediator of RNA polymerase II transcription subunit 27 | 1635 | 0 | 79.3 |
| TRINITY_DN19997_c0_g1_i1 | DEAD-box ATP-dependent RNA helicase 51 | 2156 | 0 | 91.25 |
| TRINITY_DN20000_c0_g3_i2 | BTB POZ domain-containing At1g04390 isoform X1 | 3192 | 0 | 66.95 |
| TRINITY_DN20004_c0_g1_i1 | probable inactive receptor kinase At1g27190 | 2170 | 0 | 74.05 |
| TRINITY_DN20004_c0_g1_i2 | probable inactive receptor kinase At1g27190 | 2136 | 0 | 74.05 |
| TRINITY_DN20007_c0_g2_i1 | ---NA--- | 587 |  |  |
| TRINITY_DN20007_c0_g2_i3 | ---NA--- | 899 |  |  |
| TRINITY_DN20010_c1_g1_i1 | serine threonine- kinase STY46 | 2277 | 0 | 85.15 |
| TRINITY_DN20010_c1_g1_i2 | serine threonine- kinase STY46 | 2365 | 0 | 85.8 |
| TRINITY_DN20014_c0_g1_i1 | ABC transporter B family member 1 | 4167 | 0 | 92.5 |
| TRINITY_DN20020_c0_g1_i1 | guanine nucleotide-binding subunit gamma 2-like | 1445 | 1.77E-42 | 76 |
| TRINITY_DN20039_c0_g1_i1 | 5 -nucleotidase -like | 480 | 4.92E-17 | 64.25 |
| TRINITY_DN20039_c0_g1_i2 | 5 -nucleotidase -like | 1736 | 0 | 77.95 |
| TRINITY_DN20039_c0_g1_i3 | 5 -nucleotidase -like | 1626 | 0 | 77.95 |
| TRINITY_DN20045_c0_g1_i1 | surfeit locus 6 homolog | 1369 | 7.76E-54 | 63.9 |
| TRINITY_DN20046_c0_g1_i1 | transmembrane emp24 domain-containing p24beta3 | 1013 | 1.79E-137 | 90.9 |
| TRINITY_DN20048_c0_g1_i1 | 40S ribosomal S9-2 | 1007 | 4.43E-121 | 96.95 |
| TRINITY_DN20054_c0_g1_i1 | Heme binding | 1443 | 0 | 81.3 |
| TRINITY_DN20057_c0_g1_i1 | MAP3K epsilon kinase 1-like | 2162 | 0 | 78.5 |
| TRINITY_DN20057_c0_g1_i2 | MAP3K epsilon kinase 1-like | 2056 | 0 | 91.05 |
| TRINITY_DN20057_c0_g2_i1 | MAP3K epsilon kinase 1-like | 311 | 5.23E-22 | 95.3 |
| TRINITY_DN20059_c0_g1_i1 | nucleolar 56-like | 2265 | 0 | 90.9 |
| TRINITY_DN20059_c0_g1_i2 | nucleolar 56-like | 2265 | 0 | 91.05 |
| TRINITY_DN20060_c1_g1_i2 | fructose-bisphosphate aldolase cytoplasmic | 1456 | 0 | 91.05 |
| TRINITY_DN20060_c1_g2_i1 | fructose-bisphosphate cytoplasmic isozyme | 1456 | 0 | 91.45 |
| TRINITY_DN20060_c2_g1_i1 | Nuclear receptor corepressor 1 | 5235 | 0 | 64.85 |
| TRINITY_DN20063_c0_g1_i1 | Intracellular transport USO1 isoform 3 | 1472 | 9.70E-141 | 73.5 |
| TRINITY_DN20066_c0_g1_i1 | ---NA--- | 325 |  |  |
| TRINITY_DN20066_c0_g1_i2 | ---NA--- | 369 |  |  |
| TRINITY_DN20066_c0_g1_i3 | ---NA--- | 325 |  |  |
| TRINITY_DN20066_c0_g1_i4 | ---NA--- | 369 |  |  |
| TRINITY_DN20074_c0_g1_i1 | Polyadenylate-binding 2-binding 2 | 1054 | 4.06E-93 | 90 |
| TRINITY_DN20074_c0_g1_i2 | Polyadenylate-binding 2-binding 2 | 1004 | 2.33E-93 | 90 |
| TRINITY_DN20076_c0_g1_i1 | probable ribosome-binding factor chloroplastic | 1190 | 7.32E-101 | 83.5 |
| TRINITY_DN20079_c0_g1_i2 | calcium-dependent kinase 2-like | 2727 | 0 | 90.85 |
| TRINITY_DN20079_c0_g1_i4 | calcium-dependent kinase 2-like | 2627 | 0 | 90.85 |
| TRINITY_DN2007_c0_g1_i1 | nucleobase-ascorbate transporter 3 | 914 | 2.21E-69 | 86.7 |
| TRINITY_DN2007_c0_g2_i1 | nucleobase-ascorbate transporter 3 | 827 | 8.16E-70 | 86.7 |
| TRINITY_DN20080_c0_g2_i1 | ---NA--- | 1020 |  |  |
| TRINITY_DN20081_c1_g1_i1 | 28 kDa chloroplastic | 996 | 2.20E-106 | 81.3 |
| TRINITY_DN20081_c2_g1_i1 | 33 kDa chloroplastic | 1128 | 2.07E-102 | 76.35 |
| TRINITY_DN20093_c0_g2_i2 | pentatricopeptide repeat-containing At4g04370 | 1029 | 1.78E-165 | 76.35 |
| TRINITY_DN20098_c0_g1_i1 | PREDICTED: uncharacterized protein LOC104889462 isoform X1 | 1665 | 1.07E-121 | 67.3 |
| TRINITY_DN20098_c0_g1_i2 | hypothetical protein SOVF_002010 | 1737 | 6.55E-118 | 66 |
| TRINITY_DN2009_c0_g1_i1 | ---NA--- | 679 |  |  |
| TRINITY_DN2009_c0_g2_i1 | ---NA--- | 283 |  |  |
| TRINITY_DN20102_c0_g1_i1 | PREDICTED: uncharacterized protein LOC18607943 | 541 | 5.57E-06 | 83.5 |
| TRINITY_DN20102_c0_g2_i2 | DUF1685 domain-containing | 971 | 2.24E-38 | 66.3 |
| TRINITY_DN20102_c2_g2_i1 | phenazine biosynthesis family | 1757 | 1.43E-98 | 66.85 |
| TRINITY_DN20102_c2_g3_i1 | phenazine biosynthesis family | 1745 | 1.28E-98 | 66.85 |
| TRINITY_DN20102_c2_g5_i1 | phenazine biosynthesis family | 1792 | 2.00E-98 | 66.85 |
| TRINITY_DN20102_c3_g1_i1 | hypothetical protein SOVF_022370 | 518 | 1.44E-11 | 90 |
| TRINITY_DN20110_c0_g1_i1 | SCARECROW | 2131 | 0 | 91.85 |
| TRINITY_DN20113_c0_g1_i1 | transcription factor TCP4-like | 1269 | 1.07E-58 | 51.75 |
| TRINITY_DN20113_c0_g2_i1 | transcription factor TCP4-like | 1266 | 1.26E-60 | 53.3 |
| TRINITY_DN20113_c0_g2_i2 | transcription factor TCP4-like | 1050 | 2.02E-50 | 53.75 |
| TRINITY_DN20113_c2_g1_i1 | MFP1 attachment factor 1-like | 970 | 1.94E-26 | 74.45 |
| TRINITY_DN20113_c2_g2_i1 | MFP1 attachment factor 1-like | 775 | 4.81E-27 | 74.2 |
| TRINITY_DN20113_c2_g3_i1 | MFP1 attachment factor 1-like | 976 | 2.01E-26 | 74.45 |
| TRINITY_DN20113_c2_g4_i1 | MFP1 attachment factor 1-like | 964 | 1.87E-26 | 74.45 |
| TRINITY_DN20113_c2_g5_i1 | ---NA--- | 242 |  |  |
| TRINITY_DN20113_c2_g6_i1 | MFP1 attachment factor 1-like | 967 | 1.91E-26 | 74.45 |
| TRINITY_DN20113_c2_g7_i1 | MFP1 attachment factor 1-like | 973 | 1.98E-26 | 74.45 |
| TRINITY_DN20113_c3_g1_i1 | UPF0426 chloroplastic | 565 | 9.11E-37 | 80.95 |
| TRINITY_DN20116_c0_g1_i1 | mediator of RNA polymerase II transcription subunit 10b | 779 | 4.64E-87 | 89.55 |
| TRINITY_DN20116_c0_g1_i2 | mediator of RNA polymerase II transcription subunit 10b | 890 | 1.76E-86 | 89.55 |
| TRINITY_DN20122_c0_g1_i1 | KDPG and KHG aldolase | 1493 | 5.60E-75 | 79.55 |
| TRINITY_DN20122_c0_g1_i6 | KHG KDPG aldolase-like isoform X1 | 1525 | 2.12E-64 | 84.55 |
| TRINITY_DN20122_c1_g1_i1 | ---NA--- | 282 |  |  |
| TRINITY_DN20126_c0_g1_i1 | SWI SNF-related matrix-associated actin-dependent regulator of chromatin subfamily A member 3-like 2 | 665 | 2.36E-16 | 90.15 |
| TRINITY_DN20126_c0_g1_i3 | SWI SNF-related matrix-associated actin-dependent regulator of chromatin subfamily A member 3-like 2 | 1289 | 0 | 92.1 |
| TRINITY_DN20131_c0_g1_i1 | ---NA--- | 552 |  |  |
| TRINITY_DN20131_c0_g1_i2 | ---NA--- | 1454 |  |  |
| TRINITY_DN20133_c0_g1_i1 | heat shock binding | 1713 | 4.86E-95 | 80.1 |
| TRINITY_DN20134_c0_g1_i1 | probable auxin efflux carrier component 1c | 1072 | 0 | 87.65 |
| TRINITY_DN20134_c0_g2_i1 | probable auxin efflux carrier component 1c | 266 | 6.04E-10 | 61.43 |
| TRINITY_DN20137_c1_g1_i1 | ---NA--- | 587 |  |  |
| TRINITY_DN20137_c1_g2_i1 | ---NA--- | 400 |  |  |
| TRINITY_DN20138_c0_g1_i1 | mechanosensitive ion channel chloroplastic-like | 2459 | 0 | 78.15 |
| TRINITY_DN20146_c0_g1_i1 | lactosylceramide 4-alpha-galactosyltransferase-like | 1870 | 6.12E-148 | 70.7 |
| TRINITY_DN20149_c0_g1_i2 | serine threonine- kinase SRK2E | 1608 | 0 | 96 |
| TRINITY_DN20150_c0_g1_i2 | ---NA--- | 732 |  |  |
| TRINITY_DN20150_c0_g1_i4 | ---NA--- | 910 |  |  |
| TRINITY_DN20152_c0_g1_i1 | heat stress transcription factor A-2c | 1360 | 1.03E-12 | 58.5 |
| TRINITY_DN20154_c0_g1_i3 | CSC1 At3g54510 | 1910 | 0 | 77.9 |
| TRINITY_DN20157_c0_g1_i1 | ---NA--- | 557 |  |  |
| TRINITY_DN20161_c0_g1_i1 | ---NA--- | 565 |  |  |
| TRINITY_DN20161_c0_g2_i1 | ---NA--- | 218 |  |  |
| TRINITY_DN20169_c0_g2_i1 | ABC transporter | 2281 | 0 | 92.5 |
| TRINITY_DN20173_c1_g2_i2 | ---NA--- | 630 |  |  |
| TRINITY_DN20173_c1_g2_i3 | ---NA--- | 1531 |  |  |
| TRINITY_DN20173_c1_g2_i5 | ---NA--- | 448 |  |  |
| TRINITY_DN20173_c1_g2_i6 | ---NA--- | 1424 |  |  |
| TRINITY_DN20173_c1_g2_i7 | ---NA--- | 737 |  |  |
| TRINITY_DN20179_c1_g1_i1 | systemin receptor SR160 | 759 | 6.48E-34 | 70.1 |
| TRINITY_DN2017_c0_g1_i1 | dof zinc finger PBF | 229 | 5.30E-11 | 73 |
| TRINITY_DN2017_c0_g2_i1 | ---NA--- | 229 |  |  |
| TRINITY_DN20180_c0_g1_i1 | germin 2-1 | 749 | 4.74E-94 | 73.05 |
| TRINITY_DN2018_c0_g1_i1 | ---NA--- | 547 |  |  |
| TRINITY_DN2018_c0_g1_i2 | ---NA--- | 631 |  |  |
| TRINITY_DN20192_c0_g1_i1 | aspartic ase 1 isoform X2 | 1588 | 9.47E-166 | 76.75 |
| TRINITY_DN20192_c0_g1_i2 | aspartic ase 1 isoform X1 | 1883 | 0 | 73.45 |
| TRINITY_DN20192_c0_g1_i3 | aspartic ase 1 isoform X1 | 1554 | 0 | 75.3 |
| TRINITY_DN20192_c0_g1_i4 | aspartic ase 1 isoform X1 | 1917 | 1.48E-177 | 73.75 |
| TRINITY_DN20201_c0_g1_i1 | EARLY FLOWERING 3 | 1327 | 4.13E-50 | 51.3 |
| TRINITY_DN20202_c0_g1_i1 | kinesin KIN-7E isoform X1 | 804 | 5.67E-134 | 88.6 |
| TRINITY_DN20202_c0_g1_i2 | kinesin KIN-7E isoform X1 | 420 | 4.87E-46 | 85 |
| TRINITY_DN20203_c0_g1_i1 | GDP-Man:Man(3) c(2)-PP-Dol alpha-1,2-mannosyltransferase | 1673 | 0 | 89.05 |
| TRINITY_DN20203_c0_g1_i2 | GDP-Man:Man(3) c(2)-PP-Dol alpha-1,2-mannosyltransferase | 1702 | 0 | 89.2 |
| TRINITY_DN20203_c0_g1_i3 | GDP-Man:Man(3) c(2)-PP-Dol alpha-1,2-mannosyltransferase | 1072 | 1.34E-100 | 86.45 |
| TRINITY_DN20204_c0_g1_i1 | gamma aminobutyrate transaminase chloroplastic | 829 | 1.87E-51 | 92.4 |
| TRINITY_DN20207_c0_g1_i2 | ---NA--- | 2136 |  |  |
| TRINITY_DN20207_c0_g1_i4 | ---NA--- | 744 |  |  |
| TRINITY_DN20209_c0_g1_i1 | short-chain dehydrogenase TIC chloroplastic-like | 1354 | 6.97E-130 | 84.9 |
| TRINITY_DN20211_c0_g1_i1 | galactinol synthase 2 | 1354 | 0 | 87.1 |
| TRINITY_DN20222_c0_g1_i1 | probable galactinol--sucrose galactosyltransferase 6 | 2905 | 0 | 87 |
| TRINITY_DN20224_c0_g1_i1 | ---NA--- | 281 |  |  |
| TRINITY_DN20224_c0_g1_i2 | ---NA--- | 378 |  |  |
| TRINITY_DN20224_c0_g1_i3 | ---NA--- | 872 |  |  |
| TRINITY_DN20224_c0_g2_i1 | ---NA--- | 307 |  |  |
| TRINITY_DN20224_c0_g2_i2 | ---NA--- | 366 |  |  |
| TRINITY_DN20225_c0_g1_i1 | ---NA--- | 664 |  |  |
| TRINITY_DN20225_c1_g1_i1 | CBS domain-containing CBSCBSPB3 | 4632 | 0 | 85.35 |
| TRINITY_DN20225_c1_g1_i2 | zinc finger CCCH domain-containing 44 isoform X1 | 1811 | 0 | 61.9 |
| TRINITY_DN20225_c1_g1_i3 | zinc finger CCCH domain-containing 44 isoform X1 | 1941 | 0 | 61.5 |
| TRINITY_DN20225_c1_g1_i4 | zinc finger CCCH domain-containing 44 isoform X1 | 1867 | 0 | 61.5 |
| TRINITY_DN20225_c1_g1_i5 | CBS domain-containing CBSCBSPB3 | 4505 | 0 | 85.05 |
| TRINITY_DN20225_c1_g1_i6 | CBS domain-containing CBSCBSPB3 | 4558 | 0 | 85.35 |
| TRINITY_DN20225_c2_g1_i1 | DUF2358 domain-containing | 914 | 3.88E-114 | 78.95 |
| TRINITY_DN20226_c0_g1_i1 | glucan endo-1,3-beta-glucosidase | 1579 | 0 | 72.35 |
| TRINITY_DN20226_c0_g1_i2 | glucan endo-1,3-beta-glucosidase | 1455 | 0 | 72.6 |
| TRINITY_DN20227_c0_g1_i1 | SPT2 homolog | 2431 | 1.43E-43 | 56.1 |
| TRINITY_DN20227_c0_g1_i2 | SPT2 homolog isoform X1 | 2433 | 5.53E-67 | 50.9 |
| TRINITY_DN20228_c0_g1_i1 | reticulon B12 | 1075 | 3.32E-89 | 79.8 |
| TRINITY_DN20229_c0_g1_i3 | WD repeat-containing 13 | 1917 | 0 | 84.2 |
| TRINITY_DN20229_c0_g1_i5 | WD repeat-containing 13 | 1508 | 0 | 84.3 |
| TRINITY_DN20231_c0_g1_i1 | probable S-acyltransferase 19 | 619 | 6.80E-50 | 70.95 |
| TRINITY_DN20231_c0_g1_i2 | probable S-acyltransferase 19 | 882 | 1.59E-77 | 73.65 |
| TRINITY_DN20231_c0_g1_i3 | probable S-acyltransferase 19 | 2144 | 0 | 77.75 |
| TRINITY_DN20234_c0_g1_i1 | WD repeat-containing 43-like isoform X1 | 2092 | 0 | 74.7 |
| TRINITY_DN20234_c0_g1_i2 | WD repeat-containing 43-like isoform X1 | 2359 | 0 | 74.7 |
| TRINITY_DN20239_c0_g1_i1 | GPI-anchor transamidase | 2069 | 0 | 84.95 |
| TRINITY_DN20239_c0_g1_i2 | GPI-anchor transamidase | 2050 | 0 | 84.95 |
| TRINITY_DN20240_c0_g1_i1 | uncharacterized serine-rich | 1317 | 2.88E-36 | 70.9 |
| TRINITY_DN20240_c0_g1_i2 | uncharacterized serine-rich | 1338 | 2.76E-40 | 70.8 |
| TRINITY_DN20240_c0_g2_i1 | uncharacterized serine-rich | 1340 | 3.34E-36 | 70.9 |
| TRINITY_DN20240_c0_g2_i2 | uncharacterized serine-rich | 1361 | 3.18E-40 | 70.8 |
| TRINITY_DN20242_c0_g1_i1 | GATA zinc finger domain-containing 14 isoform X1 | 1750 | 2.85E-117 | 57 |
| TRINITY_DN20246_c0_g1_i1 | ---NA--- | 317 |  |  |
| TRINITY_DN20246_c0_g2_i1 | ---NA--- | 665 |  |  |
| TRINITY_DN20246_c1_g1_i1 | cyclin-D1-1 isoform X1 | 1641 | 1.39E-120 | 74.1 |
| TRINITY_DN20246_c1_g1_i2 | cyclin-D1-1 isoform X2 | 1696 | 1.44E-110 | 79.7 |
| TRINITY_DN20246_c1_g1_i3 | cyclin-D1-1 isoform X2 | 1708 | 2.87E-107 | 80.05 |
| TRINITY_DN20254_c0_g1_i1 | G-type lectin S-receptor-like serine threonine- kinase At1g34300 | 2242 | 0 | 79.5 |
| TRINITY_DN20254_c0_g1_i2 | G-type lectin S-receptor-like serine threonine- kinase At1g34300 | 2210 | 0 | 79.5 |
| TRINITY_DN20256_c0_g2_i2 | DNA mismatch repair MSH6 | 1368 | 7.53E-38 | 85.1 |
| TRINITY_DN20257_c0_g2_i1 | S-adenosyl-L-methionine-dependent methyltransferase Mjls_1072 | 1500 | 0 | 76.3 |
| TRINITY_DN20257_c0_g2_i2 | S-adenosyl-L-methionine-dependent methyltransferase Mjls_1072 | 1476 | 0 | 78.85 |
| TRINITY_DN20258_c0_g1_i2 | DEAD-box ATP-dependent RNA helicase 20 | 1828 | 0 | 91.8 |
| TRINITY_DN20259_c0_g1_i1 | hypothetical protein SOVF_090400 | 638 | 9.21E-14 | 52.5 |
| TRINITY_DN20259_c1_g2_i1 | chloroplastic | 3988 | 5.84E-173 | 69.95 |
| TRINITY_DN20264_c0_g1_i1 | C-type lectin receptor-like tyrosine- kinase At1g52310 | 2067 | 0 | 81.7 |
| TRINITY_DN20268_c0_g2_i1 | ARM repeat superfamily isoform 1 | 3126 | 0 | 54.85 |
| TRINITY_DN20269_c0_g2_i1 | ATP-dependent RNA helicase chloroplastic-like | 4431 | 0 | 73.5 |
| TRINITY_DN20269_c0_g2_i2 | ATP-dependent RNA helicase chloroplastic-like | 2402 | 0 | 71.7 |
| TRINITY_DN20269_c0_g2_i3 | ATP-dependent RNA helicase chloroplastic-like | 2321 | 0 | 73.45 |
| TRINITY_DN20269_c0_g2_i4 | ATP-dependent RNA helicase chloroplastic-like | 4512 | 0 | 72.65 |
| TRINITY_DN20269_c1_g1_i1 | ---NA--- | 238 |  |  |
| TRINITY_DN2026_c0_g1_i1 | Chromo domain-containing | 344 | 2.96E-12 | 68.85 |
| TRINITY_DN20270_c0_g1_i1 | embryogenesis-associated EMB8 | 1799 | 0 | 77.8 |
| TRINITY_DN20270_c0_g1_i3 | embryogenesis-associated EMB8 isoform X1 | 2033 | 0 | 77.7 |
| TRINITY_DN20273_c0_g1_i1 | spermidine synthase 1 | 1134 | 0 | 93.95 |
| TRINITY_DN20273_c0_g1_i2 | spermidine synthase 1 | 1108 | 6.86E-157 | 93.95 |
| TRINITY_DN20278_c0_g2_i1 | ---NA--- | 671 |  |  |
| TRINITY_DN20280_c0_g1_i1 | ENHANCER OF AG-4 2 isoform X1 | 3079 | 0 | 67.4 |
| TRINITY_DN20280_c0_g1_i2 | ENHANCER OF AG-4 2 isoform X1 | 3195 | 0 | 67.4 |
| TRINITY_DN20280_c0_g1_i3 | ENHANCER OF AG-4 2 isoform X1 | 2910 | 0 | 67.4 |
| TRINITY_DN20283_c0_g1_i1 | zinc finger with UFM1-specific peptidase domain isoform X1 | 1530 | 0 | 67.2 |
| TRINITY_DN20283_c0_g1_i2 | zinc finger with UFM1-specific peptidase domain isoform X1 | 1702 | 0 | 67.2 |
| TRINITY_DN20283_c0_g1_i3 | zinc finger with UFM1-specific peptidase domain isoform X1 | 1578 | 0 | 67.2 |
| TRINITY_DN20283_c0_g1_i4 | zinc finger with UFM1-specific peptidase domain isoform X1 | 1607 | 0 | 67.2 |
| TRINITY_DN20283_c0_g1_i6 | zinc finger with UFM1-specific peptidase domain isoform X1 | 1250 | 1.74E-173 | 66 |
| TRINITY_DN20283_c0_g3_i1 | ---NA--- | 213 |  |  |
| TRINITY_DN20287_c0_g1_i1 | ---NA--- | 328 |  |  |
| TRINITY_DN20287_c0_g2_i1 | tRNA-specific adenosine deaminase 2 | 1048 | 7.00E-102 | 86.1 |
| TRINITY_DN20293_c0_g1_i1 | histone acetyltransferase HAC12 | 4539 | 0 | 64.6 |
| TRINITY_DN20298_c0_g2_i1 | protease Do-like 14 isoform X1 | 1146 | 7.68E-71 | 63 |
| TRINITY_DN20302_c0_g1_i1 | ---NA--- | 811 |  |  |
| TRINITY_DN20302_c0_g1_i2 | ---NA--- | 1606 |  |  |
| TRINITY_DN20323_c0_g1_i1 | MAR-binding filament 1-1 isoform X2 | 2971 | 4.31E-146 | 68.35 |
| TRINITY_DN20323_c0_g2_i1 | ycf49 | 1382 | 1.36E-61 | 88.65 |
| TRINITY_DN20324_c1_g1_i1 | PREDICTED: uncharacterized protein LOC104883979 | 1375 | 1.13E-52 | 56.6 |
| TRINITY_DN20324_c1_g1_i2 | PREDICTED: uncharacterized protein LOC104883979 | 1295 | 3.34E-49 | 56.6 |
| TRINITY_DN20324_c1_g1_i3 | PREDICTED: uncharacterized protein LOC104883979 | 1229 | 1.93E-49 | 56.6 |
| TRINITY_DN20324_c1_g1_i4 | PREDICTED: uncharacterized protein LOC104883979 | 1441 | 7.99E-49 | 56.45 |
| TRINITY_DN20324_c1_g1_i5 | PREDICTED: uncharacterized protein LOC104883979 | 1514 | 3.17E-52 | 56.6 |
| TRINITY_DN20325_c0_g1_i1 | ase inhibitor serpin emp24 gp25L p24 | 761 | 1.16E-46 | 86.75 |
| TRINITY_DN2032_c0_g1_i1 | endoplasmic reticulum-Golgi intermediate compartment 3 | 615 | 2.33E-82 | 96.95 |
| TRINITY_DN20337_c0_g1_i1 | G-box-binding factor 4 | 1130 | 1.41E-29 | 93.7 |
| TRINITY_DN20337_c0_g1_i3 | G-box-binding factor 4 | 1413 | 6.02E-29 | 93.7 |
| TRINITY_DN20343_c0_g1_i2 | auxin response factor 9 | 2377 | 0 | 70.2 |
| TRINITY_DN20343_c0_g1_i5 | auxin response factor 9 | 2318 | 0 | 63 |
| TRINITY_DN20350_c1_g2_i1 | ---NA--- | 418 |  |  |
| TRINITY_DN20350_c1_g2_i2 | ---NA--- | 647 |  |  |
| TRINITY_DN20350_c1_g2_i3 | ---NA--- | 345 |  |  |
| TRINITY_DN20355_c0_g1_i1 | probable LRR receptor-like serine threonine- kinase At2g16250 | 3013 | 0 | 77.65 |
| TRINITY_DN20361_c0_g1_i1 | DA1-related 1 isoform X1 | 2037 | 0 | 75.1 |
| TRINITY_DN20361_c0_g1_i2 | DA1-related 1-like | 1969 | 0 | 74.85 |
| TRINITY_DN20361_c0_g1_i3 | DA1-related 1-like | 1983 | 0 | 74.85 |
| TRINITY_DN20361_c0_g1_i4 | DA1-related 1 isoform X1 | 2023 | 0 | 75.1 |
| TRINITY_DN20365_c0_g1_i1 | ---NA--- | 327 |  |  |
| TRINITY_DN20365_c0_g2_i1 | ---NA--- | 908 |  |  |
| TRINITY_DN20371_c0_g1_i10 | ---NA--- | 1045 |  |  |
| TRINITY_DN20371_c0_g1_i2 | ---NA--- | 1422 |  |  |
| TRINITY_DN20371_c0_g1_i3 | ---NA--- | 1561 |  |  |
| TRINITY_DN20371_c0_g1_i5 | ---NA--- | 1283 |  |  |
| TRINITY_DN20371_c0_g1_i8 | ---NA--- | 1323 |  |  |
| TRINITY_DN20372_c0_g1_i1 | transmembrane 45B | 1821 | 1.25E-167 | 85.55 |
| TRINITY_DN20373_c0_g1_i1 | pentatricopeptide repeat-containing chloroplastic | 2270 | 0 | 91.5 |
| TRINITY_DN20373_c0_g1_i2 | pentatricopeptide repeat-containing chloroplastic | 2343 | 0 | 88.8 |
| TRINITY_DN20376_c1_g1_i1 | petal death | 880 | 1.54E-168 | 86 |
| TRINITY_DN20376_c1_g1_i2 | petal death | 1263 | 0 | 84.9 |
| TRINITY_DN20382_c0_g1_i1 | MACPF domain-containing CAD1 | 1040 | 5.79E-45 | 56.85 |
| TRINITY_DN20386_c0_g1_i3 | 5 -adenylylsulfate reductase chloroplastic-like | 1629 | 0 | 87.3 |
| TRINITY_DN20386_c0_g1_i4 | 5 -adenylylsulfate reductase chloroplastic-like | 1545 | 0 | 91.8 |
| TRINITY_DN20387_c0_g1_i1 | kinesin KIFC3 | 971 | 4.68E-99 | 73.2 |
| TRINITY_DN20387_c0_g1_i2 | kinesin KIFC3 | 2031 | 0 | 74.8 |
| TRINITY_DN20387_c0_g1_i3 | kinesin KIFC3 | 2015 | 0 | 71.5 |
| TRINITY_DN2038_c0_g1_i1 | probable NADH dehydrogenase [ubiquinone] 1 alpha subcomplex subunit mitochondrial | 807 | 8.17E-100 | 90.2 |
| TRINITY_DN2038_c0_g2_i1 | probable NADH dehydrogenase [ubiquinone] 1 alpha subcomplex subunit mitochondrial | 807 | 2.35E-98 | 89.7 |
| TRINITY_DN20391_c0_g1_i1 | MADS box interactor | 1080 | 5.04E-79 | 76.25 |
| TRINITY_DN20391_c0_g1_i2 | MADS box interactor | 861 | 5.68E-55 | 72.7 |
| TRINITY_DN20391_c0_g1_i3 | MADS box interactor | 772 | 8.31E-48 | 70.6 |
| TRINITY_DN20391_c0_g1_i4 | MADS box interactor | 1169 | 2.44E-86 | 77.2 |
| TRINITY_DN20391_c0_g1_i5 | MADS box interactor | 1207 | 3.05E-65 | 71.1 |
| TRINITY_DN20392_c1_g2_i1 | serine threonine- phosphatase 7 long form homolog | 2308 | 0 | 79.3 |
| TRINITY_DN20396_c0_g1_i1 | Mitochondrial ATP synthase subunit G | 736 | 7.72E-72 | 88.15 |
| TRINITY_DN20396_c0_g1_i2 | Mitochondrial ATP synthase subunit G | 729 | 4.92E-73 | 89.05 |
| TRINITY_DN20404_c0_g1_i1 | ---NA--- | 986 |  |  |
| TRINITY_DN20404_c0_g1_i3 | ---NA--- | 758 |  |  |
| TRINITY_DN20404_c0_g1_i4 | ---NA--- | 798 |  |  |
| TRINITY_DN20404_c0_g1_i5 | ---NA--- | 617 |  |  |
| TRINITY_DN2040_c0_g1_i1 | Maternal effect embryo arrest 59 | 955 | 8.49E-75 | 77.25 |
| TRINITY_DN20410_c1_g1_i1 | ---NA--- | 253 |  |  |
| TRINITY_DN20410_c1_g2_i1 | reverse transcriptase | 2085 | 6.09E-77 | 59.95 |
| TRINITY_DN20414_c0_g2_i1 | chloride channel CLC-b | 1297 | 9.47E-123 | 83.7 |
| TRINITY_DN20414_c0_g2_i2 | chloride channel CLC-b | 1150 | 2.90E-114 | 83.75 |
| TRINITY_DN20414_c0_g2_i3 | chloride channel CLC-b | 1089 | 8.57E-128 | 82.65 |
| TRINITY_DN20414_c0_g2_i4 | chloride channel CLC-b | 1236 | 1.69E-159 | 80.25 |
| TRINITY_DN2041_c0_g1_i1 | probable E3 ubiquitin- ligase LUL4 | 1368 | 3.14E-138 | 72.05 |
| TRINITY_DN20421_c1_g1_i1 | ---NA--- | 1357 |  |  |
| TRINITY_DN20421_c1_g2_i1 | ---NA--- | 1738 |  |  |
| TRINITY_DN20421_c1_g2_i2 | ---NA--- | 1836 |  |  |
| TRINITY_DN20421_c1_g2_i3 | ---NA--- | 2122 |  |  |
| TRINITY_DN20423_c0_g1_i1 | ubiquitin carboxyl-terminal hydrolase 23-like | 2886 | 0 | 72.95 |
| TRINITY_DN20425_c0_g1_i1 | tRNA-splicing endonuclease subunit Sen54-like isoform X1 | 1509 | 1.25E-67 | 57.8 |
| TRINITY_DN20425_c0_g1_i2 | tRNA-splicing endonuclease subunit Sen54-like isoform X1 | 1163 | 5.91E-54 | 65.1 |
| TRINITY_DN20425_c0_g1_i3 | tRNA-splicing endonuclease subunit Sen54-like isoform X1 | 1569 | 1.14E-81 | 63.1 |
| TRINITY_DN20436_c0_g1_i1 | SMR domain-containing At5g58720 | 1915 | 2.25E-166 | 64.75 |
| TRINITY_DN20440_c0_g1_i2 | clustered mitochondria | 3728 | 0 | 81.55 |
| TRINITY_DN20449_c0_g1_i1 | CRT (chloroquine-resistance transporter)-like | 1709 | 1.58E-174 | 85.25 |
| TRINITY_DN20449_c0_g1_i2 | chloroplastic | 1616 | 5.07E-147 | 72.7 |
| TRINITY_DN20452_c0_g1_i1 | ---NA--- | 750 |  |  |
| TRINITY_DN20452_c0_g3_i1 | ---NA--- | 609 |  |  |
| TRINITY_DN20462_c0_g1_i1 | GTP-binding 6 | 2164 | 0 | 92.7 |
| TRINITY_DN20467_c0_g1_i1 | peptidyl-prolyl cis-trans isomerase FKBP53 | 1733 | 7.55E-80 | 67.25 |
| TRINITY_DN20469_c0_g1_i1 | ribosome biogenesis GTPase | 1825 | 0 | 74.1 |
| TRINITY_DN20470_c0_g1_i1 | E3 ubiquitin- ligase RBBP6 isoform X1 | 868 | 1.52E-148 | 83.05 |
| TRINITY_DN20470_c0_g2_i1 | E3 ubiquitin- ligase RBBP6 isoform X2 | 2271 | 0 | 80.9 |
| TRINITY_DN20473_c0_g1_i2 | hypothetical protein SOVF_151420 | 1324 | 3.77E-09 | 65.5 |
| TRINITY_DN20473_c0_g1_i3 | hypothetical protein SOVF_151420 | 1068 | 2.72E-09 | 65.5 |
| TRINITY_DN20473_c0_g1_i4 | PREDICTED: uncharacterized protein LOC104887776 | 1363 | 8.00E-29 | 58.11 |
| TRINITY_DN20473_c1_g1_i1 | ---NA--- | 492 |  |  |
| TRINITY_DN20473_c1_g2_i1 | ---NA--- | 486 |  |  |
| TRINITY_DN20477_c0_g1_i2 | ankyrin repeat-containing At5g02620-like | 1878 | 0 | 87.05 |
| TRINITY_DN2047_c0_g1_i1 | Ubiquitin-conjugating enzyme E2 variant 1C | 250 | 7.84E-19 | 100 |
| TRINITY_DN20482_c0_g2_i1 | syntaxin-22 | 1917 | 1.29E-143 | 93 |
| TRINITY_DN20488_c0_g1_i1 | WVD2-like 5 isoform X1 | 2266 | 1.02E-108 | 60.7 |
| TRINITY_DN20488_c0_g1_i2 | WVD2-like 5 isoform X1 | 2278 | 1.13E-108 | 60.7 |
| TRINITY_DN20494_c0_g1_i1 | ---NA--- | 532 |  |  |
| TRINITY_DN20494_c0_g2_i1 | ---NA--- | 531 |  |  |
| TRINITY_DN20494_c1_g1_i1 | ---NA--- | 341 |  |  |
| TRINITY_DN20494_c1_g1_i2 | LURP-one-related 11 | 1009 | 2.43E-88 | 67.15 |
| TRINITY_DN20494_c1_g1_i3 | LURP-one-related 11-like | 341 | 3.85E-32 | 77.7 |
| TRINITY_DN20512_c0_g1_i1 | F-box At3g07870 | 250 | 6.80E-09 | 81 |
| TRINITY_DN20512_c0_g3_i1 | F-box At3g07870-like | 1107 | 3.66E-139 | 51.5 |
| TRINITY_DN20514_c0_g1_i1 | cycloartenol-C-24-methyltransferase | 1469 | 0 | 93.3 |
| TRINITY_DN20514_c0_g1_i2 | cycloartenol-C-24-methyltransferase | 1550 | 0 | 86.6 |
| TRINITY_DN20514_c0_g1_i5 | cycloartenol-C-24-methyltransferase | 753 | 1.97E-113 | 96 |
| TRINITY_DN20518_c0_g1_i2 | probable folate-biopterin transporter 3 | 1692 | 0 | 82 |
| TRINITY_DN20519_c0_g1_i1 | hypothetical protein CCACVL1_26267 | 1181 | 3.43E-12 | 62.38 |
| TRINITY_DN20519_c0_g1_i4 | hypothetical protein CCACVL1_26267 | 856 | 1.52E-12 | 62.46 |
| TRINITY_DN20519_c0_g1_i5 | hypothetical protein CCACVL1_26267 | 797 | 1.18E-12 | 62.46 |
| TRINITY_DN20519_c0_g1_i6 | hypothetical protein CCACVL1_26267 | 1240 | 3.72E-12 | 62.33 |
| TRINITY_DN20530_c0_g2_i1 | cyclin-dependent kinase E-1 | 1931 | 0 | 85.7 |
| TRINITY_DN20530_c0_g2_i2 | cyclin-dependent kinase E-1 | 1985 | 0 | 87.65 |
| TRINITY_DN20530_c0_g2_i3 | cyclin-dependent kinase E-1 | 1864 | 0 | 88.1 |
| TRINITY_DN20530_c0_g2_i4 | cyclin-dependent kinase E-1 | 2343 | 0 | 86.5 |
| TRINITY_DN20539_c0_g1_i1 | ---NA--- | 216 |  |  |
| TRINITY_DN20539_c0_g2_i1 | B3 domain-containing transcription repressor VAL1 | 2962 | 0 | 65.5 |
| TRINITY_DN20539_c0_g2_i2 | B3 domain-containing transcription repressor VAL1 | 2979 | 0 | 65.7 |
| TRINITY_DN20545_c1_g3_i2 | zinc finger CCHC domain-containing 8 isoform X2 | 2134 | 5.08E-165 | 67.3 |
| TRINITY_DN20548_c0_g1_i1 | potassium transporter 5-like | 2527 | 0 | 81.05 |
| TRINITY_DN20556_c0_g1_i1 | 7-deoxyloganetic acid glucosyltransferase-like | 1190 | 4.21E-22 | 51.45 |
| TRINITY_DN20556_c0_g1_i2 | 7-deoxyloganetic acid glucosyltransferase-like | 1577 | 4.31E-13 | 66.5 |
| TRINITY_DN20556_c0_g1_i3 | 7-deoxyloganetic acid glucosyltransferase-like | 1099 | 1.43E-13 | 66.85 |
| TRINITY_DN20556_c0_g1_i4 | 7-deoxyloganetic acid glucosyltransferase-like | 1668 | 1.89E-21 | 51.45 |
| TRINITY_DN20557_c0_g1_i1 | SPA1-RELATED 2 isoform X1 | 4004 | 0 | 65.05 |
| TRINITY_DN20557_c0_g1_i2 | SPA1-RELATED 2 isoform X1 | 4017 | 0 | 65.05 |
| TRINITY_DN20566_c0_g2_i1 | ---NA--- | 332 |  |  |
| TRINITY_DN20566_c0_g3_i1 | DNA-directed RNA polymerase chloroplastic | 3627 | 0 | 85.55 |
| TRINITY_DN20569_c0_g1_i5 | phagocyte signaling-impaired isoform X1 | 3460 | 0 | 77.4 |
| TRINITY_DN20572_c0_g1_i1 | magnesium-protoporphyrin IX monomethyl ester [oxidative] chloroplastic | 1675 | 0 | 91 |
| TRINITY_DN20573_c0_g1_i1 | Cysteine-rich receptor kinase 25 | 343 | 1.25E-30 | 58.8 |
| TRINITY_DN20573_c0_g2_i1 | Cysteine-rich receptor kinase 29 | 791 | 8.74E-91 | 59.7 |
| TRINITY_DN20573_c0_g2_i2 | receptor kinase At4g00960 | 889 | 4.05E-94 | 59.4 |
| TRINITY_DN20574_c0_g1_i1 | ---NA--- | 539 |  |  |
| TRINITY_DN20574_c0_g1_i2 | ---NA--- | 621 |  |  |
| TRINITY_DN20576_c0_g2_i1 | mitogen-activated kinase homolog NTF3 | 1655 | 0 | 94.35 |
| TRINITY_DN20577_c1_g2_i2 | ---NA--- | 518 |  |  |
| TRINITY_DN20577_c1_g2_i4 | ---NA--- | 660 |  |  |
| TRINITY_DN20577_c1_g2_i6 | ---NA--- | 595 |  |  |
| TRINITY_DN20581_c0_g2_i1 | cyclin-dependent kinase inhibitor SMR6 | 729 | 6.05E-20 | 54.2 |
| TRINITY_DN20584_c1_g1_i1 | neuroguidin | 511 | 6.30E-38 | 67.45 |
| TRINITY_DN20585_c0_g1_i1 | CDPK-related kinase 1 | 2429 | 0 | 90.75 |
| TRINITY_DN20593_c0_g2_i1 | chlorophyll a-b binding chloroplastic-like | 1147 | 4.66E-176 | 92.25 |
| TRINITY_DN20593_c0_g2_i2 | chlorophyll a-b binding chloroplastic-like | 1175 | 0 | 94.4 |
| TRINITY_DN20597_c0_g1_i1 | ABC transporter I family member 20 | 1148 | 0 | 90 |
| TRINITY_DN20598_c0_g1_i1 | ---NA--- | 203 |  |  |
| TRINITY_DN20598_c1_g2_i1 | probable E3 ubiquitin- ligase RHA4A | 600 | 1.19E-49 | 70.6 |
| TRINITY_DN20598_c2_g2_i1 | probable E3 ubiquitin- ligase RHA4A | 829 | 8.48E-42 | 72.5 |
| TRINITY_DN20600_c0_g1_i2 | coiled-coil domain-containing 97 | 708 | 2.08E-41 | 81.8 |
| TRINITY_DN20600_c0_g1_i3 | auxin response factor 2 | 4011 | 0 | 77.75 |
| TRINITY_DN20600_c0_g1_i4 | auxin response factor 2 | 4024 | 0 | 77.75 |
| TRINITY_DN20607_c0_g1_i1 | eukaryotic translation initiation factor 4G | 5542 | 0 | 72.05 |
| TRINITY_DN20607_c1_g1_i1 | PREDICTED: uncharacterized protein LOC105042494 | 1607 | 1.10E-137 | 84.4 |
| TRINITY_DN20615_c0_g4_i1 | 60S ribosomal L18a isoform X1 | 885 | 1.07E-42 | 78.55 |
| TRINITY_DN20615_c0_g8_i4 | myosin-2 isoform X1 | 2056 | 3.25E-23 | 76.65 |
| TRINITY_DN20616_c0_g1_i1 | ---NA--- | 318 |  |  |
| TRINITY_DN20616_c0_g2_i1 | cyclin-dependent kinase G-2 isoform X1 | 1225 | 7.59E-119 | 96.65 |
| TRINITY_DN20616_c0_g2_i2 | cyclin-dependent kinase G-2 isoform X1 | 3003 | 0 | 71.6 |
| TRINITY_DN20616_c0_g2_i3 | cyclin-dependent kinase G-2 isoform X1 | 1139 | 9.02E-115 | 96.75 |
| TRINITY_DN20616_c0_g2_i4 | cyclin-dependent kinase G-2 isoform X1 | 1116 | 5.42E-141 | 96.7 |
| TRINITY_DN20616_c0_g2_i5 | cyclin-dependent kinase G-2 isoform X1 | 2980 | 0 | 75.25 |
| TRINITY_DN20617_c0_g1_i1 | ---NA--- | 638 |  |  |
| TRINITY_DN20617_c0_g1_i2 | ---NA--- | 520 |  |  |
| TRINITY_DN20617_c0_g1_i3 | ---NA--- | 505 |  |  |
| TRINITY_DN20617_c0_g2_i1 | ---NA--- | 257 |  |  |
| TRINITY_DN20622_c1_g3_i10 | ---NA--- | 780 |  |  |
| TRINITY_DN20622_c1_g3_i2 | hypothetical protein TSUD_01440, partial | 917 | 6.04E-07 | 80.33 |
| TRINITY_DN20622_c1_g3_i5 | hypothetical protein TSUD_01440, partial | 853 | 5.16E-07 | 80.33 |
| TRINITY_DN20622_c1_g3_i6 | ---NA--- | 669 |  |  |
| TRINITY_DN20622_c1_g3_i7 | hypothetical protein TSUD_01440, partial | 898 | 5.79E-07 | 80.33 |
| TRINITY_DN20622_c1_g3_i8 | ---NA--- | 825 |  |  |
| TRINITY_DN20622_c1_g3_i9 | ---NA--- | 844 |  |  |
| TRINITY_DN20622_c3_g1_i1 | ---NA--- | 490 |  |  |
| TRINITY_DN20624_c0_g3_i2 | DEAD-box ATP-dependent RNA helicase 37-like | 2615 | 0 | 93.55 |
| TRINITY_DN20634_c0_g1_i1 | bZIP transcription factor 53 | 924 | 2.34E-49 | 81.65 |
| TRINITY_DN20634_c0_g1_i2 | bZIP transcription factor 53 | 936 | 2.62E-49 | 81.65 |
| TRINITY_DN20634_c0_g1_i3 | bZIP transcription factor 53 | 684 | 1.75E-50 | 81.65 |
| TRINITY_DN20635_c1_g1_i1 | disulfide isomerase-like 1-6 | 1992 | 0 | 82.6 |
| TRINITY_DN20645_c0_g1_i1 | ---NA--- | 284 |  |  |
| TRINITY_DN20645_c1_g1_i1 | ---NA--- | 222 |  |  |
| TRINITY_DN20645_c1_g2_i1 | nuclear receptor corepressor 2 | 1933 | 2.15E-145 | 69.95 |
| TRINITY_DN20645_c1_g2_i2 | nuclear receptor corepressor 2 | 1656 | 9.37E-76 | 69.7 |
| TRINITY_DN20645_c1_g3_i1 | nuclear pore complex NUP58 | 229 | 1.91E-08 | 92.4 |
| TRINITY_DN20645_c1_g3_i2 | nuclear pore complex NUP58 | 1729 | 2.18E-147 | 82.5 |
| TRINITY_DN20645_c1_g3_i3 | nuclear pore complex NUP58 | 1632 | 7.42E-148 | 82.5 |
| TRINITY_DN20645_c1_g4_i1 | nuclear receptor corepressor 2 | 1656 | 2.76E-148 | 70.15 |
| TRINITY_DN20647_c0_g1_i1 | DUF810 domain-containing | 3357 | 0 | 79.45 |
| TRINITY_DN20647_c0_g1_i2 | DUF810 domain-containing | 3358 | 0 | 78.95 |
| TRINITY_DN20650_c0_g1_i1 | gibberellin 2-beta-dioxygenase 8 | 487 | 8.88E-40 | 66.05 |
| TRINITY_DN20650_c0_g2_i1 | gibberellin 2-beta-dioxygenase 8 | 788 | 1.76E-83 | 77.8 |
| TRINITY_DN20650_c0_g2_i2 | gibberellin 2-beta-dioxygenase 8 | 764 | 3.08E-71 | 72.45 |
| TRINITY_DN20650_c0_g2_i3 | gibberellin 2-beta-dioxygenase 8 | 799 | 1.12E-67 | 73.75 |
| TRINITY_DN20650_c0_g2_i6 | gibberellin 2-beta-dioxygenase 8-like | 776 | 4.97E-29 | 83.4 |
| TRINITY_DN20650_c0_g2_i7 | gibberellin 2-beta-dioxygenase 8 | 753 | 7.66E-94 | 76 |
| TRINITY_DN20650_c1_g1_i1 | ---NA--- | 303 |  |  |
| TRINITY_DN20657_c0_g2_i2 | probable leucine-rich repeat receptor kinase At2g33170 | 3627 | 0 | 82.05 |
| TRINITY_DN20658_c0_g1_i1 | homoserine kinase | 1488 | 3.30E-142 | 78.9 |
| TRINITY_DN20658_c1_g1_i1 | root UVB sensitive chloroplastic | 2959 | 0 | 82.9 |
| TRINITY_DN2065_c0_g1_i1 | CLP protease regulatory subunit mitochondrial | 668 | 7.01E-52 | 82.2 |
| TRINITY_DN20662_c0_g1_i1 | PREDICTED: uncharacterized protein LOC104883348 isoform X1 | 3401 | 0 | 72.95 |
| TRINITY_DN20662_c0_g1_i2 | PREDICTED: uncharacterized protein LOC104883348 isoform X6 | 3344 | 0 | 71.3 |
| TRINITY_DN20663_c0_g1_i1 | ---NA--- | 1007 |  |  |
| TRINITY_DN20663_c0_g2_i1 | ---NA--- | 912 |  |  |
| TRINITY_DN20663_c1_g1_i1 | ---NA--- | 777 |  |  |
| TRINITY_DN20664_c0_g2_i1 | ABC transporter B family member 9 | 1297 | 0 | 88.55 |
| TRINITY_DN20665_c0_g1_i1 | ---NA--- | 1097 |  |  |
| TRINITY_DN20665_c0_g1_i3 | ---NA--- | 880 |  |  |
| TRINITY_DN20674_c0_g1_i1 | ---NA--- | 325 |  |  |
| TRINITY_DN20674_c0_g2_i1 | ---NA--- | 284 |  |  |
| TRINITY_DN20674_c0_g3_i1 | ---NA--- | 961 |  |  |
| TRINITY_DN20677_c0_g2_i1 | persulfide dioxygenase ETHE1 mitochondrial-like isoform X1 | 1208 | 7.38E-163 | 90.85 |
| TRINITY_DN20677_c0_g2_i2 | persulfide dioxygenase ETHE1 mitochondrial-like isoform X1 | 1218 | 8.25E-163 | 90.85 |
| TRINITY_DN20680_c1_g2_i1 | ---NA--- | 1217 |  |  |
| TRINITY_DN20681_c0_g2_i1 | ALTERED XYLOGLUCAN 4 | 2084 | 0 | 81.05 |
| TRINITY_DN20689_c0_g1_i1 | phosphoglycerate mutase 4 | 1266 | 9.17E-130 | 80.45 |
| TRINITY_DN20689_c0_g1_i2 | phosphoglycerate mutase 4 | 1285 | 7.65E-130 | 77.85 |
| TRINITY_DN2068_c0_g1_i1 | signal peptide peptidase-like 4 isoform X1 | 1956 | 0 | 87.25 |
| TRINITY_DN2068_c0_g2_i1 | signal peptide peptidase-like 4 isoform X1 | 1956 | 0 | 87.25 |
| TRINITY_DN20702_c0_g2_i1 | probable fructokinase-4 | 1278 | 0 | 89.65 |
| TRINITY_DN20710_c0_g1_i1 | hypothetical protein DCAR_028460 | 1273 | 1.87E-11 | 41.67 |
| TRINITY_DN20710_c0_g1_i2 | hypothetical protein DCAR_028460 | 899 | 2.14E-07 | 41 |
| TRINITY_DN20710_c0_g1_i3 | hypothetical protein DCAR_028460 | 957 | 1.68E-07 | 41 |
| TRINITY_DN20711_c0_g1_i1 | receptor kinase At4g00960 | 2511 | 0 | 67.25 |
| TRINITY_DN20711_c0_g1_i4 | receptor kinase At4g00960 | 2462 | 0 | 64.9 |
| TRINITY_DN20713_c0_g2_i1 | carnosine N-methyltransferase | 1841 | 0 | 75.85 |
| TRINITY_DN20713_c0_g2_i9 | carnosine N-methyltransferase | 1442 | 2.85E-143 | 69.25 |
| TRINITY_DN20713_c1_g1_i1 | ---NA--- | 203 |  |  |
| TRINITY_DN20717_c0_g3_i1 | indole-3-acetaldehyde oxidase-like | 464 | 1.79E-52 | 85.15 |
| TRINITY_DN20719_c2_g1_i1 | syntaxin-61-like isoform X1 | 1110 | 3.01E-125 | 79.7 |
| TRINITY_DN20719_c2_g2_i1 | Syntaxin 61 family | 681 | 4.08E-26 | 77.1 |
| TRINITY_DN20726_c0_g1_i1 | ---NA--- | 1517 |  |  |
| TRINITY_DN20726_c0_g1_i3 | ---NA--- | 1568 |  |  |
| TRINITY_DN20726_c0_g1_i4 | ---NA--- | 1238 |  |  |
| TRINITY_DN20730_c0_g1_i1 | sn1-specific diacylglycerol lipase beta isoform X1 | 2402 | 0 | 70.5 |
| TRINITY_DN20730_c0_g1_i2 | sn1-specific diacylglycerol lipase beta isoform X1 | 2386 | 0 | 74.35 |
| TRINITY_DN20730_c0_g1_i3 | sn1-specific diacylglycerol lipase beta isoform X1 | 2346 | 0 | 69.7 |
| TRINITY_DN20730_c0_g1_i4 | class 3 | 2388 | 0 | 66.05 |
| TRINITY_DN20730_c0_g1_i5 | sn1-specific diacylglycerol lipase beta isoform X1 | 2330 | 0 | 74.5 |
| TRINITY_DN20730_c0_g1_i6 | sn1-specific diacylglycerol lipase beta isoform X1 | 2372 | 0 | 80.9 |
| TRINITY_DN20732_c0_g1_i1 | V-type proton ATPase subunit a3 | 2931 | 0 | 89.3 |
| TRINITY_DN20732_c0_g1_i2 | V-type proton ATPase subunit a3 | 3083 | 0 | 89.45 |
| TRINITY_DN20732_c0_g1_i3 | V-type proton ATPase subunit a3 | 2928 | 0 | 89.4 |
| TRINITY_DN20732_c0_g1_i4 | V-type proton ATPase subunit a3 | 3080 | 0 | 89.4 |
| TRINITY_DN20734_c0_g3_i1 | BES1 BZR1 homolog 4-like isoform X1 | 486 | 1.23E-29 | 95.2 |
| TRINITY_DN20736_c0_g1_i1 | zinc finger ZAT10-like | 1257 | 2.80E-61 | 55.15 |
| TRINITY_DN20743_c0_g1_i1 | cyclin-dependent kinase inhibitor SMR9 | 799 | 3.38E-28 | 62.15 |
| TRINITY_DN20743_c0_g1_i2 | cyclin-dependent kinase inhibitor SMR9 | 622 | 2.87E-28 | 62.3 |
| TRINITY_DN20748_c0_g1_i1 | malate dehydrogenase [NADP] chloroplastic | 4199 | 0 | 91.4 |
| TRINITY_DN20751_c0_g1_i1 | poly [ADP-ribose] polymerase 1 | 846 | 3.83E-108 | 69.65 |
| TRINITY_DN20753_c0_g1_i1 | E3 ubiquitin- ligase Topors isoform X1 | 902 | 1.05E-63 | 66.25 |
| TRINITY_DN20753_c0_g1_i2 | E3 ubiquitin- ligase Topors isoform X1 | 915 | 1.19E-63 | 66.25 |
| TRINITY_DN20758_c0_g1_i1 | transmembrane 9 superfamily member 1 | 2381 | 0 | 93.6 |
| TRINITY_DN20758_c0_g1_i2 | transmembrane 9 superfamily member 1 | 2381 | 0 | 93.05 |
| TRINITY_DN20765_c0_g1_i1 | pentatricopeptide repeat-containing mitochondrial | 1685 | 0 | 81.8 |
| TRINITY_DN20765_c0_g1_i2 | pentatricopeptide repeat-containing mitochondrial | 1957 | 0 | 81.75 |
| TRINITY_DN20766_c0_g1_i3 | PREDICTED: uncharacterized protein LOC104908317 | 1557 | 9.69E-124 | 85.45 |
| TRINITY_DN20766_c0_g1_i4 | PREDICTED: uncharacterized protein LOC104908317 | 555 | 4.59E-48 | 78.7 |
| TRINITY_DN20766_c0_g1_i6 | PREDICTED: uncharacterized protein LOC104908317 | 1477 | 1.34E-160 | 84.35 |
| TRINITY_DN20768_c1_g1_i1 | ---NA--- | 470 |  |  |
| TRINITY_DN20768_c1_g2_i1 | ---NA--- | 467 |  |  |
| TRINITY_DN20772_c0_g1_i1 | PREDICTED: uncharacterized protein LOC104891653 | 1933 | 0 | 88.65 |
| TRINITY_DN20772_c0_g1_i2 | PREDICTED: uncharacterized protein LOC104891653 | 1979 | 0 | 89.4 |
| TRINITY_DN20772_c0_g3_i1 | AT4g27020 F10M23_360 | 642 | 1.14E-78 | 87.05 |
| TRINITY_DN20778_c0_g1_i1 | diacylglycerol kinase 7-like | 2157 | 0 | 84.8 |
| TRINITY_DN20778_c0_g2_i1 | diacylglycerol kinase 7-like | 2355 | 0 | 84.6 |
| TRINITY_DN20778_c0_g3_i1 | diacylglycerol kinase 4-like | 578 | 3.37E-27 | 67 |
| TRINITY_DN20781_c0_g1_i1 | MACPF domain-containing NSL1-like | 1993 | 0 | 81.45 |
| TRINITY_DN20790_c0_g1_i1 | ---NA--- | 1017 |  |  |
| TRINITY_DN20790_c0_g1_i3 | ---NA--- | 945 |  |  |
| TRINITY_DN20793_c0_g1_i2 | DNA polymerase eta isoform X1 | 2263 | 0 | 70.6 |
| TRINITY_DN20793_c0_g1_i4 | DNA polymerase eta isoform X1 | 2311 | 0 | 71.05 |
| TRINITY_DN20797_c0_g1_i1 | 3-hydroxyisobutyryl- hydrolase mitochondrial | 1622 | 0 | 86.65 |
| TRINITY_DN20797_c0_g1_i2 | 3-hydroxyisobutyryl- hydrolase mitochondrial | 1689 | 0 | 86.65 |
| TRINITY_DN20800_c0_g1_i1 | probable serine threonine- kinase cdc7 | 3711 | 0 | 67.9 |
| TRINITY_DN20803_c1_g1_i1 | ---NA--- | 768 |  |  |
| TRINITY_DN20816_c0_g1_i1 | AP2-like ethylene-responsive transcription factor TOE3 isoform X2 | 1336 | 9.72E-97 | 66.5 |
| TRINITY_DN20816_c0_g1_i2 | ---NA--- | 559 |  |  |
| TRINITY_DN20816_c0_g1_i3 | APETAL2-like family | 1444 | 1.02E-90 | 59.05 |
| TRINITY_DN20816_c0_g1_i4 | floral homeotic APETALA 2 | 2082 | 1.34E-143 | 60.4 |
| TRINITY_DN20816_c0_g1_i5 | floral homeotic APETALA 2 | 1974 | 2.83E-150 | 63.3 |
| TRINITY_DN20820_c0_g2_i1 | glycosyltransferase family 92 RCOM_0530710 | 2295 | 0 | 65.85 |
| TRINITY_DN20820_c0_g2_i2 | glycosyltransferase family 92 RCOM_0530710 | 2564 | 0 | 64.45 |
| TRINITY_DN20820_c0_g2_i3 | glycosyltransferase family 92 RCOM_0530710 | 2588 | 0 | 64.45 |
| TRINITY_DN20820_c0_g2_i4 | glycosyltransferase family 92 RCOM_0530710 | 2271 | 0 | 65.85 |
| TRINITY_DN20822_c0_g1_i1 | arginyl-tRNA-- transferase 2-like | 2493 | 0 | 68.35 |
| TRINITY_DN20822_c0_g1_i2 | arginyl-tRNA-- transferase 2-like | 2387 | 0 | 68.25 |
| TRINITY_DN20824_c0_g2_i1 | Heparan-alpha-glucosaminide N-acetyltransferase | 722 | 1.97E-18 | 93.1 |
| TRINITY_DN20825_c0_g4_i1 | ---NA--- | 572 |  |  |
| TRINITY_DN20825_c0_g5_i1 | ---NA--- | 545 |  |  |
| TRINITY_DN20827_c0_g1_i2 | probable sugar phosphate phosphate translocator At3g11320 | 1666 | 0 | 96.7 |
| TRINITY_DN20827_c0_g2_i1 | probable sugar phosphate phosphate translocator At3g11320 | 1666 | 0 | 96.65 |
| TRINITY_DN20830_c1_g1_i1 | ATPase family AAA domain-containing 1-like | 1442 | 5.63E-140 | 80.1 |
| TRINITY_DN20830_c1_g1_i3 | ATPase family AAA domain-containing 1-like | 982 | 1.06E-48 | 70.1 |
| TRINITY_DN20831_c0_g1_i1 | ---NA--- | 619 |  |  |
| TRINITY_DN20831_c0_g1_i2 | hypothetical protein DCAR_024801 | 1636 | 1.28E-10 | 43 |
| TRINITY_DN20834_c0_g1_i1 | serine threonine- kinase CDL1-like | 1771 | 0 | 78.05 |
| TRINITY_DN20834_c0_g1_i2 | serine threonine- kinase CDL1-like | 1672 | 0 | 77.7 |
| TRINITY_DN20834_c0_g2_i1 | serine threonine- kinase CDL1 | 690 | 7.63E-93 | 73.65 |
| TRINITY_DN20838_c0_g1_i1 | U-box domain-containing 44-like | 2624 | 0 | 73.7 |
| TRINITY_DN20847_c0_g1_i1 | peroxisome biosynthesis PAS1-like | 2963 | 0 | 82.8 |
| TRINITY_DN20848_c1_g1_i1 | vacuolar-processing enzyme | 1528 | 0 | 73.95 |
| TRINITY_DN20848_c3_g2_i1 | calmodulin | 841 | 2.29E-102 | 99.9 |
| TRINITY_DN20848_c3_g3_i1 | calmodulin | 841 | 2.29E-102 | 99.9 |
| TRINITY_DN20848_c3_g4_i1 | calmodulin | 841 | 2.29E-102 | 99.9 |
| TRINITY_DN2084_c0_g1_i1 | ---NA--- | 246 |  |  |
| TRINITY_DN2084_c0_g2_i1 | ---NA--- | 257 |  |  |
| TRINITY_DN20851_c0_g1_i1 | Phox domain-containing isoform 1 | 3520 | 0 | 74.75 |
| TRINITY_DN20851_c0_g1_i2 | Phox domain-containing isoform 1 | 3507 | 0 | 63.3 |
| TRINITY_DN20853_c0_g10_i1 | ---NA--- | 363 |  |  |
| TRINITY_DN20853_c0_g1_i1 | ---NA--- | 322 |  |  |
| TRINITY_DN20853_c0_g2_i1 | ---NA--- | 351 |  |  |
| TRINITY_DN20853_c0_g3_i1 | ---NA--- | 357 |  |  |
| TRINITY_DN20853_c0_g4_i1 | ---NA--- | 355 |  |  |
| TRINITY_DN20853_c0_g5_i1 | ---NA--- | 361 |  |  |
| TRINITY_DN20853_c0_g6_i1 | ---NA--- | 525 |  |  |
| TRINITY_DN20853_c0_g6_i2 | AC099400_1 poly | 1564 | 2.40E-11 | 44.1 |
| TRINITY_DN20853_c0_g7_i1 | ---NA--- | 365 |  |  |
| TRINITY_DN20853_c0_g8_i1 | ---NA--- | 359 |  |  |
| TRINITY_DN20853_c0_g9_i1 | ---NA--- | 353 |  |  |
| TRINITY_DN20857_c0_g1_i1 | probable xyloglucan galactosyltransferase GT11 | 461 | 2.02E-21 | 81.05 |
| TRINITY_DN20857_c0_g2_i1 | probable xyloglucan galactosyltransferase GT11 | 468 | 5.32E-25 | 81.05 |
| TRINITY_DN20857_c1_g1_i1 | probable xyloglucan galactosyltransferase GT11 | 1164 | 7.39E-143 | 69.35 |
| TRINITY_DN20857_c1_g1_i2 | probable xyloglucan galactosyltransferase GT11 | 1236 | 1.68E-139 | 68.55 |
| TRINITY_DN20857_c2_g1_i1 | PREDICTED: uncharacterized protein LOC104901855 | 1202 | 2.22E-52 | 52.6 |
| TRINITY_DN20858_c0_g1_i1 | transcription elongation factor SPT5 homolog 1 | 910 | 1.52E-73 | 80.4 |
| TRINITY_DN20858_c0_g1_i2 | transcription elongation factor SPT5 homolog 1 | 3536 | 0 | 86.55 |
| TRINITY_DN20860_c1_g1_i1 | ---NA--- | 288 |  |  |
| TRINITY_DN20860_c1_g2_i1 | ---NA--- | 294 |  |  |
| TRINITY_DN20863_c0_g1_i1 | yrdC domain-containing mitochondrial isoform X3 | 1638 | 3.92E-120 | 82.25 |
| TRINITY_DN20863_c0_g1_i2 | yrdC domain-containing mitochondrial isoform X3 | 1491 | 2.61E-116 | 82.25 |
| TRINITY_DN20863_c0_g1_i3 | yrdC domain-containing mitochondrial isoform X1 | 1373 | 6.29E-113 | 81.75 |
| TRINITY_DN20863_c0_g1_i4 | yrdC domain-containing mitochondrial isoform X3 | 1398 | 9.44E-117 | 82.45 |
| TRINITY_DN20863_c0_g1_i5 | yrdC domain-containing mitochondrial isoform X1 | 1226 | 1.20E-113 | 81.75 |
| TRINITY_DN20863_c0_g1_i6 | yrdC domain-containing mitochondrial isoform X3 | 1392 | 8.83E-117 | 82.25 |
| TRINITY_DN20863_c0_g1_i7 | yrdC domain-containing mitochondrial isoform X3 | 1251 | 1.79E-117 | 82.45 |
| TRINITY_DN20863_c0_g1_i8 | yrdC domain-containing mitochondrial isoform X3 | 1539 | 4.38E-116 | 82.25 |
| TRINITY_DN20863_c1_g1_i1 | ---NA--- | 258 |  |  |
| TRINITY_DN20871_c1_g1_i1 | CAAX amino terminal protease | 585 | 1.61E-23 | 89 |
| TRINITY_DN20871_c1_g2_i1 | CAAX amino terminal protease | 5530 | 0 | 63.8 |
| TRINITY_DN20872_c0_g1_i1 | argonaute 16 | 3063 | 0 | 74.35 |
| TRINITY_DN20872_c0_g1_i2 | argonaute 16 | 2790 | 0 | 74.35 |
| TRINITY_DN20874_c0_g1_i1 | phospholipid:diacylglycerol acyltransferase 1 | 2572 | 0 | 89.55 |
| TRINITY_DN20874_c0_g1_i2 | phospholipid:diacylglycerol acyltransferase 1 | 2685 | 0 | 87.25 |
| TRINITY_DN20877_c0_g1_i3 | ---NA--- | 302 |  |  |
| TRINITY_DN20877_c0_g1_i4 | ---NA--- | 355 |  |  |
| TRINITY_DN20888_c1_g2_i1 | ---NA--- | 1116 |  |  |
| TRINITY_DN20889_c0_g1_i1 | ---NA--- | 372 |  |  |
| TRINITY_DN2088_c0_g1_i1 | PREDICTED: uncharacterized protein LOC18600838 | 458 | 4.92E-32 | 92.4 |
| TRINITY_DN2088_c0_g1_i2 | PREDICTED: uncharacterized protein LOC104896729 | 575 | 4.05E-58 | 86.15 |
| TRINITY_DN20890_c0_g3_i1 | disease resistance RGA3 | 946 | 4.05E-27 | 53 |
| TRINITY_DN20891_c1_g1_i1 | ---NA--- | 319 |  |  |
| TRINITY_DN20898_c0_g1_i1 | PREDICTED: uncharacterized protein LOC104896290 | 1253 | 6.83E-122 | 73.55 |
| TRINITY_DN20907_c0_g1_i1 | tyrosine--tRNA ligase cytoplasmic | 2570 | 0 | 77.45 |
| TRINITY_DN20908_c0_g1_i1 | serine threonine- kinase EDR1 isoform X1 | 2782 | 0 | 82.9 |
| TRINITY_DN20908_c0_g1_i2 | serine threonine- kinase EDR1 isoform X1 | 2449 | 0 | 83.25 |
| TRINITY_DN20908_c0_g1_i3 | serine threonine- kinase EDR1 isoform X1 | 2870 | 0 | 84.65 |
| TRINITY_DN20908_c0_g1_i4 | serine threonine- kinase EDR1 isoform X1 | 2955 | 0 | 84.65 |
| TRINITY_DN20908_c0_g1_i5 | serine threonine- kinase EDR1 isoform X1 | 2867 | 0 | 82.9 |
| TRINITY_DN20915_c0_g2_i1 | ---NA--- | 487 |  |  |
| TRINITY_DN20915_c0_g6_i1 | LNK1 isoform X1 | 2589 | 5.92E-112 | 50.2 |
| TRINITY_DN20917_c0_g1_i1 | trihelix transcription factor GT-2-like | 1232 | 1.67E-101 | 69.45 |
| TRINITY_DN20917_c0_g1_i2 | trihelix transcription factor GT-2-like | 1265 | 2.05E-102 | 65.05 |
| TRINITY_DN20917_c0_g2_i1 | trihelix transcription factor GT-2 | 1829 | 1.96E-121 | 75.65 |
| TRINITY_DN20920_c0_g2_i1 | molybdopterin synthase catalytic subunit | 836 | 4.97E-98 | 80.5 |
| TRINITY_DN20923_c0_g1_i1 | receptor kinase At1g72540 | 1609 | 0 | 79.15 |
| TRINITY_DN20925_c1_g1_i1 | zinc finger family | 1234 | 9.69E-105 | 84.55 |
| TRINITY_DN20936_c1_g1_i1 | ABC transporter B family member 11 | 4016 | 0 | 87.55 |
| TRINITY_DN20942_c0_g1_i1 | probable pectin methyltransferase QUA2 isoform X1 | 3205 | 0 | 80.05 |
| TRINITY_DN20942_c0_g1_i2 | probable pectin methyltransferase QUA2 isoform X2 | 1766 | 0 | 86.15 |
| TRINITY_DN20942_c0_g1_i3 | probable pectin methyltransferase QUA2 isoform X1 | 2107 | 0 | 86.35 |
| TRINITY_DN20942_c0_g1_i4 | probable pectin methyltransferase QUA2 isoform X1 | 2864 | 0 | 80.65 |
| TRINITY_DN20942_c0_g1_i5 | probable pectin methyltransferase QUA2 isoform X1 | 2109 | 0 | 80.1 |
| TRINITY_DN20942_c0_g1_i6 | probable pectin methyltransferase QUA2 isoform X2 | 2944 | 0 | 84.95 |
| TRINITY_DN20946_c0_g1_i1 | U3 small nucleolar ribonucleo MPP10 | 1554 | 3.01E-128 | 84.6 |
| TRINITY_DN20946_c0_g1_i2 | U3 small nucleolar ribonucleo MPP10 | 1542 | 9.64E-129 | 84.3 |
| TRINITY_DN20952_c0_g1_i2 | phosphate transporter PHO1 | 1195 | 4.58E-17 | 82.5 |
| TRINITY_DN20952_c0_g1_i6 | phosphate transporter PHO1 | 978 | 3.40E-17 | 82.5 |
| TRINITY_DN20953_c0_g2_i1 | Methyl-CPG-binding domain isoform 2 | 3014 | 0 | 53.25 |
| TRINITY_DN20957_c0_g2_i3 | ---NA--- | 918 |  |  |
| TRINITY_DN20958_c0_g1_i3 | AP-1 complex subunit sigma-1 | 1021 | 5.90E-109 | 95.4 |
| TRINITY_DN20958_c0_g1_i4 | AP-1 complex subunit sigma-1 | 1021 | 6.87E-109 | 95.45 |
| TRINITY_DN20958_c1_g1_i1 | ---NA--- | 220 |  |  |
| TRINITY_DN20963_c0_g1_i1 | translation initiation factor IF-2 | 1226 | 1.74E-82 | 56.95 |
| TRINITY_DN20965_c0_g1_i1 | myb-related Myb4-like | 1408 | 7.49E-103 | 91.15 |
| TRINITY_DN20965_c0_g2_i1 | myb-related Myb4-like | 1484 | 1.65E-102 | 91.25 |
| TRINITY_DN20965_c2_g2_i1 | myb family transcription factor family | 514 | 4.00E-18 | 56.4 |
| TRINITY_DN20965_c2_g2_i2 | myb-related 306 | 1521 | 1.91E-112 | 69.75 |
| TRINITY_DN20967_c0_g1_i1 | thioredoxin domain-containing 9 homolog | 1245 | 3.88E-109 | 91.8 |
| TRINITY_DN20967_c0_g1_i2 | thioredoxin domain-containing 9 homolog | 1264 | 2.55E-109 | 91.7 |
| TRINITY_DN20968_c1_g1_i1 | ---NA--- | 417 |  |  |
| TRINITY_DN20968_c1_g2_i1 | RING U-box isoform 2 | 2198 | 0 | 61.75 |
| TRINITY_DN20971_c0_g2_i3 | fumarate hydratase mitochondrial | 1978 | 0 | 95.1 |
| TRINITY_DN20973_c0_g1_i1 | BURP domain-containing 5 | 1197 | 2.41E-103 | 54.15 |
| TRINITY_DN20982_c0_g1_i1 | hypothetical protein SOVF_100040 | 1030 | 5.53E-07 | 57.63 |
| TRINITY_DN20982_c0_g1_i2 | hypothetical protein SOVF_138000 | 1037 | 4.97E-07 | 54.5 |
| TRINITY_DN20992_c0_g1_i1 | zinc finger CCCH domain-containing 66 isoform X1 | 485 | 6.52E-13 | 63.55 |
| TRINITY_DN20992_c1_g1_i1 | zinc finger CCCH domain-containing 66-like isoform X1 | 1992 | 0 | 68.85 |
| TRINITY_DN20992_c1_g2_i1 | zinc finger CCCH domain-containing 66 | 2271 | 0 | 69.8 |
| TRINITY_DN20996_c0_g1_i1 | heat shock 18 | 715 | 2.66E-67 | 87.95 |
| TRINITY_DN20996_c0_g1_i3 | heat shock 18 | 793 | 1.57E-67 | 90.45 |
| TRINITY_DN20997_c1_g1_i1 | ---NA--- | 955 |  |  |
| TRINITY_DN20997_c1_g4_i1 | ---NA--- | 817 |  |  |
| TRINITY_DN21003_c0_g1_i1 | DETOXIFICATION chloroplastic | 2224 | 0 | 75.55 |
| TRINITY_DN21003_c0_g1_i2 | DETOXIFICATION chloroplastic | 2224 | 0 | 75.35 |
| TRINITY_DN21003_c0_g2_i1 | DETOXIFICATION chloroplastic | 1100 | 3.85E-133 | 75.25 |
| TRINITY_DN21012_c0_g1_i1 | fructose-2,6- | 1760 | 0 | 95.7 |
| TRINITY_DN21012_c0_g1_i2 | fructose-2,6- | 1261 | 0 | 96.7 |
| TRINITY_DN21013_c0_g1_i1 | hypothetical protein SOVF_137360 | 2319 | 0 | 82.35 |
| TRINITY_DN21022_c0_g1_i1 | Phosphomethylpyrimidine synthase | 1135 | 2.29E-95 | 91.55 |
| TRINITY_DN21022_c0_g1_i2 | Phosphomethylpyrimidine synthase | 1175 | 4.88E-97 | 90.75 |
| TRINITY_DN21022_c0_g1_i3 | PREDICTED: uncharacterized protein LOC104893180 | 1264 | 4.95E-83 | 90.8 |
| TRINITY_DN21023_c0_g1_i1 | calmodulin-binding transcription activator 2-like | 3462 | 0 | 70.55 |
| TRINITY_DN21023_c0_g1_i2 | calmodulin-binding transcription activator 2-like isoform X1 | 3525 | 0 | 66.35 |
| TRINITY_DN21036_c0_g1_i1 | nascent polypeptide-associated complex subunit muscle-specific form | 2776 | 3.35E-60 | 61.65 |
| TRINITY_DN21036_c0_g1_i2 | nascent polypeptide-associated complex subunit muscle-specific form | 2942 | 4.90E-60 | 61.65 |
| TRINITY_DN21036_c0_g1_i3 | nascent polypeptide-associated complex subunit muscle-specific form | 2681 | 6.72E-61 | 61.5 |
| TRINITY_DN21036_c0_g1_i4 | nascent polypeptide-associated complex subunit muscle-specific form | 2848 | 3.99E-60 | 61.65 |
| TRINITY_DN21036_c0_g1_i5 | nascent polypeptide-associated complex subunit muscle-specific form | 2775 | 8.71E-61 | 61.5 |
| TRINITY_DN21047_c0_g1_i1 | ABC transporter mitochondrial | 1657 | 0 | 87.65 |
| TRINITY_DN21047_c0_g1_i2 | ABC transporter mitochondrial | 1164 | 0 | 85.95 |
| TRINITY_DN21047_c0_g2_i1 | aarF domain-containing kinase 4 | 370 | 2.12E-29 | 75.45 |
| TRINITY_DN21050_c0_g1_i1 | CBL-interacting serine threonine- kinase 8 isoform X1 | 1586 | 0 | 90.6 |
| TRINITY_DN21051_c0_g1_i1 | ---NA--- | 273 |  |  |
| TRINITY_DN21051_c0_g2_i1 | ---NA--- | 274 |  |  |
| TRINITY_DN21051_c0_g4_i1 | STRUBBELIG-RECEPTOR FAMILY 3 isoform X2 | 1786 | 0 | 85.65 |
| TRINITY_DN21051_c0_g4_i2 | STRUBBELIG-RECEPTOR FAMILY 3 isoform X2 | 1662 | 0 | 86.45 |
| TRINITY_DN21051_c0_g5_i1 | ---NA--- | 405 |  |  |
| TRINITY_DN21054_c0_g1_i6 | F-box kelch-repeat At3g06240 | 630 | 8.86E-12 | 74.5 |
| TRINITY_DN21057_c0_g1_i1 | WAT1-related At5g40240 | 1008 | 8.40E-106 | 74.15 |
| TRINITY_DN21057_c0_g1_i2 | WAT1-related At5g40240-like | 1529 | 0 | 75.4 |
| TRINITY_DN21057_c0_g1_i3 | WAT1-related At5g40240 | 1563 | 4.84E-103 | 74.15 |
| TRINITY_DN21057_c0_g1_i4 | WAT1-related At5g40240-like | 1200 | 0 | 75.4 |
| TRINITY_DN21057_c0_g1_i5 | WAT1-related At5g40240 | 1247 | 1.31E-104 | 74.15 |
| TRINITY_DN21057_c0_g1_i6 | WAT1-related At5g40240 | 1337 | 4.36E-104 | 74.15 |
| TRINITY_DN21057_c0_g1_i7 | WAT1-related At5g40240-like | 1755 | 0 | 75.4 |
| TRINITY_DN21057_c0_g1_i8 | WAT1-related At5g40240-like | 1439 | 0 | 75.4 |
| TRINITY_DN21059_c0_g2_i2 | nitrogen regulatory P-II homolog | 1054 | 2.32E-80 | 87.45 |
| TRINITY_DN21071_c0_g3_i1 | heterogeneous nuclear ribonucleo 1 | 2525 | 0 | 71.5 |
| TRINITY_DN21071_c0_g3_i2 | heterogeneous nuclear ribonucleo 1 | 2349 | 0 | 73.05 |
| TRINITY_DN21071_c0_g3_i3 | heterogeneous nuclear ribonucleo 1 | 2521 | 0 | 72.25 |
| TRINITY_DN21072_c0_g1_i1 | trimethylguanosine synthase isoform X3 | 2252 | 6.01E-117 | 84.85 |
| TRINITY_DN21072_c0_g1_i2 | trimethylguanosine synthase isoform X3 | 2270 | 8.45E-113 | 84.9 |
| TRINITY_DN21075_c0_g1_i1 | probable plastidic glucose transporter 3 isoform X1 | 2024 | 0 | 81.95 |
| TRINITY_DN21075_c0_g1_i10 | probable plastidic glucose transporter 3 isoform X1 | 1875 | 0 | 81.95 |
| TRINITY_DN21075_c0_g1_i13 | probable plastidic glucose transporter 3 isoform X1 | 1823 | 0 | 84.75 |
| TRINITY_DN21075_c0_g1_i14 | probable plastidic glucose transporter 3 isoform X1 | 1972 | 0 | 84.75 |
| TRINITY_DN21075_c0_g1_i2 | probable plastidic glucose transporter 3 isoform X1 | 1937 | 0 | 83.4 |
| TRINITY_DN21075_c0_g1_i3 | probable plastidic glucose transporter 3 isoform X1 | 1788 | 0 | 83.4 |
| TRINITY_DN21075_c0_g1_i4 | probable plastidic glucose transporter 3 isoform X1 | 1840 | 0 | 79.65 |
| TRINITY_DN21075_c0_g1_i9 | probable plastidic glucose transporter 3 isoform X1 | 1989 | 0 | 79.65 |
| TRINITY_DN21087_c0_g1_i1 | SAC3 family A isoform X1 | 3482 | 0 | 73.9 |
| TRINITY_DN21089_c0_g1_i1 | transport SEC23-like | 2922 | 0 | 85.05 |
| TRINITY_DN21090_c0_g2_i1 | NADPH--cytochrome P450 reductase | 2789 | 0 | 85.4 |
| TRINITY_DN21090_c0_g2_i2 | NADPH--cytochrome P450 reductase | 2566 | 0 | 85.55 |
| TRINITY_DN21092_c0_g1_i1 | threonine--tRNA chloroplastic mitochondrial 2 | 2428 | 0 | 89.8 |
| TRINITY_DN21097_c0_g1_i1 | calcium-dependent kinase 34 | 2024 | 0 | 88.9 |
| TRINITY_DN21097_c0_g2_i1 | calcium-dependent kinase 17 | 2024 | 0 | 89 |
| TRINITY_DN21104_c0_g2_i1 | ---NA--- | 497 |  |  |
| TRINITY_DN21104_c0_g2_i3 | glucan endo-1,3-beta-glucosidase 8-like | 970 | 1.87E-53 | 76.9 |
| TRINITY_DN21107_c0_g1_i1 | Putrescine-binding periplasmic | 2584 | 0 | 73.15 |
| TRINITY_DN21107_c0_g1_i2 | Putrescine-binding periplasmic | 2570 | 0 | 80 |
| TRINITY_DN21108_c0_g1_i1 | GDSL esterase lipase At5g55050 | 783 | 1.80E-31 | 85.15 |
| TRINITY_DN21108_c0_g2_i1 | DNA mismatch repair MSH5 | 4050 | 0 | 87.7 |
| TRINITY_DN21108_c0_g2_i2 | DNA mismatch repair MSH5 | 4218 | 0 | 88.35 |
| TRINITY_DN21108_c0_g2_i3 | DNA mismatch repair MSH5 | 4216 | 0 | 87.05 |
| TRINITY_DN21108_c0_g2_i4 | DNA mismatch repair MSH5 | 4236 | 0 | 87.45 |
| TRINITY_DN21108_c0_g2_i5 | DNA mismatch repair MSH5 | 4088 | 0 | 87.05 |
| TRINITY_DN21108_c0_g2_i6 | DNA mismatch repair MSH5 | 4068 | 0 | 87 |
| TRINITY_DN21108_c0_g2_i7 | DNA mismatch repair MSH5 | 4070 | 0 | 88 |
| TRINITY_DN21108_c0_g2_i8 | DNA mismatch repair MSH5 | 4198 | 0 | 87.85 |
| TRINITY_DN21110_c5_g2_i3 | ---NA--- | 1426 |  |  |
| TRINITY_DN21110_c6_g1_i1 | ATP synthase subunit mitochondrial | 1054 | 5.65E-95 | 94.95 |
| TRINITY_DN21110_c6_g2_i1 | ATP synthase subunit mitochondrial | 1042 | 9.55E-91 | 95.5 |
| TRINITY_DN21110_c6_g3_i1 | ATP synthase subunit mitochondrial | 1048 | 5.28E-95 | 94.95 |
| TRINITY_DN21111_c0_g2_i1 | mitogen-activated kinase kinase kinase 1-like | 2877 | 0 | 69.1 |
| TRINITY_DN21111_c0_g2_i2 | mitogen-activated kinase kinase kinase 1 | 2353 | 0 | 66.5 |
| TRINITY_DN21111_c0_g2_i3 | mitogen-activated kinase kinase kinase 1 | 2599 | 0 | 67.25 |
| TRINITY_DN21111_c0_g2_i4 | mitogen-activated kinase kinase kinase 1-like | 2591 | 0 | 69.1 |
| TRINITY_DN21111_c1_g1_i1 | mitogen-activated kinase kinase kinase 1 | 1438 | 1.08E-66 | 53.95 |
| TRINITY_DN21116_c0_g1_i1 | ---NA--- | 240 |  |  |
| TRINITY_DN21119_c0_g1_i1 | soyasapogenol B glucuronide galactosyltransferase-like | 1737 | 2.53E-168 | 63.65 |
| TRINITY_DN21126_c0_g1_i1 | beta-glucosidase 3B-like | 2306 | 0 | 85.15 |
| TRINITY_DN21126_c0_g1_i2 | beta-glucosidase 3B-like | 2157 | 0 | 84.85 |
| TRINITY_DN21134_c0_g1_i1 | root UVB sensitive 6 | 2239 | 0 | 83.2 |
| TRINITY_DN21138_c0_g4_i1 | copper-transporting ATPase chloroplastic-like isoform X2 | 1196 | 3.25E-52 | 84.65 |
| TRINITY_DN21139_c1_g2_i1 | synaptotagmin-5 | 1758 | 0 | 86.8 |
| TRINITY_DN21142_c0_g1_i1 | lon protease homolog peroxisomal | 3215 | 0 | 90.85 |
| TRINITY_DN21151_c1_g1_i1 | chloride channel CLC-b | 4502 | 0 | 88.3 |
| TRINITY_DN21151_c1_g1_i4 | chloride channel CLC-b | 3824 | 0 | 88.3 |
| TRINITY_DN21151_c1_g1_i5 | chloride channel CLC-b | 4208 | 0 | 90.05 |
| TRINITY_DN21161_c0_g1_i1 | PREDICTED: uncharacterized protein LOC104896742 | 2145 | 9.37E-23 | 57.45 |
| TRINITY_DN21161_c0_g1_i2 | PREDICTED: uncharacterized protein LOC104896742 | 2253 | 9.89E-23 | 57.45 |
| TRINITY_DN21162_c0_g1_i1 | zinc finger CCCH domain-containing 19 | 5550 | 0 | 66.15 |
| TRINITY_DN21163_c0_g1_i2 | galacturonosyltransferase 8 | 1950 | 0 | 89.5 |
| TRINITY_DN21165_c0_g2_i1 | lipase-like | 1894 | 0 | 89.05 |
| TRINITY_DN21165_c1_g1_i1 | PREDICTED: uncharacterized protein LOC104897268 | 304 | 8.03E-08 | 62.63 |
| TRINITY_DN21167_c0_g1_i1 | hypothetical protein SOVF_062580 isoform B | 1380 | 8.06E-56 | 73.75 |
| TRINITY_DN21167_c0_g1_i2 | hypothetical protein SOVF_062580 isoform B | 1353 | 8.46E-52 | 71.9 |
| TRINITY_DN21170_c0_g1_i1 | altered inheritance rate of mitochondria 25-like | 1432 | 9.33E-118 | 87.7 |
| TRINITY_DN21170_c0_g1_i2 | altered inheritance rate of mitochondria 25-like | 1185 | 6.69E-29 | 84.15 |
| TRINITY_DN21170_c0_g1_i3 | altered inheritance rate of mitochondria 25 | 1367 | 5.26E-154 | 76.65 |
| TRINITY_DN21170_c0_g1_i4 | altered inheritance rate of mitochondria 25 | 1266 | 1.05E-28 | 84.05 |
| TRINITY_DN21173_c0_g2_i3 | HBS1 isoform X1 | 2745 | 0 | 73.15 |
| TRINITY_DN21173_c0_g2_i4 | elongation factor 1-alpha isoform X1 | 2726 | 0 | 87.45 |
| TRINITY_DN21174_c0_g1_i1 | UDP-glucose 4-epimerase GEPI48 | 1965 | 0 | 92.3 |
| TRINITY_DN21175_c0_g1_i1 | CHROMATIN REMODELING 25 | 901 | 1.27E-16 | 77.85 |
| TRINITY_DN21175_c0_g1_i2 | DNA repair and recombination RAD54 | 1018 | 4.25E-16 | 79.75 |
| TRINITY_DN21175_c0_g1_i3 | CHROMATIN REMODELING 25 | 1054 | 1.50E-15 | 78.95 |
| TRINITY_DN21175_c0_g1_i4 | DNA repair and recombination RAD54 | 941 | 1.42E-16 | 69.55 |
| TRINITY_DN21175_c0_g1_i5 | DNA repair and recombination RAD54 | 1156 | 1.63E-20 | 77.3 |
| TRINITY_DN21178_c1_g4_i2 | ---NA--- | 235 |  |  |
| TRINITY_DN21178_c1_g4_i3 | ---NA--- | 394 |  |  |
| TRINITY_DN21178_c1_g4_i4 | ---NA--- | 382 |  |  |
| TRINITY_DN21192_c0_g1_i1 | RNA-binding 39 | 2007 | 0 | 78.1 |
| TRINITY_DN21205_c0_g1_i1 | transcription factor bHLH137 | 825 | 2.48E-49 | 59.55 |
| TRINITY_DN21205_c0_g2_i1 | transcription factor bHLH137 | 1316 | 2.50E-74 | 63.65 |
| TRINITY_DN21205_c0_g2_i2 | transcription factor bHLH137 | 1484 | 1.63E-87 | 58.7 |
| TRINITY_DN21208_c0_g1_i1 | plant mmn10-180 | 1435 | 0 | 85.25 |
| TRINITY_DN21208_c0_g1_i2 | V-type ATP synthase beta chain 1 | 499 | 8.35E-27 | 88.7 |
| TRINITY_DN21208_c0_g1_i3 | V-type ATP synthase beta chain 1 | 1464 | 1.09E-136 | 88.2 |
| TRINITY_DN2120_c0_g1_i1 | very-long-chain (3R)-3-hydroxyacyl- dehydratase PASTICCINO 2A | 1159 | 1.72E-131 | 88.35 |
| TRINITY_DN2120_c0_g2_i1 | very-long-chain (3R)-3-hydroxyacyl- dehydratase PASTICCINO 2A | 1158 | 5.20E-136 | 88.2 |
| TRINITY_DN21216_c0_g1_i1 | oxalate-- ligase-like | 2091 | 0 | 87.85 |
| TRINITY_DN21221_c1_g1_i1 | ---NA--- | 234 |  |  |
| TRINITY_DN21227_c0_g1_i1 | WD repeat-containing 26-like | 2246 | 0 | 79.42 |
| TRINITY_DN21235_c0_g1_i1 | ---NA--- | 305 |  |  |
| TRINITY_DN21235_c1_g1_i3 | far upstream element-binding 2 isoform X1 | 1916 | 1.91E-114 | 68.94 |
| TRINITY_DN21237_c0_g1_i1 | ISWI chromatin-remodeling complex ATPase CHR11 | 3637 | 0 | 95.3 |
| TRINITY_DN21237_c0_g1_i2 | ISWI chromatin-remodeling complex ATPase CHR11 | 3367 | 0 | 95.32 |
| TRINITY_DN21237_c0_g1_i3 | ISWI chromatin-remodeling complex ATPase CHR11 | 3637 | 0 | 95.58 |
| TRINITY_DN21240_c0_g1_i1 | transcription factor bHLH18-like | 975 | 1.32E-33 | 67.15 |
| TRINITY_DN21240_c0_g1_i2 | transcription factor bHLH18-like | 1065 | 2.64E-33 | 67.15 |
| TRINITY_DN21240_c1_g1_i1 | formin 20 | 1391 | 1.33E-100 | 57.1 |
| TRINITY_DN21240_c1_g1_i2 | formin 20 | 2495 | 0 | 74.4 |
| TRINITY_DN21243_c0_g1_i1 | WALLS ARE THIN 1 | 1948 | 0 | 89.05 |
| TRINITY_DN2124_c0_g1_i1 | alcohol dehydrogenase | 1487 | 0 | 94.1 |
| TRINITY_DN21251_c0_g1_i2 | Myb family transcription factor At1g14600 | 1357 | 9.17E-57 | 77.32 |
| TRINITY_DN21251_c0_g1_i3 | ---NA--- | 923 |  |  |
| TRINITY_DN21252_c3_g2_i3 | ethylene-responsive transcription factor 1B-like | 981 | 1.34E-55 | 70.05 |
| TRINITY_DN21255_c0_g1_i1 | UPF0481 At3g47200-like | 1933 | 2.60E-84 | 60.89 |
| TRINITY_DN21255_c0_g1_i2 | UPF0481 At3g47200-like | 1986 | 5.09E-103 | 53.35 |
| TRINITY_DN21255_c0_g3_i1 | UPF0481 At3g47200-like | 1112 | 1.35E-50 | 55.95 |
| TRINITY_DN21256_c0_g2_i3 | Cyclopropane-fatty-acyl-phospholipid synthase | 634 | 1.56E-22 | 87.7 |
| TRINITY_DN21256_c0_g2_i4 | Cyclopropane-fatty-acyl-phospholipid synthase | 594 | 6.55E-36 | 64.55 |
| TRINITY_DN21256_c0_g2_i5 | Cyclopropane-fatty-acyl-phospholipid synthase | 612 | 1.23E-22 | 87.7 |
| TRINITY_DN21256_c0_g2_i8 | Cyclopropane-fatty-acyl-phospholipid synthase | 840 | 9.44E-22 | 88.2 |
| TRINITY_DN21258_c0_g1_i2 | ---NA--- | 1518 |  |  |
| TRINITY_DN21265_c0_g1_i1 | 187-kDa microtubule-associated AIR9 | 5251 | 0 | 83.68 |
| TRINITY_DN21268_c0_g1_i1 | cytochrome B5 | 747 | 3.85E-49 | 81.15 |
| TRINITY_DN21268_c0_g1_i2 | cytochrome B5 | 774 | 2.14E-51 | 81.45 |
| TRINITY_DN21268_c0_g1_i3 | cytochrome B5 | 552 | 2.92E-27 | 95.5 |
| TRINITY_DN21268_c0_g1_i4 | cytochrome B5 | 448 | 1.00E-48 | 86.9 |
| TRINITY_DN21273_c0_g1_i1 | endoribonuclease Dicer homolog 1 | 5744 | 0 | 90.05 |
| TRINITY_DN21274_c0_g1_i1 | ---NA--- | 245 |  |  |
| TRINITY_DN21274_c0_g5_i2 | ---NA--- | 434 |  |  |
| TRINITY_DN21277_c0_g2_i1 | autophagy-related 11 | 3837 | 0 | 76.83 |
| TRINITY_DN21278_c0_g2_i1 | rho-N domain-containing chloroplastic | 1060 | 7.88E-83 | 60.45 |
| TRINITY_DN21278_c0_g2_i2 | rho-N domain-containing chloroplastic | 1067 | 7.73E-72 | 60.45 |
| TRINITY_DN21278_c1_g1_i1 | jagunal homolog 1 | 830 | 3.15E-44 | 69.85 |
| TRINITY_DN21280_c0_g1_i1 | GCN5-related N-acetyltransferase family | 2650 | 7.46E-105 | 76.9 |
| TRINITY_DN21280_c0_g1_i10 | serine threonine- kinase PBS1 | 2949 | 0 | 89 |
| TRINITY_DN21280_c0_g1_i11 | serine threonine- kinase PBS1 | 2694 | 0 | 88.8 |
| TRINITY_DN21280_c0_g1_i13 | serine threonine- kinase PBS1 | 1961 | 2.68E-107 | 80.35 |
| TRINITY_DN21280_c0_g1_i14 | serine threonine- kinase PBS1 | 2082 | 0 | 89 |
| TRINITY_DN21280_c0_g1_i15 | serine threonine- kinase PBS1 | 1827 | 0 | 88.79 |
| TRINITY_DN21280_c0_g1_i16 | serine threonine- kinase PBS1 | 2516 | 1.90E-159 | 89.95 |
| TRINITY_DN21280_c0_g1_i3 | GCN5-related N-acetyltransferase family | 2395 | 1.10E-105 | 76.9 |
| TRINITY_DN21280_c0_g1_i5 | GCN5-related N-acetyltransferase family | 2216 | 2.53E-106 | 76.9 |
| TRINITY_DN21280_c0_g1_i6 | serine threonine- kinase PBS1 | 3383 | 3.83E-156 | 90.06 |
| TRINITY_DN21280_c0_g1_i8 | serine threonine- kinase PBS1 | 2261 | 1.49E-160 | 89.95 |
| TRINITY_DN21280_c0_g1_i9 | serine threonine- kinase PBS1 | 3128 | 4.84E-157 | 89.95 |
| TRINITY_DN21286_c0_g1_i1 | transcription factor TCP2 | 1247 | 8.52E-46 | 83.85 |
| TRINITY_DN21287_c0_g1_i1 | myb D isoform X2 | 1743 | 2.20E-92 | 63.15 |
| TRINITY_DN21287_c0_g1_i2 | myb D | 712 | 2.92E-71 | 78.55 |
| TRINITY_DN21289_c0_g1_i1 | caffeoylshikimate esterase-like | 1504 | 0 | 84.74 |
| TRINITY_DN21291_c0_g1_i1 | probable WRKY transcription factor 48 | 1519 | 2.44E-51 | 60.42 |
| TRINITY_DN21294_c0_g1_i1 | ---NA--- | 296 |  |  |
| TRINITY_DN21296_c1_g3_i1 | elongation factor 1-alpha | 1958 | 0 | 99 |
| TRINITY_DN21296_c1_g3_i3 | elongation factor 1-alpha | 1984 | 0 | 99 |
| TRINITY_DN21296_c1_g3_i4 | elongation factor 1-alpha | 1419 | 0 | 99 |
| TRINITY_DN21296_c1_g4_i1 | Elongation factor 1- | 212 | 5.55E-21 | 98.85 |
| TRINITY_DN21306_c0_g1_i1 | mitochondrial glyco | 1712 | 1.68E-28 | 69.2 |
| TRINITY_DN21306_c0_g1_i2 | mitochondrial glyco | 1642 | 1.39E-28 | 69.2 |
| TRINITY_DN21306_c0_g1_i3 | mitochondrial glyco | 1727 | 1.29E-36 | 70.5 |
| TRINITY_DN21311_c0_g1_i2 | Casein kinase II subunit alpha-2 | 1756 | 0 | 96.75 |
| TRINITY_DN21311_c0_g1_i3 | Casein kinase II subunit alpha-2 | 1635 | 0 | 97.55 |
| TRINITY_DN21314_c0_g1_i1 | 3-oxo-Delta(4,5)-steroid 5-beta-reductase | 3097 | 0 | 82.65 |
| TRINITY_DN21314_c1_g1_i1 | kelch domain-containing 4 | 1766 | 0 | 84.2 |
| TRINITY_DN21314_c1_g2_i1 | kelch domain-containing 4 | 1847 | 0 | 84.25 |
| TRINITY_DN21314_c2_g1_i1 | DNA polymerase V | 2836 | 0 | 77.4 |
| TRINITY_DN21314_c3_g1_i1 | NADPH-dependent pterin aldehyde reductase | 1191 | 9.39E-125 | 86.5 |
| TRINITY_DN21314_c5_g1_i1 | kelch domain-containing 4 | 679 | 4.30E-64 | 89.45 |
| TRINITY_DN21315_c0_g1_i1 | ultraviolet-B receptor UVR8 | 1834 | 4.91E-134 | 88.6 |
| TRINITY_DN21319_c0_g1_i1 | ---NA--- | 380 |  |  |
| TRINITY_DN21319_c0_g1_i2 | ---NA--- | 705 |  |  |
| TRINITY_DN21327_c0_g1_i1 | ABC transporter B family member chloroplastic isoform X2 | 2478 | 0 | 90.35 |
| TRINITY_DN21327_c0_g1_i2 | ABC transporter B family member chloroplastic | 2595 | 0 | 84.7 |
| TRINITY_DN21327_c0_g1_i3 | ABC transporter B family member chloroplastic | 2467 | 0 | 86.85 |
| TRINITY_DN21327_c0_g1_i4 | ABC transporter B family member chloroplastic | 2584 | 0 | 82.2 |
| TRINITY_DN21328_c0_g1_i2 | heat shock 70 kDa mitochondrial | 2501 | 0 | 91.1 |
| TRINITY_DN21328_c0_g1_i4 | heat shock 70 kDa mitochondrial | 2587 | 0 | 91.45 |
| TRINITY_DN21330_c0_g1_i2 | histone H4 | 437 | 1.20E-52 | 99.7 |
| TRINITY_DN21330_c0_g1_i3 | histone H4 | 613 | 2.54E-56 | 99.7 |
| TRINITY_DN21331_c1_g2_i1 | E3 ubiquitin- ligase MARCH8 | 872 | 8.64E-81 | 77.55 |
| TRINITY_DN21331_c1_g2_i2 | E3 ubiquitin- ligase MARCH8 | 884 | 9.94E-81 | 77.55 |
| TRINITY_DN21334_c1_g1_i1 | magnesium-chelatase subunit chloroplastic | 4501 | 0 | 94 |
| TRINITY_DN21337_c0_g1_i1 | myosin heavy chain kinase C | 1688 | 0 | 74.4 |
| TRINITY_DN21339_c0_g1_i1 | ---NA--- | 277 |  |  |
| TRINITY_DN21339_c0_g2_i1 | ---NA--- | 229 |  |  |
| TRINITY_DN21339_c0_g3_i1 | lysM domain-containing GPI-anchored 2-like isoform X2 | 244 | 1.59E-11 | 76 |
| TRINITY_DN21339_c1_g1_i1 | pre-mRNA-processing factor 39 isoform X1 | 3342 | 0 | 71.9 |
| TRINITY_DN21339_c1_g1_i2 | pre-mRNA-processing factor 39 isoform X1 | 3434 | 1.81E-180 | 72.7 |
| TRINITY_DN21339_c1_g1_i3 | pre-mRNA-processing factor 39 isoform X1 | 3518 | 5.24E-177 | 72.7 |
| TRINITY_DN21339_c1_g1_i4 | Tetratricopeptide repeat (TPR)-like superfamily | 3258 | 0 | 69.6 |
| TRINITY_DN21339_c1_g1_i5 | pre-mRNA-processing factor 39 isoform X1 | 3033 | 0 | 74.25 |
| TRINITY_DN21339_c1_g1_i6 | pre-mRNA-processing factor 39 isoform X3 | 3357 | 0 | 72.2 |
| TRINITY_DN21344_c0_g1_i2 | plastid division chloroplastic | 2800 | 0 | 74.35 |
| TRINITY_DN21360_c0_g1_i1 | PREDICTED: uncharacterized protein LOC104896312 isoform X1 | 1223 | 3.83E-170 | 66.05 |
| TRINITY_DN21364_c0_g3_i1 | VQ motif-containing | 748 | 5.58E-29 | 50.9 |
| TRINITY_DN21364_c0_g3_i4 | GTP-binding At2g22870 | 1631 | 2.54E-135 | 90.55 |
| TRINITY_DN21364_c0_g3_i5 | GTP-binding At2g22870 | 1442 | 3.05E-136 | 90.55 |
| TRINITY_DN21365_c0_g1_i1 | zinc finger BRUTUS | 4408 | 0 | 82.35 |
| TRINITY_DN21365_c0_g2_i1 | zinc finger BRUTUS | 1983 | 0 | 79.05 |
| TRINITY_DN2136_c0_g1_i1 | GTP-binding nuclear Ran-3 | 1242 | 2.88E-145 | 96.5 |
| TRINITY_DN21373_c0_g1_i1 | ---NA--- | 239 |  |  |
| TRINITY_DN21373_c0_g2_i1 | PREDICTED: protein SRG1 | 2583 | 1.82E-06 | 85 |
| TRINITY_DN21373_c0_g2_i2 | PREDICTED: protein SRG1 | 2085 | 1.36E-06 | 85 |
| TRINITY_DN21375_c0_g1_i1 | ---NA--- | 1092 |  |  |
| TRINITY_DN21375_c0_g1_i2 | ---NA--- | 1166 |  |  |
| TRINITY_DN21384_c0_g1_i1 | ---NA--- | 632 |  |  |
| TRINITY_DN21385_c0_g1_i1 | tubby-like F-box 3 | 2093 | 1.99E-152 | 82.55 |
| TRINITY_DN21385_c0_g1_i2 | tubby-like F-box 3 | 2223 | 0 | 85.35 |
| TRINITY_DN21385_c0_g1_i3 | tubby-like F-box 3 | 2224 | 0 | 85.35 |
| TRINITY_DN21391_c0_g1_i1 | tudor domain-containing 3 | 463 | 7.83E-64 | 74.8 |
| TRINITY_DN21391_c0_g1_i3 | tudor domain-containing 3 | 1334 | 5.27E-156 | 69.15 |
| TRINITY_DN21392_c1_g1_i3 | lysophospholipid acyltransferase LPEAT2 | 1879 | 0 | 81.8 |
| TRINITY_DN21392_c1_g1_i4 | lysophospholipid acyltransferase LPEAT2 | 520 | 5.13E-38 | 66.75 |
| TRINITY_DN21392_c1_g1_i6 | lysophospholipid acyltransferase LPEAT2 | 1892 | 0 | 77.75 |
| TRINITY_DN21393_c0_g1_i1 | DDB1- and CUL4-associated factor homolog 1 | 4183 | 0 | 80.75 |
| TRINITY_DN21393_c0_g1_i2 | DDB1- and CUL4-associated factor homolog 1 | 4210 | 0 | 80.9 |
| TRINITY_DN21394_c0_g1_i2 | cyclic nucleotide-gated ion channel 1-like | 1260 | 2.88E-113 | 76.4 |
| TRINITY_DN21394_c0_g1_i3 | cyclic nucleotide-gated ion channel 1 | 2646 | 0 | 73.45 |
| TRINITY_DN21394_c0_g1_i4 | cyclic nucleotide-gated ion channel 1 | 2569 | 0 | 73.45 |
| TRINITY_DN21394_c0_g1_i5 | cyclic nucleotide-gated ion channel 1-like | 1273 | 4.17E-148 | 79.45 |
| TRINITY_DN21394_c0_g1_i6 | cyclic nucleotide-gated ion channel 1-like | 2582 | 0 | 73.7 |
| TRINITY_DN21394_c0_g1_i7 | cyclic nucleotide-gated ion channel 1-like | 2659 | 0 | 73.7 |
| TRINITY_DN21395_c0_g1_i1 | nuclear RNA polymerase A1 | 5218 | 0 | 67.8 |
| TRINITY_DN21395_c0_g1_i2 | DNA-directed RNA polymerase I subunit 1 | 5183 | 0 | 71.35 |
| TRINITY_DN21401_c0_g1_i1 | jasmonate-induced homolog | 642 | 1.40E-16 | 47.11 |
| TRINITY_DN21401_c0_g1_i2 | jasmonate-induced homolog | 630 | 1.26E-13 | 46.4 |
| TRINITY_DN21403_c0_g1_i1 | transcription factor MYB1R1 | 1425 | 1.79E-168 | 76.8 |
| TRINITY_DN21409_c0_g1_i1 | nuclear poly(A) polymerase 4-like | 2762 | 0 | 84 |
| TRINITY_DN21409_c0_g1_i2 | nuclear poly(A) polymerase 4-like | 2860 | 0 | 84.2 |
| TRINITY_DN21409_c0_g1_i3 | nuclear poly(A) polymerase 4-like | 2665 | 0 | 88.45 |
| TRINITY_DN21409_c1_g1_i1 | ---NA--- | 380 |  |  |
| TRINITY_DN2140_c0_g1_i1 | pentatricopeptide repeat-containing At1g71210 | 5111 | 0 | 70.95 |
| TRINITY_DN21411_c0_g2_i2 | E3 ubiquitin- ligase UPL5 | 2254 | 0 | 71 |
| TRINITY_DN21411_c0_g3_i1 | WD repeat-containing 44 | 452 | 4.03E-12 | 63.88 |
| TRINITY_DN21416_c0_g1_i1 | ---NA--- | 258 |  |  |
| TRINITY_DN21416_c0_g2_i1 | ---NA--- | 586 |  |  |
| TRINITY_DN21416_c0_g2_i2 | ---NA--- | 914 |  |  |
| TRINITY_DN21416_c0_g2_i5 | ---NA--- | 452 |  |  |
| TRINITY_DN21416_c0_g2_i8 | ---NA--- | 1048 |  |  |
| TRINITY_DN21418_c0_g1_i1 | disulfide-isomerase 5-3-like | 1958 | 0 | 86.25 |
| TRINITY_DN21418_c0_g1_i2 | disulfide-isomerase 5-3-like | 1905 | 0 | 86.85 |
| TRINITY_DN21420_c0_g1_i1 | poly [ADP-ribose] polymerase 2-like isoform X2 | 663 | 5.25E-19 | 64.8 |
| TRINITY_DN21420_c0_g1_i2 | poly [ADP-ribose] polymerase 2 | 1071 | 9.68E-111 | 68.75 |
| TRINITY_DN21420_c0_g1_i3 | poly [ADP-ribose] polymerase 2 | 2307 | 0 | 66.5 |
| TRINITY_DN21421_c1_g1_i1 | ARM REPEAT PROTEIN INTERACTING WITH ABF2 | 2832 | 0 | 87.8 |
| TRINITY_DN21428_c0_g1_i1 | PREDICTED: uncharacterized protein LOC104904259 | 1186 | 6.26E-46 | 51 |
| TRINITY_DN21428_c1_g1_i1 | ---NA--- | 219 |  |  |
| TRINITY_DN21432_c1_g1_i1 | U11 U12 small nuclear ribonucleo 25 kDa -like isoform X2 | 967 | 2.16E-64 | 71.8 |
| TRINITY_DN21432_c1_g2_i1 | U11 U12 small nuclear ribonucleo 25 kDa isoform X2 | 1050 | 2.98E-92 | 64.55 |
| TRINITY_DN21432_c2_g1_i1 | Dev_Cell_Death domain-containing | 2451 | 8.41E-79 | 66.5 |
| TRINITY_DN21432_c2_g1_i2 | Dev_Cell_Death domain-containing | 2533 | 2.10E-67 | 79.2 |
| TRINITY_DN21434_c0_g1_i1 | thaumatin 1b isoform X2 | 1155 | 7.67E-147 | 80.9 |
| TRINITY_DN21434_c0_g1_i2 | thaumatin 1b isoform X2 | 1344 | 7.58E-146 | 80.9 |
| TRINITY_DN21439_c0_g1_i1 | ABC transporter B family member 28 | 1137 | 1.45E-48 | 93.75 |
| TRINITY_DN21439_c0_g1_i6 | ABC transporter B family member 28 | 1101 | 1.02E-51 | 93.75 |
| TRINITY_DN21439_c0_g1_i7 | ABC transporter B family member 28 | 2582 | 0 | 86.65 |
| TRINITY_DN21442_c0_g1_i1 | pentatricopeptide repeat-containing mitochondrial | 661 | 3.98E-41 | 64.95 |
| TRINITY_DN21442_c0_g1_i2 | pentatricopeptide repeat-containing mitochondrial | 711 | 1.60E-40 | 61.45 |
| TRINITY_DN21442_c0_g1_i3 | pentatricopeptide repeat-containing mitochondrial | 682 | 2.01E-41 | 63.85 |
| TRINITY_DN21443_c0_g1_i1 | chloroplastic | 863 | 5.61E-32 | 57.3 |
| TRINITY_DN21444_c0_g2_i2 | ---NA--- | 1438 |  |  |
| TRINITY_DN21449_c0_g3_i1 | Nudix hydrolase 9 | 1388 | 1.27E-163 | 81.65 |
| TRINITY_DN21449_c0_g3_i2 | Nudix hydrolase 9 | 1242 | 2.09E-164 | 81.65 |
| TRINITY_DN21449_c0_g3_i3 | Nudix hydrolase 9 | 1223 | 5.80E-169 | 81.65 |
| TRINITY_DN21449_c0_g3_i4 | Nudix hydrolase 9 | 1148 | 6.53E-165 | 81.65 |
| TRINITY_DN21449_c0_g3_i5 | Nudix hydrolase 9 | 1387 | 1.27E-163 | 81.65 |
| TRINITY_DN21449_c1_g1_i2 | ---NA--- | 715 |  |  |
| TRINITY_DN21450_c0_g1_i5 | hypothetical protein BVRB_5g098800 | 458 | 9.43E-18 | 68.71 |
| TRINITY_DN21453_c0_g1_i1 | homeobox -like isoform X1 | 3174 | 4.18E-179 | 63.35 |
| TRINITY_DN21460_c0_g1_i1 | methyl- -binding domain-containing 13-like | 805 | 1.91E-20 | 50.15 |
| TRINITY_DN21460_c0_g1_i2 | methyl- -binding domain-containing 13-like | 2327 | 5.66E-37 | 48.8 |
| TRINITY_DN21460_c0_g1_i3 | Methyl- -binding domain-containing 13 | 2405 | 1.17E-30 | 46.95 |
| TRINITY_DN21462_c0_g1_i1 | ---NA--- | 320 |  |  |
| TRINITY_DN21462_c0_g2_i8 | ---NA--- | 727 |  |  |
| TRINITY_DN21466_c0_g1_i1 | C2 domain-containing At1g53590 isoform X1 | 5176 | 0 | 77.7 |
| TRINITY_DN21466_c0_g1_i4 | C2 domain-containing At1g53590 isoform X1 | 5697 | 0 | 77.45 |
| TRINITY_DN21476_c0_g1_i1 | ---NA--- | 419 |  |  |
| TRINITY_DN21476_c0_g1_i2 | ---NA--- | 325 |  |  |
| TRINITY_DN21476_c0_g1_i3 | ---NA--- | 394 |  |  |
| TRINITY_DN21493_c0_g2_i1 | probable serine threonine- kinase At1g01540 | 561 | 2.95E-50 | 61.3 |
| TRINITY_DN21493_c0_g2_i2 | probable serine threonine- kinase At1g01540 | 1557 | 0 | 77.35 |
| TRINITY_DN21495_c0_g1_i1 | probable aminotransferase ACS10 | 1965 | 0 | 76.4 |
| TRINITY_DN21495_c0_g1_i2 | probable aminotransferase ACS10 | 929 | 4.62E-32 | 68.8 |
| TRINITY_DN21496_c0_g1_i1 | ---NA--- | 712 |  |  |
| TRINITY_DN21497_c0_g1_i4 | probable plastid-lipid-associated chloroplastic | 1070 | 1.69E-76 | 90.95 |
| TRINITY_DN21497_c0_g1_i9 | probable plastid-lipid-associated chloroplastic | 1047 | 1.35E-76 | 90.8 |
| TRINITY_DN2149_c0_g1_i1 | 50S ribosomal chloroplastic | 869 | 4.92E-55 | 94.05 |
| TRINITY_DN21503_c0_g1_i1 | transport sec23 | 3124 | 0 | 82.45 |
| TRINITY_DN21503_c0_g1_i2 | transport sec23 | 3103 | 0 | 82.45 |
| TRINITY_DN21514_c0_g2_i1 | DNA polymerase delta catalytic subunit | 3729 | 0 | 92.1 |
| TRINITY_DN21522_c0_g1_i1 | ---NA--- | 752 |  |  |
| TRINITY_DN21524_c0_g2_i1 | DENN domain-containing isoform 1 | 3306 | 0 | 76.9 |
| TRINITY_DN21524_c0_g2_i2 | ---NA--- | 835 |  |  |
| TRINITY_DN21528_c0_g1_i1 | zinc finger CCCH domain-containing 13-like | 2772 | 0 | 74.7 |
| TRINITY_DN21532_c1_g1_i1 | alpha-galactosidase 1-like | 1888 | 0 | 88.6 |
| TRINITY_DN21533_c0_g1_i1 | ---NA--- | 496 |  |  |
| TRINITY_DN21533_c0_g2_i1 | U-box domain-containing 44 | 3191 | 0 | 81.75 |
| TRINITY_DN21533_c0_g2_i3 | U-box domain-containing 44 | 3136 | 0 | 82.55 |
| TRINITY_DN2153_c0_g1_i1 | abscisic stress-ripening 2-like | 799 | 6.70E-23 | 90.15 |
| TRINITY_DN21543_c0_g1_i1 | nifU mitochondrial | 1169 | 2.73E-138 | 90.5 |
| TRINITY_DN21548_c0_g1_i2 | ---NA--- | 221 |  |  |
| TRINITY_DN21549_c0_g1_i1 | indeterminate-domain 7-like isoform X2 | 631 | 3.89E-15 | 56.22 |
| TRINITY_DN21549_c0_g2_i1 | DNA-3-methyladenine glycosylase 1 | 1077 | 2.64E-105 | 86.75 |
| TRINITY_DN21549_c1_g2_i1 | nucleosome assembly 1 2-like | 2033 | 6.79E-137 | 91.65 |
| TRINITY_DN21549_c1_g2_i2 | nucleosome assembly 1 2-like | 2054 | 7.78E-137 | 90.5 |
| TRINITY_DN21550_c0_g1_i1 | ---NA--- | 987 |  |  |
| TRINITY_DN21550_c0_g1_i2 | ---NA--- | 353 |  |  |
| TRINITY_DN21554_c0_g1_i1 | NEDD1 isoform X1 | 2840 | 0 | 76.8 |
| TRINITY_DN21554_c0_g2_i1 | F-box LRR-repeat 15 | 3641 | 0 | 77.95 |
| TRINITY_DN21554_c0_g2_i2 | F-box LRR-repeat 15 | 797 | 1.98E-06 | 50 |
| TRINITY_DN21554_c0_g2_i3 | F-box LRR-repeat 15 | 2817 | 0 | 78 |
| TRINITY_DN21555_c0_g1_i1 | D-lactate dehydrogenase [cytochrome] mitochondrial | 2263 | 0 | 86.9 |
| TRINITY_DN21555_c0_g1_i2 | D-lactate dehydrogenase [cytochrome] mitochondrial | 2264 | 0 | 89.25 |
| TRINITY_DN21557_c5_g7_i1 | ---NA--- | 223 |  |  |
| TRINITY_DN21557_c5_g7_i2 | ---NA--- | 272 |  |  |
| TRINITY_DN21559_c0_g1_i1 | serine threonine- kinase AFC2-like isoform X1 | 248 | 1.35E-11 | 87.21 |
| TRINITY_DN21559_c0_g1_i2 | serine threonine- kinase AFC2 isoform X1 | 1762 | 0 | 91.4 |
| TRINITY_DN21559_c0_g1_i3 | serine threonine- kinase AFC2 isoform X1 | 1563 | 0 | 95.6 |
| TRINITY_DN21559_c0_g1_i4 | serine threonine- kinase AFC2 isoform X2 | 2189 | 7.61E-138 | 93.65 |
| TRINITY_DN21559_c0_g1_i5 | serine threonine- kinase AFC2 isoform X1 | 640 | 1.17E-91 | 88 |
| TRINITY_DN21564_c1_g1_i1 | Ras domain-containing Adaptin_binding domain-containing | 1883 | 7.95E-152 | 73.8 |
| TRINITY_DN21564_c1_g1_i2 | PREDICTED: uncharacterized protein LOC104888193 | 861 | 1.21E-12 | 84.5 |
| TRINITY_DN21564_c1_g1_i3 | ---NA--- | 615 |  |  |
| TRINITY_DN2156_c0_g1_i1 | exocyst complex component SEC5A-like | 3251 | 0 | 84.35 |
| TRINITY_DN21570_c0_g1_i1 | DEK isoform X2 | 1790 | 3.16E-96 | 78.8 |
| TRINITY_DN21570_c0_g2_i1 | histone -specific chaperone CHZ1-like | 977 | 2.00E-10 | 93 |
| TRINITY_DN21570_c1_g1_i1 | ---NA--- | 408 |  |  |
| TRINITY_DN21571_c1_g1_i1 | ---NA--- | 557 |  |  |
| TRINITY_DN21575_c1_g1_i3 | ---NA--- | 1006 |  |  |
| TRINITY_DN21575_c1_g1_i5 | ---NA--- | 887 |  |  |
| TRINITY_DN21575_c1_g2_i1 | ---NA--- | 463 |  |  |
| TRINITY_DN21575_c1_g2_i2 | ---NA--- | 521 |  |  |
| TRINITY_DN21576_c1_g1_i1 | pericentriolar material 1 | 2179 | 4.15E-138 | 53.05 |
| TRINITY_DN21576_c1_g1_i2 | pericentriolar material 1 | 2200 | 2.90E-146 | 53.85 |
| TRINITY_DN21576_c1_g2_i1 | pericentriolar material 1 | 2422 | 5.94E-127 | 54 |
| TRINITY_DN21583_c0_g1_i4 | DUF3128 domain-containing | 753 | 9.13E-54 | 80.8 |
| TRINITY_DN21583_c0_g1_i6 | DUF3128 domain-containing | 826 | 6.44E-52 | 78.85 |
| TRINITY_DN21583_c0_g1_i7 | DUF3128 domain-containing | 457 | 1.45E-30 | 76.95 |
| TRINITY_DN21583_c0_g1_i8 | DUF3128 domain-containing | 582 | 4.20E-53 | 78.85 |
| TRINITY_DN21586_c0_g1_i1 | thylakoid membrane chloroplastic isoform X1 | 1188 | 1.90E-92 | 72.4 |
| TRINITY_DN21586_c0_g1_i10 | thylakoid membrane chloroplastic isoform X1 | 1573 | 0 | 85.05 |
| TRINITY_DN21586_c0_g1_i11 | thylakoid membrane chloroplastic isoform X1 | 1535 | 1.01E-129 | 72.1 |
| TRINITY_DN21586_c0_g1_i12 | thylakoid membrane chloroplastic isoform X1 | 1516 | 8.66E-168 | 79.9 |
| TRINITY_DN21586_c0_g1_i14 | thylakoid membrane chloroplastic isoform X1 | 992 | 1.63E-135 | 78.1 |
| TRINITY_DN21586_c0_g1_i15 | thylakoid membrane chloroplastic isoform X1 | 1169 | 1.03E-120 | 75.05 |
| TRINITY_DN21586_c0_g1_i3 | thylakoid membrane chloroplastic isoform X1 | 970 | 1.69E-118 | 82 |
| TRINITY_DN21586_c0_g1_i4 | thylakoid membrane chloroplastic isoform X1 | 1245 | 3.71E-105 | 80.4 |
| TRINITY_DN21586_c0_g1_i5 | thylakoid membrane chloroplastic isoform X1 | 913 | 3.93E-102 | 74.7 |
| TRINITY_DN21586_c0_g1_i6 | thylakoid membrane chloroplastic isoform X1 | 1226 | 5.60E-137 | 81.15 |
| TRINITY_DN21586_c0_g1_i7 | thylakoid membrane chloroplastic isoform X1 | 1592 | 1.86E-142 | 80.5 |
| TRINITY_DN21586_c0_g1_i8 | thylakoid membrane chloroplastic isoform X1 | 1494 | 1.83E-126 | 81.9 |
| TRINITY_DN21586_c0_g1_i9 | thylakoid membrane chloroplastic isoform X1 | 1437 | 9.26E-114 | 74.55 |
| TRINITY_DN21590_c0_g5_i2 | ty1-copia retrotransposon | 509 | 2.02E-13 | 79.05 |
| TRINITY_DN21595_c0_g2_i1 | nucleolin 1 isoform X1 | 2467 | 1.22E-79 | 75.95 |
| TRINITY_DN21595_c0_g2_i3 | nucleolin 1 isoform X1 | 2482 | 2.80E-68 | 72.85 |
| TRINITY_DN21598_c0_g1_i1 | pentatricopeptide repeat-containing At5g27270 | 4306 | 0 | 76.3 |
| TRINITY_DN21598_c0_g1_i2 | pentatricopeptide repeat-containing At5g27270 | 4549 | 0 | 76.3 |
| TRINITY_DN21598_c1_g1_i1 | transcription initiation factor IIA large subunit-like | 1372 | 5.37E-166 | 73.4 |
| TRINITY_DN21604_c0_g1_i1 | mediator-associated 1-like | 1716 | 6.87E-27 | 63.95 |
| TRINITY_DN21607_c0_g2_i1 | ---NA--- | 395 |  |  |
| TRINITY_DN21609_c0_g1_i2 | cryptochrome-1 isoform X1 | 2709 | 0 | 85.4 |
| TRINITY_DN21614_c0_g1_i1 | ---NA--- | 278 |  |  |
| TRINITY_DN21614_c0_g3_i1 | potassium transporter 6-like | 2838 | 0 | 88.65 |
| TRINITY_DN21619_c1_g1_i1 | ---NA--- | 353 |  |  |
| TRINITY_DN21619_c1_g1_i2 | ---NA--- | 390 |  |  |
| TRINITY_DN21623_c0_g1_i1 | allene oxide synthase | 1909 | 0 | 84.9 |
| TRINITY_DN21625_c0_g1_i1 | MATH domain-containing At5g43560 isoform X1 | 3397 | 0 | 70.65 |
| TRINITY_DN21625_c0_g1_i2 | MATH domain-containing At5g43560 isoform X1 | 3370 | 0 | 70.05 |
| TRINITY_DN21625_c0_g1_i3 | MATH domain-containing At5g43560-like | 3746 | 0 | 71.65 |
| TRINITY_DN21625_c0_g1_i4 | MATH domain-containing At5g43560 isoform X1 | 3719 | 0 | 71.8 |
| TRINITY_DN21625_c0_g1_i5 | MATH domain-containing At5g43560 isoform X1 | 3674 | 0 | 71.7 |
| TRINITY_DN21625_c0_g1_i6 | MATH domain-containing At5g43560 | 2438 | 0 | 60.85 |
| TRINITY_DN21625_c0_g1_i7 | MATH domain-containing At5g43560 isoform X1 | 3701 | 0 | 71.85 |
| TRINITY_DN21625_c0_g1_i8 | MATH domain-containing At5g43560-like | 1817 | 1.29E-165 | 58.15 |
| TRINITY_DN21634_c0_g1_i1 | ---NA--- | 257 |  |  |
| TRINITY_DN21634_c0_g2_i4 | ---NA--- | 350 |  |  |
| TRINITY_DN21635_c0_g1_i1 | probable transmembrane GTPase FZO- chloroplastic | 2978 | 0 | 75.85 |
| TRINITY_DN21635_c0_g1_i10 | probable transmembrane GTPase FZO- chloroplastic | 3068 | 0 | 80.05 |
| TRINITY_DN21635_c0_g1_i2 | probable transmembrane GTPase FZO- chloroplastic | 2834 | 0 | 75 |
| TRINITY_DN21635_c0_g1_i3 | probable transmembrane GTPase FZO- chloroplastic | 2884 | 0 | 79.6 |
| TRINITY_DN21635_c0_g1_i4 | probable transmembrane GTPase FZO- chloroplastic | 2744 | 0 | 72.15 |
| TRINITY_DN21635_c0_g1_i7 | probable transmembrane GTPase FZO- chloroplastic | 2928 | 0 | 72.55 |
| TRINITY_DN21635_c0_g1_i8 | probable transmembrane GTPase FZO- chloroplastic | 2794 | 0 | 75.85 |
| TRINITY_DN21635_c0_g1_i9 | probable transmembrane GTPase FZO- chloroplastic | 3018 | 0 | 75.3 |
| TRINITY_DN21635_c0_g2_i1 | probable transmembrane GTPase FZO- chloroplastic | 341 | 1.24E-10 | 71.1 |
| TRINITY_DN21637_c0_g1_i1 | cytochrome P450 CYP72A219-like | 528 | 1.98E-54 | 68.5 |
| TRINITY_DN21637_c0_g2_i2 | cytochrome P450 CYP72A219-like | 1385 | 1.37E-170 | 64.2 |
| TRINITY_DN21637_c0_g2_i3 | cytochrome P450 CYP72A219-like | 1694 | 0 | 71.45 |
| TRINITY_DN21637_c0_g2_i5 | cytochrome P450 CYP72A219-like | 1697 | 0 | 71.35 |
| TRINITY_DN21639_c0_g1_i1 | root phototropism 3 | 2585 | 0 | 87.3 |
| TRINITY_DN21639_c0_g1_i2 | root phototropism 3 | 2566 | 0 | 87.3 |
| TRINITY_DN21639_c1_g1_i1 | root phototropism 3 | 889 | 1.18E-161 | 85.8 |
| TRINITY_DN21639_c1_g3_i1 | vacuolar sorting-associated chloroplastic | 3893 | 0 | 79.9 |
| TRINITY_DN21639_c1_g3_i2 | vacuolar sorting-associated chloroplastic isoform X1 | 3879 | 0 | 79.55 |
| TRINITY_DN21639_c1_g3_i3 | vacuolar sorting-associated chloroplastic isoform X1 | 3501 | 0 | 79.55 |
| TRINITY_DN21639_c1_g3_i4 | vacuolar sorting-associated chloroplastic | 3515 | 0 | 79.9 |
| TRINITY_DN21639_c1_g3_i5 | vacuolar sorting-associated chloroplastic | 3464 | 0 | 79.9 |
| TRINITY_DN21639_c1_g3_i6 | vacuolar sorting-associated chloroplastic isoform X1 | 3450 | 0 | 79.55 |
| TRINITY_DN21642_c0_g2_i1 | ---NA--- | 460 |  |  |
| TRINITY_DN21644_c0_g2_i2 | metal-nicotianamine transporter YSL1 | 2385 | 0 | 86.75 |
| TRINITY_DN21644_c0_g3_i1 | DUF632 domain-containing DUF630 domain-containing | 1305 | 1.00E-18 | 83.45 |
| TRINITY_DN21645_c0_g2_i1 | EMSY-LIKE 3 | 1633 | 1.89E-174 | 72.65 |
| TRINITY_DN21645_c0_g2_i3 | EMSY-LIKE 3-like isoform X1 | 2799 | 1.02E-179 | 75.05 |
| TRINITY_DN21645_c0_g2_i4 | probable polygalacturonase At1g80170 | 736 | 2.08E-32 | 78.5 |
| TRINITY_DN21645_c0_g2_i5 | EMSY-LIKE 3 | 1600 | 1.42E-166 | 71.25 |
| TRINITY_DN21651_c0_g1_i2 | ABIL1 | 1459 | 8.93E-167 | 79.4 |
| TRINITY_DN21651_c0_g1_i3 | ABIL1 | 1606 | 5.09E-166 | 79.4 |
| TRINITY_DN21652_c0_g2_i1 | histone-lysine N-methyltransferase trithorax | 865 | 6.47E-20 | 57.65 |
| TRINITY_DN2165_c0_g1_i1 | DUF506 domain-containing | 1095 | 7.94E-113 | 78.3 |
| TRINITY_DN2165_c1_g1_i1 | ---NA--- | 410 |  |  |
| TRINITY_DN21662_c0_g1_i1 | transcription factor PIF3 isoform X1 | 2751 | 1.45E-163 | 55.7 |
| TRINITY_DN21662_c0_g1_i2 | transcription factor PIF3 isoform X1 | 2628 | 2.72E-158 | 54.8 |
| TRINITY_DN21668_c1_g5_i1 | SPX domain-containing 1 | 1032 | 5.22E-141 | 73.65 |
| TRINITY_DN21671_c0_g1_i1 | ethylene-overproduction 1 | 775 | 7.16E-64 | 73.9 |
| TRINITY_DN21671_c0_g2_i1 | BEL1-like homeodomain 4 | 1476 | 4.54E-150 | 84.85 |
| TRINITY_DN21671_c0_g2_i2 | BEL1-like homeodomain 4 | 543 | 1.85E-15 | 81.55 |
| TRINITY_DN21671_c0_g2_i3 | BEL1-like homeodomain 4 | 1839 | 0 | 77.1 |
| TRINITY_DN21672_c1_g1_i1 | ---NA--- | 326 |  |  |
| TRINITY_DN21672_c2_g1_i1 | ---NA--- | 322 |  |  |
| TRINITY_DN21672_c2_g2_i1 | ---NA--- | 323 |  |  |
| TRINITY_DN21673_c0_g1_i1 | methionine S-methyltransferase | 3853 | 0 | 83.85 |
| TRINITY_DN21677_c0_g1_i1 | probable WRKY transcription factor 57 | 1290 | 7.36E-71 | 69.1 |
| TRINITY_DN21677_c0_g1_i2 | probable WRKY transcription factor 57 | 1308 | 8.65E-71 | 69.1 |
| TRINITY_DN21682_c0_g1_i1 | PRA1 family H | 1270 | 8.98E-87 | 69.45 |
| TRINITY_DN21682_c0_g1_i4 | PRA1 family H | 1239 | 6.35E-87 | 69.45 |
| TRINITY_DN21685_c0_g1_i1 | ---NA--- | 205 |  |  |
| TRINITY_DN21685_c1_g2_i1 | ---NA--- | 1039 |  |  |
| TRINITY_DN21685_c1_g2_i2 | ---NA--- | 727 |  |  |
| TRINITY_DN21685_c1_g2_i3 | ---NA--- | 931 |  |  |
| TRINITY_DN21692_c0_g1_i1 | flowering time control FY isoform X1 | 2560 | 0 | 85.2 |
| TRINITY_DN21699_c0_g1_i1 | formin 14 | 2227 | 0 | 88.85 |
| TRINITY_DN21703_c0_g1_i5 | ---NA--- | 823 |  |  |
| TRINITY_DN21703_c0_g1_i8 | ---NA--- | 787 |  |  |
| TRINITY_DN21708_c0_g2_i1 | DNA polymerase delta catalytic subunit | 1291 | 5.26E-61 | 54.85 |
| TRINITY_DN21708_c1_g1_i3 | plant calmodulin-binding | 6357 | 0 | 48.55 |
| TRINITY_DN2170_c0_g1_i1 | ---NA--- | 920 |  |  |
| TRINITY_DN21712_c0_g1_i1 | nuclear pore complex NUP96 | 855 | 2.22E-91 | 63.25 |
| TRINITY_DN21712_c0_g2_i1 | nuclear pore complex NUP96 | 3179 | 0 | 76.25 |
| TRINITY_DN21716_c0_g1_i1 | squamosa promoter-binding 1 | 3112 | 0 | 68.85 |
| TRINITY_DN21723_c0_g1_i1 | GDSL esterase lipase 1-like | 643 | 4.29E-86 | 70.7 |
| TRINITY_DN21723_c0_g2_i1 | GDSL esterase lipase 1-like | 757 | 1.73E-80 | 69.6 |
| TRINITY_DN21723_c0_g3_i1 | GDSL esterase lipase 1 | 1407 | 8.61E-170 | 66.45 |
| TRINITY_DN21724_c0_g1_i1 | V-type proton ATPase catalytic subunit A | 2392 | 0 | 96.9 |
| TRINITY_DN21724_c0_g1_i2 | V-type proton ATPase catalytic subunit A | 2392 | 0 | 96.9 |
| TRINITY_DN21726_c1_g2_i1 | two-component response regulator-like APRR1 | 2100 | 0 | 67.05 |
| TRINITY_DN21726_c1_g2_i2 | two-component response regulator-like APRR1 | 2117 | 0 | 67.05 |
| TRINITY_DN21729_c0_g1_i1 | transcription repressor OFP1-like | 1057 | 8.77E-43 | 72.15 |
| TRINITY_DN2172_c0_g1_i1 | Arginine--tRNA ligase | 931 | 1.21E-33 | 67.9 |
| TRINITY_DN21732_c0_g1_i1 | probable glycerol-3-phosphate dehydrogenase [NAD(+)] cytosolic | 1190 | 6.56E-166 | 87.7 |
| TRINITY_DN21732_c0_g1_i2 | probable glycerol-3-phosphate dehydrogenase [NAD(+)] cytosolic | 1235 | 7.74E-170 | 87.7 |
| TRINITY_DN21732_c0_g1_i3 | probable glycerol-3-phosphate dehydrogenase [NAD(+)] cytosolic | 1185 | 6.08E-166 | 87.7 |
| TRINITY_DN21732_c0_g1_i4 | probable glycerol-3-phosphate dehydrogenase [NAD(+)] cytosolic | 1794 | 0 | 90.3 |
| TRINITY_DN21738_c0_g1_i1 | 2-dehydro-3-deoxyphosphooctonate aldolase 1 | 1515 | 0 | 94.35 |
| TRINITY_DN21738_c0_g1_i2 | 2-dehydro-3-deoxyphosphooctonate aldolase 1 | 1442 | 0 | 94.35 |
| TRINITY_DN21740_c0_g1_i1 | ---NA--- | 503 |  |  |
| TRINITY_DN21741_c1_g1_i5 | squalene synthase | 1510 | 0 | 88.2 |
| TRINITY_DN21741_c1_g1_i6 | squalene synthase | 1510 | 0 | 88.1 |
| TRINITY_DN21741_c1_g1_i7 | squalene synthase | 1526 | 0 | 88.1 |
| TRINITY_DN21741_c1_g1_i8 | squalene synthase | 1494 | 0 | 88.2 |
| TRINITY_DN21745_c0_g1_i1 | ---NA--- | 857 |  |  |
| TRINITY_DN21745_c0_g1_i2 | ---NA--- | 1070 |  |  |
| TRINITY_DN21745_c0_g1_i3 | ---NA--- | 1010 |  |  |
| TRINITY_DN21748_c0_g1_i1 | DUF616 domain-containing | 1655 | 0 | 78.8 |
| TRINITY_DN21748_c0_g1_i2 | DUF616 domain-containing | 1038 | 3.32E-178 | 81.75 |
| TRINITY_DN21749_c1_g2_i1 | ---NA--- | 763 |  |  |
| TRINITY_DN21749_c2_g2_i1 | lysM domain-containing GPI-anchored 2 | 1287 | 3.17E-129 | 71.6 |
| TRINITY_DN21749_c2_g2_i2 | lysM domain-containing GPI-anchored 2 | 1166 | 5.85E-118 | 74.55 |
| TRINITY_DN21749_c2_g2_i3 | lysM domain-containing GPI-anchored 2 | 1246 | 1.12E-123 | 73.3 |
| TRINITY_DN21755_c0_g1_i1 | ---NA--- | 534 |  |  |
| TRINITY_DN21755_c0_g1_i8 | ---NA--- | 561 |  |  |
| TRINITY_DN21757_c0_g2_i10 | L-ascorbate oxidase homolog | 1294 | 0 | 88.5 |
| TRINITY_DN21757_c0_g2_i14 | L-ascorbate oxidase homolog | 2022 | 0 | 88.65 |
| TRINITY_DN21757_c0_g2_i9 | L-ascorbate oxidase homolog | 2125 | 0 | 86.95 |
| TRINITY_DN21763_c0_g2_i1 | FRIGIDA 4a | 1002 | 2.73E-76 | 60.25 |
| TRINITY_DN21763_c0_g3_i1 | ---NA--- | 364 |  |  |
| TRINITY_DN21768_c0_g4_i1 | cytochrome c oxidase copper chaperone | 512 | 7.05E-51 | 76.85 |
| TRINITY_DN21768_c0_g4_i2 | cytochrome c oxidase copper chaperone 1 | 658 | 6.33E-45 | 76.9 |
| TRINITY_DN21768_c0_g5_i1 | ATP-binding cassette sub-family B member mitochondrial-like | 270 | 3.93E-15 | 59.35 |
| TRINITY_DN21768_c0_g7_i2 | NADH dehydrogenase [ubiquinone] complex assembly factor 7 | 2060 | 0 | 86 |
| TRINITY_DN21768_c0_g7_i5 | ATP-binding cassette sub-family B member mitochondrial-like | 325 | 2.27E-18 | 58.35 |
| TRINITY_DN21768_c0_g7_i6 | ATP-binding cassette sub-family B member mitochondrial-like | 227 | 1.04E-14 | 66.5 |
| TRINITY_DN21769_c0_g1_i1 | FREE1 | 1985 | 0 | 73.6 |
| TRINITY_DN21772_c0_g2_i3 | somatic embryogenesis receptor kinase 1-like | 1564 | 1.02E-45 | 77 |
| TRINITY_DN21772_c0_g2_i5 | somatic embryogenesis receptor kinase 1-like | 2113 | 6.45E-27 | 74.35 |
| TRINITY_DN21772_c0_g2_i6 | somatic embryogenesis receptor kinase 2-like isoform X1 | 1771 | 3.54E-37 | 78 |
| TRINITY_DN21774_c0_g2_i1 | PREDICTED: uncharacterized protein LOC104893175 | 2569 | 0 | 74.5 |
| TRINITY_DN21775_c0_g1_i4 | non-LTR retroelement reverse transcriptase-like | 1092 | 1.00E-19 | 52.5 |
| TRINITY_DN21778_c0_g1_i4 | alcohol dehydrogenase | 1867 | 0 | 89.6 |
| TRINITY_DN21781_c0_g1_i1 | epidermal growth factor receptor substrate 15 isoform X1 | 3532 | 0 | 63.55 |
| TRINITY_DN21781_c0_g1_i2 | epidermal growth factor receptor substrate 15-like 1 | 3380 | 0 | 65.25 |
| TRINITY_DN21786_c0_g1_i1 | arginine--tRNA cytoplasmic isoform X1 | 2255 | 0 | 88.25 |
| TRINITY_DN21786_c0_g1_i2 | arginine--tRNA cytoplasmic-like isoform X1 | 1504 | 0 | 84.8 |
| TRINITY_DN21787_c0_g1_i2 | ---NA--- | 725 |  |  |
| TRINITY_DN21787_c0_g1_i4 | WD repeat-containing 43 | 842 | 8.73E-11 | 43 |
| TRINITY_DN21789_c0_g2_i3 | hypothetical protein BVRB_7g162140 | 468 | 1.11E-29 | 72.9 |
| TRINITY_DN2178_c0_g1_i1 | Ribosomal RNA large subunit methyltransferase H | 1319 | 3.08E-62 | 71.35 |
| TRINITY_DN21790_c1_g1_i1 | nucleolar 14 | 3089 | 0 | 65.4 |
| TRINITY_DN21790_c1_g1_i2 | nucleolar 14 | 3092 | 0 | 70.65 |
| TRINITY_DN21791_c0_g1_i1 | probable WRKY transcription factor 20 isoform X1 | 1318 | 6.47E-170 | 69.1 |
| TRINITY_DN21791_c0_g1_i2 | probable WRKY transcription factor 20 isoform X1 | 2052 | 0 | 68.2 |
| TRINITY_DN21802_c0_g1_i3 | E3 SUMO- ligase SIZ1 isoform X1 | 3273 | 0 | 67.75 |
| TRINITY_DN21813_c0_g3_i1 | disease resistance RGA3 | 2665 | 4.27E-130 | 52.65 |
| TRINITY_DN21814_c0_g1_i1 | probable acyl-activating enzyme chloroplastic | 2627 | 0 | 82.25 |
| TRINITY_DN21817_c0_g1_i1 | Outer arm dynein light chain 1 isoform 1 | 2732 | 0 | 64.05 |
| TRINITY_DN21817_c0_g1_i2 | Outer arm dynein light chain 1 isoform 1 | 2928 | 0 | 64.05 |
| TRINITY_DN2181_c0_g1_i1 | transcription factor VIP1 | 1178 | 4.04E-80 | 76.45 |
| TRINITY_DN21824_c0_g5_i1 | ---NA--- | 334 |  |  |
| TRINITY_DN21824_c0_g6_i1 | ---NA--- | 233 |  |  |
| TRINITY_DN21829_c1_g1_i1 | probable ubiquitin-like-specific protease 2B isoform X2 | 2589 | 0 | 64.4 |
| TRINITY_DN21829_c1_g1_i2 | probable ubiquitin-like-specific protease 2B isoform X2 | 1495 | 1.11E-157 | 68.45 |
| TRINITY_DN21830_c1_g1_i1 | ---NA--- | 862 |  |  |
| TRINITY_DN21830_c1_g1_i2 | ---NA--- | 841 |  |  |
| TRINITY_DN21837_c0_g2_i1 | hypothetical chloroplast RF34 (chloroplast) | 3688 | 2.94E-46 | 98.2 |
| TRINITY_DN21839_c0_g1_i1 | myb family transcription factor EFM | 1146 | 1.39E-68 | 68.05 |
| TRINITY_DN21839_c0_g3_i1 | myb family transcription factor EFM | 1203 | 1.15E-25 | 87.3 |
| TRINITY_DN21839_c0_g3_i2 | myb family transcription factor EFM | 1050 | 1.27E-24 | 82.25 |
| TRINITY_DN21839_c0_g5_i1 | myb family transcription factor EFM | 661 | 1.33E-10 | 81 |
| TRINITY_DN2183_c0_g1_i1 | zinc finger CCCH domain-containing ZFN-like isoform X1 | 2022 | 0 | 83.4 |
| TRINITY_DN21840_c0_g1_i1 | ---NA--- | 723 |  |  |
| TRINITY_DN21840_c0_g2_i1 | Chromodomain-helicase-DNA-binding 5 | 3560 | 0 | 68.55 |
| TRINITY_DN21840_c0_g2_i9 | Chromodomain-helicase-DNA-binding 5 | 3571 | 0 | 68 |
| TRINITY_DN21841_c0_g1_i1 | serine threonine- kinase EDR1 isoform X1 | 3411 | 0 | 69.35 |
| TRINITY_DN21842_c1_g1_i1 | YLP motif-containing 1 | 2885 | 0 | 71 |
| TRINITY_DN21842_c1_g1_i2 | YLP motif-containing 1 isoform X2 | 2859 | 0 | 68.8 |
| TRINITY_DN21842_c1_g3_i1 | Ribosomal S21 family isoform 1 | 733 | 8.27E-37 | 72.15 |
| TRINITY_DN21846_c0_g1_i1 | Kelch-type beta propeller | 2050 | 1.66E-10 | 39.11 |
| TRINITY_DN21846_c0_g1_i2 | Kelch-type beta propeller | 1595 | 1.04E-10 | 39.11 |
| TRINITY_DN21851_c0_g1_i1 | ---NA--- | 832 |  |  |
| TRINITY_DN21852_c0_g1_i1 | SDA1 homolog | 2762 | 0 | 85.55 |
| TRINITY_DN21854_c0_g1_i1 | homeobox-leucine zipper ANTHOCYANINLESS 2 | 3081 | 0 | 84.35 |
| TRINITY_DN21860_c0_g1_i2 | interactor of constitutive active ROPs 3 isoform X1 | 2312 | 3.44E-165 | 62.55 |
| TRINITY_DN21864_c0_g3_i10 | ---NA--- | 327 |  |  |
| TRINITY_DN21878_c0_g1_i1 | ATP-dependent helicase BRM | 7030 | 0 | 78 |
| TRINITY_DN21878_c0_g1_i2 | ATP-dependent helicase BRM | 6956 | 0 | 78.15 |
| TRINITY_DN21879_c0_g1_i1 | spermatogenesis-associated 20 | 1399 | 0 | 89.25 |
| TRINITY_DN21879_c0_g1_i2 | spermatogenesis-associated 20 isoform X1 | 227 | 5.05E-23 | 85.75 |
| TRINITY_DN21879_c0_g1_i3 | spermatogenesis-associated 20 | 1381 | 0 | 88.75 |
| TRINITY_DN21879_c0_g2_i1 | spermatogenesis-associated 20 | 1331 | 0 | 83.6 |
| TRINITY_DN2187_c0_g1_i1 | soluble inorganic pyrophosphatase 1 | 1149 | 2.24E-133 | 92.8 |
| TRINITY_DN21888_c0_g1_i2 | ATP-dependent 6-phosphofructokinase 3 isoform X1 | 948 | 0 | 91.2 |
| TRINITY_DN21902_c0_g1_i1 | hypothetical protein SOVF_058080 | 903 | 3.03E-66 | 82.15 |
| TRINITY_DN21912_c0_g1_i1 | WEB family At5g55860 | 2433 | 0 | 76.8 |
| TRINITY_DN21912_c0_g1_i3 | WEB family At5g55860 | 2355 | 0 | 76.8 |
| TRINITY_DN21912_c0_g1_i4 | WEB family At5g55860 | 2409 | 0 | 76.8 |
| TRINITY_DN2191_c0_g1_i1 | CBL-interacting serine threonine- kinase 14 | 244 | 4.35E-15 | 74.7 |
| TRINITY_DN21921_c0_g1_i1 | mechanosensitive ion channel 6-like | 3536 | 0 | 75.65 |
| TRINITY_DN21922_c0_g3_i1 | PERQ amino acid-rich with GYF domain-containing | 5128 | 0 | 60.65 |
| TRINITY_DN21924_c0_g1_i1 | translation initiation factor eIF-2B subunit delta | 1305 | 0 | 88.35 |
| TRINITY_DN21928_c0_g1_i1 | Zinc-finger homeodomain 9 | 1461 | 2.33E-73 | 67.85 |
| TRINITY_DN21928_c0_g1_i2 | calcium sensing chloroplastic | 2092 | 0 | 81.05 |
| TRINITY_DN21928_c0_g1_i3 | calcium sensing chloroplastic | 2047 | 1.77E-170 | 78.15 |
| TRINITY_DN21929_c0_g1_i3 | PREDICTED: uncharacterized protein LOC100839848 | 1738 | 2.15E-07 | 40 |
| TRINITY_DN2193_c1_g1_i1 | translationally-controlled tumor homolog | 1073 | 3.44E-93 | 86.8 |
| TRINITY_DN21940_c5_g2_i1 | PGR5 chloroplastic | 1676 | 6.82E-99 | 74.45 |
| TRINITY_DN21940_c5_g2_i2 | PGR5 chloroplastic | 1310 | 3.07E-108 | 77.35 |
| TRINITY_DN21941_c0_g1_i1 | hypothetical protein MP_TR23805_c0_g1_i1_g.69444, partial | 566 | 1.32E-19 | 74.75 |
| TRINITY_DN21941_c0_g1_i2 | dynamin-related 1C | 2933 | 0 | 93.5 |
| TRINITY_DN21941_c0_g1_i3 | hypothetical protein MP_TR23805_c0_g1_i1_g.69444, partial | 565 | 6.75E-20 | 75.85 |
| TRINITY_DN21941_c0_g1_i4 | dynamin-related 1C | 2897 | 0 | 93.5 |
| TRINITY_DN21943_c0_g5_i1 | NEDD8-specific protease 1 | 1438 | 7.47E-106 | 72.5 |
| TRINITY_DN21946_c0_g1_i1 | CRAL_TRIO domain-containing | 2188 | 0 | 69.4 |
| TRINITY_DN21946_c0_g1_i2 | CRAL_TRIO domain-containing | 1886 | 0 | 69.4 |
| TRINITY_DN21946_c0_g1_i4 | CRAL_TRIO domain-containing | 2062 | 0 | 69.4 |
| TRINITY_DN21951_c0_g1_i1 | DUF707 domain-containing | 1662 | 0 | 81.25 |
| TRINITY_DN21951_c0_g1_i2 | DUF707 domain-containing | 1837 | 0 | 81.25 |
| TRINITY_DN21951_c0_g1_i3 | hypothetical protein SOVF_137340 | 1662 | 0 | 80.55 |
| TRINITY_DN21951_c0_g1_i4 | hypothetical protein SOVF_137340 | 1837 | 0 | 79.4 |
| TRINITY_DN21951_c0_g1_i5 | hypothetical protein SOVF_137340 | 1662 | 0 | 79.4 |
| TRINITY_DN21953_c1_g1_i1 | ---NA--- | 377 |  |  |
| TRINITY_DN21954_c0_g1_i1 | ---NA--- | 471 |  |  |
| TRINITY_DN21954_c5_g1_i1 | ---NA--- | 333 |  |  |
| TRINITY_DN21954_c5_g1_i3 | ---NA--- | 261 |  |  |
| TRINITY_DN21954_c7_g1_i1 | E3 ubiquitin- ligase XBAT31 | 451 | 2.64E-50 | 79.4 |
| TRINITY_DN21956_c1_g1_i1 | STRUBBELIG-RECEPTOR FAMILY 3 isoform X1 | 1698 | 4.41E-129 | 68.4 |
| TRINITY_DN21956_c1_g1_i2 | STRUBBELIG-RECEPTOR FAMILY 3 isoform X1 | 1477 | 6.15E-128 | 68.45 |
| TRINITY_DN21958_c0_g1_i1 | BOLA2 | 645 | 5.76E-45 | 92.8 |
| TRINITY_DN21958_c2_g1_i2 | disease resistance RGA2-like | 2386 | 2.94E-171 | 53.75 |
| TRINITY_DN2195_c0_g1_i1 | nucleosome assembly 1 4-like isoform X2 | 224 | 1.07E-11 | 100 |
| TRINITY_DN2195_c1_g1_i1 | 60S ribosomal L18a | 1026 | 8.85E-23 | 72.75 |
| TRINITY_DN21961_c0_g1_i2 | nucleolar MIF4G domain-containing 1 | 2678 | 0 | 78.75 |
| TRINITY_DN21962_c0_g2_i1 | ---NA--- | 1230 |  |  |
| TRINITY_DN21962_c0_g2_i2 | ---NA--- | 1205 |  |  |
| TRINITY_DN21964_c1_g2_i1 | ---NA--- | 233 |  |  |
| TRINITY_DN21966_c0_g1_i1 | sphingomyelin phosphodiesterase 4 | 2748 | 0 | 71.2 |
| TRINITY_DN21967_c0_g1_i1 | ---NA--- | 219 |  |  |
| TRINITY_DN21967_c0_g2_i1 | glutamyl-tRNA reductase chloroplastic | 2301 | 0 | 94.95 |
| TRINITY_DN21967_c0_g3_i1 | ---NA--- | 270 |  |  |
| TRINITY_DN21969_c1_g2_i1 | calcium-dependent kinase 13 | 1883 | 0 | 91.15 |
| TRINITY_DN21969_c1_g2_i2 | calcium-dependent kinase 13 | 1985 | 0 | 91.7 |
| TRINITY_DN21976_c0_g1_i1 | shewanella phosphatase 1 | 3314 | 0 | 84.75 |
| TRINITY_DN21976_c0_g1_i2 | shewanella phosphatase 1 | 1806 | 0 | 84.75 |
| TRINITY_DN21976_c0_g1_i3 | shewanella phosphatase 1 | 3283 | 0 | 84.75 |
| TRINITY_DN21976_c0_g1_i4 | shewanella phosphatase 1 | 2126 | 0 | 84.75 |
| TRINITY_DN21976_c0_g1_i5 | shewanella phosphatase 1 | 1837 | 0 | 84.75 |
| TRINITY_DN21976_c0_g1_i6 | shewanella phosphatase 1 | 2095 | 0 | 84.75 |
| TRINITY_DN21978_c0_g1_i1 | ---NA--- | 2638 |  |  |
| TRINITY_DN21978_c0_g1_i2 | ---NA--- | 2416 |  |  |
| TRINITY_DN21985_c0_g1_i1 | hypothetical protein SOVF_033390 | 1785 | 1.18E-131 | 64.6 |
| TRINITY_DN21986_c0_g1_i1 | ---NA--- | 890 |  |  |
| TRINITY_DN21986_c0_g1_i2 | ---NA--- | 873 |  |  |
| TRINITY_DN21986_c0_g1_i3 | ---NA--- | 903 |  |  |
| TRINITY_DN21986_c0_g1_i4 | ---NA--- | 886 |  |  |
| TRINITY_DN21986_c0_g1_i5 | ---NA--- | 796 |  |  |
| TRINITY_DN21986_c0_g1_i6 | transcription factor E2FB | 813 | 3.29E-10 | 75.4 |
| TRINITY_DN21993_c0_g1_i2 | vacuolar sorting-associated 35A | 2867 | 0 | 90.65 |
| TRINITY_DN21997_c0_g1_i4 | ---NA--- | 1075 |  |  |
| TRINITY_DN21999_c0_g1_i1 | plasminogen activator inhibitor 1 RNA-binding -like | 1822 | 3.08E-62 | 71.9 |
| TRINITY_DN22007_c0_g1_i1 | E3 ubiquitin- ligase RGLG2-like | 2147 | 0 | 82.55 |
| TRINITY_DN22014_c0_g1_i4 | phox domain-containing | 2840 | 0 | 67.3 |
| TRINITY_DN22014_c0_g1_i5 | Sorting nexin-16 | 3467 | 0 | 69.05 |
| TRINITY_DN22014_c0_g1_i7 | phox domain-containing | 3452 | 0 | 68.9 |
| TRINITY_DN22028_c0_g1_i1 | rab3 GTPase-activating catalytic subunit isoform X2 | 3245 | 0 | 78.6 |
| TRINITY_DN22028_c0_g1_i2 | rab3 GTPase-activating catalytic subunit isoform X2 | 1481 | 1.46E-138 | 85.2 |
| TRINITY_DN22028_c0_g1_i3 | rab3 GTPase-activating catalytic subunit isoform X2 | 1516 | 1.22E-175 | 80.8 |
| TRINITY_DN22028_c0_g1_i4 | rab3 GTPase-activating catalytic subunit isoform X2 | 3263 | 0 | 78.3 |
| TRINITY_DN22028_c0_g1_i5 | rab3 GTPase-activating catalytic subunit isoform X1 | 1498 | 4.90E-178 | 82.45 |
| TRINITY_DN22028_c0_g1_i6 | rab3 GTPase-activating catalytic subunit isoform X1 | 3246 | 0 | 78.55 |
| TRINITY_DN22028_c0_g1_i7 | rab3 GTPase-activating catalytic subunit isoform X1 | 1499 | 7.60E-137 | 85.15 |
| TRINITY_DN22031_c0_g1_i2 | probable pectate lyase 8 | 1923 | 0 | 87.45 |
| TRINITY_DN22037_c0_g1_i1 | PREDICTED: uncharacterized protein LOC104894795 | 1490 | 6.61E-122 | 63.35 |
| TRINITY_DN22037_c0_g1_i2 | PREDICTED: uncharacterized protein LOC104894795 | 611 | 5.88E-36 | 56.15 |
| TRINITY_DN22039_c0_g1_i1 | Phox Bem1p | 1728 | 5.94E-77 | 61.45 |
| TRINITY_DN2203_c0_g1_i1 | fatty acid amide hydrolase | 2067 | 0 | 81.8 |
| TRINITY_DN2203_c0_g1_i2 | fatty acid amide hydrolase | 1985 | 0 | 81 |
| TRINITY_DN22040_c0_g2_i1 | ---NA--- | 451 |  |  |
| TRINITY_DN22041_c0_g1_i1 | pre-mRNA 3 -end-processing factor FIP1-like | 1359 | 4.10E-34 | 62.15 |
| TRINITY_DN22049_c0_g1_i1 | hypothetical protein SOVF_195290 | 1677 | 0 | 91.85 |
| TRINITY_DN22049_c0_g1_i2 | hypothetical protein SOVF_195290 | 1668 | 0 | 91.6 |
| TRINITY_DN22049_c0_g1_i3 | hypothetical protein SOVF_195290 | 1203 | 0 | 94.35 |
| TRINITY_DN22049_c1_g1_i1 | chaperone dnaJ chloroplastic | 456 | 8.20E-35 | 72.35 |
| TRINITY_DN22050_c0_g1_i1 | ---NA--- | 209 |  |  |
| TRINITY_DN22050_c0_g1_i2 | ---NA--- | 387 |  |  |
| TRINITY_DN22050_c0_g1_i3 | ---NA--- | 367 |  |  |
| TRINITY_DN22053_c0_g1_i1 | 60S ribosomal export NMD3-like | 1996 | 1.57E-180 | 67.9 |
| TRINITY_DN22055_c3_g2_i1 | oxygen-independent coproporphyrinogen-III oxidase sll1917 | 1548 | 0 | 79.35 |
| TRINITY_DN22055_c3_g3_i1 | oxygen-independent coproporphyrinogen-III oxidase sll1917 | 634 | 1.10E-30 | 51.25 |
| TRINITY_DN22055_c3_g3_i2 | oxygen-independent coproporphyrinogen-III oxidase sll1917 | 648 | 4.18E-30 | 50.8 |
| TRINITY_DN22055_c3_g3_i3 | oxygen-independent coproporphyrinogen-III oxidase sll1917 | 654 | 1.68E-30 | 50.45 |
| TRINITY_DN22055_c3_g3_i4 | oxygen-independent coproporphyrinogen-III oxidase sll1917 | 883 | 4.07E-29 | 50.65 |
| TRINITY_DN22055_c3_g6_i1 | LRR receptor-like serine threonine- kinase GSO1 | 352 | 7.06E-37 | 74.95 |
| TRINITY_DN22057_c0_g1_i1 | probable methyltransferase PMT3 | 2481 | 0 | 88.1 |
| TRINITY_DN22057_c0_g1_i2 | probable methyltransferase PMT3 | 2481 | 0 | 88.8 |
| TRINITY_DN22058_c2_g1_i1 | 50S ribosomal mitochondrial-like | 764 | 8.91E-75 | 82.7 |
| TRINITY_DN22058_c2_g1_i2 | 50S ribosomal mitochondrial-like | 987 | 1.14E-73 | 82.7 |
| TRINITY_DN22058_c3_g1_i1 | CWC15 homolog | 1210 | 2.60E-89 | 87.35 |
| TRINITY_DN22062_c1_g2_i1 | SUPPRESSOR OF FRI 4-like isoform X1 | 1386 | 3.08E-24 | 77.5 |
| TRINITY_DN22062_c1_g3_i1 | SUPPRESSOR OF FRI 4 isoform X1 | 1226 | 3.65E-112 | 72.2 |
| TRINITY_DN22062_c1_g3_i3 | SUPPRESSOR OF FRI 4 isoform X2 | 1321 | 3.54E-133 | 65.05 |
| TRINITY_DN22062_c1_g5_i1 | SUPPRESSOR OF FRI 4 isoform X2 | 1190 | 3.81E-138 | 70.9 |
| TRINITY_DN22067_c5_g1_i1 | ---NA--- | 592 |  |  |
| TRINITY_DN22067_c7_g1_i1 | KINESIN LIGHT CHAIN-RELATED 1 | 3842 | 3.99E-126 | 68.6 |
| TRINITY_DN22067_c7_g1_i4 | KINESIN LIGHT CHAIN-RELATED 1 | 4499 | 5.23E-90 | 63.95 |
| TRINITY_DN22067_c7_g2_i1 | ---NA--- | 2347 |  |  |
| TRINITY_DN22076_c1_g1_i1 | myosin-1 | 4111 | 0 | 87.7 |
| TRINITY_DN22076_c1_g1_i2 | myosin-1 | 4029 | 0 | 84.25 |
| TRINITY_DN22076_c1_g1_i3 | myosin-1 | 4087 | 0 | 86.8 |
| TRINITY_DN22077_c1_g3_i1 | F-box At1g78280 isoform X2 | 553 | 8.65E-18 | 70.8 |
| TRINITY_DN22077_c1_g3_i3 | F-box At1g78280 isoform X1 | 575 | 7.99E-13 | 65.95 |
| TRINITY_DN22077_c1_g3_i4 | hypothetical protein SOVF_097330 | 348 | 6.12E-06 | 71 |
| TRINITY_DN22081_c0_g1_i1 | ---NA--- | 1215 |  |  |
| TRINITY_DN22081_c0_g1_i2 | hypothetical protein SOVF_075740 | 803 | 2.22E-07 | 56 |
| TRINITY_DN22081_c1_g1_i1 | nodal modulator 1 | 4095 | 0 | 79.95 |
| TRINITY_DN22082_c1_g1_i4 | ureidoglycolate hydrolase | 1926 | 0 | 87.4 |
| TRINITY_DN22082_c1_g1_i5 | ---NA--- | 1887 |  |  |
| TRINITY_DN22082_c1_g1_i6 | ureidoglycolate hydrolase | 1994 | 0 | 87.4 |
| TRINITY_DN22084_c0_g1_i1 | polygalacturonate 4-alpha-galacturonosyltransferase | 2415 | 0 | 88.15 |
| TRINITY_DN22085_c0_g1_i1 | ECERIFERUM 26-like | 1688 | 1.23E-164 | 65.6 |
| TRINITY_DN22088_c0_g2_i2 | histone-lysine N-methyltransferase | 3103 | 0 | 68.8 |
| TRINITY_DN22088_c0_g2_i3 | histone-lysine N-methyltransferase | 2749 | 0 | 69.9 |
| TRINITY_DN22090_c0_g1_i2 | ---NA--- | 1002 |  |  |
| TRINITY_DN22090_c0_g1_i3 | ---NA--- | 861 |  |  |
| TRINITY_DN22090_c0_g1_i4 | ---NA--- | 680 |  |  |
| TRINITY_DN22097_c0_g1_i1 | phosphoinositide phosphatase SAC2-like | 2647 | 0 | 66.35 |
| TRINITY_DN22099_c0_g2_i1 | hexokinase-3-like | 262 | 6.11E-14 | 83.95 |
| TRINITY_DN22099_c0_g3_i1 | hexokinase-3 isoform X1 | 1498 | 0 | 86.7 |
| TRINITY_DN22101_c3_g1_i1 | vacuolar sorting-associated 25 | 921 | 2.14E-90 | 92.3 |
| TRINITY_DN22101_c3_g1_i2 | vacuolar sorting-associated 25 | 1126 | 6.70E-119 | 95.95 |
| TRINITY_DN22102_c0_g2_i1 | ---NA--- | 230 |  |  |
| TRINITY_DN22102_c0_g2_i3 | pentatricopeptide repeat-containing At1g56570 | 1377 | 6.14E-113 | 79.6 |
| TRINITY_DN22103_c0_g1_i1 | exportin-T isoform X1 | 2491 | 0 | 85.25 |
| TRINITY_DN22103_c0_g1_i2 | exportin-T isoform X1 | 708 | 1.45E-23 | 81.7 |
| TRINITY_DN22104_c0_g1_i1 | ---NA--- | 715 |  |  |
| TRINITY_DN22106_c0_g1_i1 | E3 ubiquitin- ligase BRE1-like 1 isoform X1 | 3245 | 0 | 72.15 |
| TRINITY_DN22106_c0_g1_i2 | E3 ubiquitin- ligase BRE1-like 1 | 3319 | 0 | 77.6 |
| TRINITY_DN22117_c0_g2_i1 | probable xyloglucan glycosyltransferase 6 | 2989 | 0 | 90.95 |
| TRINITY_DN22118_c0_g3_i1 | MORN repeat-containing 1 | 1425 | 1.03E-131 | 78.55 |
| TRINITY_DN22118_c0_g3_i2 | phosphatidylinositol-4-phosphate 5-kinase | 1096 | 1.31E-118 | 76.1 |
| TRINITY_DN22119_c1_g2_i1 | tubby-like F-box 7 | 549 | 2.04E-23 | 75.45 |
| TRINITY_DN22119_c1_g6_i1 | PREDICTED: uncharacterized protein LOC104897980 | 3027 | 0 | 69.85 |
| TRINITY_DN22121_c0_g1_i1 | Galactose mutarotase-like domain-containing | 1157 | 7.78E-12 | 63.55 |
| TRINITY_DN22121_c0_g1_i10 | Fatty acid hydroxylase superfamily isoform 2 | 1292 | 8.97E-23 | 80.15 |
| TRINITY_DN22121_c0_g1_i11 | alpha-mannosidase isoform X2 | 1303 | 5.12E-10 | 87.7 |
| TRINITY_DN22121_c0_g1_i12 | alpha-mannosidase isoform X2 | 371 | 1.06E-12 | 82.8 |
| TRINITY_DN22121_c0_g1_i13 | alpha-mannosidase isoform X2 | 1202 | 4.55E-10 | 75.8 |
| TRINITY_DN22121_c0_g1_i14 | ---NA--- | 593 |  |  |
| TRINITY_DN22121_c0_g1_i15 | alpha-mannosidase isoform X2 | 1219 | 4.65E-10 | 78.2 |
| TRINITY_DN22121_c0_g1_i16 | Galactose mutarotase-like domain-containing | 980 | 5.52E-10 | 70.5 |
| TRINITY_DN22121_c0_g1_i17 | Galactose mutarotase-like domain-containing | 879 | 9.44E-12 | 63.82 |
| TRINITY_DN22121_c0_g1_i18 | ---NA--- | 570 |  |  |
| TRINITY_DN22121_c0_g1_i19 | Fatty acid hydroxylase superfamily isoform 2 | 1553 | 1.14E-22 | 80.15 |
| TRINITY_DN22121_c0_g1_i2 | alpha-mannosidase isoform X2 | 1480 | 6.02E-10 | 78.2 |
| TRINITY_DN22121_c0_g1_i20 | ---NA--- | 643 |  |  |
| TRINITY_DN22121_c0_g1_i21 | ---NA--- | 848 |  |  |
| TRINITY_DN22121_c0_g1_i3 | Fatty acid hydroxylase superfamily isoform 2 | 1275 | 8.82E-23 | 80.15 |
| TRINITY_DN22121_c0_g1_i4 | alpha-mannosidase-like | 1208 | 5.39E-24 | 80.15 |
| TRINITY_DN22121_c0_g1_i6 | alpha-mannosidase-like | 1191 | 5.29E-24 | 80.15 |
| TRINITY_DN22121_c0_g1_i7 | Galactose mutarotase-like domain-containing | 896 | 2.07E-12 | 63.55 |
| TRINITY_DN22121_c0_g1_i8 | alpha-mannosidase-like | 1469 | 7.01E-24 | 80.15 |
| TRINITY_DN22121_c0_g1_i9 | 2-phosphoglycolate phosphatase 1 | 587 | 1.07E-09 | 99.8 |
| TRINITY_DN22122_c1_g1_i2 | transcription factor LHW | 1445 | 0 | 85 |
| TRINITY_DN22122_c1_g1_i3 | transcription factor LHW | 1456 | 0 | 81.8 |
| TRINITY_DN22122_c2_g1_i1 | BEL1-like homeodomain 1 | 3323 | 0 | 59.85 |
| TRINITY_DN22126_c0_g4_i1 | ---NA--- | 1183 |  |  |
| TRINITY_DN22126_c0_g4_i2 | ---NA--- | 1038 |  |  |
| TRINITY_DN22126_c0_g4_i3 | ---NA--- | 1258 |  |  |
| TRINITY_DN22126_c0_g4_i4 | ---NA--- | 1113 |  |  |
| TRINITY_DN22136_c0_g1_i1 | pollen-specific SF21-like | 1329 | 7.57E-159 | 90.5 |
| TRINITY_DN22136_c0_g1_i2 | pollen-specific SF21-like | 1318 | 0 | 91.15 |
| TRINITY_DN22137_c1_g2_i1 | F-box SKIP23-like | 918 | 7.78E-15 | 48.29 |
| TRINITY_DN22137_c2_g1_i1 | hypothetical protein BVRB_5g118720 | 1151 | 1.54E-19 | 46.14 |
| TRINITY_DN22142_c1_g2_i3 | PREDICTED: uncharacterized protein At4g26485-like | 567 | 5.67E-56 | 73.35 |
| TRINITY_DN22142_c1_g2_i4 | PREDICTED: uncharacterized protein At4g26485-like | 486 | 9.89E-43 | 60.3 |
| TRINITY_DN22143_c1_g3_i3 | E3 ubiquitin- ligase RHF2A | 1882 | 2.99E-174 | 74.75 |
| TRINITY_DN22143_c1_g3_i7 | E3 ubiquitin- ligase RHF2A | 1879 | 4.75E-180 | 76.2 |
| TRINITY_DN22143_c1_g4_i1 | transcription factor UNE12 | 1504 | 3.89E-126 | 77.75 |
| TRINITY_DN22143_c1_g5_i1 | ---NA--- | 343 |  |  |
| TRINITY_DN22145_c2_g1_i1 | ---NA--- | 270 |  |  |
| TRINITY_DN22146_c0_g1_i3 | morphogenetic regulator of filamentous growth 1-like | 909 | 4.16E-76 | 66.1 |
| TRINITY_DN22150_c0_g1_i1 | Wall-associated receptor kinase-like 20 | 1499 | 1.48E-172 | 76.25 |
| TRINITY_DN22158_c0_g1_i1 | GBF-interacting 1-like isoform X1 | 1192 | 1.60E-77 | 66.45 |
| TRINITY_DN22158_c0_g1_i2 | GBF-interacting 1-like isoform X4 | 2750 | 0 | 53.7 |
| TRINITY_DN22160_c0_g1_i1 | Serine threonine- kinase fray2 | 2361 | 0 | 74.15 |
| TRINITY_DN22160_c0_g1_i2 | serine threonine- kinase BLUS1-like isoform X3 | 2349 | 0 | 74.35 |
| TRINITY_DN22160_c0_g1_i3 | Serine threonine- kinase fray2 | 2361 | 0 | 74.4 |
| TRINITY_DN22160_c0_g1_i5 | serine threonine- kinase BLUS1-like isoform X3 | 2349 | 0 | 73.65 |
| TRINITY_DN22162_c0_g1_i1 | ---NA--- | 479 |  |  |
| TRINITY_DN22162_c0_g2_i1 | glucan endo-1,3-beta-glucosidase 14 | 1736 | 0 | 84.65 |
| TRINITY_DN22162_c0_g2_i2 | glucan endo-1,3-beta-glucosidase 14 | 1919 | 0 | 84.6 |
| TRINITY_DN22162_c0_g2_i3 | glucan endo-1,3-beta-glucosidase 14 | 1930 | 0 | 84.6 |
| TRINITY_DN22162_c0_g2_i4 | glucan endo-1,3-beta-glucosidase 14 | 1725 | 0 | 84.65 |
| TRINITY_DN22164_c0_g1_i1 | FER2_PHYAM ame: Full=Ferredoxin-2 ame: Full=Ferredoxin II | 306 | 7.48E-18 | 65.75 |
| TRINITY_DN22164_c0_g2_i1 | N-acetylglucosaminyl-phosphatidylinositol de-N-acetylase-like isoform X1 | 1245 | 4.90E-141 | 76.55 |
| TRINITY_DN22165_c0_g1_i1 | DNA repair helicase XPD isoform X1 | 2582 | 0 | 89.95 |
| TRINITY_DN22165_c0_g1_i2 | DNA repair helicase XPD isoform X1 | 2690 | 0 | 93.7 |
| TRINITY_DN22171_c1_g1_i2 | phosphatidylinositol phosphatidylcholine transfer SFH8 isoform X1 | 2284 | 0 | 70.75 |
| TRINITY_DN22176_c0_g1_i10 | Endoplasmic reticulum metallopeptidase 1 | 1463 | 0 | 85.1 |
| TRINITY_DN22176_c0_g1_i4 | endoplasmic reticulum metallopeptidase 1 | 1099 | 6.47E-178 | 88.8 |
| TRINITY_DN22176_c0_g1_i6 | endoplasmic reticulum metallopeptidase 1 | 2826 | 0 | 80.55 |
| TRINITY_DN22186_c0_g1_i1 | serine threonine- kinase | 1602 | 0 | 80.45 |
| TRINITY_DN2218_c0_g1_i1 | non-canonical poly(A) RNA polymerase PAPD5 | 2024 | 0 | 77.35 |
| TRINITY_DN22193_c0_g1_i3 | Ribosomal L7Ae L30e S12e Gadd45 | 1332 | 1.41E-60 | 70.8 |
| TRINITY_DN22193_c0_g1_i4 | Ribosomal L7Ae L30e S12e Gadd45 | 1320 | 1.40E-60 | 70.85 |
| TRINITY_DN22193_c0_g3_i1 | ATP-dependent zinc metalloprotease FTSH chloroplastic | 3646 | 0 | 88.8 |
| TRINITY_DN22196_c0_g3_i1 | transcription factor MYB51-like isoform X1 | 377 | 2.28E-52 | 88.85 |
| TRINITY_DN22197_c0_g1_i1 | thioredoxin domain-containing PLP3B-like | 1243 | 2.97E-92 | 90.8 |
| TRINITY_DN22198_c0_g1_i2 | GBF-interacting 1-like isoform X2 | 2083 | 0 | 68.95 |
| TRINITY_DN22200_c0_g1_i1 | FRIGIDA-ESSENTIAL 1 isoform X1 | 3092 | 2.77E-125 | 51.75 |
| TRINITY_DN22200_c0_g1_i3 | FRIGIDA-ESSENTIAL 1 isoform X1 | 3894 | 5.03E-123 | 51.75 |
| TRINITY_DN22201_c0_g1_i1 | dnaJ homolog subfamily C member 2-like | 2453 | 0 | 81.7 |
| TRINITY_DN22201_c0_g1_i2 | dnaJ homolog subfamily C member 2-like | 2793 | 0 | 81.1 |
| TRINITY_DN22201_c0_g1_i3 | dnaJ homolog subfamily C member 2-like | 1330 | 6.43E-122 | 91.5 |
| TRINITY_DN22201_c0_g1_i4 | dnaJ homolog subfamily C member 2-like | 2793 | 0 | 81.1 |
| TRINITY_DN22206_c0_g1_i1 | ---NA--- | 223 |  |  |
| TRINITY_DN22206_c0_g1_i2 | scarecrow 22 | 1642 | 0 | 76.15 |
| TRINITY_DN22208_c2_g1_i1 | ---NA--- | 780 |  |  |
| TRINITY_DN22208_c2_g1_i2 | ---NA--- | 788 |  |  |
| TRINITY_DN22212_c2_g1_i12 | PREDICTED: uncharacterized protein LOC104883495 | 3000 | 6.99E-20 | 44.32 |
| TRINITY_DN22212_c2_g1_i8 | AC099400_1 poly | 1615 | 1.35E-16 | 43.7 |
| TRINITY_DN22212_c2_g1_i9 | PREDICTED: uncharacterized protein LOC104883495 | 2584 | 1.04E-15 | 45.47 |
| TRINITY_DN22213_c0_g1_i1 | ---NA--- | 223 |  |  |
| TRINITY_DN22213_c0_g2_i1 | polyadenylation and cleavage factor homolog 4 isoform X2 | 3596 | 0 | 53.85 |
| TRINITY_DN22217_c0_g2_i1 | ACT domain-containing ACR10-like | 1766 | 0 | 81.15 |
| TRINITY_DN22219_c0_g2_i1 | violaxanthin de- chloroplastic | 2683 | 0 | 84.2 |
| TRINITY_DN22221_c0_g1_i4 | sodium calcium exchanger | 2194 | 0 | 82.45 |
| TRINITY_DN22222_c0_g1_i1 | sister chromatid cohesion PDS5 homolog A isoform X1 | 5382 | 0 | 72.5 |
| TRINITY_DN22222_c0_g1_i2 | sister chromatid cohesion PDS5 homolog A isoform X1 | 5395 | 0 | 72.5 |
| TRINITY_DN22224_c0_g1_i1 | quinolinate chloroplastic | 2995 | 0 | 83.1 |
| TRINITY_DN22226_c0_g1_i1 | alpha-1,4 glucan phosphorylase L-2 chloroplastic amyloplastic-like | 3563 | 0 | 85.25 |
| TRINITY_DN22227_c0_g2_i1 | retrotransposon Ty3-gypsy subclass | 2104 | 4.74E-70 | 76.35 |
| TRINITY_DN22227_c0_g2_i2 | retrotransposon Ty3-gypsy subclass | 1957 | 4.35E-70 | 76.35 |
| TRINITY_DN22227_c0_g2_i3 | retrotransposon Ty3-gypsy subclass | 2062 | 4.62E-70 | 76.35 |
| TRINITY_DN22227_c0_g2_i5 | retrotransposon Ty3-gypsy subclass | 2041 | 4.56E-70 | 76.35 |
| TRINITY_DN22228_c0_g2_i1 | ---NA--- | 823 |  |  |
| TRINITY_DN22231_c0_g1_i2 | ALA-interacting subunit 3-like | 1304 | 0 | 87 |
| TRINITY_DN22238_c0_g1_i1 | NADPH-dependent diflavin oxidoreductase 1 isoform X1 | 2162 | 0 | 80.25 |
| TRINITY_DN22238_c0_g1_i2 | NADPH-dependent diflavin oxidoreductase 1 isoform X1 | 2202 | 0 | 80.2 |
| TRINITY_DN22242_c0_g3_i1 | probable transcriptional regulatory At2g25830 | 1245 | 4.69E-124 | 86 |
| TRINITY_DN22242_c0_g3_i2 | probable transcriptional regulatory At2g25830 isoform X1 | 1149 | 1.16E-93 | 72.9 |
| TRINITY_DN22242_c0_g3_i3 | probable transcriptional regulatory At2g25830 isoform X2 | 683 | 1.38E-07 | 72.73 |
| TRINITY_DN22245_c0_g1_i1 | THUMP domain-containing 1 homolog | 1428 | 2.19E-153 | 71.2 |
| TRINITY_DN22245_c0_g1_i4 | THUMP domain-containing 1 homolog | 1392 | 4.52E-158 | 71.2 |
| TRINITY_DN22245_c0_g1_i6 | THUMP domain-containing 1 homolog | 1549 | 5.85E-148 | 65.5 |
| TRINITY_DN22247_c1_g1_i1 | serine threonine- phosphatase 6 regulatory ankyrin repeat subunit A isoform X2 | 568 | 2.27E-30 | 53.1 |
| TRINITY_DN22248_c1_g2_i1 | ---NA--- | 1053 |  |  |
| TRINITY_DN22255_c0_g1_i2 | DNA topoisomerase 3-alpha | 1049 | 1.21E-16 | 65.85 |
| TRINITY_DN22255_c0_g1_i3 | DNA topoisomerase 3-alpha isoform X1 | 866 | 4.28E-18 | 67.9 |
| TRINITY_DN22255_c0_g1_i6 | DNA topoisomerase 3-alpha isoform X2 | 852 | 3.25E-16 | 69 |
| TRINITY_DN22266_c0_g1_i1 | A G-specific adenine DNA glycosylase | 1357 | 4.07E-136 | 78.8 |
| TRINITY_DN22266_c0_g1_i2 | adenine DNA glycosylase | 1630 | 0 | 78.95 |
| TRINITY_DN22266_c0_g1_i3 | adenine DNA glycosylase | 1614 | 1.84E-160 | 76.6 |
| TRINITY_DN22266_c0_g1_i4 | A G-specific adenine DNA glycosylase | 1341 | 4.45E-99 | 81 |
| TRINITY_DN22273_c0_g1_i2 | NAR1 | 1569 | 0 | 87.6 |
| TRINITY_DN22273_c0_g2_i1 | cytosolic Fe-S cluster assembly factor narfl | 299 | 2.52E-31 | 93.5 |
| TRINITY_DN22273_c1_g1_i1 | ---NA--- | 479 |  |  |
| TRINITY_DN22287_c0_g1_i1 | cytochrome P450 CYP72A219-like | 2104 | 0 | 78.25 |
| TRINITY_DN22287_c0_g1_i2 | cytochrome P450 CYP72A219-like | 684 | 3.36E-89 | 77.65 |
| TRINITY_DN22287_c0_g1_i4 | cytochrome P450 CYP72A219-like | 2104 | 0 | 79.2 |
| TRINITY_DN22291_c0_g2_i1 | ---NA--- | 539 |  |  |
| TRINITY_DN22291_c0_g2_i3 | ---NA--- | 652 |  |  |
| TRINITY_DN22291_c0_g2_i4 | ---NA--- | 279 |  |  |
| TRINITY_DN22291_c0_g2_i6 | ---NA--- | 623 |  |  |
| TRINITY_DN22291_c0_g2_i7 | ---NA--- | 617 |  |  |
| TRINITY_DN22291_c0_g2_i8 | ---NA--- | 568 |  |  |
| TRINITY_DN22291_c0_g3_i1 | ---NA--- | 389 |  |  |
| TRINITY_DN22294_c0_g2_i1 | phospholipase D Z | 1850 | 0 | 83.1 |
| TRINITY_DN22294_c0_g2_i2 | probable serine threonine- kinase DDB_G0272282 | 1655 | 2.21E-48 | 57.7 |
| TRINITY_DN22294_c0_g2_i3 | phospholipase D Z | 1770 | 0 | 83.1 |
| TRINITY_DN22297_c0_g1_i1 | calmodulin-binding transcription activator 4 isoform X1 | 3328 | 0 | 70.9 |
| TRINITY_DN222_c0_g1_i1 | probable sucrose-phosphate synthase 1 | 3440 | 0 | 83.8 |
| TRINITY_DN22301_c0_g1_i1 | NDR1 HIN1 10 | 1130 | 3.53E-38 | 62.95 |
| TRINITY_DN22303_c0_g3_i4 | ---NA--- | 575 |  |  |
| TRINITY_DN22303_c0_g3_i6 | ---NA--- | 586 |  |  |
| TRINITY_DN22303_c0_g4_i1 | ---NA--- | 917 |  |  |
| TRINITY_DN22303_c0_g4_i2 | ---NA--- | 562 |  |  |
| TRINITY_DN22308_c2_g2_i1 | ACT domain-containing ACR9-like | 1965 | 0 | 84.15 |
| TRINITY_DN22308_c2_g3_i1 | mechanosensitive ion channel 10-like | 3283 | 0 | 77.85 |
| TRINITY_DN22308_c2_g4_i1 | ACT domain-containing ACR9-like | 1478 | 0 | 85 |
| TRINITY_DN2230_c0_g2_i1 | PREDICTED: uncharacterized protein LOC104907537 isoform X1 | 1499 | 5.77E-164 | 76.35 |
| TRINITY_DN22310_c2_g2_i1 | oligouridylate-binding 1B | 1589 | 0 | 90.2 |
| TRINITY_DN22310_c2_g2_i3 | oligouridylate-binding 1B | 1675 | 0 | 90.25 |
| TRINITY_DN22324_c0_g1_i1 | RNA pseudouridine synthase chloroplastic | 1670 | 0 | 84.5 |
| TRINITY_DN22324_c0_g1_i4 | RNA pseudouridine synthase chloroplastic | 1622 | 0 | 84.5 |
| TRINITY_DN22325_c0_g1_i1 | Zinc RING FYVE PHD-type | 4080 | 0 | 81.05 |
| TRINITY_DN22329_c0_g2_i1 | Pseudouridine-5 -phosphate glycosidase | 1190 | 4.81E-141 | 89.8 |
| TRINITY_DN22329_c0_g2_i2 | pseudouridine-5 -phosphate glycosidase | 1454 | 0 | 87.3 |
| TRINITY_DN22329_c0_g2_i4 | pseudouridine-5 -phosphate glycosidase | 1501 | 2.16E-155 | 89.65 |
| TRINITY_DN22329_c0_g2_i5 | Pseudouridine-5 -phosphate glycosidase | 1143 | 1.68E-178 | 87.15 |
| TRINITY_DN22332_c1_g3_i1 | ---NA--- | 313 |  |  |
| TRINITY_DN22352_c6_g17_i1 | ---NA--- | 201 |  |  |
| TRINITY_DN22353_c0_g10_i1 | ---NA--- | 749 |  |  |
| TRINITY_DN22353_c0_g12_i1 | ---NA--- | 202 |  |  |
| TRINITY_DN22353_c0_g13_i1 | ---NA--- | 752 |  |  |
| TRINITY_DN22353_c0_g15_i1 | ---NA--- | 750 |  |  |
| TRINITY_DN22353_c0_g16_i1 | ---NA--- | 747 |  |  |
| TRINITY_DN22353_c0_g2_i1 | ---NA--- | 758 |  |  |
| TRINITY_DN22353_c0_g3_i1 | ---NA--- | 751 |  |  |
| TRINITY_DN22353_c0_g4_i1 | ---NA--- | 755 |  |  |
| TRINITY_DN22353_c0_g5_i1 | ---NA--- | 756 |  |  |
| TRINITY_DN22353_c0_g6_i1 | ---NA--- | 748 |  |  |
| TRINITY_DN22353_c0_g7_i1 | ---NA--- | 746 |  |  |
| TRINITY_DN22353_c0_g8_i1 | blue-light photoreceptor PHR2 | 1936 | 0 | 83.15 |
| TRINITY_DN22353_c0_g9_i1 | ---NA--- | 783 |  |  |
| TRINITY_DN22355_c0_g1_i1 | probable mitochondrial saccharopine dehydrogenase-like oxidoreductase At5g39410 | 1648 | 0 | 81 |
| TRINITY_DN22355_c0_g2_i1 | ---NA--- | 203 |  |  |
| TRINITY_DN22355_c0_g3_i1 | ---NA--- | 202 |  |  |
| TRINITY_DN22355_c2_g1_i1 | ---NA--- | 370 |  |  |
| TRINITY_DN22355_c2_g1_i2 | ---NA--- | 387 |  |  |
| TRINITY_DN22355_c2_g3_i1 | ---NA--- | 445 |  |  |
| TRINITY_DN22355_c3_g4_i1 | ---NA--- | 302 |  |  |
| TRINITY_DN22356_c0_g1_i10 | CLP1 homolog | 1900 | 0 | 90.6 |
| TRINITY_DN22356_c0_g1_i12 | CLP1 homolog | 1810 | 0 | 90.6 |
| TRINITY_DN22356_c0_g1_i13 | CLP1 homolog | 1782 | 0 | 90.6 |
| TRINITY_DN22356_c0_g1_i2 | CLP1 homolog | 1797 | 0 | 90.6 |
| TRINITY_DN22356_c0_g1_i3 | CLP1 homolog | 1808 | 0 | 90.6 |
| TRINITY_DN22356_c0_g1_i4 | CLP1 homolog | 1836 | 0 | 90.6 |
| TRINITY_DN22356_c0_g1_i5 | CLP1 homolog | 1928 | 0 | 90.6 |
| TRINITY_DN22356_c0_g1_i6 | CLP1 homolog | 1947 | 0 | 90.6 |
| TRINITY_DN22356_c0_g1_i7 | CLP1 homolog | 1825 | 0 | 90.6 |
| TRINITY_DN22356_c0_g1_i8 | CLP1 homolog | 1919 | 0 | 90.6 |
| TRINITY_DN22356_c1_g1_i1 | ---NA--- | 213 |  |  |
| TRINITY_DN22357_c0_g1_i1 | ---NA--- | 561 |  |  |
| TRINITY_DN22357_c0_g1_i2 | ---NA--- | 420 |  |  |
| TRINITY_DN22357_c0_g1_i3 | ---NA--- | 738 |  |  |
| TRINITY_DN22357_c0_g1_i4 | ---NA--- | 661 |  |  |
| TRINITY_DN22357_c0_g1_i5 | ---NA--- | 520 |  |  |
| TRINITY_DN22357_c0_g1_i6 | ---NA--- | 638 |  |  |
| TRINITY_DN22361_c0_g1_i3 | potassium transporter 12 isoform X1 | 2682 | 0 | 87.6 |
| TRINITY_DN22362_c0_g1_i1 | hypothetical protein SOVF_125740 | 1562 | 0 | 68.55 |
| TRINITY_DN22363_c0_g1_i1 | PREDICTED: uncharacterized protein LOC104907615 | 1731 | 3.10E-103 | 54.35 |
| TRINITY_DN22363_c0_g1_i2 | PREDICTED: uncharacterized protein LOC104907615 | 1735 | 6.86E-101 | 54.5 |
| TRINITY_DN22367_c0_g1_i1 | PREDICTED: uncharacterized protein LOC100266406 | 7725 | 0 | 60.45 |
| TRINITY_DN22371_c0_g1_i1 | dihydroxy-acid chloroplastic | 1555 | 0 | 95.55 |
| TRINITY_DN22373_c2_g1_i2 | ATPase family AAA domain-containing 1 | 4326 | 0 | 75 |
| TRINITY_DN22373_c2_g1_i4 | AAA-type ATPase family isoform 1 | 4311 | 0 | 73.7 |
| TRINITY_DN22373_c2_g1_i6 | AAA-type ATPase family isoform 1 | 4312 | 0 | 83.95 |
| TRINITY_DN22373_c2_g1_i7 | AAA-type ATPase family isoform 1 | 4312 | 0 | 83.35 |
| TRINITY_DN22378_c1_g4_i3 | CASP | 1275 | 1.38E-26 | 92.3 |
| TRINITY_DN22378_c1_g4_i4 | CASP | 1773 | 1.02E-21 | 92.2 |
| TRINITY_DN22378_c1_g4_i5 | CASP | 1231 | 6.46E-25 | 92.85 |
| TRINITY_DN22378_c1_g4_i6 | CASP | 1334 | 1.05E-24 | 92.85 |
| TRINITY_DN22378_c1_g4_i7 | CASP | 2273 | 1.08E-129 | 91.6 |
| TRINITY_DN22378_c1_g4_i8 | CASP | 1172 | 1.52E-22 | 92.3 |
| TRINITY_DN22378_c1_g4_i9 | CASP | 1832 | 4.56E-24 | 92.75 |
| TRINITY_DN22383_c1_g2_i2 | ---NA--- | 882 |  |  |
| TRINITY_DN22383_c1_g2_i3 | ---NA--- | 269 |  |  |
| TRINITY_DN22383_c1_g2_i4 | ---NA--- | 531 |  |  |
| TRINITY_DN22383_c2_g2_i1 | ---NA--- | 232 |  |  |
| TRINITY_DN22383_c2_g2_i2 | serine threonine- phosphatase 7 long form homolog | 670 | 8.35E-35 | 54.55 |
| TRINITY_DN22383_c2_g2_i3 | ---NA--- | 603 |  |  |
| TRINITY_DN22390_c0_g1_i1 | probable CCR4-associated factor 1 homolog 7 | 720 | 7.33E-105 | 90.35 |
| TRINITY_DN22390_c0_g2_i1 | probable CCR4-associated factor 1 homolog 7 | 1407 | 9.20E-115 | 89 |
| TRINITY_DN22390_c0_g2_i2 | probable CCR4-associated factor 1 homolog 7 | 871 | 4.72E-111 | 90.65 |
| TRINITY_DN22390_c0_g2_i3 | probable CCR4-associated factor 1 homolog 7 | 1406 | 1.22E-177 | 92.1 |
| TRINITY_DN22390_c0_g2_i4 | probable CCR4-associated factor 1 homolog 7 | 1301 | 2.23E-176 | 91.4 |
| TRINITY_DN22390_c0_g2_i5 | probable CCR4-associated factor 1 homolog 7 | 402 | 2.83E-10 | 83.4 |
| TRINITY_DN22391_c0_g2_i1 | (-)-germacrene D synthase-like | 981 | 3.36E-28 | 68.2 |
| TRINITY_DN22392_c0_g1_i1 | cellulose synthase D3 | 1085 | 0 | 91.6 |
| TRINITY_DN22392_c0_g2_i1 | cellulose synthase D3 | 3038 | 0 | 92.6 |
| TRINITY_DN22392_c1_g1_i1 | SENSITIVE TO PROTON RHIZOTOXICITY 1-like | 430 | 5.70E-44 | 74.6 |
| TRINITY_DN22392_c1_g1_i3 | SENSITIVE TO PROTON RHIZOTOXICITY 1-like | 1047 | 3.03E-94 | 67.75 |
| TRINITY_DN22392_c1_g1_i4 | SENSITIVE TO PROTON RHIZOTOXICITY 1-like | 966 | 7.65E-101 | 67.7 |
| TRINITY_DN22394_c1_g1_i1 | endoglucanase 6 | 2483 | 0 | 83.9 |
| TRINITY_DN22394_c1_g1_i2 | endoglucanase 6 | 2483 | 0 | 84.2 |
| TRINITY_DN22394_c1_g2_i1 | endoglucanase 6 | 814 | 2.58E-137 | 89.6 |
| TRINITY_DN22394_c2_g1_i1 | DNA-directed RNA polymerase subunit 10 | 697 | 8.77E-45 | 98.25 |
| TRINITY_DN22399_c0_g2_i1 | ---NA--- | 232 |  |  |
| TRINITY_DN22399_c0_g4_i1 | ---NA--- | 201 |  |  |
| TRINITY_DN22399_c0_g5_i1 | ---NA--- | 275 |  |  |
| TRINITY_DN22399_c0_g6_i1 | ---NA--- | 297 |  |  |
| TRINITY_DN22399_c0_g7_i1 | ---NA--- | 204 |  |  |
| TRINITY_DN2239_c0_g1_i1 | psbP chloroplastic | 977 | 7.57E-119 | 78.9 |
| TRINITY_DN2239_c0_g2_i1 | psbP chloroplastic | 978 | 6.38E-76 | 74.7 |
| TRINITY_DN22414_c28_g2_i1 | RNA polymerase sigma factor sigA | 2201 | 0 | 79.8 |
| TRINITY_DN22417_c0_g2_i1 | disease resistance RGA1 isoform X1 | 1304 | 3.26E-48 | 50.3 |
| TRINITY_DN22417_c0_g2_i3 | ---NA--- | 476 |  |  |
| TRINITY_DN22418_c0_g1_i1 | serine threonine- kinase STY46-like | 1968 | 0 | 93.55 |
| TRINITY_DN22418_c0_g1_i2 | serine threonine- kinase STY46-like | 763 | 4.85E-88 | 90.85 |
| TRINITY_DN22418_c0_g1_i3 | serine threonine- kinase STY46-like | 1968 | 0 | 93.3 |
| TRINITY_DN22418_c2_g1_i1 | ---NA--- | 341 |  |  |
| TRINITY_DN22420_c1_g3_i1 | calcium-dependent kinase 26 | 2478 | 0 | 88.45 |
| TRINITY_DN22422_c0_g2_i3 | L-2-hydroxyglutarate mitochondrial | 1927 | 0 | 82.45 |
| TRINITY_DN22422_c0_g2_i4 | L-2-hydroxyglutarate mitochondrial | 1903 | 0 | 84 |
| TRINITY_DN22423_c0_g1_i1 | AP-2 complex subunit mu | 1905 | 0 | 97.5 |
| TRINITY_DN22424_c3_g2_i1 | homeobox-leucine zipper MERISTEM L1-like | 842 | 9.19E-50 | 89.05 |
| TRINITY_DN22426_c0_g1_i1 | serine threonine- kinase 38-like isoform X2 | 363 | 7.25E-50 | 91.9 |
| TRINITY_DN22426_c0_g1_i2 | serine threonine- kinase tricorner-like | 2000 | 0 | 90.1 |
| TRINITY_DN22426_c0_g1_i3 | serine threonine- kinase tricorner-like | 2058 | 0 | 89.7 |
| TRINITY_DN22426_c0_g1_i4 | serine threonine- kinase tricorner-like | 2160 | 0 | 89.25 |
| TRINITY_DN22426_c0_g1_i5 | serine threonine- kinase tricorner-like | 2102 | 0 | 89.9 |
| TRINITY_DN22427_c4_g1_i1 | ---NA--- | 234 |  |  |
| TRINITY_DN22428_c0_g2_i4 | WD repeat-containing 91 homolog isoform X1 | 1351 | 6.37E-173 | 71.85 |
| TRINITY_DN22438_c1_g1_i1 | glycine-rich RNA-binding mitochondrial-like isoform X2 | 1550 | 1.62E-36 | 84.95 |
| TRINITY_DN22438_c1_g1_i15 | BOI-related E3 ubiquitin- ligase 1-like | 980 | 1.26E-25 | 65.7 |
| TRINITY_DN22438_c1_g1_i2 | ---NA--- | 335 |  |  |
| TRINITY_DN22438_c1_g1_i4 | glycine-rich RNA-binding mitochondrial-like isoform X1 | 768 | 2.44E-26 | 82.65 |
| TRINITY_DN22438_c1_g1_i6 | BOI-related E3 ubiquitin- ligase 1-like | 1517 | 1.52E-24 | 65.7 |
| TRINITY_DN22438_c1_g1_i8 | glycine-rich RNA-binding mitochondrial-like isoform X2 | 1013 | 6.14E-38 | 84.95 |
| TRINITY_DN22438_c1_g4_i7 | ---NA--- | 480 |  |  |
| TRINITY_DN22438_c1_g4_i9 | ---NA--- | 629 |  |  |
| TRINITY_DN22452_c0_g2_i1 | RING finger 141 | 1390 | 3.73E-149 | 79.8 |
| TRINITY_DN22452_c0_g2_i3 | RING finger 141 | 1291 | 1.14E-155 | 92.25 |
| TRINITY_DN22452_c0_g2_i4 | RING finger 141 | 1367 | 1.28E-158 | 91.9 |
| TRINITY_DN22452_c1_g1_i1 | RING finger 141 | 623 | 1.07E-106 | 92.8 |
| TRINITY_DN22452_c2_g1_i1 | eukaryotic translation initiation factor 3 subunit C-like | 3061 | 0 | 81.05 |
| TRINITY_DN22452_c2_g1_i2 | eukaryotic translation initiation factor 3 subunit C-like | 3048 | 0 | 79.85 |
| TRINITY_DN22453_c0_g2_i1 | ENHANCED DISEASE RESISTANCE 4 | 2786 | 0 | 62.8 |
| TRINITY_DN22453_c0_g3_i1 | ENHANCED DISEASE RESISTANCE 4 | 1011 | 2.95E-126 | 63.95 |
| TRINITY_DN22453_c1_g1_i1 | ---NA--- | 442 |  |  |
| TRINITY_DN22455_c0_g1_i2 | RNA-binding 25-like isoform X2 | 2720 | 2.46E-97 | 69.5 |
| TRINITY_DN22455_c0_g1_i3 | ---NA--- | 422 |  |  |
| TRINITY_DN22458_c0_g1_i1 | ---NA--- | 1236 |  |  |
| TRINITY_DN22458_c0_g2_i1 | ---NA--- | 431 |  |  |
| TRINITY_DN22458_c0_g4_i1 | uncharacterized CRM domain-containing chloroplastic | 1904 | 1.93E-171 | 75.65 |
| TRINITY_DN22463_c0_g1_i3 | twinkle homolog chloroplastic mitochondrial | 2518 | 0 | 85.85 |
| TRINITY_DN22463_c0_g2_i1 | twinkle homolog chloroplastic mitochondrial isoform X2 | 2277 | 0 | 79.4 |
| TRINITY_DN22464_c0_g1_i1 | eukaryotic translation initiation factor | 2905 | 0 | 79.45 |
| TRINITY_DN22464_c0_g1_i2 | eukaryotic translation initiation factor | 3029 | 0 | 79.45 |
| TRINITY_DN22464_c0_g2_i1 | eukaryotic translation initiation factor | 2908 | 0 | 80.65 |
| TRINITY_DN22464_c1_g1_i1 | telomere repeat-binding 5 isoform X1 | 2943 | 0 | 64.4 |
| TRINITY_DN22466_c0_g1_i1 | type IV inositol polyphosphate 5-phosphatase 3 isoform X2 | 2238 | 0 | 76.95 |
| TRINITY_DN22466_c0_g1_i2 | type IV inositol polyphosphate 5-phosphatase 3 isoform X3 | 2283 | 0 | 78.1 |
| TRINITY_DN22467_c1_g1_i2 | 39S ribosomal mitochondrial-like | 1170 | 4.82E-152 | 77.3 |
| TRINITY_DN22467_c1_g1_i5 | 50S ribosomal chloroplastic-like | 953 | 1.19E-47 | 89.15 |
| TRINITY_DN22467_c1_g1_i6 | 50S ribosomal L22-like | 423 | 8.56E-37 | 91.25 |
| TRINITY_DN22468_c0_g2_i10 | ---NA--- | 559 |  |  |
| TRINITY_DN22468_c0_g2_i13 | ---NA--- | 471 |  |  |
| TRINITY_DN22473_c1_g1_i2 | carbon catabolite repressor 4 homolog 1-like | 2075 | 0 | 58.1 |
| TRINITY_DN22473_c2_g4_i1 | PREDICTED: uncharacterized protein LOC104892385 | 942 | 5.44E-81 | 57.7 |
| TRINITY_DN22476_c1_g1_i1 | exocyst complex component EXO84B | 317 | 1.02E-22 | 87.3 |
| TRINITY_DN22487_c0_g1_i1 | ---NA--- | 260 |  |  |
| TRINITY_DN22487_c0_g4_i1 | ---NA--- | 273 |  |  |
| TRINITY_DN22487_c0_g5_i1 | ---NA--- | 260 |  |  |
| TRINITY_DN22488_c0_g1_i2 | decapping nuclease DXO chloroplastic | 1587 | 0 | 90.2 |
| TRINITY_DN22489_c0_g1_i4 | 2-carboxy-1,4-naphthoquinone chloroplastic | 1544 | 2.52E-143 | 79.55 |
| TRINITY_DN22492_c0_g1_i1 | mitogen-activated kinase kinase kinase NPK1 isoform X1 | 2343 | 0 | 71.2 |
| TRINITY_DN22492_c0_g1_i2 | mitogen-activated kinase kinase kinase NPK1 isoform X1 | 2442 | 0 | 71.2 |
| TRINITY_DN22497_c0_g1_i1 | DUF21 domain-containing chloroplastic | 2219 | 0 | 79.6 |
| TRINITY_DN22497_c0_g1_i4 | DUF21 domain-containing chloroplastic | 2200 | 0 | 84.6 |
| TRINITY_DN22506_c0_g1_i1 | squamous cell carcinoma antigen recognized by T-cells 3 | 2849 | 0 | 72.95 |
| TRINITY_DN22507_c0_g1_i2 | BTB POZ domain-containing At3g05675-like | 1473 | 0 | 75.25 |
| TRINITY_DN22507_c1_g1_i1 | DCD (Development and Cell Death) domain | 1642 | 8.83E-52 | 71.5 |
| TRINITY_DN22507_c1_g1_i2 | DCD (Development and Cell Death) domain | 1656 | 7.56E-55 | 71.5 |
| TRINITY_DN22507_c1_g1_i3 | DCD (Development and Cell Death) domain | 1549 | 1.14E-51 | 71.5 |
| TRINITY_DN22507_c1_g1_i4 | DCD (Development and Cell Death) domain | 1749 | 2.03E-51 | 71.5 |
| TRINITY_DN22507_c1_g2_i1 | ---NA--- | 566 |  |  |
| TRINITY_DN22510_c1_g2_i2 | PREDICTED: uncharacterized protein LOC104883032 isoform X1 | 1013 | 5.18E-50 | 55.85 |
| TRINITY_DN22510_c1_g2_i3 | PREDICTED: uncharacterized protein LOC104883032 isoform X1 | 1076 | 9.29E-50 | 56.3 |
| TRINITY_DN22510_c1_g2_i4 | PREDICTED: uncharacterized protein LOC104883032 isoform X4 | 657 | 9.92E-36 | 59.75 |
| TRINITY_DN22514_c1_g2_i1 | ---NA--- | 318 |  |  |
| TRINITY_DN22515_c0_g1_i3 | EH domain-containing 1 | 1907 | 0 | 90.25 |
| TRINITY_DN22516_c0_g1_i1 | ubiquitin-like-specific protease 1D | 2116 | 1.50E-152 | 66.45 |
| TRINITY_DN22517_c0_g1_i1 | kinesin KIN- mitochondrial isoform X1 | 3606 | 0 | 84.15 |
| TRINITY_DN22517_c0_g2_i1 | kinesin KIN- mitochondrial isoform X1 | 1549 | 0 | 91.95 |
| TRINITY_DN22519_c0_g1_i1 | 40S ribosomal S15a-5 | 739 | 7.97E-70 | 85.55 |
| TRINITY_DN22519_c0_g1_i3 | 40S ribosomal S15a-5 | 732 | 7.17E-70 | 85.55 |
| TRINITY_DN2251_c0_g1_i1 | transcription factor FAMA isoform X2 | 1233 | 1.58E-107 | 63.5 |
| TRINITY_DN2251_c1_g1_i1 | glycerol-3-phosphate acyltransferase 3 | 891 | 1.24E-85 | 85.7 |
| TRINITY_DN22528_c0_g1_i1 | SWI SNF complex component SNF12 homolog | 1849 | 0 | 88.55 |
| TRINITY_DN22529_c2_g1_i3 | ---NA--- | 729 |  |  |
| TRINITY_DN22530_c0_g1_i1 | Rpr2 Rpp21 subunit | 1134 | 4.05E-96 | 62 |
| TRINITY_DN22535_c2_g1_i1 | FRA10AC1 | 893 | 1.31E-113 | 83.65 |
| TRINITY_DN22535_c2_g1_i2 | ---NA--- | 565 |  |  |
| TRINITY_DN22535_c2_g1_i4 | FRA10AC1 | 679 | 1.45E-78 | 83.15 |
| TRINITY_DN22536_c2_g2_i5 | F-box FBD LRR-repeat At1g13570 isoform X1 | 1411 | 9.73E-101 | 52.95 |
| TRINITY_DN22539_c1_g10_i1 | ---NA--- | 254 |  |  |
| TRINITY_DN22539_c1_g11_i2 | ---NA--- | 285 |  |  |
| TRINITY_DN22539_c1_g2_i1 | ---NA--- | 285 |  |  |
| TRINITY_DN22539_c1_g3_i1 | ---NA--- | 246 |  |  |
| TRINITY_DN22539_c1_g4_i1 | ---NA--- | 243 |  |  |
| TRINITY_DN22539_c1_g7_i1 | 50S ribosomal L27 | 1102 | 1.28E-81 | 90.5 |
| TRINITY_DN22539_c1_g7_i3 | 50S ribosomal L27 | 1113 | 1.42E-81 | 90.5 |
| TRINITY_DN22539_c2_g2_i1 | B3 domain-containing Os03g0621600 | 1359 | 4.83E-27 | 50.4 |
| TRINITY_DN22539_c2_g2_i10 | B3 domain-containing Os11g0197600-like isoform X1 | 1325 | 2.87E-19 | 53.9 |
| TRINITY_DN22539_c2_g2_i11 | B3 domain-containing Os03g0621600 | 1679 | 4.83E-23 | 50.4 |
| TRINITY_DN22539_c2_g2_i12 | B3 domain-containing Os03g0621600 | 1648 | 4.60E-23 | 50.4 |
| TRINITY_DN22539_c2_g2_i2 | B3 domain-containing Os11g0197600-like isoform X1 | 1355 | 3.10E-19 | 53.9 |
| TRINITY_DN22539_c2_g2_i3 | B3 domain-containing Os11g0197600-like isoform X1 | 1294 | 2.63E-19 | 53.9 |
| TRINITY_DN22539_c2_g2_i4 | B3 domain-containing Os03g0621600 | 1420 | 2.77E-23 | 50.4 |
| TRINITY_DN22539_c2_g2_i5 | B3 domain-containing Os11g0197600-like isoform X1 | 1386 | 3.34E-19 | 53.9 |
| TRINITY_DN22539_c2_g2_i6 | B3 domain-containing Os03g0621600 | 1536 | 3.69E-23 | 50.4 |
| TRINITY_DN22539_c2_g2_i7 | B3 domain-containing Os03g0621600 | 1390 | 2.51E-23 | 50.4 |
| TRINITY_DN22539_c2_g2_i8 | B3 domain-containing Os03g0621600 | 1451 | 3.01E-23 | 50.4 |
| TRINITY_DN22539_c2_g2_i9 | B3 domain-containing Os03g0621600 | 1505 | 7.25E-27 | 50.4 |
| TRINITY_DN22541_c0_g1_i1 | GTP-binding ERG | 1759 | 0 | 77.55 |
| TRINITY_DN22541_c0_g1_i2 | GTP-binding ERG | 1220 | 1.10E-127 | 77.3 |
| TRINITY_DN22541_c0_g1_i3 | GTP-binding ERG | 1892 | 3.43E-180 | 77.55 |
| TRINITY_DN22541_c0_g1_i4 | GTP-binding ERG | 1852 | 2.12E-180 | 77.55 |
| TRINITY_DN22543_c0_g1_i1 | ---NA--- | 778 |  |  |
| TRINITY_DN22543_c0_g1_i2 | ---NA--- | 923 |  |  |
| TRINITY_DN22543_c0_g1_i3 | ---NA--- | 720 |  |  |
| TRINITY_DN22543_c0_g2_i1 | ---NA--- | 680 |  |  |
| TRINITY_DN22543_c0_g3_i1 | ---NA--- | 389 |  |  |
| TRINITY_DN22546_c5_g7_i1 | ---NA--- | 401 |  |  |
| TRINITY_DN22546_c5_g7_i2 | ---NA--- | 274 |  |  |
| TRINITY_DN22546_c5_g7_i3 | ---NA--- | 708 |  |  |
| TRINITY_DN22547_c0_g10_i1 | ---NA--- | 242 |  |  |
| TRINITY_DN22547_c0_g12_i1 | ---NA--- | 223 |  |  |
| TRINITY_DN22547_c0_g13_i1 | ---NA--- | 225 |  |  |
| TRINITY_DN22547_c0_g1_i1 | ---NA--- | 237 |  |  |
| TRINITY_DN22547_c0_g2_i1 | ---NA--- | 221 |  |  |
| TRINITY_DN22547_c0_g3_i1 | ---NA--- | 224 |  |  |
| TRINITY_DN22547_c0_g4_i1 | ---NA--- | 222 |  |  |
| TRINITY_DN22547_c0_g5_i1 | ---NA--- | 235 |  |  |
| TRINITY_DN22547_c0_g6_i1 | ---NA--- | 231 |  |  |
| TRINITY_DN22547_c0_g7_i1 | ---NA--- | 220 |  |  |
| TRINITY_DN22547_c0_g8_i1 | ---NA--- | 226 |  |  |
| TRINITY_DN22547_c0_g9_i1 | two-component response regulator | 1545 | 1.22E-26 | 53.55 |
| TRINITY_DN22547_c0_g9_i2 | two-component response regulator | 785 | 6.79E-30 | 57.3 |
| TRINITY_DN22547_c0_g9_i3 | two-component response regulator | 859 | 1.17E-27 | 58.9 |
| TRINITY_DN22547_c1_g1_i1 | ---NA--- | 324 |  |  |
| TRINITY_DN22548_c0_g1_i1 | aminodeoxychorismate chloroplastic | 3437 | 0 | 75.65 |
| TRINITY_DN22548_c0_g1_i2 | aminodeoxychorismate chloroplastic | 3426 | 0 | 80.75 |
| TRINITY_DN22551_c0_g1_i1 | Calcineurin-like metallo-phosphoesterase superfamily | 363 | 6.54E-20 | 70.15 |
| TRINITY_DN22551_c0_g3_i12 | Metallophos domain-containing | 1748 | 0 | 74.5 |
| TRINITY_DN22551_c0_g3_i5 | Metallophos domain-containing | 1770 | 0 | 73.25 |
| TRINITY_DN22551_c0_g3_i6 | Metallophos domain-containing | 1832 | 0 | 73.4 |
| TRINITY_DN22551_c0_g3_i7 | Metallophos domain-containing | 1795 | 0 | 71.2 |
| TRINITY_DN22551_c0_g3_i8 | Metallophos domain-containing | 1841 | 0 | 73.4 |
| TRINITY_DN22552_c1_g1_i11 | paired amphipathic helix Sin3-like 4 isoform X1 | 4658 | 0 | 73.7 |
| TRINITY_DN22552_c1_g1_i12 | paired amphipathic helix Sin3-like 4 isoform X1 | 4864 | 0 | 75.2 |
| TRINITY_DN22552_c1_g1_i16 | paired amphipathic helix Sin3-like 4 isoform X1 | 4658 | 0 | 73.6 |
| TRINITY_DN22552_c1_g1_i3 | paired amphipathic helix Sin3-like 4 isoform X1 | 4942 | 0 | 73.75 |
| TRINITY_DN22552_c1_g1_i9 | paired amphipathic helix Sin3-like 4 isoform X1 | 4577 | 0 | 75.2 |
| TRINITY_DN22553_c0_g3_i2 | ankyrin repeat-containing NPR4-like isoform X1 | 2200 | 3.79E-102 | 56.85 |
| TRINITY_DN22555_c0_g11_i1 | ---NA--- | 274 |  |  |
| TRINITY_DN22555_c0_g3_i1 | ---NA--- | 337 |  |  |
| TRINITY_DN22555_c0_g3_i2 | ---NA--- | 333 |  |  |
| TRINITY_DN22555_c0_g7_i1 | ---NA--- | 281 |  |  |
| TRINITY_DN22556_c0_g1_i1 | RIK isoform X2 | 640 | 5.99E-17 | 54.05 |
| TRINITY_DN22556_c0_g1_i2 | RIK isoform X2 | 2212 | 4.02E-86 | 71.65 |
| TRINITY_DN22556_c0_g1_i3 | ---NA--- | 941 |  |  |
| TRINITY_DN22556_c0_g1_i6 | RIK isoform X2 | 2157 | 7.03E-77 | 60.95 |
| TRINITY_DN22556_c0_g1_i9 | RIK isoform X2 | 2165 | 7.33E-77 | 76.3 |
| TRINITY_DN2255_c0_g1_i1 | ---NA--- | 589 |  |  |
| TRINITY_DN2255_c0_g1_i2 | ---NA--- | 504 |  |  |
| TRINITY_DN22560_c0_g1_i1 | pentatricopeptide repeat-containing At1g25360 | 1357 | 2.70E-158 | 83.85 |
| TRINITY_DN22560_c0_g1_i2 | ---NA--- | 868 |  |  |
| TRINITY_DN22563_c4_g3_i2 | ---NA--- | 646 |  |  |
| TRINITY_DN22567_c0_g3_i1 | ---NA--- | 219 |  |  |
| TRINITY_DN22567_c0_g4_i1 | dual specificity kinase shkC | 2027 | 0 | 87 |
| TRINITY_DN22567_c0_g4_i2 | dual specificity kinase shkC | 2062 | 0 | 87 |
| TRINITY_DN22567_c0_g4_i3 | dual specificity kinase shkC-like | 1962 | 0 | 87.05 |
| TRINITY_DN22567_c0_g4_i4 | dual specificity kinase shkC-like | 1997 | 0 | 87.05 |
| TRINITY_DN22572_c0_g2_i2 | probable serine threonine- kinase At1g54610 isoform X1 | 1869 | 0 | 91.7 |
| TRINITY_DN22572_c0_g2_i3 | probable serine threonine- kinase At1g54610 isoform X1 | 1737 | 0 | 91.7 |
| TRINITY_DN22573_c1_g1_i5 | ABC transporter D family member 1 | 3939 | 0 | 84.05 |
| TRINITY_DN22573_c1_g1_i6 | ABC transporter D family member 1 | 4013 | 0 | 89.45 |
| TRINITY_DN22576_c1_g1_i1 | UDP-glucuronate 4-epimerase 6 | 278 | 2.71E-24 | 87.7 |
| TRINITY_DN22581_c0_g1_i1 | hypothetical protein SOVF_165580 | 1352 | 3.12E-126 | 69.85 |
| TRINITY_DN22582_c0_g1_i6 | E3 ubiquitin- ligase COP1-like isoform X1 | 2065 | 0 | 77.4 |
| TRINITY_DN22582_c0_g1_i7 | E3 ubiquitin- ligase COP1-like isoform X1 | 2036 | 0 | 77 |
| TRINITY_DN22583_c0_g1_i3 | Cys Met pyridoxal phosphate-dependent enzyme | 1645 | 0 | 93.3 |
| TRINITY_DN22583_c0_g1_i5 | cystathionine beta-lyase family | 684 | 3.52E-23 | 96.9 |
| TRINITY_DN22584_c1_g2_i1 | acetylglutamate chloroplastic | 1391 | 0 | 92.2 |
| TRINITY_DN22584_c4_g1_i1 | proline-rich cell wall | 1186 | 3.92E-75 | 71.3 |
| TRINITY_DN22584_c7_g1_i1 | sm LSM8 | 852 | 6.58E-43 | 90.55 |
| TRINITY_DN22584_c7_g1_i2 | flowering locus K homology domain | 2450 | 4.89E-144 | 78.6 |
| TRINITY_DN22584_c7_g1_i3 | flowering locus K homology domain | 2216 | 0 | 78.85 |
| TRINITY_DN22584_c7_g2_i1 | PREDICTED: uncharacterized protein LOC104887566 | 770 | 1.14E-54 | 94 |
| TRINITY_DN22584_c8_g1_i1 | chlorophyll(ide) b reductase chloroplastic | 1355 | 8.35E-172 | 78 |
| TRINITY_DN22584_c8_g1_i2 | chlorophyll(ide) b reductase chloroplastic | 1205 | 0 | 92 |
| TRINITY_DN22584_c8_g1_i4 | chlorophyll(ide) b reductase chloroplastic | 1147 | 3.45E-127 | 88.9 |
| TRINITY_DN22584_c8_g1_i5 | chlorophyll(ide) b reductase chloroplastic isoform X1 | 1120 | 3.57E-149 | 80.45 |
| TRINITY_DN22587_c0_g1_i1 | probable phosphatase 2C 52 | 2850 | 0 | 82.45 |
| TRINITY_DN22587_c0_g1_i2 | probable phosphatase 2C 52 | 1944 | 0 | 88.15 |
| TRINITY_DN22596_c0_g1_i1 | aspartate--tRNA ligase cytoplasmic-like | 1942 | 0 | 82.7 |
| TRINITY_DN22596_c0_g1_i2 | aspartate--tRNA ligase cytoplasmic-like | 2045 | 0 | 84.65 |
[truncated: 351,785 more chars]
