# Supplementary material for: Transcriptome-wide mining, characterization, and development of microsatellite markers in Lychnis kiusiana (Caryophyllaceae)
Source: BMC Plant Biol. 2019 Jan 8;19:14. doi: 10.1186/s12870-018-1621-x (PMC6325733; doi:10.1186/s12870-018-1621-x)

Figure S1. The distributions of the major repeat types in the *Lychnis kiusiana* transcriptome.

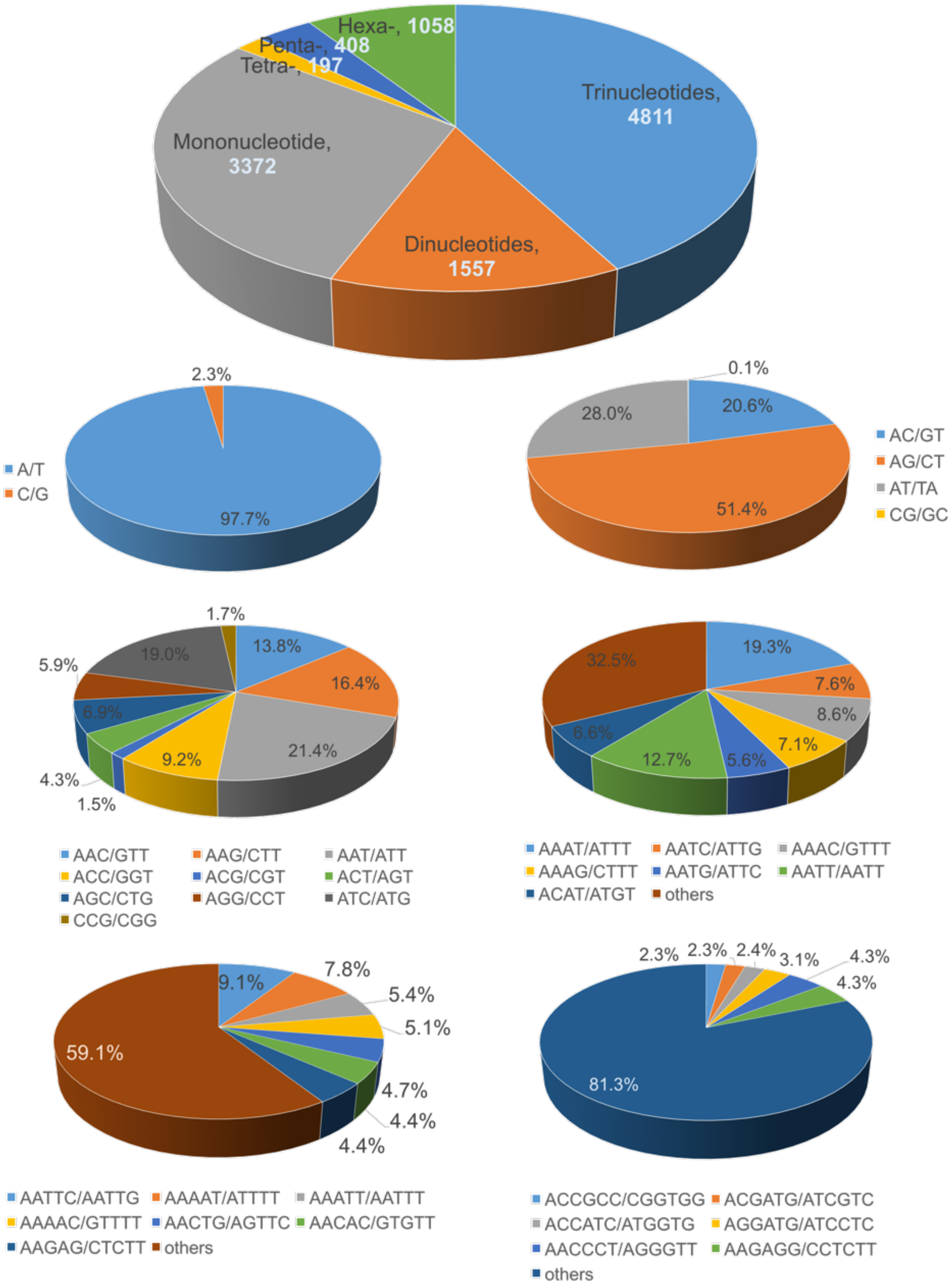

Figure S2. Box plots of the sizes of different repeat motifs. The box represents values between quartiles, solid lines extend to minimum and maximum values, outliers are shown as circles and horizontal lines in boxes show median values.

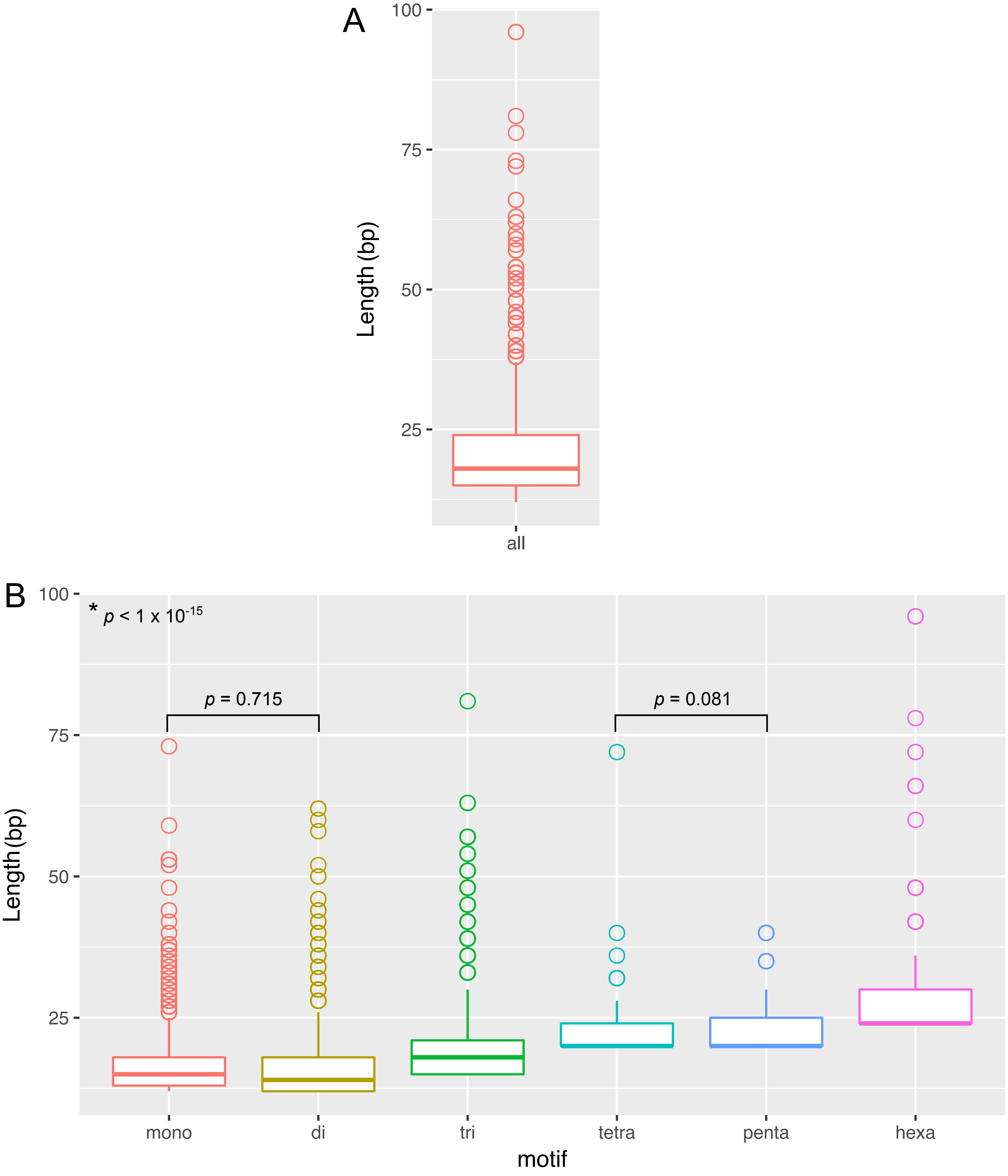

Figure S3. Box plots of the sizes of different repeat motifs in different genic regions. The box represents values between quartiles, solid lines extend to minimum and maximum values, outliers are shown as circles and horizontal lines in boxes show median values.

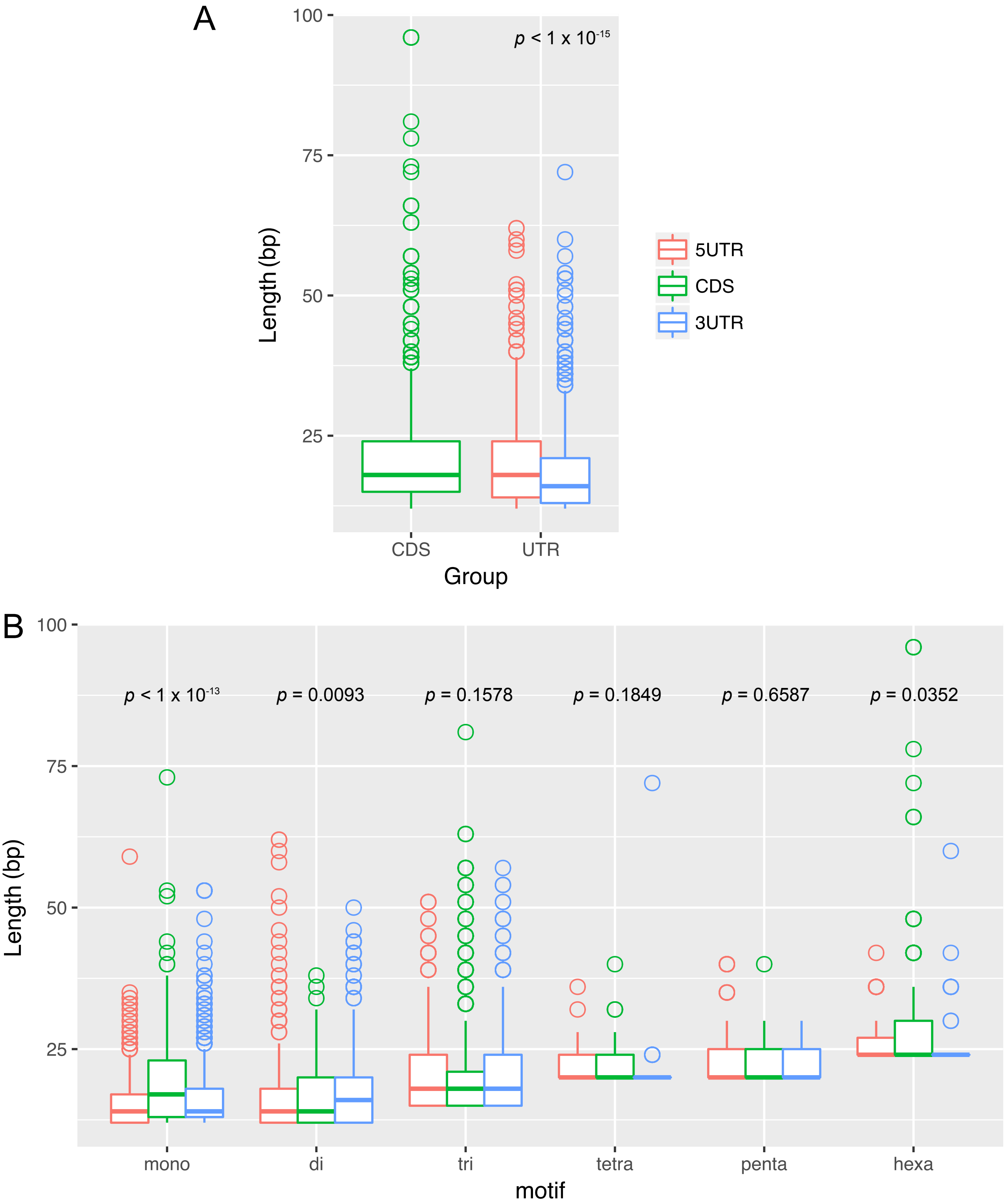

Figure S4. Linkage Disequilibrium (LD) map for the 25 transcriptomic SSR markers. Shading is based on the LD test statistic. White are significant at the p < 0.05 level after Bonferroni correction for 300 pairwise tests.

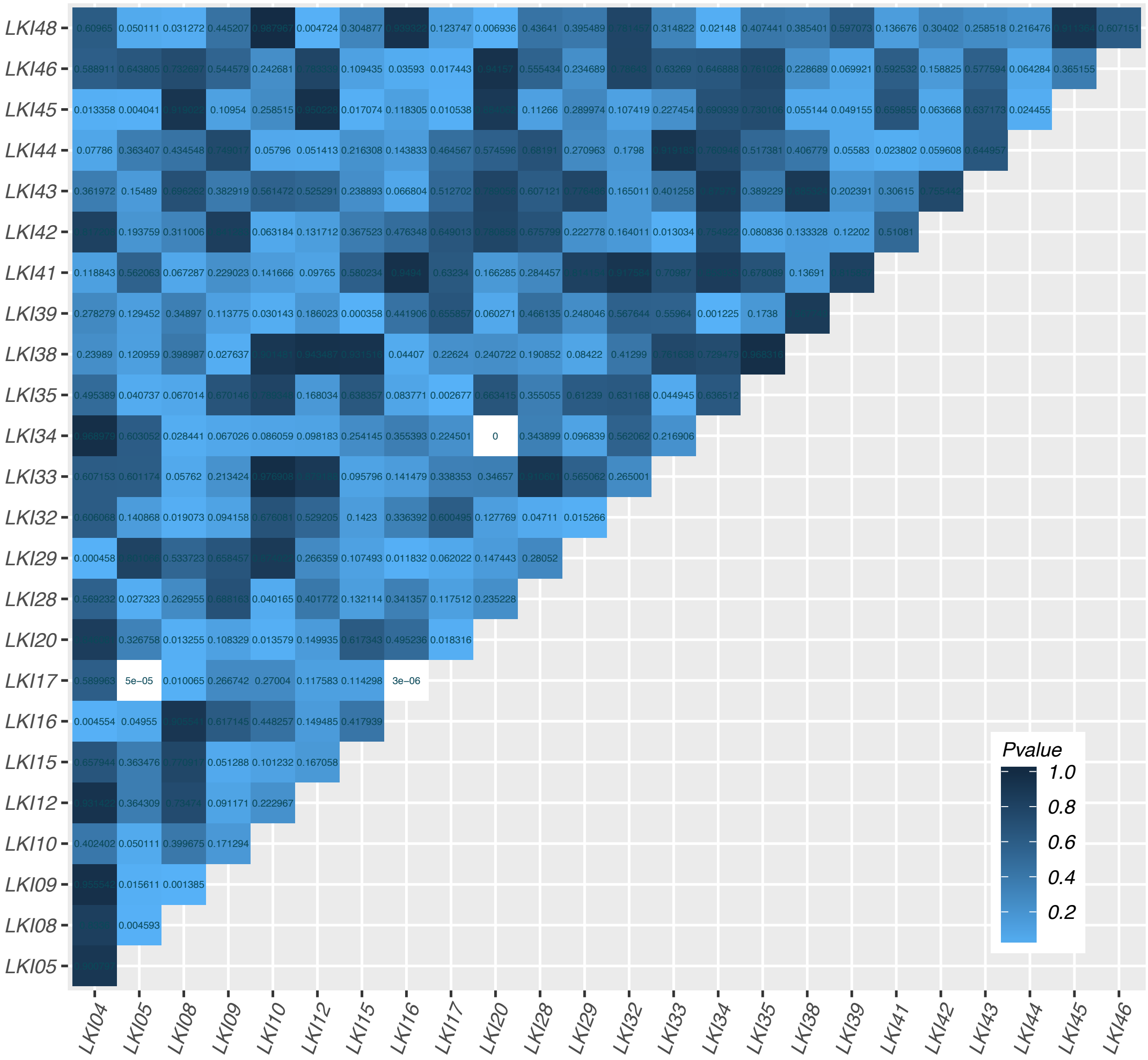

Supplement: Supplementary file 2 — Figure S1. The distributions of the major repeat types in the Lychnis kiusiana leaf transcriptome. Figure S2. Box plots of the sizes of different repeat motifs. Figure S3. Box plots of the sizes of different repeat motifs in different genic regions. Figure S4. Linkage disequilibrium (LD) map for the 25 transcriptomic SSR markers. (PDF 556 kb) [file 12870_2018_1621_MOESM2_ESM.pdf]
